# Tert promotes cardiac regenerative repair after MI through alleviating ROS-induced DNA damage response in cardiomyocyte

**DOI:** 10.1038/s41420-024-02135-8

**Published:** 2024-08-26

**Authors:** Xiaomin Wei, Yilin Zhou, Enge Shao, Xiaoran Shi, Yuan Han, Yeshen Zhang, Guoquan Wei, Hao Zheng, Senlin Huang, Yanmei Chen, Jie Sun, Yulin Liao, Wangjun Liao, Yanbing Wang, Jianping Bin, Xinzhong Li

**Affiliations:** 1 Department of Cardiology, State Key Laboratory of Organ Failure Research, Nanfang Hospital, Southern Medical University, Guangzhou, China; 2 Cardiovascular Center, the Sixth Affiliated Hospital, School of Medicine, South China University of Technology, Foshan, China; 3 Guangdong Provincial Key Laboratory of Cardiac Function and Microcirculation, Guangzhou, China; 4 Department of Cardiology, Guangdong Provincial People’s Hospital (Guangdong Academy of Medical Sciences), Southern Medical University, Guangzhou, China; 5 Department of Cardiology, Zhongshan City People’s Hospital, Zhongshan, China; 6 Department of Oncology, the Sixth Affiliated Hospital, School of Medicine, South China University of Technology, Foshan, China

**Keywords:** Cell-cycle exit, Myocardial infarction

## Abstract

Telomerase reverse transcriptase (Tert) has been found to have a protective effect on telomeric DNA, but whether it could improve the repair of reactive oxygen species (ROS)-induced DNA damage and promote myocardial regenerative repair after myocardial infarction (MI) by protecting telomeric DNA is unclear. The immunofluorescence staining with TEL-CY3 and the TeloTAGGG Telomerase PCR ELISA kit were used to show the telomere length and telomerase activity. The heart-specific Tert-deletion homozygotes were generated by using commercial Cre tool mice and flox heterozygous mice for mating. We measured the telomere length and telomerase activity of mouse cardiomyocytes (CMs) at different days of age, and the results showed that they were negatively correlated with age. Overexpressed Tert could enhance telomerase activity and lengthen telomeres, thereby repairing the DNA damage induced by ROS and promoting CM proliferation in vitro. The in vivo results indicated that enhanced Tert could significantly improve cardiac function and prognosis by alleviating CM DNA damage and promoting angiogenesis post-MI. In terms of mechanism, DNA pulldown assay was used to identify that nuclear ribonucleoprotein A2B1 (hnRNPA2B1) could be an upstream regulator of Tert in CMs. Overexpressed Tert could activate the NF-κB signaling pathway in CMs and bind to the VEGF promoter in the endothelium to increase the VEGF level. Further immunoblotting showed that Tert protected DNA from ROS-induced damage by inhibiting ATM phosphorylation and blocking the Chk1/p53/p21 pathway activation. HnRNPA2B1-activated Tert could repair the ROS-induced telomeric DNA damage to induce the cell cycle re-entry in CMs and enhance the interaction between CMs and endothelium, thus achieving cardiac regenerative repair after MI.

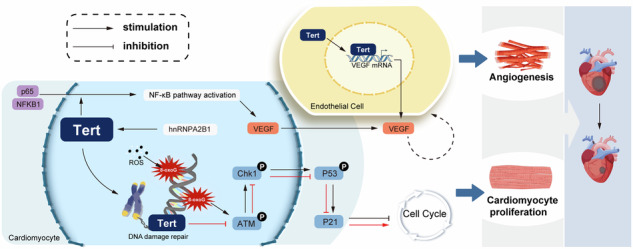

## Introduction

The limited self-renewal capacity of the adult heart could not counterbalance the loss of cardiomyocytes (CMs) after myocardial injuries such as myocardial infarction (MI) and myocarditis, which thus leads to a poor prognosis [1]. Since cell cycle arrest is the key factor leading to the loss of proliferation capacity of CMs, it is considered to be a viable way to induce adult CMs cell cycle re-entry to treat heart failure and myocardial damage [2, 3]. Several lines of evidence have demonstrated that increased ROS caused by MI or aging are substantial contributors to the DNA damage response (DDR) in CMs, which is the critical factor of cell cycle arrest [4]. Some efforts have been made to promote CM cell cycle re-entry by attenuating DDR. Recent studies have revealed that damage to telomeric DNA, which is hastened by elevated ROS, appears first and initiates the subsequent DDR, then triggers the phosphorylation of Chk2’s downstream targets through the action on the checkpoint protein ATM to block the G1/S or G2/M phase transition of CMs [5]. Telomere shortening caused by multiple factors leads to the exposure of guanine-rich repeats (GGGs) at chromosomal ends, which are more vulnerable to ROS compared with non-telomeric DNA [6]. There is a cellular intrinsic self-repair response called the base excision repair (BER) pathway, which is initiated when telomeric DNA is attacked by ROS [7]. However, this self-healing mechanism has limited capacity and fails to excise the 8-oxoG in the 3′-end successfully when more than one guanine in the GGG of telomeric DNA is oxidized to 8-oxoguanine (8-oxoG), and thus accumulating 8-oxoG occupies the position so that new base recruitment cannot be achieved, ultimately leading to the telomere shortening [8]. Therefore, it is necessary to protect telomeric DNA from ROS attacks, which helps to maintain telomere length and avoid subsequent DDR, thus achieving the regenerative repair effect after MI.

It has been established that telomerase can achieve the complementation of DNA damage-induced telomere attrition by extending telomere length, which is helpful in blocking the DDR [9]. Tert, the catalytic component of telomerase, is the rate-limiting element of telomerase activity and helps to maintain telomere homeostasis [10]. A recent study found that Tert knockdown exacerbated telomere shortening and DDRs in spermatogenic cells [11]. Moreover, recovery of Tert rescued the defect of telomere length and partially attenuated cell apoptosis. Another investigation revealed that Tert overexpression in fibroblasts could enhance genomic DNA repair and diminish DNA damage [12]. These data indicate that Tert has an important role in telomere elongation and DNA protection. Further, Tert level and telomere length reveal a tight association with cell proliferative capacity. Previous studies have demonstrated that Tert level and telomere extension are highly significant in embryonic stem cells and most cancer cells but are extremely low or undetectable in somatic cells [13–15]. More importantly, after silencing Tert, the impaired CM proliferation response in zebrafish was accompanied by the absence of CMs with long telomeres and an increased proportion of CMs exhibiting DNA damage characteristics [16], indicating that Tert has the potential to promote cardiac regeneration through elongated telomeres and DNA damage elimination. Accordingly, we anticipated that Tert could minimize ROS-induced DDR via telomere elongation and help CMs re-enter the cell cycle, thus accomplishing cardiac regenerative repair after MI.

In the present study, through gain- and loss-of-function and rescue experiments, we showed that Tert could repair telomeric DNA damage by extending telomere length and enhancing telomerase activity, thus reversing ROS-induced cell cycle arrest and promoting CM proliferation, thereby dramatically improving cardiac function after MI. Furthermore, by performing the DNA pulldown assay and related molecular biology experiments, we explored the underlying mechanism and found that Tert, upregulated by hnRNPA2B1, could block the ATM/Chk1/p53/p21 signaling pathway in DDR.

## Results

### Tert, telomere, and telomerase activity characterize cardiac development and cardiac repair after myocardial infarction

**Fig. 1:**
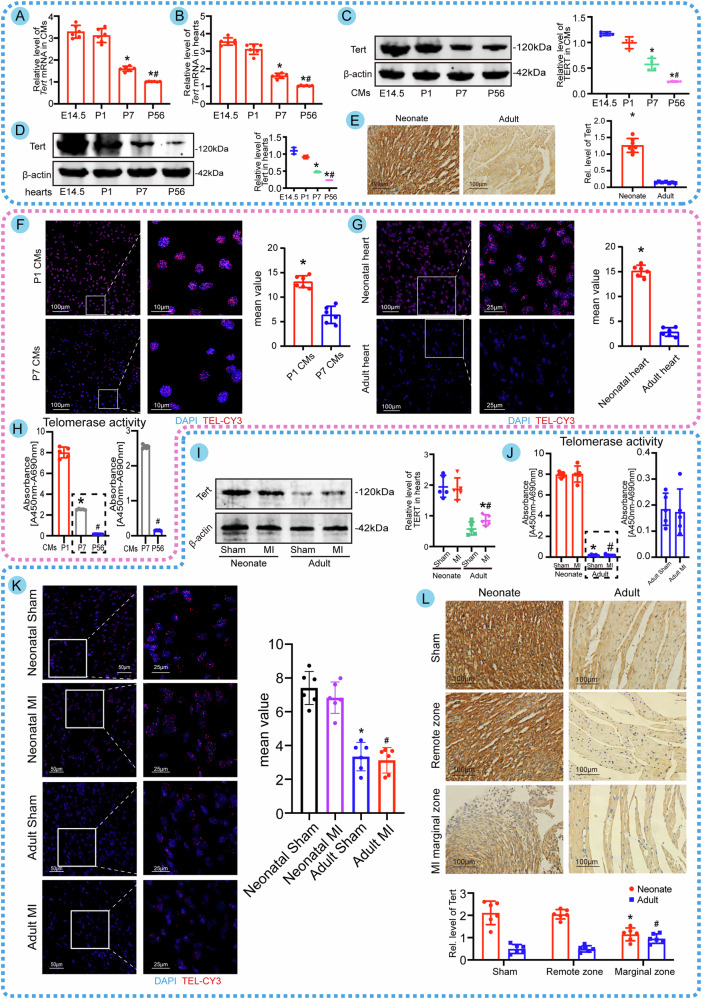
Tert, telomere, and telomerase activity characterize cardiac development and cardiac repair after myocardial infarction. **A** qPCR analysis of Tert expression in CMs from fetal mice and different day-old mice (*P < 0.05 vs. P1 group; ^#^*P* < 0.05 vs. P7 group; *n* = 6 cell samples per condition). **B** qPCR analysis of Tert expression in cardiac tissues from fetal mice and different day-old mice (*P < 0.05 vs. P1 group; ^#^*P* < 0.05 vs. P7 group; *n* = 6 heart samples per group). **C** Western blot assay of Tert expression in CMs from fetal mice and different day-old mice (*P < 0.05 vs. P1 group; ^#^*P* < 0.05 vs. P7 group; *n* = 6 cell samples per condition, the experiment was replicated in the laboratory a total of three times). **D** Western blot assay of Tert expression in cardiac tissues from fetal mice and different day-old mice (*P < 0.05 vs. P1 group; ^#^*P* < 0.05 vs. P7 group; *n* = 6 heart samples per group, the experiment was replicated in the laboratory a total of three times). **E** Immunohistochemical images of Tert in the normal cardiac tissues of neonatal mice and adult ones (*P < 0.05 vs. adult group; *n* = 6 slices from 6 animals per group). **F** Comparison of telomere lengths of P1 CMs and P7 CMs by Q-FISH (*P < 0.05 vs. P7 CMs; 501 CMs from 12 images of 6 mice in P1 CMs group and 492 CMs from 12 images of 6 mice in P7 CMs group). **G** Comparison of telomere lengths in neonatal and adult hearts by Q-FISH (*P < 0.05 vs. Adult heart; *n* = 6 slices from 6 animals per group). **H** Telomerase activity of isolated CMs from P1, P7, and P56 mice was detected by the TeloTAGGG Telomerase PCR ELISA kits. The right panel is a magnified view of the dashed box (*P < 0.05 vs. P1 group; ^#^*P* < 0.05 vs. P7 group; *n* = 5 cell samples per condition). **I** Detection of Tert using Western blotting assay in neonatal and adult hearts with a sham or MI model, respectively (*P < 0.05 vs. Neonate MI group; ^#^*P* < 0.05 vs. Adult Sham group; *n* = 5 heart samples per group). **J** Telomerase activity of cardiac tissues from neonatal and adult mice with a sham and MI model. The right panel is an enlarged view of the dashed box (*P < 0.05 vs. Neonate Sham group; ^#^*P* < 0.05 vs. Neonate MI group; *n* = 5 heart samples per group). **K** Q-FISH comparison of telomere lengths of cardiac tissues in post-infarct hearts from neonatal and adult mice of both Sham and MI groups (*P < 0.05 vs. Neonatal Sham group; ^#^*P* < 0.05 vs. Neonatal MI group；*n* = 6 slices from 6 animals per group). **L** Immunohistochemical analysis of Tert in neonatal and adult heart of both Sham and MI groups (different regions) (*P < 0.05 vs. Neonate Remote zone group; ^#^*P* < 0.05 vs. Adult Remote zone group; *n* = 6 slices from 6 animals per group). DAPI represents the nucleus. TEL-CY3 represents the telomere length, and brighter fluorescence indicates increased telomere length. Error bars indicate SD of six biological repeats in **A**–**H**/**K**, **L** and that of five biological repeats in **H**–**J**. An unpaired *t* test in **E**–**G**, one-way ANOVA in **A**–**D**/**H** and two-way ANOVA in **I**–**L** were utilized to determine the statistical significance.

In prior research [17], high levels of telomerase activity have been linked to embryonic heart development. In the present study, we analyzed Tert, telomere length, and telomerase activity during cardiac development. We first examined how age affected the level of Tert and found that maximum levels of Tert were observed in embryonic hearts and embryonic CMs by real-time qPCR and western blotting, and it decreased with increasing age (Fig. 1A–D). Furthermore, the level of Tert was found to be substantially greater in neonatal hearts than in adult hearts, as evidenced by immunohistochemistry data (Fig. 1E). Subsequently, we employed quantitative FISH (Q-FISH) and qPCR assays to further examine telomere length. Additionally, we utilized telomerase PCR ELISA to evaluate telomerase activity in both the CMs and cardiac tissues of mice with similar ages. Consistent with the reported reduction in Tert, we discovered that telomere length and telomerase activity tended to decrease in P7 CMs and adult hearts compared to P1 CMs and neonatal hearts, respectively (Fig. 1F–H, Fig. S1A, B). Considering these observations, it is possible to conclude that Tert, telomeres, and telomerase contribute to CM proliferation and cardiac development. We also explored whether low Tert levels and telomerase inactivation are responsible for the poor degree of cardiac regeneration following adult MI. Compared to nearly completely healed hearts after neonatal MI, adult infarcted hearts lacked Tert expression, possessed little telomerase activity, and decreased telomere length (Fig. 1I–K, Fig. S1C). Since the renewal CMs are derived from the division of pre-existing CMs in the marginal infarct zone [18], we focus on Tert level in this area. Interestingly, although adult hearts exhibited significantly lower amounts of Tert than neonatal ones, we did find that Tert levels in the marginal zone of adult MI hearts were slightly more remarkable than in the remote zone (Fig. 1L). These results suggest that high Tert-level telomere preservation and telomerase activity may be linked to normal cardiac development and the capacity to heal myocardial damage.

### Tert overexpression diminishes telomeric DNA damage by lengthening telomeres, which promotes CM proliferation in vitro

**Fig. 2:**
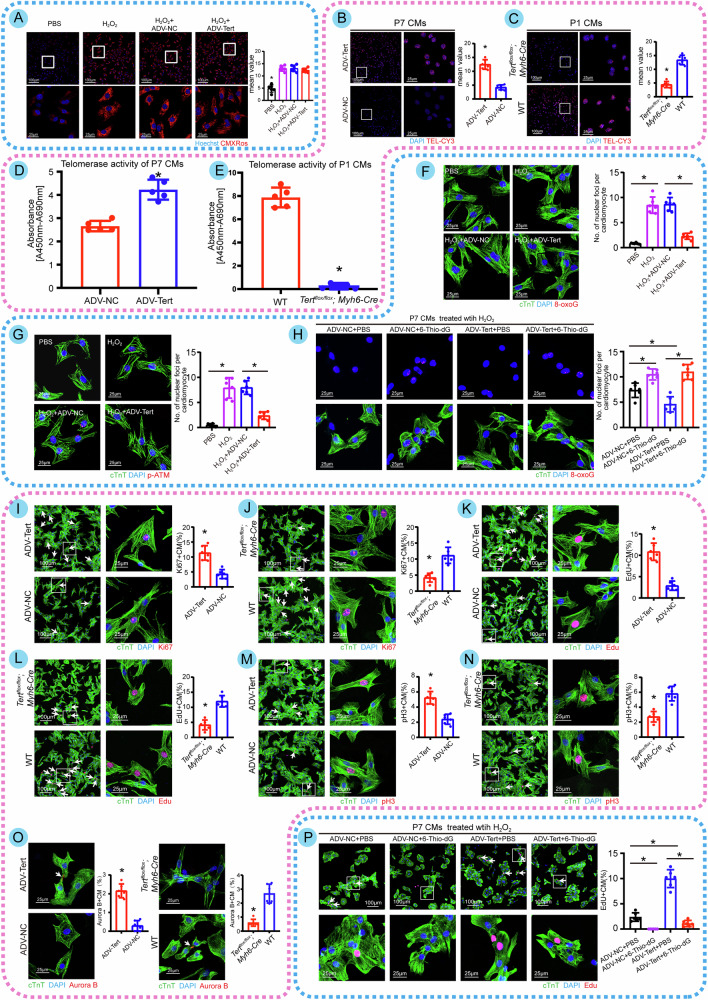
Tert overexpression diminishes telomeric DNA damage by lengthening telomeres, which promotes CM proliferation in vitro. **A** Immunofluorescence images of ROS in P7 CMs after different treatments and quantitative analysis (*P < 0.05 vs. other three groups; 596 CMs from 12 images of 6 mice in PBS group; 553 CMs from 12 images of 6 mice in H_2_O_2_ group; 589 CMs from 12 images of 6 mice in H_2_O_2_ + ADV-NC group and 521 CMs from 12 images of 6 mice in H_2_O_2_ + ADV-Tert group). Hoechst represents the nuclei and CMXRos represents ROS in CMs. H_2_O_2_ treatment increased ROS levels, but overexpression of Tert did not reduce this change. **B**, **C** Immunofluorescence images of telomere length in P7 CMs with Tert overexpression and P1 CMs isolated from WT or *Tert*^*flox/flox*^ mice and quantitative analysis (*P < 0.05 vs. ADV-NC group or WT group; 612 CMs from 12 images of 6 mice in ADV-Tert group; 609 CMs from 12 images of 6 mice in ADV-NC group; 573 CMs from 12 images of 6 mice in *Tert*^*flox/flox*^ *Myh6-Cre* group; 582 CMs from 12 images of 6 mice in WT group). **D**, **E** Telomerase activity of P7 CMs with Tert overexpression and P1 CMs isolated from WT or *Tert*^*flox/flox*^ mice, using TeloTAGGG Telomerase PCR ELISA kits (*P < 0.05; *n* = 5 cell samples per group). **F** Immunofluorescence images of 8-oxoG in P7 CMs after various treatments and quantitative analysis (*P < 0.05; 222 CMs from 18 images of 6 mice in PBS group; 271 CMs from 18 images of 6 mice in H_2_O_2_ group; 202 CMs from 18 images of 6 mice in H_2_O_2_ + ADV-NC group; 300 CMs from 18 images of 6 mice in H_2_O_2_ + ADV-Tert group). cTnT represents the myocardium, DAPI represents the nucleus and 8-oxoG represents telomeric DNA damage. **G** Immunofluorescence images of p-ATM in P7 CMs after various treatments and quantitative analysis (*P < 0.05; 203 CMs from 18 images of 6 mice in PBS group; 202 CMs from 18 images of 6 mice in H_2_O_2_ group; 240 CMs from 18 images of 6 mice in H_2_O_2_ + ADV-NC group; 212 CMs from 18 images of 6 mice in H_2_O_2_ + ADV-Tert group). cTnT represents the myocardium, DAPI represents the nucleus and p-ATM represents DDR protein. **H** Immunofluorescence images of 8-oxoG in H_2_O_2_-treated P7 CMs with different treatments and quantitative analysis (*P < 0.05; 291 CMs from 18 images of 6 mice in ADV-NC + PBS group; 248 CMs from 18 images of 6 mice in ADV-NC + 6-Thio-dG group; 329 CMs from 18 images of 6 mice in ADV-Tert+PBS group; 303 CMs from 18 images of 6 mice in ADV-Tert+6-Thio-dG group). 6-Thio-dG is an inhibitor of telomere elongation, which has no effect on Tert. **I**, **J** Immunofluorescence images of Ki67 in P7 CMs with Tert overexpression and P1 CMs isolated from WT or *Tert*^*flox/flox*^ mice. The right panels show statistical plots of the relevant quantitative analyses (Ki67+ CMs are indicated by arrows, *P < 0.05; 527 CMs from 18 images of 6 mice in ADV-Tert group; 603 CMs from 18 images of 6 mice in ADV-NC group; 512 CMs from 18 images of 6 mice in *Tert*^*flox/flox*^ *Myh6-Cre* group; 521 CMs from 18 images of 6 mice in WT group). **K**, **L** Immunofluorescence images of Edu in P7 CMs with Tert overexpression and P1 CMs isolated from WT or *Tert*^*flox/flox*^ mice. The right panels show statistical plots of the relevant quantitative analyses (Edu+ CMs are indicated by arrows, *P < 0.05; 514 CMs from 18 images of 6 mice in ADV-Tert group; 513 CMs from 18 images of 6 mice in ADV-NC group; 621 CMs from 18 images of 6 mice in *Tert*^*flox/flox*^ *Myh6-Cre* group; 495 CMs from 18 images of 6 mice in WT group). **M**, **N** Immunofluorescence images of pH3 in P7 CMs with Tert overexpression and P1 CMs isolated from WT or *Tert*^*flox/flox*^ mice. The right panel shows statistical plots of the relevant quantitative analyses (pH3+ CMs are indicated by arrows, *P < 0.05; 532 CMs from 18 images of 6 mice in ADV-Tert group; 513 CMs from 18 images of 6 mice in ADV-NC group; 609 CMs from 18 images of 6 mice in *Tert*^*flox/flox*^ *Myh6-Cre* group; 513 CMs from 18 images of 6 mice in WT group). **O** Immunofluorescence images of Aurora B in P7 CMs with Tert overexpression and P1 CMs with Tert knockout. The right panel shows relevant statistical plots (Aurora B+ CMs are indicated by arrows, *P < 0.05; 162 CMs from 18 images of 6 mice in ADV-Tert group; 133 CMs from 18 images of 6 mice in ADV-NC group; 119 CMs from 18 images of 6 mice in *Tert*^*flox/flox*^ *Myh6-Cre* group; 115 CMs from 18 images of 6 mice in WT group). **P** Immunofluorescence images of Edu in H_2_O_2_-treated P7 CMs with different treatments and quantitative analysis (Edu+ CMs are indicated by arrows, *P < 0.05; 321 CMs from 18 images of 6 mice in ADV-NC + PBS group; 298 CMs from 18 images of 6 mice in ADV-NC + 6-Thio-dG group; 309 CMs from 18 images of six mice in ADV-Tert+PBS group; 287 CMs from 18 images of 6 mice in ADV-Tert+6-Thio-dG group). Error bars indicate SD of six biological repeats in **A**–**C**/**F**–**P** and that of five biological repeats in **D**, **E**. An unpaired *t* test in **B**–**E**/**I**–**O**, one-way ANOVA in **A**/**F**–**H**/**P** were utilized to determine the statistical significance.

We needed to guarantee successful viral transfection and efficient viral intervention for Tert before applying ADV to up-regulate Tert in vitro. Colocalization of cTnT by EGFP fluorescence demonstrated that the transfection effectiveness of both empty and loaded vectors remained ~90% (Fig. S2A) without altering the size of CMs (Fig. S2B). Compared with the control group, western blotting findings for Tert overexpression in P7 CMs exhibited substantial upregulation, indicating that the virus transfected in vitro effectively overexpressed Tert (Fig. S2C). In addition, we utilized Tert floxed mice mated with Myh6-Cre mice to produce heart-specific Tert-deletion mice (*Tert*^*flox/flox*^*; Myh6-*Cre), which would be used for in vitro and in vivo experiments (Fig. S2D). Furthermore, the gel electrophoresis image validated the successful construction (Fig. S2E), with western blotting data demonstrating substantial knockout efficiency (Fig. S2F). Based on the results of ROS labeling (Fig. 2A), we observed that overexpression of Tert did not decrease the amount of ROS in P7 CMs following H_2_O_2_ treatment. Consequently, we analyzed Tert’s impact on telomeres and telomerase activity and concluded that Tert successfully extended telomere length and elevated telomerase activity (Fig. 2B–E, Fig. S2G, H). Furthermore, we discovered that Tert overexpression significantly reduced the levels of 8-oxoG, indicating telomeric DNA damage, and p-ATM, representing DDR (Fig. 2F, G). In addition, we used 6-Thio-dG, an agent that suppresses telomere lengthening without altering telomerase activity, to confirm that 6-Thio-dG was able to block Tert-induced downregulation of telomeric DNA damage, establishing that Tert needs telomere extension to play a role in decreasing telomeric DNA damage (Fig. 2H). These results demonstrated that Tert-mediated alleviation of telomeric DNA damage is telomere length-dependent.

We then investigated how Tert affected CM proliferative capacity. As we hypothesized, Ki67, EdU, pH3, and Aurora B, markers of proliferation and mitosis, were considerably higher in P7 CMs with Tert overexpression and much lower in P1 CMs isolated from *Tert*^*flox/flox*^*; Myh6-*Cre mice (Fig. 2I–O), similar trend was also noticed when we utilized microplate assays to investigate Edu uptake in CMs (Fig. S2I, J). Additionally, flow cytometry analysis showed that Tert overexpression raised the percentage of P7 CMs in the S and G2/M phases of the cell cycle while decreasing the accumulation of G0/G1 phases; in contrast, Tert-knockout demonstrated the reverse (Fig. S3A). In addition, Tert significantly inhibited the apoptosis induced by H_2_O_2_ (Fig. S3B). These data imply that Tert is essential for maintaining CM proliferative potential.

As we observed, H_2_O_2_ substantially decreased the percentage of cells positive for Edu and the intracellular uptake of Edu in CMs from P1 wild-type mice (Fig. S3C, D), indicating ROS worsened the proliferative capacity of CMs. We then performed experiments on P7 CMs with Tert overexpression using 6-Thio-dG. Under the circumstance of oxidative stress injury, Tert overexpression increased the proliferative capacity of P7 CMs, while 6-Thio-dG inhibited the impact of Tert on CM proliferation (Fig. 2P, Fig. S3E), demonstrating that the action of Tert to boost CM proliferation would have to be reliant on telomere stabilization and extension. MitoQ10 is an antioxidant that blocks H_2_O_2_-induced intracellular ROS reactions and prevents oxidative damage, so we employed it to investigate how Tert modifies the proliferative capability of CMs by influencing DNA damage. As the results showed, the declined number of CMs positive for Edu, caused by H_2_O_2_ treatment, was reversed by MitoQ10. Additionally, MitoQ10 partially mitigated the further reduction in the proportion of EdU-positive CMs and the reduced uptake of Edu resulting from Tert knockout in the presence of ROS-induced injury (Fig. S3F, G); meanwhile, MitoQ10 dramatically increased the proportion of pH3 in H_2_O_2_-treated P1 CMs (Fig. S3H). These findings suggest that Tert minimizes telomeric DNA damage by extending telomere length, which enhances CM proliferation in vitro.

### Tert overexpression improves adult cardiac regeneration in vivo by protecting CMs from telomeric DNA damage

**Fig. 3:**
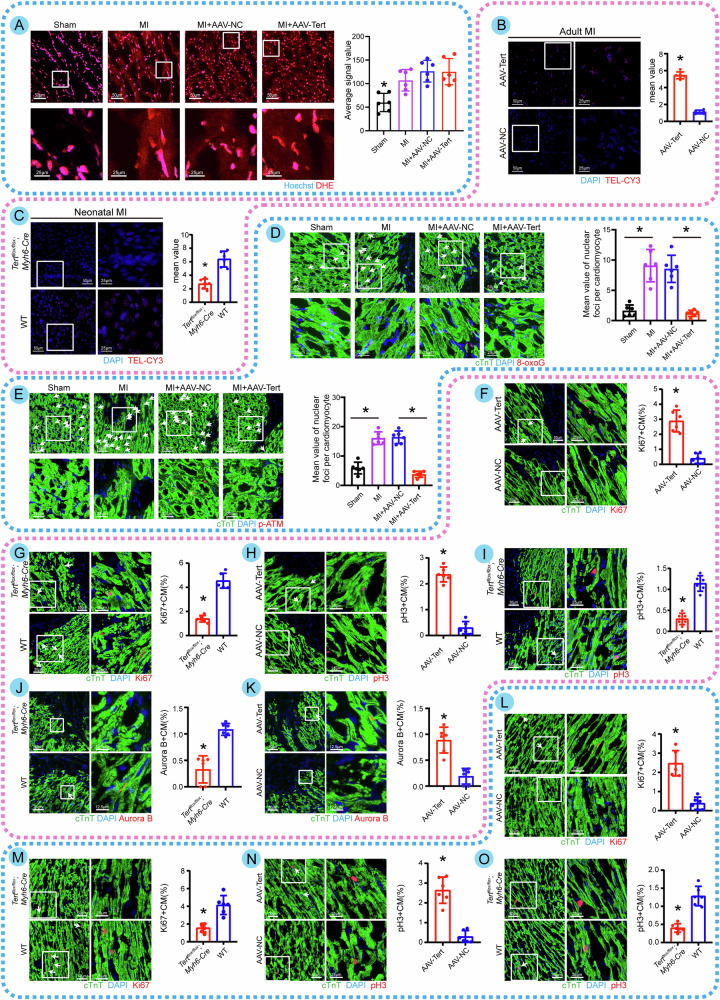
Tert overexpression improves adult cardiac regeneration in vivo by protecting CMs from telomeric DNA damage. **A** Immunofluorescence images and quantification of ROS in frozen sections of heart tissue from different groups of adult mice (*P < 0.05 vs.other three groups; *n* = 6 slices from 6 animals per group). Hoechst represents the nuclei, DHE labels ROS in myocardial tissue. Overexpression of Tert in vivo did not reduce ROS levels after MI, when compared to controls. **B**, **C** Immunofluorescence images and quantitative analysis of telomere length in heart tissues from adult mice with Tert overexpression and neonatal *Tert*^*flox/flox*^ mice (*P < 0.05; *n* = 6 slices from 6 animals per group). **D**, **E** Immunofluorescence images and quantitative analysis of 8-oxoG and p-ATM in each group of adult mice after various treatments (8-oxoG and p-ATM are indicated by arrows, *P < 0.05; *n* = 6 slices from 6 animals per group). **F**, **G** Immunofluorescence images of Ki67 in frozen heart sections from adult MI mice with Tert overexpression and neonatal *Tert*^*flox/flox*^ MI mice, respectively. The right panels show statistical plots of the relevant quantitative analyses (Ki67+ CMs are indicated by arrows, *P < 0.05; *n* = 6 slices from 6 animals per group). **H**, **I** Immunofluorescence images of pH3 in frozen heart sections from adult MI mice with Tert overexpression and neonatal *Tert*^*flox/flox*^ MI mice, respectively. The right panels show statistical plots of the relevant quantitative analyses (pH3+ CMs are indicated by arrows, *P < 0.05; *n* = 6 slices from 6 animals per group). **J**, **K** Immunofluorescence images of Aurora B in frozen heart sections from adult MI mice with Tert overexpression and neonatal *Tert*^*flox/flox*^ MI mice, respectively. (Aurora B CMs are indicated by arrows, *P < 0.05; *n* = 6 slices from 6 animals per group). **L**, **M** Immunofluorescence images of Ki67 in frozen heart sections from normal adult mice with Tert overexpression and normal neonatal *Tert*^*flox/flox*^ mice, respectively. The right panels show statistical plots of the relevant quantitative analyses (Ki67+ CMs are indicated by arrows, *P < 0.05; *n* = 6 slices from 6 animals per group). **N**, **O** Immunofluorescence images of pH3 in frozen heart sections from normal adult mice with Tert overexpression and normal neonatal *Tert*^*flox/flox*^ mice, respectively. The right panels show statistical plots of the relevant quantitative analyses (pH3+ CMs are indicated by arrows, *P < 0.05; *n* = 6 slices from 6 animals per group). Error bars indicate SD of six biological repeats. An unpaired *t* test in **B**, **C**/**F**–**O**, one-way ANOVA in **A**/**D**, **E** were utilized to determine the statistical significance.

We delivered AAV9 locally to normal adult mouse hearts. The immunohistochemistry findings indicated considerably higher Tert expression in the AAV-Tert group than in the control group, indicating stable and effective overexpression efficiency of the AAV vector in vivo (Fig. S4A, B). However, there was no significant increase in CM size in normal hearts following transfection with AAV-Tert (Fig. S4C). Consistent with the in vitro findings, Tert did not reduce the ROS levels in CMs observed in the infarct group (Fig. 3A). In order to determine the association between Tert and telomere length, telomeric DNA damage, and CM proliferation, a positive and negative argument was conducted in vivo. When Tert was overexpressed in adult MI mice and knocked out in neonatal MI ones, we discovered that Tert levels maintained a positive association with TEL-CY3 fluorescence signal intensity (Fig. 3B, C) and telomere copies (Fig. S4D, E), and a negative correlation with telomeric DNA damage and DDR mediators (Fig. 3D, E). Additionally, we performed Tert knockdown and overexpression in the hearts of 1-day-old neonatal mice and 18-month-old aged mice to study Tert’s impact on DNA damage and aging under natural conditions. Immunofluorescence indicates significant differences in the levels of DNA damage (8-oxoG) and aging (p21) markers between neonatal and aged mouse cardiac tissues (Fig. S5A, B), which revealed that overexpressed Tert was able to reduce DNA damage and inhibit aging. In addition, overexpression of Tert substantially enhanced the proportion of Ki67, pH3, and Aurora B-positive CMs in infarct hearts (Fig. 3F–K) and the proportion of Ki67, pH3-positive CMs in normal hearts (Fig. 3L–O), whereas downregulation of Tert had the reverse effect.

To establish that Tert overexpression reduces telomeric DNA damage through telomere length extension and hence enhances CM proliferation in vivo, we employed the telomere elongation inhibitor 6-Thio-dG in the heart-specific Tert-knockout mice. Tert overexpression, as predicted, could not restore the increased oxidative DNA damage and decreased fraction of pH3-positive CMs generated by 6-Thio-dG (Fig. S4F, G), suggesting that telomere length plays a crucial bridging function in Tert’s involvement in lowering telomeric DNA damage and increasing CM proliferation. In the rescue test on *Tert*^*flox/flox*^*; Myh6-*Cre mice, MitoQ10 was used as a supplement. Our experiments with P1 MI *Tert*^*flox/flox*^*; Myh6-*Cre mice found that MitoQ10 significantly increased the percentage of Ki67-positive CMs within the marginal infarct zone when used as an anti-DNA damage reagent (Fig. S4H), indicating MitoQ10 was capable of rescuing reduced CM proliferation caused by Tert knockout. These findings showed that Tert-mediated telomere extension might decrease telomeric DNA damage and improve CM proliferation in vivo.

### Tert overexpression improves cardiac function after MI in adult mice

**Fig. 4:**
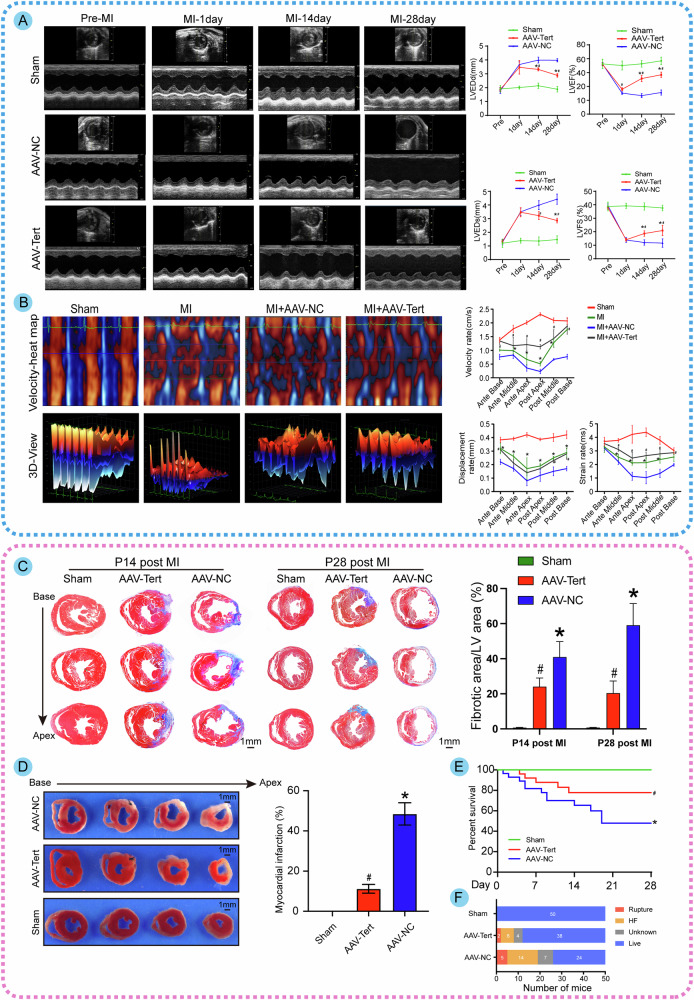
Tert overexpression improves cardiac function after MI in adult mice. **A** Echocardiography of left ventricular short axis and the analysis of the left ventricular end-diastolic diameter(LVEDd), left ventricular end-systolic diameter(LVEDs), left ventricular ejection fraction(LVEF) and left ventricular shortening fraction(LVFS) at different time points between different groups (*P < 0.05 vs. Sham group; ^#^*P* < 0.05 vs. AAV-NC group; *n* = 6 mice per group). **B** STI analysis of ventricular wall motion and staining traces in different groups (*P < 0.05 vs. Sham group; ^#^*P* < 0.05 vs. MI + AAV-NC group; *n* = 6 mice per group). **C** Masson staining of adult hearts following 14 and 28 days post-infarction (*P < 0.05 vs. Sham group; ^#^*P* < 0.05 vs. AAV-NC group; *n* = 6 mice per group). **D** TTC staining of adult hearts after 48 hours post-infarction (*P < 0.05 vs. Sham group; ^#^*P* < 0.05 vs. AAV-NC group; *n* = 6 mice per group). **E** Statistical plots of prognostic survival rates in different groups (*P < 0.05 vs. Sham group; ^#^*P* < 0.05 vs. AAV-NC group; *n* = 6 mice per group). **F** Comparative mortality analysis of mice post-MI in different treatment groups. White numbers on the colored bars represented the respective numbers of mice for each category of death. Error bars indicate SD of six biological repeats. A one-way ANOVA in **C**, two-way ANOVA in **A**, **B**/**D** and the log-rank (Mantel-Cox) test in **E** were utilized to determine the statistical significance.

In light of these in vivo findings, we sought to investigate whether Tert is involved in regulating heart repair in response to ischemic injury. According to two-dimensional thoracic echocardiography, the AAV-Tert group preserved significantly higher LVEF and LVFS compared to the control group (Fig. 4A). Using STI analysis, we found that Tert overexpression increased the velocity of myocardial motion and staining traces, as well as the ventricular wall displacement rate (Fig. 4B). Histologically, triphenyltetrazolium chloride (TTC) staining and Masson trichrome staining showed a significant reduction in infarct size and cardiac fibrosis in the AAV-Tert group compared to controls (Fig. 4C, D). Importantly, the AAV-Tert group demonstrated a significantly higher survival rate after MI compared to the control group (Fig. 4E). Further analysis of the causes of death revealed that, compared to the control group, the AAV-Tert group experienced significantly fewer deaths due to cardiac rupture and heart failure, which suggested that Tert may provide protective effects on the prognosis of MI (Fig. 4F). All the above results suggested that Tert overexpression contributes to the recovery of cardiac function after MI in vivo.

### HnRNPA2B1 regulates Tert and promotes CM proliferation

**Fig. 5:**
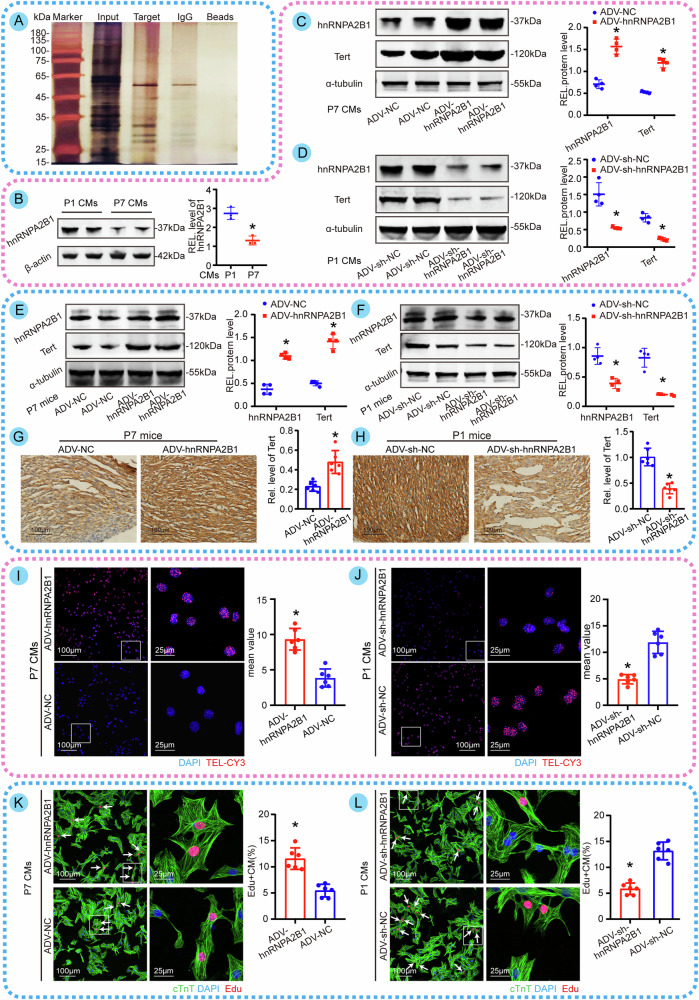
HnRNPA2B1 regulates Tert and promotes CM proliferation. **A** SDS-PAGE images of proteins captured by biotin-labeled Tert probes. There were significant variations in bands between the Target and IgG groups. **B** The basal expression of hnRNPA2B1 in P1 CMs and P7 CMs (*P < 0.05 vs. P1; *n* = 3 cell samples). **C**, **D** Western blotting assay and quantitative analysis of hnRNPA2B1 and Tert in P7 CMs with hnRNPA2B1 overexpression and P1 CMs with hnRNPA2B1 knockdown (*P < 0.05 vs. ADV-NC or ADV-sh-NC; *n* = 4 cell samples, the experiment was replicated in the laboratory a total of three times). **E**, **F** Western blotting assay and quantitative analysis of hnRNPA2B1 and Tert in hearts from P7 mice with hnRNPA2B1 overexpression and P1 mice with hnRNPA2B1 knockdown (*P < 0.05 vs. ADV-NC or ADV-sh-NC; *n* = 4 cell samples, the experiment was replicated in the laboratory a total of three times). **G**, **H** Immunohistochemical assay and quantitative analysis of Tert in cardiac tissues from P7 mice with hnRNPA2B1 overexpression and P1 mice with hnRNPA2B1 knockdown (*P < 0.05 vs. ADV-NC or ADV-sh-NC; *n* = 6 slices from 6 mice per group). **I**, **J** Immunofluorescence images and quantitative analysis of telomere length in P7 CMs with hnRNPA2B1 overexpression and P1 CMs with hnRNPA2B1 knockdown (*P < 0.05 vs. ADV-NC or ADV-sh-NC; 544 CMs from 18 images of 6 mice in ADV-hnRNPA2B1 group; 579 CMs from 18 images of 6 mice in ADV-NC group; 532 CMs from 18 images of 6 mice in ADV-sh-hnRNPA2B1 group; 497 CMs from 18 images of 6 mice in ADV-sh-NC group). **K**, **L** Immunofluorescence images and quantitative analysis of Edu in P7 CMs with hnRNPA2B1 overexpression and P1 CMs with hnRNPA2B1 knockdown (Edu+ CMs are indicated by arrows, *P < 0.05 vs. ADV-NC or ADV-sh-NC; 498 CMs from 18 images of 6 mice in ADV-hnRNPA2B1 group; 491 CMs from 18 images of 6 mice in ADV-NC group; 501 CMs from 18 images of 6 mice in ADV-sh-hnRNPA2B1 group; 439 CMs from 18 images of 6 mice in ADV-sh-NC group). Error bars indicate SD of three biological repeats in **B**, that of four biological repeats in **C**–**F** and that of six biological repeats in **G**–**L**. An unpaired *t* test in **B**/**I**–**L**, two-way ANOVA in **C**–**F** were utilized to determine the statistical significance.

**Fig. 6:**
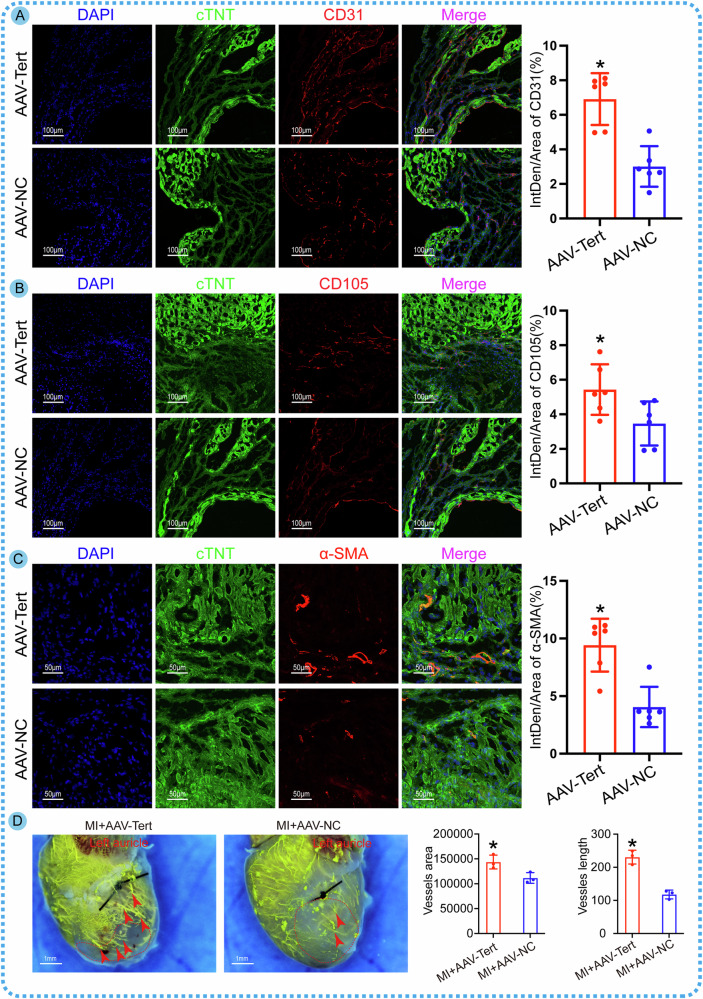
Overexpression of Tert specifically in CMs promotes angiogenesis in vivo following MI. **A** Immunofluorescent labeling of CD31 in adult mice with MI to assess the effects of myocardial cell-specific Tert overexpression on angiogenesis, and quantitative statistical analysis of CD31 (*P < 0.05 vs. AAV-NC; *n* = 6 slices from 6 mice per group). **B** Immunofluorescent labeling of CD105 in adult mice with MI to evaluate the effects of myocardial cell-specific Tert overexpression on angiogenesis, and quantitative statistical analysis of CD105 (*P < 0.05 vs. AAV-NC; *n* = 6 slices from 6 mice per group). **C** Immunofluorescent labeling of α-SMA in adult mice with MI to evaluate the effects of myocardial cell-specific Tert overexpression on angiogenesis, and quantitative statistical analysis of α-SMA (*P < 0.05 vs. AAV-NC; *n* = 6 slices from 6 mice per group). **D** Microfil injection casting of coronary artery demonstrates the formation of collateral blood vessels following MI (*P < 0.05 vs. AAV-NC; *n* = 3 mice per group). The red arrows indicate the collateral blood vessels. Error bars indicate SD of six biological repeats in **A**–**C** and that of three biological repeats in **D**. An unpaired *t* test in **A**–**D** were utilized to determine the statistical significance.

To determine the mechanisms involved in the upstream regulation of Tert, we performed DNA pulldown assays using biotinylated probes that targeted transcriptional regulation of Tert (Fig. 5A) and analyzed the precipitates using mass spectrometry. RNA-binding proteins were identified through mass spectrometry analysis (Supplementary Tables 1–3). We checked the candidates one by one in these three listed results and reviewed previous research [19, 20], finding that hnRNPA2B1, which increases telomerase activity and telomere length in cancer cells, appeared in ranks of the list of these proteins. Hence, we speculate that hnRNPA2B1 is the most likely to regulate Tert expression in CMs. To corroborate this hypothesis, we first detected the basal level of hnRNPA2B1. We found that it was significantly higher in P1 CMs than in P7 CMs (Fig. 5B). Then we performed a positive and negative validation by overexpressing hnRNPA2B1 in P7 CMs and downregulating hnRNPA2B1 in P1 CMs, which showed that overexpression but not knockdown of hnRNPA2B1 could promote upregulation of Tert level (Fig. 5C, D). Performing the same manipulation in neonatal hearts, we also observed similar results (Fig. 6E–H). Also, the results (Fig. 5C–F) established the validity of Tert overexpression and knockdown. Next, we investigated the role of hnRNPA2B1 on telomere length and CM proliferation. It was observed that the overexpression of hnRNPA2B1 led to an augmentation in the fluorescence intensity of TEL-CY3 and the number of telomere copies in CMs (Fig. 5I, J, Fig. S6A, B), along with an elevation in the proportion of EdU-positive CMs and the uptake of Edu in CMs (Fig. 5K, L, Fig. S6C, D). Overall, our data suggest that hnRNPA2B2 acts as an upstream regulator of Tert to promote telomere lengthening and CM proliferation.

### Tert facilitates angiogenesis by activating the NF-κB pathway in CMs and directly binding to the VEGF promoter in endothelial cells

**Fig. 7:**
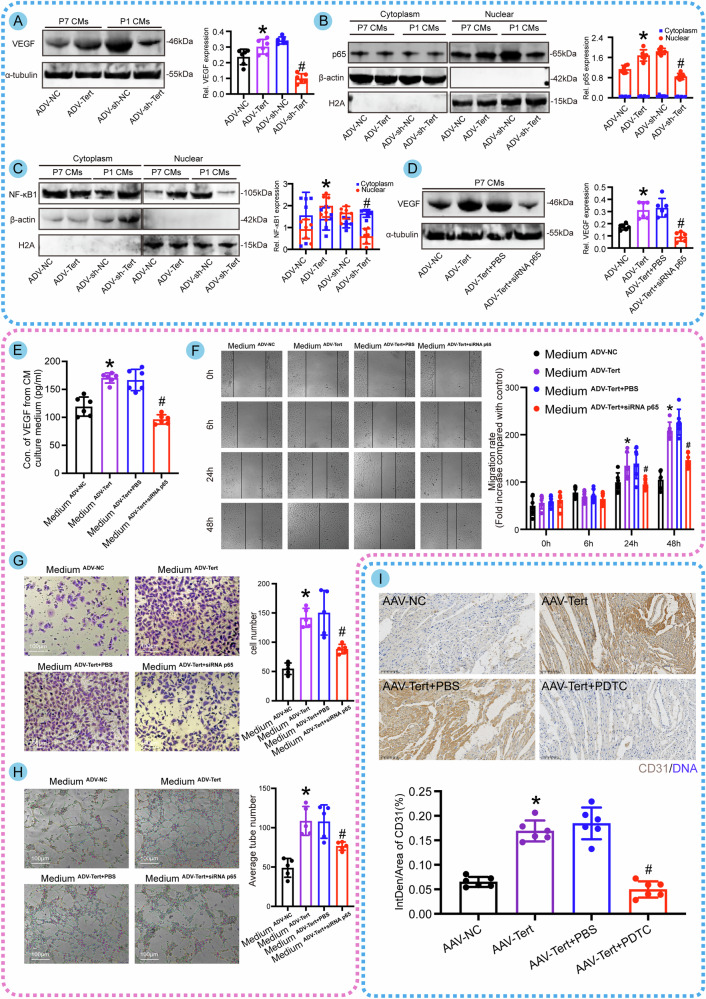
The activation of the NF-κB pathway by Tert in CMs boosts VEGF secretion, ultimately resulting in heightened angiogenesis. **A** Western blot analysis of VEGF expression in CMs with Tert overexpression or knockdown (*P < 0.05 vs. ADV-NC; ^#^*P* < 0.05 vs.ADV-sh-NC; *n* = 6 cell samples per group, the experiment was replicated in the laboratory a total of three times). **B** Western blot analysis of cytoplasmic and nuclear p65 expression in CMs with Tert overexpression or knockdown. Tert enhanced the p65 expression in nuclear, while the p65 within the cytoplasm was maintained at a constant and relatively low level (*P < 0.05 vs. ADV-NC in the Nuclear group; ^#^*P* < 0.05 vs.ADV-sh-NC in the Nuclear group; *n* = 6 cell samples per group, the experiment was replicated in the laboratory a total of three times). **C** Western blot analysis of NF-κB1 protein expression in CMs with Tert overexpression or knockdown. Tert facilitated the translocation of NF-κB1 protein from the cytoplasm to the nucleus, leading to the activation of the NF-κB pathway (*P < 0.05 vs. ADV-NC in Nuclear group; ^#^*P* < 0.05 vs.ADV-sh-NC in Nuclear group; *n* = 6 cell samples per group, the experiment was replicated in the laboratory a total of three times). **D** Western blot analysis of VEGF expression in CMs to assess whether Tert overexpression can rescue the inhibitory effect of siRNA p65 on the NF-κB pathway (*P < 0.05 vs. ADV-NC; ^#^*P* < 0.05 vs. ADV-Tert+PBS; *n* = 6 cell samples per group). **E** ELISA detection of VEGF concentrations in the culture medium of CMs under different treatments (*P < 0.05 vs. ADV-NC; ^#^*P* < 0.05 vs. ADV-Tert+PBS; *n* = 6 cell samples per group). **F** Scratch assay of HUVEC cells cultured in the CM culture medium with different treatments (*P < 0.05 vs. Medium^ADV-NC^; ^#^*P* < 0.05 vs. Medium^ADV-Tert+PBS^; *n* = 6 cell samples per group). **G** Transwell assay of HUVEC cells cultured in the CM culture medium with different treatments (*P < 0.05 vs. Medium^ADV-NC^; ^#^*P* < 0.05 vs. Medium^ADV-Tert+PBS^; *n* = 5 cell samples per group). **H** Tube-formation assay of HUVEC cells cultured in the CM culture medium with different treatments (*P < 0.05 vs. Medium^ADV-NC^; ^#^*P* < 0.05 vs. Medium^ADV-Tert+PBS^; *n* = 5 cell samples per group). **I** Immunohistochemical detection of CD31 expression in the infarcted heart to evaluate whether Tert overexpression can rescue the inhibitory effect of PDTC, an NF-κB pathway inhibitor, on angiogenesis in vivo (*P < 0.05 vs. ADV-NC; ^#^*P* < 0.05 vs. ADV-Tert+PBS; *n* = 6 slices from 6 mice per group). Error bars indicate SD of six biological repeats in **A**–**F**/**I** and that of five biological repeats in **G**, **H**. A one-way ANOVA in **A**/**D**, **E**/**G**–**I** and two-way ANOVA in **B**–**C**/**F** were utilized to determine the statistical significance.

A previous study has identified that Tert promotes tumor angiogenesis by directly interacting with the VEGF gene [21], leading to the upregulation of VEGF expression. However, whether Tert enhances cardiac angiogenesis remains unclear. Our in vivo research demonstrated that myocardium-specific overexpression of Tert can promote angiogenesis in infarcted hearts as evidenced by increased expression of CD31-, CD105-, and α-SMA-positive signals in the infarct border zone (Fig. 6A–C), Additionally, enhanced collateral angiogenesis in the peri-infarct region was observed by microfil injection (Fig. 6D) following local injection of AAV-Tert overexpression vector containing myocardium cell-specific promoters. These observations suggested that myocardial intervention can enhance angiogenesis, leading us to propose the hypothesis that myocardial cells promote angiogenesis through paracrine patterns onto endothelial cells. Previous studies have shown that Tert overexpression can activate the NF-κB signaling pathway, leading to the migration of p65 and NF-κB1 into the nucleus, thereby promoting angiogenesis [22]. Other studies have shown that CMs are a significant source of VEGF secretion in the heart and promote coronary artery angiogenesis by increasing VEGF secretion [23, 24]. Therefore, we speculated that myocardial Tert overexpression promotes VEGF synthesis and secretion through activation of the NF-κB signaling pathway, thereby promoting endothelial cell proliferation and angiogenesis. To investigate this, we performed overexpression and knockdown of Tert in cultured CMs. Western blotting results showed upregulation of VEGF in CMs with Tert overexpression (Fig. 7A), while levels of p65 and NF-κB1 proteins in the nuclear protein extracted from CMs showed a significant downward trend with Tert knockdown (Fig. 7B, C). To verify that the upregulation of VEGF by Tert is dependent on the NF-κB signaling pathway, we inhibited the activation of p65 while overexpressing Tert and found that the induction of VEGF expression by Tert can be counteracted by the inhibitory effect of p65 (Fig. 7D). These results indicated that Tert induces VEGF expression in CMs through the NF-κB pathway. To further confirm the communication between CMs and endothelial cells, we used ELISA detection at first to confirm the positive effect of Tert overexpression on VEGF secretion in the cultured CM medium (Fig. 7E). Subsequently, we co-cultured HUVEC cells using the CM conditioned medium with different treatments and performed scratch assays, transwell assays, and tube-formation assays, which indicated that Tert overexpression in CMs enhances endothelial cell migration, invasion, and sprouting, thereby promoting angiogenesis. However, inhibition of NF-κB activation weakened this trend (Fig. 7F–H). Immunohistochemical results of infarct hearts showed that specific overexpression of Tert in CMs significantly increased CD31, representing vascular endothelial cells. However, the NF-κB pathway inhibitor PDTC significantly weakened the promotional effect of Tert on angiogenesis (Fig. 7I). Taking all the results into consideration, we conclude that Tert promotes endothelial cell function by activating the NF-κB pathway in CMs and inducing VEGF expression and secretion. Additionally, we examined the independent effect of Tert in endothelial cells to explore its direct influence on VEGF. We found that Tert overexpression in endothelial cells, but not knockdown, enhanced the migration and invasive capacity of HUVEC cells and promoted tube formation and endothelial sprouting (Fig. S7A–D). Considering the role of Tert in transcription, we designed an anti-Tert chromatin immunoprecipitation (ChIP)-qPCR assay. According to the results, the anti-Tert antibody enriched the VEGF promoter gene, suggesting a direct interaction between Tert and the VEGF promoter (Fig. S7E, F). Further experiments using cloned VEGF promoter regions with or without mutations in binding sites in the pGL3-basic reporter gene vector and co-transfection of ADV-Tert with the reporter gene vector into HUVEC cells showed a significant decrease in luciferase expression when putative VEGF binding sites were altered (Fig. S7G). This provides clear evidence that Tert does indeed bind to the VEGF promoter, which may establish another mechanism for the Tert-VEGF interaction. Immunoblotting experiments on samples also showed that Tert overexpression significantly affected VEGF expression (Fig. S7H, I). Based on these findings, we can conclude that Tert is a direct positive transcriptional regulator of VEGF expression and promotes angiogenesis in endothelial cells. In summary, Tert facilitates angiogenesis by activating the NF-κB pathway in CMs and directly binding to the VEGF promoter in endothelial cells.

### Tert-mediated repair of telomeric DNA damage blocks the ATM/Chk1/p53/p21 pathway to promote cell cycle re-entry

**Fig. 8:**
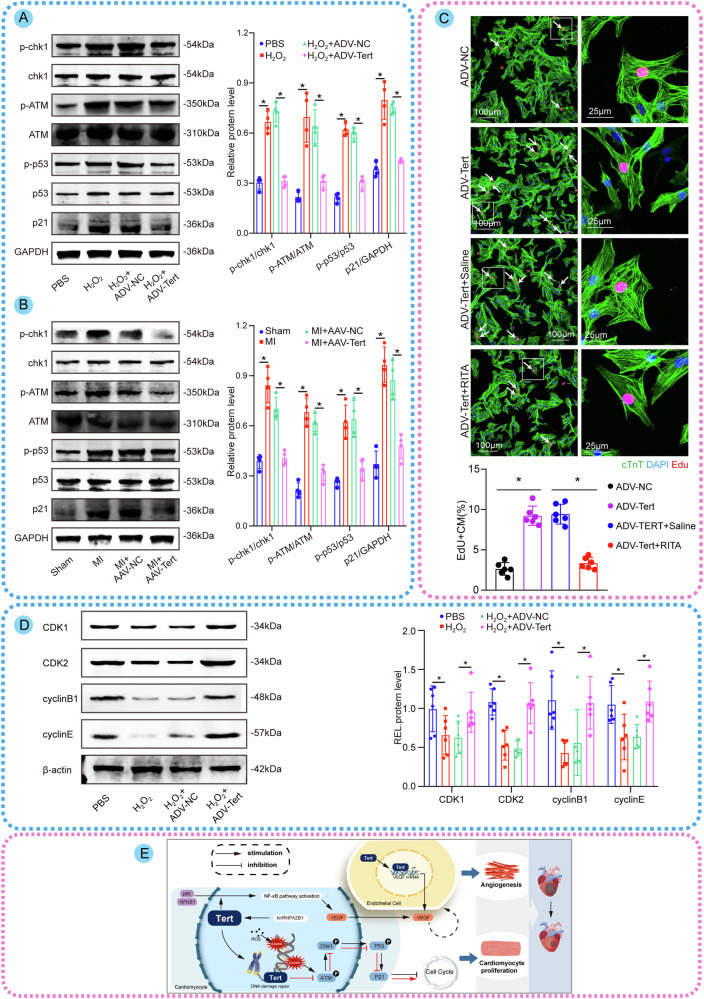
Tert-mediated repair of telomeric DNA damage blocks the ATM/Chk1/p53/p21 pathway to promote cell cycle re-entry. **A** Western blotting assay and quantitative analysis of DDR-associated proteins in P7 CMs of groups with different treatments (*P < 0.05; *n* = 4 cell samples per group, the experiment was replicated in the laboratory a total of three times). **B** Western blotting assay and quantitative analysis of DDR-associated proteins in cardiac tissues of groups with different treatments (*P < 0.05; *n* = 4 cell samples per group, the experiment was replicated in the laboratory a total of three times). **C** Immunofluorescence images and quantitative analysis of Edu in P7 CMs with different treatments (Edu+ CMs are indicated by arrows, *P < 0.05; 459 CMs from 18 images of 6 mice in ADV-NC group; 398 CMs from 18 images of 6 mice in ADV-Tert group; 503 CMs from 18 images of 6 mice in ADV-Tert+Saline group; 433 CMs from 18 images of 6 mice in ADV-Tert+RITA group). RITA is an activator that stimulates the phosphorylation of p53. **D** Western blotting assay and quantitative analysis of proteins involved in the cell cycle of P7 CMs with different treatments (*P < 0.05; *n* = 6 cell samples per group, the experiment was replicated in the laboratory a total of three times). **E** Schematic illustration of the main findings in this study: Tert, which is upregulated by hnRNPA2B1, could reduce telomeric DNA damage induced by ROS or MI via extending telomere length, thus allowing CM cycle re-entry through inhibiting DDR initiation to enhance CM proliferation. Besides, Tert can facilitate angiogenesis by activating the NF-κB pathway in CMs and directly binding to the VEGF promoter in endothelial cells to improve cardiac repair after MI. Error bars indicate SD of four biological repeats in **A**, **B** and that of six biological repeats in **C**, **D**. A one-way ANOVA in **C** and two-way ANOVA in **A**, **B**/**D** were utilized to determine the statistical significance.

Our other aim here is to explore in more detail how Tert-mediated telomeric DNA damage repair impacts the proliferation of cells through the possible mechanisms involved. Since DNA damage usually triggers the DDR pathway, we focused on detecting changes in the expression of the key proteins in this pathway. Compared with controls, H_2_O_2_-treated P7 CM showed higher levels of DDR proteins, including phosphorylated ATM(p-ATM), phosphorylated Chk1(p-Chk1), phosphorylated p53(p-p53), and p21. Interestingly, Tert overexpression was partially effective in reversing the changes in these phosphorylated proteins induced by H_2_O_2_ (Fig. 8A). Additionally, we investigated the effect of Tert overexpression on DDR pathway expression in vivo. As expected, Tert overexpression led to a marked reduction in the expression of p-ATM, p-Chk1, p-p53, and p21 in the MI model (Fig. 8B). So, Tert may inhibit the phosphorylation of the key proteins in the DDR pathway both in vitro and in vivo. We then investigated whether Tert affects CM proliferation by inhibiting the DDR pathway. RITA was used to activate p53 phosphorylation, leading to high expression of p21 and causing cell cycle arrest, which prompted us to perform a rescue assay here. Immunofluorescence experiments and Edu microplates assays showed that RITA remarkably prevented the proliferation of CMs induced by Tert overexpression (Fig. 8C, Fig. S8), suggesting that Tert’s promotion of CM proliferation is dependent on the downregulation of p-p53. Western blotting also confirmed that Tert overexpression reverses the H_2_O_2_-induced downregulation of cell cycle proteins CDK1, CDK2, cyclin B1, and cyclinE (Fig. 8D), demonstrating that Tert benefits the continuation of the cell cycle. In conclusion, these results suggest that Tert-mediated repair of telomeric DNA damage via extending telomere length could block the ATM/Chk1/p53/p21 pathway to promote cell cycle re-entry (Fig. 8E).

## Discussion

In this study, we identified an important role of Tert in inducing cell cycle re-entry by alleviating ROS-induced telomeric DNA damage in CMs and promoting angiogenesis after MI. We further elucidated that Tert could enhance the communication between CMs and endothelium to increase VEGF levels. Finally, we demonstrated that Tert-mediated telomeric DNA damage repair was achieved by elongating telomere length, further blocking the Chk1/p53/p21 pathway to alleviate DDR. Overall, our results revealed that Tert could be a novel therapeutic target for reducing ROS-induced DNA damage in CMs and facilitating angiogenesis after MI.

Our present study demonstrated that Tert can promote myocardial proliferation by inducing the cell cycle re-entry of CMs and inducing angiogenesis, thus improving cardiac function after MI. Previous studies have shown that targeting DNA damage-induced cell cycle re-entry in CMs could promote myocardial regeneration after MI [25, 26]. However, these studies did not make a more detailed classification of the damaged DNA, which made therapeutic targets for DNA damage repair less precise. Our current study anchored the telomere length controlled regenerative repair effect, the initial link of DNA damage, which is of great significance for the target exploration of HF after MI. The DNA damage caused by ROS is the main reason why CMs exit the cell cycle [27]. We found that overexpressed Tert boosted DNA synthesis and mitotic markers in CMs subjected to oxidative stress in vitro and in vivo, therefore achieving the improvement of cardiac function and prognosis post-MI. Enhanced Tert led to a significant reduction in the region of fibrotic scarring that was caused by MI. Furthermore, it increased cardiac function 28 days after MI, suggesting that Tert may be a potential target for myocardial regeneration and repair. Besides, we found that Tert overexpression protected CMs from apoptosis and promoted angiogenesis in the peri-infarct zone, both of which are necessary for myocardium regenerative repair after MI.

Although previous studies have also focused on the anti-apoptotic effects of Tert [28], our research delved deeper into the mechanisms of DNA damage repair and the promotion of CM cell cycle re-entry. We demonstrated that Tert overexpression alleviated ROS-induced telomeric DNA damage by enhancing telomerase activity and elongating telomeres, which in turn blocked the Chk1/p53/p21 pathway, reducing the DDR. This precise targeting of telomeric DNA damage repair represented a significant advancement over general DNA damage repair strategies as a therapeutic intervention for HF post-MI. Additionally, our study revealed that Tert not only protected CMs but also played an important role in promoting angiogenesis around the infarcted myocardium. We showed that overexpressed Tert increased VEGF levels, promoting angiogenesis and thereby improving blood supply to the damaged heart tissue. The dual role of Tert in promoting CM proliferation and enhancing angiogenesis underscored its potential as a multifaceted therapeutic target for myocardial repair. Using in vivo models, we also demonstrated that overexpressed Tert significantly reduced fibrotic scarring and markedly improved cardiac function 28 days post-MI. This finding was particularly important as it suggested that Tert could mitigate long-term adverse remodeling of the heart, which was a major challenge in the treatment of MI.

There has been an increasing awareness that ROS/oxidative stress is an important factor in DNA damage, therefore, making the reduction of DNA damage a prominent topic in the area of myocardial regeneration. In the present study, we further revealed that an important underlying reason why CM proliferation was inhibited is due to the inability of telomere wear and tear shortening to repair telomeric DNA damage induced by ROS/oxidative stress. However, Tert is able to alter this situation. Tert could achieve DNA damage repair and promote the re-entry of the CM cell cycle by increasing telomerase activity and lengthening telomeres. It had been demonstrated that activation of DDR was the culprit of CMs cell-cycle arrest [27]. Previous studies showed that interfering with enzymes involved in DNA repair pathways or supplementing with antioxidants may reduce oxidative DNA damage and offer cardiac benefits to combat the adverse repercussions of DNA damage [29, 30]. Recently, the critical role of telomere shortening in DNA damage has been explored, which showed that the capped elongation of telomeres helped to maintain telomere structural and functional homeostasis to avoid the recognition of aberrant telomere ends as DNA damage that triggers the DDR [31], indicating that telomere length intervention was of great value in alleviating DDR. Our present results found that Tert could enhance telomerase activity and lengthen telomeres, thus significantly improving DNA damage repair. When we mimicked oxidative stress by treating P1 CMs with hydrogen peroxide, overexpressed Tert decreased the telomeric DNA damage markers 8-oxoG and p-ATM in CM. Correspondingly, when we shortened the telomere length with 6-Thio-dG, enhanced Tert failed to reduce the high levels of 8-oxoG and p-ATM in CM treated with hydrogen peroxide, suggesting the ability of Tert to repair telomeric DNA damage depends on preservation of telomere length. These results indicated that Tert may serve as a reliable DNA protector in oxidative stress environments. Furthermore, we found that Tert could significantly increase the level of cell cycle regulators inhibited by ROS including cyclin B1, cyclinE, CDK1, and CDK2 in CMs, which indicated the potential of Tert to repair DNA damage and induce cell cycle re-entry. Taken together, our data indicated that ROS-induced telomere DNA damage might be an important mechanism for telomere shortening, further demonstrating that Tert could be a therapeutic target for DDR-induced CM cell cycle suppression by promoting telomerase activity and extending telomere length.

While direct regulation of cell cycle protein expression or activation of related pathways may promote cell cycle progression [27], these measures cannot alter the fact that telomere length shortens with a new round of DNA replication due to the end replication mechanism [32]. The presence of such pros and cons may prevent the cell proliferation potential from being fully stimulated. In this study, we focused on Tert, starting with telomere lengthening. We demonstrated that Tert repairs telomeric DNA damage by extending telomere length, which avoided the side effects of the telomere end replication mechanism. Our research further investigated the underlying mechanisms of how Tert-mediated repair of telomeric DNA damage regulated cell cycle re-entry. Phosphorylated ATM was considered to be the key factor involved in DDR, which could activate the Chk1/Chk2 kinases [33]. These kinases then phosphorylated p53 and increased the level of p21, all of which were responsible for the arrest in the cell cycle at different stages [34]. In this study, we found that Tert has the ability to suppress the phosphorylation of ATM and further inhibit Chk1/p53 pathway, then resulting in a decrease in p21 production. This suggests that Tert has the potential to limit the DDR that is triggered by telomeric DNA damage. We also explored the upstream mechanism by performing the DNA pulldown assay and found that the transcription factor hnRNPA2B1 was responsible for regulating Tert. It had been reported that hnRNPA2B1 could bind to the DNA sequences at the forum domain termini to maintain their function [35]. Our study indicated that hnRNPA2B1 could bind to the promoter of Tert and facilitate its expression, which was consistent with the DNA protective effect of Tert. Besides, interestingly, we found that Tert could activate the NF-κB signaling pathway in CMs to promote VEGF secretion and directly interact with the *VEGF* promoter in endothelium to increase VEGF level. The distinct mechanisms by which TERT regulates VEGF expression in different cell types are due to the physiological functions and response requirements of these cells, which work together to increase VEGF expression quickly and effectively. Collectively, our results indicated that Tert activated by hnRNPA2B1 could help myocardial repair following MI by inhibiting the ATM/Chk1/p53/p21 pathway to alleviate the ROS-induced DNA damage and increasing the level of VEGF to promote angiogenesis.

In this study, several limitations should be noted. Firstly, although hnRNPA2B1 has been identified as the upstream transcription factor of Tert to promote its transcription, future research should investigate the role of hnRNPA2B1 in increasing telomere length. Secondly, we illustrated the effect of Tert on the cardiac protection after MI, further studies should evaluate the weight of cardiomyocyte (CMs) regeneration and angiogenesis in the maintenance of cardiac function after MI. Thirdly, in this study, we applied AAV with cTnT promoter to perform in vivo overexpression of Tert in CMs. Further in vivo studies should design a vector for endothelial overexpression of Tert to prove that Tert is directly bound to the VEGF promoter in the endothelium to increase its level. Additionally, the inhibition of ATM phosphorylation by Tert may be one of the mechanisms to protect genomic DNA globally from ROS attack, and more comprehensive mechanisms warrant further exploration. Finally, it is difficult for current efforts to identify overlapping functions of Tert in nuclear and mitochondrial DNA, which will be our future investigation.

In conclusion, overexpressed Tert could promote myocardial regenerative repair post-MI by resuming the halted CM cell cycle and promoting angiogenesis. hnRNPA2B1-activated Tert blocked the ATM dependent chk1/p53/p21 pathway and enhanced the communication between CMs and endothelium. Thus, Tert involved in CM cell cycle re-entry and angiogenesis may be a promising target for HF after MI.

## Materials and methods

All mouse procedures were approved by the Nanfang Hospital Animal Ethics Committee of Southern Medical University and were conducted in line with the National Institutes of Health Guide for the Care and Use of Laboratory Animals [36]. Mice were deeply anesthetized with 5% isoflurane and then euthanized by cervical dislocation. Animals were confirmed euthanized if they were unresponsive to forceful toe pinching. All the wild-type mice of postnatal day 1 (P1 or neonatal mice), day 7 (P7), and day 56 (P56 or adult mice) used in this study were purchased from Guangdong Medical Laboratory Animal Center.

### Generation of heart-specific Tert-deletion mice and animal experiments

Tert-floxed heterozygous mice (C57BL/6J-Tert^em1cyagen^) and Myh6-Cre transgenic mice were purchased from Cyagen (Jiangsu, China). First, we generated Tert-floxed homozygous mice by mating Tert-floxed heterozygotes with each other. Next, we crossed Tert floxed homozygous mice with Myh6-Cre mice to create Myh6-Cre and loxP positive heterozygotes, which we then paired with Tert floxed homozygotes to generate heart-specific Tert-deletion homozygotes (*Tert*^*flox/flox*^*; Myh6-*Cre) for our research. Mouse MI was performed as described [37]. Briefly, 8-week-old adult male C57BL/6 mice were anesthetized with 1.5% isoflurane in 80% oxygen by using an anesthetic machine following oral intubation. A skin incision was made in the left 4th intercostal space, and then the left anterior descending coronary artery (LAD) was ligated with 8–0 silk suture approximately 2–3 mm away from the tip of the left auricle. Paleness of the left ventricular wall confirmed MI. About 4 weeks before LAD ligation, adeno-associated virus 9 harboring Tert for Tert overexpression (AAV-Tert) or harboring empty vector for control (AAV-NC) was administered into the ventricular wall at a concentration of 1 × 10^11^ viral genome particles per animal using an insulin syringe with a 30 G needle. After the LAD ligation and viral injection, the mice were warmed for a few minutes until recovery.

### Transfection of virus in vivo and in vitro

Vigene (Shandong, China) designed and produced the adenoviral vectors ADV-Tert and ADV-shTert, respectively, for Tert overexpression and Tert depletion. Transfection of cells with ADV-Tert, ADV-NC, ADV-sh-Tert, or ADV-sh-NC at a MOI of 10, as referenced from the reagent instructions and judiciously revised based on the insights from our prior study [38], was followed by RNA or protein extraction or immunofluorescence analysis 24 hours after transfection. The siRNA p65(Integrated DNA Technologies (IDT), USA; 5′-GGAGUACCCUGAGGCUAUAACUCGC-3′ (sense) and 5′-GCGAGUUAUAGCCUCAGGGUACUCCAU-3′ (antisense)), as referenced in a previous literature [39], was applied to the ADV-Tert+siRNA p65 group, in which CMs were transfected with siRNA p65 using lipo3000 24 hours before transfection with the Tert-carrying adenoviral vector. Neonatal and adult mice were infected with adenovirus (ADV) and adeno-associated virus 9 (AAV) vectors. P1 newborn mice were intracardially injected with ADV-sh-Tert or ADV-NC at a dosage of 3 × 10^8^ viral genome particles per animal using an insulin syringe with a 30-gauge needle (BD, NJ, USA), as referenced from the reagent instructions. Following injection, the hearts of mice were retrieved 7 days later. For long-term investigations (up to 28 or even 56 days), AAV-Tert or AAV-NC was administered intramyocardially at a dosage of 1 × 10^11^ viral genome particles per animal to 8-week-old mice.

### Perfusion fixation and coronary vessel casting

On the 28th day after MI, the animals were anesthetized again and the previously reported method [40] was used to perfuse the Microfil vascular casting agent (Flow Tech, Carver, MA) into the heart’s blood vessels. Subsequently, images were captured using a dissecting microscope. The objective analysis of angiogenesis data in our study employed previously reported analysis tools and methodologies from the literature [41].

### Cell isolation and culture

Neonatal CMs were isolated as previously described [42] from 1-day-old (P1) (derived from the heart-specific Tert-deletion neonatal mice) or 7-day-old (P7) C57BL/6 J mice (bought from the Experimental Animal Center of Southern Medical University). After anesthesia with 2% isoflurane inhalation, neonatal mice ventricles were separated, cut into pieces, and digested in 0.25% trypsin (Sigma) at 4 °C for 13 hours. After repeated digestion with type II collagenase (Roche) and bovine serum albumin (BSA, Sigma) in PBS at 37 °C for 15 minutes, the supernatant was collected after each step. The collected supernatant was centrifuged, and the cells were resuspended in 10% FBS-supplemented DMEM/F12 media (Invitrogen, Carlsbad, CA, USA). Cell suspensions were seeded onto 100 mm uncoated plastic dishes and incubated for 2 hours at 37 °C in a humidified atmosphere of 5% CO_2_. The CM-rich supernatant was collected and pelleted, and the cells were resuspended in DMEM/F12 medium, counted, and plated at the appropriate density. The purity of ventricular CMs generated with this method was consistently >90%. Additionally, these cells were tested for mycoplasma contamination, and the results were negative.

### Telomerase activity and telomere length assay

Samples of both the heart and the CMs were prepared before the procedure. After that, the telomerase activity was quantified using the TeloTAGGG Telomerase PCR ELISA kit (Roche) in accordance with the guidelines provided in the instruction manual. We utilized qPCR assays and the Q-FISH method to assess telomere length. The operation protocol for qPCR was followed according to the experimental manual supplied with the Relative Mouse Telomere-Length Quantification qPCR Assay Kit (ScienCell). For Q-FISH, frozen slides of hearts (3.5 mm) or cultured CMs were fixed in 4% polyformaldehyde for 4 minutes, washed twice in PBS at 37 °C for 2 minutes, and incubated in RNase A solution for 1 hour. The samples were placed in 0.005% pepsin solution at 37 °C for 5 minutes. Then, they were dehydrated in a cold ethanol series (for 1 minute in 70%, 85%, and 100%) after a second round of washing and fixing as described above [38]. After being air dried for 10 minutes at room temperature, the samples were heated in an incubator at 80 °C for 5 minutes, and then 20 µl of PNA probe (TEL-CY3) in Hybridization buffer was added to each sample. The samples were denatured for 10 minutes at 85 °C and then incubated for another hour at room temperature while in the dark. Samples were washed twice for 10 minutes at 55–60 °C in washing solution (2× SSC/0.1% Tween-20), followed by being stained with DAPI for 30 minutes (ab104139, Abcam). All samples were stained simultaneously, and all images were captured at the same laser intensity. For telomere-length analysis, DAPI and TEL-CY3 signals were acquired sequentially using confocal microscopy (Carl Zeiss, LSM880), and telomere signal intensity was quantified using Definiens software.

### Pharmacological administration

For the rescue experiment in vitro, CMs were treated with 1umol/L MitoQ10 (Selleck, NO. S8978) for 72 hours with daily medium replacement. In contrast, neonatal mice were administered intraperitoneally with MitoQ10 at 25 mg/kg daily for five days. The agent 6-Thio-dG (Selleck, NO.S7757) was dissolved in DMSO to produce a 100 mmol/L storage solution and kept at −80 °C according to the manufacturer’s guidelines. For in vitro investigations, the 6-Thio-dG storage solution was diluted with culture medium to obtain 1 mmol/L solution used to treat CMs for 72 hours. For in vivo experiments, mice were administered intraperitoneally with 10 mg/kg for 14 days. NF-κB specific inhibitor was used for the AAV-Tert+PDTC group, which was administered intraperitoneally with PDTC (Selleck, NO.S3633; 200 mg/kg) on the 28th day after AAV-Tert injection. RITA (Selleck, NO. S2781), also known as p53 activator III, was dissolved in DMSO at a concentration of 1 mmol/L as recommended by the manufacturer. After further diluting the above solution with medium to obtain a 10 nmol/L RITA stimulant, we applied it to cultured CMs and replaced it daily for in vitro experiments. The cells were harvested at 72 hours for further analysis.

### ROS detection and H_2_O_2_ treatment

The total cellular ROS detection and H_2_O_2_ treatment for CMs were performed as in our previous study [43]. For the detection of ROS in the cardiac tissue, we employed DHE (10 μM) reagent (Sigma-Aldrich) to incubate the frozen sections of cardiac tissue at 37 °C for 30 minutes, followed by imaging using a confocal microscope. The fluorescence intensity of DHE was quantified using ImageJ software.

### DNA pulldown assays

After being lysed in a cell lysis solution that included protease and phosphatase inhibitors, cultured cells were then treated at 4 °C for one hour with 40 μL of washed streptavidin-agarose beads. To continue, 2 μg of biotinylated double-stranded oligonucleotides and 40 μg of poly (dI-dC) (dI-dC) were added to the cells for a 16-hour incubation at 4 °C. The DNA-bound proteins were extracted using 60 μl of streptavidin–sepharose beads for one hour at 4 °C and washed twice in cell lysis buffer. The bound protein was identified using western blotting, and the specific bands were extracted and then analyzed by mass spectrometry. The results of the mass spectrometry are shown in Supplemental Tables 1–3.

### Luciferase reporter assay

The luciferase reporter experiments were performed as described previously [37]. Firstly, the luciferase vector pGL3-basic was cloned with VEGF-sv-wt and VEGF-sv-mut (Saiqing Biosciences, Guangzhou, China). Then, the ADV-Tert was co-transfected into HUVECs with the luciferase reporters using Lipofectamine 3000 (Invitrogen, Thermo Fisher Scientific). Finally, the Dual-Luciferase Reporter Assay System was used to measure the luciferase activity (Promega, Madison, WI).

### Chromatin immunoprecipitation assay

As previously described [42, 44], ChIP assays were carried out. Immunoprecipitation was performed using an anti-Tert antibody (1:100, sc-377511, SANTA CRUZ, CA, USA). qRT-PCR and PCR gel electrophoresis were then employed to detect the immunoprecipitated DNA. The primers of Vegf promoter and GAPDH promoter are listed as follows: Vegf-pro1: gtgttcttgcggttagagacagac (forward), gttagtaaatgtttgctggtccagag (reverse); Vegf-pro2: gatagcgtgaggagccagct (forward), cagtgtaatccactatctccatccc (reverse); Vegf-pro3: ggactggttggtccctctctt (forward), cccacctctaacccaccct (reverse); Vegf-pro4: agtacacccctgaattctgtttagaag (forward), cctaccctagcattcagaaaggtag (reverse); Vegf-pro5: gagagagagagatcaggaggaacaa (forward), caaatttgtggcactgagaacg (reverse); GAPDH-promoter: agtcctatcctgggaaccatcacc (forward), gcacgcaccaagcgtgtg (reverse).

### Immunofluorescence analysis

Immunofluorescence staining was performed as previously described [37, 45]. After being fixed in 4% polyformaldehyde for 30 minutes, frozen slices of cardiac tissues (3.5 mm) and cultured cells were permeabilized in 1% Triton X-100 PBS for 10 minutes and blocked with 1% bovine serum albumin (BSA) for 1 hour at room temperature. After that, the samples were incubated overnight at 4 °C with primary antibodies including anti-Ki67 antibody (1:100, ab16667, Abcam, Cambridge, UK), anti-p-Histone H3 (pH3) antibody (1:100, ab47297, Abcam, Cambridge, UK), anti-cTnT (1:50, ab8295, Abcam, Cambridge, UK), β-actin (1:100, 20536-1-AP, Proteintech, Wuhan, China), anti-aurora B (1:100, ab2254, Abcam, Cambridge, UK), anti-CD31 (1:100, ab222783, Abcam, Cambridge, UK), anti-CD105 (1:100, ab221675, Abcam, Cambridge, UK), anti-α-SMA (1:100, ab62623, Abcam, Cambridge, UK), anti-p-ATM (1:100, sc-47739, SANTA CRUZ Biotechnology, CA, USA), anti-8-oxoG (1:100, MAB3560, MilliporeSigma), anti-p21 (1:200, 28248-1-AP, Proteintech, Wuhan, China). Cells were stained with a Click-iT EdU Alexa Fluor 555 Imaging Kit (Invitrogen, CA, USA) according to the instruction manual provided by the manufacturer in order to detect EdU incorporation. Cells were processed with the use of an In Situ Cell Death Detection Kit (Roche, Shanghai, China) in order to perform TUNEL experiments. After being washed with PBS, the samples were incubated for 1 hour at room temperature with goat anti-mouse IgG/Alexa Fluor 488 (1:100, Biosynthesis, bs-0296G-A488, Beijing, China) or goat anti-rabbit IgG/Alexa Fluor 594 (1:100, Biosynthesis, bs-0295G-AF594, Beijing, China) secondary antibodies followed by 10 minutes of DAPI staining (1:500, ab104139, Abcam, Cambridge, UK). The confocal microscope (Carl Zeiss, LSM880) was utilized for the process of image acquisition. According to previous investigations, vessel density was defined as the percentage of the α-SMA-positive stained regions compared to the total tissue area in the corresponding slight field using Image-J software (Wayne Rasband).

### Edu uptake detected by microplate assays

To measure the EdU uptake in CMs, we conducted experiments following the guidelines provided by the supplier in the Click-iT^TM^ Edu Proliferation Assay for Microplates kits (Invitrogen).

### Western blot analysis

The procedure for western blotting was carried out as previously described [46] and replicated in the laboratory a total of three times. Lysates of whole cells or tissue extracts were prepared by incubating them in ice-cold RIPA lysis buffer (BestBio, Shanghai, China) with a protease inhibitor cocktail Set I. (BestBio, Shanghai, China). The BCA Protein Quantitative Analysis kit was used to quantify protein concentrations (Fudebio-tech, Hangzhou, China). Proteins were separated on 10% SDS-PAGE gels and then transferred to PVDF membranes (Millipore, USA). The following primary antibodies were employed: Tert (1:1000, sc-377511, SANTA CRUZ, CA, USA), hnRNPA2B1 (1:1000, sc-374053, SANTA CRUZ, CA, USA), p-CHK1 (1:1000, 28805-1-AP, Proteintech, Wuhan, China), CHK1 (1:1000, 60277-1-Ig, Proteintech, Wuhan, China), p-ATM (1:1000, sc-47739, SANTA CRUZ, CA, USA), ATM (1:1000, 27156-1-AP, Proteintech, Wuhan, China), p-p53 (1:1000, 60283-2-Ig, Proteintech, Wuhan, China), p53 (1:1000, 28961-1-AP, Proteintech, Wuhan, China), p21 (1:1000, 28248-1-AP, Proteintech, Wuhan, China), CDK1 (1:1000, 19532-1-AP, Proteintech, Wuhan, China), CDK2 (1:1000, 10122-1-AP, Proteintech, Wuhan, China), cyclin B1 (1:1000, 28603-1-AP, Proteintech, Wuhan, China), cyclinE (1:1000, 11554-1-AP, Proteintech, Wuhan, China), VEGF (1:1000, 19003-1-AP, Proteintech, Wuhan, China), p65 (1:1000, 10745-1-AP, Proteintech, Wuhan, China), NF-κB1 (1:5000, 14220-1-AP, Proteintech, Wuhan, China), H2A (1:1000, 10856-1-AP, Proteintech, Wuhan, China), β-actin (1:2000, 66009-1-Ig, Proteintech, Wuhan, China), GAPDH (1:5000, 60004-1-Ig, Proteintech, Wuhan, China), α-tubulin (1:1000, 66031-1-Ig, Proteintech, Wuhan, China). We used either an anti-rabbit IgG H&L antibody (1:10,000, ab16284, Abcam, Cambridge, UK) or an anti-mouse IgG H&L antibody (1:10,000, ab6728, Abcam, Cambridge, UK) or an anti-rabbit IgG H&L (Alexa Fluor680) antibody (1:10,000, ab175771, Abcam, Cambridge, UK) as the secondary antibody. A Chemiluminescence imaging GeneGnome XRQ (Syngene, MD, USA) was used to detect the signal. ImageJ software (NIH, Bethesda, MD, USA) was used to determine the relative density of proteins. The experiments were replicated in the laboratory a total of three times.

### Cardiac TTC staining and Masson trichrome staining

TTC staining was carried out in the same manner as previously reported [47]. Using metal slicers, frozen hearts were sectioned into 3 mm thick slices. These slices were stained with 1% TTC (Sigma-Aldrich) dissolved in PBS for 15 minutes at room temperature before being rinsed with PBS. The slices were then imaged, and the infarcted area was calculated using Image-Pro Plus 6.0. (Media Cybernetics, Bethesda, MD, USA). Masson trichrome staining was employed as directed by the manufacturer to measure the degree of heart fibrosis (blue for fibrotic areas; red for normal myocardium). The proportion of cardiac fibrosis in each animal was assessed using Image J analysis software (NIH, Bethesda, MD, USA) after reviewing five random images.

### Echocardiography

Transthoracic echocardiography was used to assess cardiac functions before MI, as well as at 1 day, 14 days, and 28 days following MI induction, utilizing a Vevo 2100 high-resolution imaging system (Visual Sonics, ON, Canada) coupled with a 40-MHz transducer. The left ventricular internal diameter at end-diastole (LVEDd) and end-systole (LVEDs) were measured using M-mode echocardiography. The left ventricular fractional shortening (LVFS) and ejection fraction (LVEF) were then computed using the previously reported measures. Tracking imaging (STI) was applied to comprehensively assess the abnormal motion of the ventricular wall after MI. The peak time of the left ventricle in the parasternal long-axis view was determined after acquiring B- and M-mode pictures of the ventricle. The strain capacity of the left ventricle wall was then evaluated for myocardial strain analysis using VevoStrain software version 1.4.0 (VisualSonics, Inc.), followed by obtaining the clearest endocardial and epicardial interface under the parasternal long-axis view. To exclude operator factors, all operations were performed by the same trained operator. The endocardium and epicardium tracking spots were set up in three consecutive cardiac cycles and manually adjusted to achieve optimal tracking. The images were then analyzed frame by frame to obtain the strain values and corresponding strain rate curves for each segment in the long-axis direction of the heart. Each set of measurements was averaged to assess the overall function.

### Flow cytometry

Flow cytometry was carried out exactly as previously reported [42]. Isolated CMs were transfected with ADV-NC or ADV-Tert at a dosage of 10^10^/ml for 24 hours before being collected. After fixation with cold 70% ethanol, samples were centrifuged at 1000 × *g* for 5 minutes before resuspending in FxCycle^TM^ PI/RNase Staining Solution (Thermo Fisher Scientific). MoFlo XDP (Cell Sorter) was used to examine suspensions.

### Scratch wound (migration) assay, transwell migration assay, tube-formation, and spheroid-sprouting assay

HUVECs motility (migration and invasion) was evaluated using scratch wound healing and transwell assays to determine Tert’s effect on angiogenesis [42]. In a humidified incubator at 37 °C and 5% CO_2_, HUVECs transfected with the virus were cultured to 90% cell fusion, followed by a scratch generated in the cell monolayer using a sterile pipette tip. Morphological characteristics were acquired using a Zeiss Axiovert 135 microscope following further incubation for 0, 12, 24, and 48 hours. The AxioVision (Zeiss) recording equipment was then used to quantify the intercellular distance. To evaluate the migration ability of HUVECs transfected with virus, we performed transwell experiments in which the upper chamber was cultured with a concentration of 1 × 10^5^ HUVECs, while the lower chamber was inoculated with ECM containing 10% FBS. After a further 48 h-incubation, cells were fixed in 4% PFA for 20 minutes and stained with crystal violet staining solution for 10 minutes to confirm cell movement. Zeiss AxioVision microscope (Jena, Germany) was used to photograph and count the cells under the microscope in five random fields.

HUVECs transfected with the virus were seeded onto 96-well plates (2 × 10^4^ cells/well) coated with 50 µl of matrix gel to visualize microvasculature and cultured at 37 °C in a humidified atmosphere containing 5% CO_2_. Cells were fixed in 4% PFA and imaged under a Zeiss AxioVision microscope (Jena, Germany) to quantify tube formation. Image Pro Plus software (Media Cybernetics, Bethesda, MD, USA) was used to analyze micrographs of tubules taken under a microscope. HUVEC spheroids were measured in terms of the greatest distance that migrating cells traveled or the total length of all shoots per spheroid. About 6 spheroids were used in each experiment.

### Volume analysis of CMs

As previously stated [47], isolated CMs were stained with anti-cTnT antibody (ab8295, Abcam, Cambridge, UK) and DAPI. To measure CM volume in cardiac tissue, primary antibody anti-WGA (T4144, Millipore Sigma) and anti-cTnT were used to mark the cell membrane and the myocardium, respectively. A Zeiss confocal LSM700 microscope was used to acquire Z-stack images of CMs, and the individual CM volume was quantified using the Imaris 8 (Bitplane) 3D image processing software application.

### Real-time polymerase chain reaction

The E.Z.N.A.® Total RNA Kit II (Omega 8 Biotek, Norcross, GA, USA) was used to extract total RNA from cell or tissue lysates [43]. Using a PrimeScript^TM^ RT Master Mix, reverse transcription was designed to produce cDNA (TaKaRa, Dalian, China). Quantitative PCR (qPCR) was carried out in a Light Cycler 480 II system using a SYBR Premix Ex Taq^TM^ Kit (Biotechnology, Dalian, China) (Roche, Basel, Switzerland). Glyceraldehyde-3-phosphate dehydrogenase (GAPDH) was used as an internal control to standardize amplified PCR results. The primer sequences of Tert are: GCACTTTGGTTGCCCAATG (forward), GCACGTTTCTCGTTGCG (reverse); The primer sequences of GAPDH are: TGACCTCAACTACATGGTCTACA (forward), CTTCCCATTCGGCCTTG (reverse).

### Statistical analysis

All statistical analyses were carried out using SPSS 20.0 software (IBM, New York, USA), and quantitative results are provided as mean ± SD. Unless otherwise specified, differences between two groups were assessed using Student’s *t* tests, and multiple comparisons were carried out using one-way ANOVA followed by Bonferroni’s multiple comparisons test or two-way ANOVA followed by Sidak’s test. The survival rate was determined using the Kaplan–Meier method, and the log-rank (Mantel–Cox) test was used to examine the variation in survival time across groups. A value of *P* < 0.05 was deemed statistically significant.

### Supplementary information

Proteins identified by mass spectrometry from sample 1

Proteins identified by mass spectrometry from sample 2

Proteins identified by mass spectrometry from sample 3

uncropped blots and supplementary figures and legends

## Data Availability

All data are contained within the manuscript.
