# Supplementary material for: Tert promotes cardiac regenerative repair after MI through alleviating ROS-induced DNA damage response in cardiomyocyte
Source: Cell Death Discov. 2024 Aug 26;10:381. doi: 10.1038/s41420-024-02135-8 (PMC11347641; doi:10.1038/s41420-024-02135-8)
Supplement: Supplementary file 1 — Proteins identified by mass spectrometry from sample 1 [file 41420_2024_2135_MOESM1_ESM.docx]

Proteins identified by mass spectrometry from sample 1

| Protein Group | Protein ID | Accession | -10lgP | Coverage (%) | Coverage (%) 1 | Area 1 | #Peptides | #Unique | #Spec 1 | PTM | Avg. Mass | Description |
| --- | --- | --- | --- | --- | --- | --- | --- | --- | --- | --- | --- | --- |
| 4 | 50 | sp\|P15864\|H12_MOUSE | 178.59 | 44 | 44 | 2.36E+07 | 13 | 4 | 39 | Acetylation (Protein N-term); Deamidation (NQ) | 21267 | Histone H1.2 OS=Mus musculus OX=10090 GN=H1-2 PE=1 SV=2 |
| 4 | 51 | Q5SZA3\|Q5SZA3_MOUSE | 178.59 | 44 | 44 | 2.36E+07 | 13 | 4 | 39 | Acetylation (Protein N-term); Deamidation (NQ) | 21267 | Histone cluster 1 H1c OS=Mus musculus OX=10090 GN=H1f2 PE=1 SV=1 |
| 9 | 11 | sp\|P56480\|ATPB_MOUSE | 177.7 | 25 | 25 | 1.13E+08 | 11 | 11 | 33 | Oxidation (M) | 56301 | ATP synthase subunit beta mitochondrial OS=Mus musculus OX=10090 GN=Atp5f1b PE=1 SV=2 |
| 5 | 5 | Q8C2Q8\|Q8C2Q8_MOUSE | 176.31 | 42 | 42 | 1.08E+08 | 13 | 13 | 36 | Carbamidomethylation; Deamidation (NQ); Pyro-glu from Q | 30256 | ATP synthase subunit gamma OS=Mus musculus OX=10090 GN=Atp5c1 PE=1 SV=1 |
| 5 | 6 | A2AKU9\|A2AKU9_MOUSE | 176.31 | 39 | 39 | 1.08E+08 | 13 | 13 | 36 | Carbamidomethylation; Deamidation (NQ); Pyro-glu from Q | 32771 | ATP synthase subunit gamma OS=Mus musculus OX=10090 GN=Atp5c1 PE=1 SV=1 |
| 5 | 10 | Q9ERA8\|Q9ERA8_MOUSE | 176.31 | 39 | 39 | 1.08E+08 | 13 | 13 | 36 | Carbamidomethylation; Deamidation (NQ); Pyro-glu from Q | 32852 | ATP synthase subunit gamma OS=Mus musculus OX=10090 GN=Atp5c1 PE=2 SV=1 |
| 5 | 8 | sp\|Q91VR2\|ATPG_MOUSE | 176.31 | 39 | 39 | 1.08E+08 | 13 | 13 | 36 | Carbamidomethylation; Deamidation (NQ); Pyro-glu from Q | 32886 | ATP synthase subunit gamma mitochondrial OS=Mus musculus OX=10090 GN=Atp5f1c PE=1 SV=1 |
| 5 | 7 | Q3UD06\|Q3UD06_MOUSE | 176.31 | 39 | 39 | 1.08E+08 | 13 | 13 | 36 | Carbamidomethylation; Deamidation (NQ); Pyro-glu from Q | 32886 | ATP synthase subunit gamma OS=Mus musculus OX=10090 GN=Atp5c1 PE=1 SV=1 |
| 14 | 101 | sp\|P43274\|H14_MOUSE | 169.95 | 37 | 37 | 2.26E+07 | 10 | 3 | 29 | Acetylation (Protein N-term); Deamidation (NQ) | 21977 | Histone H1.4 OS=Mus musculus OX=10090 GN=H1-4 PE=1 SV=2 |
| 12 | 14 | sp\|P48962\|ADT1_MOUSE | 164.54 | 35 | 35 | 1.80E+07 | 12 | 5 | 31 | Carbamidomethylation; Acetylation (Protein N-term); Deamidation (NQ); Oxidation (M); Pyro-glu from Q | 32904 | ADP/ATP translocase 1 OS=Mus musculus OX=10090 GN=Slc25a4 PE=1 SV=4 |
| 11 | 12 | Q3V235\|Q3V235_MOUSE | 162.14 | 44 | 44 | 4.95E+07 | 16 | 16 | 31 | Acetylation (Protein N-term); Pyro-glu from Q | 33296 | Prohibitin OS=Mus musculus OX=10090 GN=Phb2 PE=1 SV=1 |
| 11 | 13 | sp\|O35129\|PHB2_MOUSE | 162.14 | 44 | 44 | 4.95E+07 | 16 | 16 | 31 | Acetylation (Protein N-term); Pyro-glu from Q | 33296 | Prohibitin-2 OS=Mus musculus OX=10090 GN=Phb2 PE=1 SV=1 |
| 19 | 16 | Q3TRH3\|Q3TRH3_MOUSE | 161.77 | 17 | 17 | 4.25E+07 | 12 | 10 | 21 | Carbamidomethylation | 70901 | Uncharacterized protein OS=Mus musculus OX=10090 GN=Hspa8 PE=2 SV=1 |
| 19 | 17 | Q504P4\|Q504P4_MOUSE | 161.77 | 18 | 18 | 4.25E+07 | 12 | 10 | 21 | Carbamidomethylation | 68779 | Heat shock cognate 71 kDa protein OS=Mus musculus OX=10090 GN=Hspa8 PE=1 SV=1 |
| 19 | 20 | Q3UBA6\|Q3UBA6_MOUSE | 161.77 | 17 | 17 | 4.25E+07 | 12 | 10 | 21 | Carbamidomethylation | 70899 | Uncharacterized protein OS=Mus musculus OX=10090 GN=Hspa8 PE=2 SV=1 |
| 19 | 18 | sp\|P63017\|HSP7C_MOUSE | 161.77 | 17 | 17 | 4.25E+07 | 12 | 10 | 21 | Carbamidomethylation | 70871 | Heat shock cognate 71 kDa protein OS=Mus musculus OX=10090 GN=Hspa8 PE=1 SV=1 |
| 19 | 15 | Q3TEK2\|Q3TEK2_MOUSE | 161.77 | 17 | 17 | 4.25E+07 | 12 | 10 | 21 | Carbamidomethylation | 70857 | Uncharacterized protein OS=Mus musculus OX=10090 GN=Hspa8 PE=2 SV=1 |
| 19 | 21 | Q3TH56\|Q3TH56_MOUSE | 161.77 | 17 | 17 | 4.25E+07 | 12 | 10 | 21 | Carbamidomethylation | 70872 | Uncharacterized protein OS=Mus musculus OX=10090 GN=Hspa8 PE=2 SV=1 |
| 19 | 19 | Q3TQ13\|Q3TQ13_MOUSE | 161.77 | 17 | 17 | 4.25E+07 | 12 | 10 | 21 | Carbamidomethylation | 70871 | Uncharacterized protein OS=Mus musculus OX=10090 GN=Hspa8 PE=2 SV=1 |
| 23 | 25 | Q9CXK3\|Q9CXK3_MOUSE | 157.35 | 31 | 31 | 1.41E+07 | 11 | 3 | 18 | Oxidation (M); Pyro-glu from Q | 41947 | Uncharacterized protein OS=Mus musculus OX=10090 GN=Actc1 PE=2 SV=1 |
| 23 | 31 | Q3TG92\|Q3TG92_MOUSE | 157.35 | 31 | 31 | 1.41E+07 | 11 | 3 | 18 | Oxidation (M); Pyro-glu from Q | 42047 | Uncharacterized protein OS=Mus musculus OX=10090 GN=Actc1 PE=2 SV=1 |
| 23 | 29 | sp\|P68134\|ACTS_MOUSE | 157.35 | 31 | 31 | 1.41E+07 | 11 | 3 | 18 | Oxidation (M); Pyro-glu from Q | 42051 | Actin alpha skeletal muscle OS=Mus musculus OX=10090 GN=Acta1 PE=1 SV=1 |
| 23 | 26 | sp\|P68033\|ACTC_MOUSE | 157.35 | 31 | 31 | 1.41E+07 | 11 | 3 | 18 | Oxidation (M); Pyro-glu from Q | 42019 | Actin alpha cardiac muscle 1 OS=Mus musculus OX=10090 GN=Actc1 PE=1 SV=1 |
| 23 | 27 | sp\|P62737\|ACTA_MOUSE | 157.35 | 31 | 31 | 1.41E+07 | 11 | 3 | 18 | Oxidation (M); Pyro-glu from Q | 42009 | Actin aortic smooth muscle OS=Mus musculus OX=10090 GN=Acta2 PE=1 SV=1 |
| 23 | 28 | Q497E4\|Q497E4_MOUSE | 157.35 | 31 | 31 | 1.41E+07 | 11 | 3 | 18 | Oxidation (M); Pyro-glu from Q | 42019 | Actin alpha cardiac muscle 1 OS=Mus musculus OX=10090 GN=Actc1 PE=2 SV=1 |
| 3 | 249 | A0A0M4KM70\|A0A0M4KM70_MOUSE | 150.88 | 17 | 17 | 8.98E+08 | 4 | 2 | 41 | Carbamidomethylation; Deamidation (NQ); Oxidation (M) | 50295 | Monoclonal 11D8 anti-human butyrylcholinesterase (BChE) heavy chain OS=Mus musculus OX=10090 PE=2 SV=1 |
| 3 | 251 | Q99LC4\|Q99LC4_MOUSE | 150.88 | 16 | 16 | 8.98E+08 | 4 | 2 | 41 | Carbamidomethylation; Deamidation (NQ); Oxidation (M) | 51008 | Igh protein OS=Mus musculus OX=10090 GN=Igh PE=1 SV=1 |
| 3 | 250 | U5LP42\|U5LP42_MOUSE | 150.88 | 16 | 16 | 8.98E+08 | 4 | 2 | 41 | Carbamidomethylation; Deamidation (NQ); Oxidation (M) | 51051 | Anti-H5N1 hemagglutinin monoclonal anitbody H5M9 heavy chain (Fragment) OS=Mus musculus OX=10090 PE=2 SV=1 |
| 3 | 288 | sp\|P01868\|IGHG1_MOUSE | 150.88 | 23 | 23 | 8.98E+08 | 4 | 2 | 41 | Carbamidomethylation; Deamidation (NQ); Oxidation (M) | 35705 | Ig gamma-1 chain C region secreted form OS=Mus musculus OX=10090 GN=Ighg1 PE=1 SV=1 |
| 3 | 289 | sp\|P01869\|IGH1M_MOUSE | 150.88 | 19 | 19 | 8.98E+08 | 4 | 2 | 41 | Carbamidomethylation; Deamidation (NQ); Oxidation (M) | 43387 | Ig gamma-1 chain C region membrane-bound form OS=Mus musculus OX=10090 GN=Ighg1 PE=1 SV=2 |
| 26 | 43 | Q4KL81\|Q4KL81_MOUSE | 148.97 | 27 | 27 | 5.97E+06 | 10 | 2 | 16 | Oxidation (M) | 41793 | Actin gamma cytoplasmic 1 OS=Mus musculus OX=10090 GN=Actg1 PE=2 SV=1 |
| 26 | 44 | Q3U5R4\|Q3U5R4_MOUSE | 148.97 | 27 | 27 | 5.97E+06 | 10 | 2 | 16 | Oxidation (M) | 41709 | Uncharacterized protein OS=Mus musculus OX=10090 GN=Actb PE=2 SV=1 |
| 26 | 45 | sp\|P63260\|ACTG_MOUSE | 148.97 | 27 | 27 | 5.97E+06 | 10 | 2 | 16 | Oxidation (M) | 41793 | Actin cytoplasmic 2 OS=Mus musculus OX=10090 GN=Actg1 PE=1 SV=1 |
| 26 | 42 | Q3UAF6\|Q3UAF6_MOUSE | 148.97 | 27 | 27 | 5.97E+06 | 10 | 2 | 16 | Oxidation (M) | 41811 | Uncharacterized protein OS=Mus musculus OX=10090 GN=Actb PE=2 SV=1 |
| 26 | 46 | sp\|P60710\|ACTB_MOUSE | 148.97 | 27 | 27 | 5.97E+06 | 10 | 2 | 16 | Oxidation (M) | 41737 | Actin cytoplasmic 1 OS=Mus musculus OX=10090 GN=Actb PE=1 SV=1 |
| 26 | 47 | B2RRX1\|B2RRX1_MOUSE | 148.97 | 27 | 27 | 5.97E+06 | 10 | 2 | 16 | Oxidation (M) | 41737 | Actin beta OS=Mus musculus OX=10090 GN=Actb PE=2 SV=1 |
| 26 | 48 | Q3UAF7\|Q3UAF7_MOUSE | 148.97 | 27 | 27 | 5.97E+06 | 10 | 2 | 16 | Oxidation (M) | 41751 | Uncharacterized protein OS=Mus musculus OX=10090 GN=Actb PE=2 SV=1 |
| 16 | 75 | Q3U6K8\|Q3U6K8_MOUSE | 144.02 | 26 | 26 | 5.68E+07 | 8 | 7 | 19 | Carbamidomethylation; Acetylation (Protein N-term); Deamidation (NQ) | 30725 | Uncharacterized protein OS=Mus musculus OX=10090 GN=Vdac1 PE=2 SV=1 |
| 16 | 76 | sp\|Q60932\|VDAC1_MOUSE | 144.02 | 25 | 25 | 5.68E+07 | 8 | 7 | 19 | Carbamidomethylation; Acetylation (Protein N-term); Deamidation (NQ) | 32351 | Voltage-dependent anion-selective channel protein 1 OS=Mus musculus OX=10090 GN=Vdac1 PE=1 SV=3 |
| 31 | 137 | Q1WWK3\|Q1WWK3_MOUSE | 143.6 | 27 | 27 | 1.44E+07 | 7 | 5 | 14 | Acetylation (Protein N-term) | 22445 | Hist1h1b protein (Fragment) OS=Mus musculus OX=10090 GN=H1f5 PE=2 SV=1 |
| 31 | 138 | sp\|P43276\|H15_MOUSE | 143.6 | 27 | 27 | 1.44E+07 | 7 | 5 | 14 | Acetylation (Protein N-term) | 22576 | Histone H1.5 OS=Mus musculus OX=10090 GN=H1-5 PE=1 SV=2 |
| 28 | 128 | sp\|Q9DCT2\|NDUS3_MOUSE | 140.18 | 27 | 27 | 6.41E+07 | 7 | 7 | 16 | Pyro-glu from Q | 30149 | NADH dehydrogenase [ubiquinone] iron-sulfur protein 3 mitochondrial OS=Mus musculus OX=10090 GN=Ndufs3 PE=1 SV=2 |
| 20 | 22 | sp\|P67778\|PHB_MOUSE | 139.24 | 38 | 38 | 5.19E+07 | 11 | 11 | 19 | Pyro-glu from Q | 29820 | Prohibitin OS=Mus musculus OX=10090 GN=Phb PE=1 SV=1 |
| 25 | 98 | D2KHZ9\|D2KHZ9_MOUSE | 136.97 | 23 | 23 | 5.72E+07 | 7 | 7 | 16 | Carbamidomethylation; Deamidation (NQ); Oxidation (M) | 35810 | Glyceraldehyde-3-phosphate dehydrogenase OS=Mus musculus OX=10090 GN=GAPDH PE=2 SV=1 |
| 25 | 97 | sp\|P16858\|G3P_MOUSE | 136.97 | 23 | 23 | 5.72E+07 | 7 | 7 | 16 | Carbamidomethylation; Deamidation (NQ); Oxidation (M) | 35810 | Glyceraldehyde-3-phosphate dehydrogenase OS=Mus musculus OX=10090 GN=Gapdh PE=1 SV=2 |
| 25 | 99 | A0A0A0MQF6\|A0A0A0MQF6_MOUSE | 136.97 | 21 | 21 | 5.72E+07 | 7 | 7 | 16 | Carbamidomethylation; Deamidation (NQ); Oxidation (M) | 38653 | Glyceraldehyde-3-phosphate dehydrogenase OS=Mus musculus OX=10090 GN=Gapdh PE=1 SV=1 |
| 33 | 67 | B2RQQ1\|B2RQQ1_MOUSE | 136.12 | 6 | 6 | 1.41E+07 | 13 | 13 | 14 | Deamidation (NQ); Pyro-glu from Q | 223563 | MCG133649 isoform CRA_a OS=Mus musculus OX=10090 GN=Myh6 PE=1 SV=1 |
| 33 | 68 | sp\|Q02566\|MYH6_MOUSE | 136.12 | 6 | 6 | 1.41E+07 | 13 | 13 | 14 | Deamidation (NQ); Pyro-glu from Q | 223563 | Myosin-6 OS=Mus musculus OX=10090 GN=Myh6 PE=1 SV=2 |
| 33 | 69 | Q2TAW4\|Q2TAW4_MOUSE | 136.12 | 6 | 6 | 1.41E+07 | 13 | 13 | 14 | Deamidation (NQ); Pyro-glu from Q | 223562 | Myosin heavy polypeptide 6 cardiac muscle alpha OS=Mus musculus OX=10090 GN=Myh6 PE=2 SV=1 |
| 43 | 82 | sp\|Q9DB77\|QCR2_MOUSE | 128.81 | 17 | 17 | 1.79E+07 | 7 | 7 | 9 |  | 48235 | Cytochrome b-c1 complex subunit 2 mitochondrial OS=Mus musculus OX=10090 GN=Uqcrc2 PE=1 SV=1 |
| 27 | 91 | sp\|Q9DCW4\|ETFB_MOUSE | 125.95 | 33 | 33 | 2.99E+07 | 8 | 8 | 16 | Carbamidomethylation | 27623 | Electron transfer flavoprotein subunit beta OS=Mus musculus OX=10090 GN=Etfb PE=1 SV=3 |
| 37 | 55 | sp\|P62908\|RS3_MOUSE | 124.16 | 44 | 44 | 1.68E+07 | 10 | 10 | 12 | Carbamidomethylation | 26674 | 40S ribosomal protein S3 OS=Mus musculus OX=10090 GN=Rps3 PE=1 SV=1 |
| 37 | 57 | Q9CZP6\|Q9CZP6_MOUSE | 124.16 | 44 | 44 | 1.68E+07 | 10 | 10 | 12 | Carbamidomethylation | 26602 | KH type-2 domain-containing protein OS=Mus musculus OX=10090 GN=Rps3 PE=2 SV=1 |
| 37 | 56 | Q5YLW3\|Q5YLW3_MOUSE | 124.16 | 44 | 44 | 1.68E+07 | 10 | 10 | 12 | Carbamidomethylation | 26674 | Ribosomal protein S3 OS=Mus musculus OX=10090 GN=Rps3 PE=1 SV=1 |
| 37 | 79 | Q9D0A2\|Q9D0A2_MOUSE | 124.16 | 44 | 44 | 1.68E+07 | 10 | 10 | 12 | Carbamidomethylation | 26640 | KH type-2 domain-containing protein OS=Mus musculus OX=10090 GN=Rps3 PE=2 SV=1 |
| 39 | 64 | sp\|P08249\|MDHM_MOUSE | 119.77 | 27 | 27 | 1.82E+07 | 9 | 9 | 11 | Carbamidomethylation | 35611 | Malate dehydrogenase mitochondrial OS=Mus musculus OX=10090 GN=Mdh2 PE=1 SV=3 |
| 35 | 122 | sp\|P10126\|EF1A1_MOUSE | 116.47 | 14 | 14 | 1.65E+07 | 7 | 7 | 12 | Pyro-glu from Q | 50114 | Elongation factor 1-alpha 1 OS=Mus musculus OX=10090 GN=Eef1a1 PE=1 SV=3 |
| 35 | 123 | Q3UA81\|Q3UA81_MOUSE | 116.47 | 14 | 14 | 1.65E+07 | 7 | 7 | 12 | Pyro-glu from Q | 50113 | Elongation factor 1-alpha OS=Mus musculus OX=10090 GN=Eef1a1 PE=2 SV=1 |
| 35 | 124 | Q3UZQ3\|Q3UZQ3_MOUSE | 116.47 | 14 | 14 | 1.65E+07 | 7 | 7 | 12 | Pyro-glu from Q | 50066 | Elongation factor 1-alpha OS=Mus musculus OX=10090 GN=Eef1a1 PE=2 SV=1 |
| 35 | 125 | Q3TII3\|Q3TII3_MOUSE | 116.47 | 14 | 14 | 1.65E+07 | 7 | 7 | 12 | Pyro-glu from Q | 50104 | Elongation factor 1-alpha OS=Mus musculus OX=10090 GN=Eef1a1 PE=2 SV=1 |
| 35 | 121 | Q58E64\|Q58E64_MOUSE | 116.47 | 14 | 14 | 1.65E+07 | 7 | 7 | 12 | Pyro-glu from Q | 50114 | Elongation factor 1-alpha OS=Mus musculus OX=10090 GN=Eef1a1 PE=1 SV=1 |
| 18 | 94 | sp\|P51881\|ADT2_MOUSE | 115.6 | 20 | 20 | 6.98E+06 | 8 | 1 | 22 | Oxidation (M) | 32931 | ADP/ATP translocase 2 OS=Mus musculus OX=10090 GN=Slc25a5 PE=1 SV=3 |
| 18 | 95 | Q545A2\|Q545A2_MOUSE | 115.6 | 20 | 20 | 6.98E+06 | 8 | 1 | 22 | Oxidation (M) | 32931 | MCG11560 OS=Mus musculus OX=10090 GN=Slc25a5 PE=1 SV=1 |
| 63 | 178 | sp\|P17751\|TPIS_MOUSE | 113.71 | 18 | 18 | 5.02E+06 | 5 | 5 | 5 | Carbamidomethylation | 32192 | Triosephosphate isomerase OS=Mus musculus OX=10090 GN=Tpi1 PE=1 SV=4 |
| 30 | 70 | sp\|P62259\|1433E_MOUSE | 113.45 | 31 | 31 | 2.61E+07 | 9 | 8 | 13 | Acetylation (Protein N-term); Pyro-glu from Q | 29174 | 14-3-3 protein epsilon OS=Mus musculus OX=10090 GN=Ywhae PE=1 SV=1 |
| 30 | 71 | Q8BPH1\|Q8BPH1_MOUSE | 113.45 | 31 | 31 | 2.61E+07 | 9 | 8 | 13 | Acetylation (Protein N-term); Pyro-glu from Q | 29189 | 14_3_3 domain-containing protein OS=Mus musculus OX=10090 GN=Ywhae PE=2 SV=1 |
| 30 | 72 | Q5SS40\|Q5SS40_MOUSE | 113.45 | 31 | 31 | 2.61E+07 | 9 | 8 | 13 | Acetylation (Protein N-term); Pyro-glu from Q | 29174 | Tyrosine 3-monooxygenase/tryptophan 5-monooxygenase activation protein epsilon polypeptide isoform CRA_c OS=Mus musculus OX=10090 GN=Ywhae PE=1 SV=1 |
| 36 | 179 | sp\|P43275\|H11_MOUSE | 110.34 | 30 | 30 | 3.96E+06 | 7 | 4 | 12 | Acetylation (Protein N-term); Deamidation (NQ) | 21785 | Histone H1.1 OS=Mus musculus OX=10090 GN=H1-1 PE=1 SV=2 |
| 32 | 112 | Q3TJD4\|Q3TJD4_MOUSE | 108.65 | 20 | 20 | 1.93E+07 | 7 | 7 | 13 | Carbamidomethylation; Pyro-glu from Q | 28948 | Uncharacterized protein OS=Mus musculus OX=10090 GN=Atp5pb PE=2 SV=1 |
| 32 | 113 | sp\|Q9CQQ7\|AT5F1_MOUSE | 108.65 | 20 | 20 | 1.93E+07 | 7 | 7 | 13 | Carbamidomethylation; Pyro-glu from Q | 28949 | ATP synthase F(0) complex subunit B1 mitochondrial OS=Mus musculus OX=10090 GN=Atp5pb PE=1 SV=1 |
| 32 | 111 | Q3UF04\|Q3UF04_MOUSE | 108.65 | 20 | 20 | 1.93E+07 | 7 | 7 | 13 | Carbamidomethylation; Pyro-glu from Q | 28919 | Uncharacterized protein OS=Mus musculus OX=10090 GN=Atp5pb PE=2 SV=1 |
| 32 | 114 | Q5I0W0\|Q5I0W0_MOUSE | 108.65 | 20 | 20 | 1.93E+07 | 7 | 7 | 13 | Carbamidomethylation; Pyro-glu from Q | 28949 | ATP synthase H+ transporting mitochondrial F0 complex subunit b isoform 1 OS=Mus musculus OX=10090 GN=Atp5pb PE=1 SV=1 |
| 53 | 153 | sp\|Q99LC5\|ETFA_MOUSE | 105.9 | 12 | 12 | 1.06E+07 | 4 | 4 | 6 | Carbamidomethylation | 35009 | Electron transfer flavoprotein subunit alpha mitochondrial OS=Mus musculus OX=10090 GN=Etfa PE=1 SV=2 |
| 34 | 172 | Q08EK4\|Q08EK4_MOUSE | 100.4 | 7 | 7 | 5.38E+05 | 6 | 1 | 12 | Deamidation (NQ) | 61302 | Keratin 77 OS=Mus musculus OX=10090 GN=Krt77 PE=2 SV=1 |
| 34 | 174 | Q08EK5\|Q08EK5_MOUSE | 100.4 | 7 | 7 | 5.38E+05 | 6 | 1 | 12 | Deamidation (NQ) | 61359 | Keratin 77 OS=Mus musculus OX=10090 GN=Krt77 PE=1 SV=1 |
| 34 | 175 | sp\|Q6IFZ6\|K2C1B_MOUSE | 100.4 | 7 | 7 | 5.38E+05 | 6 | 1 | 12 | Deamidation (NQ) | 61359 | Keratin type II cytoskeletal 1b OS=Mus musculus OX=10090 GN=Krt77 PE=1 SV=1 |
| 47 | 267 | Q3TWG9\|Q3TWG9_MOUSE | 99.33 | 7 | 7 | 1.73E+07 | 3 | 3 | 8 |  | 46530 | SERPIN domain-containing protein OS=Mus musculus OX=10090 GN=Serpinh1 PE=2 SV=1 |
| 47 | 268 | Q3TJK3\|Q3TJK3_MOUSE | 99.33 | 7 | 7 | 1.73E+07 | 3 | 3 | 8 |  | 46535 | SERPIN domain-containing protein OS=Mus musculus OX=10090 GN=Serpinh1 PE=2 SV=1 |
| 47 | 269 | Q3TMD2\|Q3TMD2_MOUSE | 99.33 | 7 | 7 | 1.73E+07 | 3 | 3 | 8 |  | 46520 | SERPIN domain-containing protein OS=Mus musculus OX=10090 GN=Serpinh1 PE=2 SV=1 |
| 47 | 270 | Q8BVU9\|Q8BVU9_MOUSE | 99.33 | 7 | 7 | 1.73E+07 | 3 | 3 | 8 |  | 46511 | SERPIN domain-containing protein OS=Mus musculus OX=10090 GN=Serpinh1 PE=2 SV=1 |
| 47 | 271 | sp\|P19324\|SERPH_MOUSE | 99.33 | 7 | 7 | 1.73E+07 | 3 | 3 | 8 |  | 46534 | Serpin H1 OS=Mus musculus OX=10090 GN=Serpinh1 PE=1 SV=3 |
| 47 | 272 | Q8BV87\|Q8BV87_MOUSE | 99.33 | 7 | 7 | 1.73E+07 | 3 | 3 | 8 |  | 46520 | SERPIN domain-containing protein OS=Mus musculus OX=10090 GN=Serpinh1 PE=2 SV=1 |
| 70 | 191 | Q3UFI4\|Q3UFI4_MOUSE | 94.4 | 12 | 12 | 5.59E+06 | 4 | 4 | 4 |  | 33529 | 60S ribosomal protein L6 OS=Mus musculus OX=10090 GN=Rpl6 PE=2 SV=1 |
| 70 | 192 | sp\|P47911\|RL6_MOUSE | 94.4 | 12 | 12 | 5.59E+06 | 4 | 4 | 4 |  | 33510 | 60S ribosomal protein L6 OS=Mus musculus OX=10090 GN=Rpl6 PE=1 SV=3 |
| 70 | 193 | Q3UCH0\|Q3UCH0_MOUSE | 94.4 | 12 | 12 | 5.59E+06 | 4 | 4 | 4 |  | 33510 | 60S ribosomal protein L6 OS=Mus musculus OX=10090 GN=Rpl6 PE=1 SV=1 |
| 55 | 225 | Q3U4Y0\|Q3U4Y0_MOUSE | 94.35 | 21 | 21 | 9.00E+06 | 4 | 4 | 6 | Acetylation (Protein N-term) | 20847 | H15 domain-containing protein OS=Mus musculus OX=10090 GN=H1f0 PE=2 SV=1 |
| 55 | 204 | sp\|P10922\|H10_MOUSE | 94.35 | 21 | 21 | 9.00E+06 | 4 | 4 | 6 | Acetylation (Protein N-term) | 20861 | Histone H1.0 OS=Mus musculus OX=10090 GN=H1-0 PE=2 SV=4 |
| 55 | 235 | Q8C1Y3\|Q8C1Y3_MOUSE | 94.35 | 22 | 22 | 9.00E+06 | 4 | 4 | 6 | Acetylation (Protein N-term) | 19253 | H15 domain-containing protein OS=Mus musculus OX=10090 GN=H1f0 PE=2 SV=1 |
| 38 | 108 | sp\|Q03265\|ATPA_MOUSE | 94.18 | 10 | 10 | 9.41E+06 | 6 | 5 | 8 | Pyro-glu from Q | 59753 | ATP synthase subunit alpha mitochondrial OS=Mus musculus OX=10090 GN=Atp5f1a PE=1 SV=1 |
| 67 | 252 | sp\|Q9DB20\|ATPO_MOUSE | 91.62 | 14 | 14 | 5.40E+06 | 3 | 3 | 3 |  | 23364 | ATP synthase subunit O mitochondrial OS=Mus musculus OX=10090 GN=Atp5po PE=1 SV=1 |
| 67 | 253 | Q3TF25\|Q3TF25_MOUSE | 91.62 | 14 | 14 | 5.40E+06 | 3 | 3 | 3 |  | 23364 | Uncharacterized protein OS=Mus musculus OX=10090 GN=Atp5o PE=2 SV=1 |
| 56 | 188 | sp\|Q9CR62\|M2OM_MOUSE | 91.18 | 18 | 18 | 4.66E+06 | 5 | 5 | 5 | Acetylation (Protein N-term) | 34155 | Mitochondrial 2-oxoglutarate/malate carrier protein OS=Mus musculus OX=10090 GN=Slc25a11 PE=1 SV=3 |
| 56 | 189 | Q5SX53\|Q5SX53_MOUSE | 91.18 | 18 | 18 | 4.66E+06 | 5 | 5 | 5 | Acetylation (Protein N-term) | 34155 | Solute carrier family 25 (Mitochondrial carrier oxoglutarate carrier) member 11 isoform CRA_b OS=Mus musculus OX=10090 GN=Slc25a11 PE=1 SV=1 |
| 52 | 154 | sp\|Q99JY0\|ECHB_MOUSE | 88.86 | 10 | 10 | 7.92E+06 | 5 | 5 | 7 |  | 51386 | Trifunctional enzyme subunit beta mitochondrial OS=Mus musculus OX=10090 GN=Hadhb PE=1 SV=1 |
| 50 | 145 | A0A0A6YWC8\|A0A0A6YWC8_MOUSE | 88.56 | 16 | 16 | 3.21E+06 | 7 | 5 | 8 | Pyro-glu from Q | 49193 | Vimentin OS=Mus musculus OX=10090 GN=Vim PE=1 SV=1 |
| 50 | 147 | Q3TWV0\|Q3TWV0_MOUSE | 88.56 | 15 | 15 | 3.21E+06 | 7 | 5 | 8 | Pyro-glu from Q | 53666 | IF rod domain-containing protein OS=Mus musculus OX=10090 GN=Vim PE=2 SV=1 |
| 50 | 148 | Q3UAX1\|Q3UAX1_MOUSE | 88.56 | 15 | 15 | 3.21E+06 | 7 | 5 | 8 | Pyro-glu from Q | 53558 | IF rod domain-containing protein OS=Mus musculus OX=10090 GN=Vim PE=2 SV=1 |
| 50 | 149 | sp\|P20152\|VIME_MOUSE | 88.56 | 15 | 15 | 3.21E+06 | 7 | 5 | 8 | Pyro-glu from Q | 53688 | Vimentin OS=Mus musculus OX=10090 GN=Vim PE=1 SV=3 |
| 50 | 150 | Q5FWJ3\|Q5FWJ3_MOUSE | 88.56 | 15 | 15 | 3.21E+06 | 7 | 5 | 8 | Pyro-glu from Q | 53688 | Vimentin OS=Mus musculus OX=10090 GN=Vim PE=1 SV=1 |
| 50 | 151 | Q3TFD9\|Q3TFD9_MOUSE | 88.56 | 15 | 15 | 3.21E+06 | 7 | 5 | 8 | Pyro-glu from Q | 53689 | IF rod domain-containing protein OS=Mus musculus OX=10090 GN=Vim PE=2 SV=1 |
| 50 | 152 | Q3U6S1\|Q3U6S1_MOUSE | 88.56 | 15 | 15 | 3.21E+06 | 7 | 5 | 8 | Pyro-glu from Q | 53674 | IF rod domain-containing protein OS=Mus musculus OX=10090 GN=Vim PE=2 SV=1 |
| 50 | 146 | Q3V2S4\|Q3V2S4_MOUSE | 88.56 | 15 | 15 | 3.21E+06 | 7 | 5 | 8 | Pyro-glu from Q | 53748 | IF rod domain-containing protein OS=Mus musculus OX=10090 GN=Vim PE=2 SV=1 |
| 62 | 266 | Q3TXR5\|Q3TXR5_MOUSE | 87.41 | 14 | 14 | 7.73E+06 | 4 | 4 | 5 |  | 24206 | S5 DRBM domain-containing protein OS=Mus musculus OX=10090 GN=Rps2 PE=2 SV=1 |
| 62 | 273 | D3YVC1\|D3YVC1_MOUSE | 87.41 | 12 | 12 | 7.73E+06 | 4 | 4 | 5 |  | 28601 | 40S ribosomal protein S2 (Fragment) OS=Mus musculus OX=10090 GN=Rps2 PE=1 SV=1 |
| 62 | 274 | Q58EU3\|Q58EU3_MOUSE | 87.41 | 11 | 11 | 7.73E+06 | 4 | 4 | 5 |  | 31231 | MCG12811 isoform CRA_b OS=Mus musculus OX=10090 GN=Rps2 PE=1 SV=1 |
| 62 | 275 | Q3TI78\|Q3TI78_MOUSE | 87.41 | 11 | 11 | 7.73E+06 | 4 | 4 | 5 |  | 31209 | S5 DRBM domain-containing protein OS=Mus musculus OX=10090 GN=Rps2 PE=2 SV=1 |
| 62 | 277 | sp\|P25444\|RS2_MOUSE | 87.41 | 11 | 11 | 7.73E+06 | 4 | 4 | 5 |  | 31231 | 40S ribosomal protein S2 OS=Mus musculus OX=10090 GN=Rps2 PE=1 SV=3 |
| 62 | 276 | Q3TL20\|Q3TL20_MOUSE | 87.41 | 11 | 11 | 7.73E+06 | 4 | 4 | 5 |  | 31230 | S5 DRBM domain-containing protein OS=Mus musculus OX=10090 GN=Rps2 PE=2 SV=1 |
| 62 | 278 | Q3TXS9\|Q3TXS9_MOUSE | 87.41 | 11 | 11 | 7.73E+06 | 4 | 4 | 5 |  | 31188 | S5 DRBM domain-containing protein OS=Mus musculus OX=10090 GN=Rps2 PE=2 SV=1 |
| 62 | 279 | Q3TLE5\|Q3TLE5_MOUSE | 87.41 | 11 | 11 | 7.73E+06 | 4 | 4 | 5 |  | 31255 | S5 DRBM domain-containing protein OS=Mus musculus OX=10090 GN=Rps2 PE=2 SV=1 |
| 62 | 280 | Q3UB36\|Q3UB36_MOUSE | 87.41 | 11 | 11 | 7.73E+06 | 4 | 4 | 5 |  | 31682 | S5 DRBM domain-containing protein OS=Mus musculus OX=10090 GN=Rps2 PE=2 SV=1 |
| 62 | 281 | D3YWJ3\|D3YWJ3_MOUSE | 87.41 | 11 | 11 | 7.73E+06 | 4 | 4 | 5 |  | 32105 | 40S ribosomal protein S2 OS=Mus musculus OX=10090 GN=Rps2 PE=1 SV=1 |
| 40 | 243 | sp\|Q3UV17\|K22O_MOUSE | 84.74 | 5 | 5 | 6.58E+07 | 5 | 2 | 9 |  | 62845 | Keratin type II cytoskeletal 2 oral OS=Mus musculus OX=10090 GN=Krt76 PE=1 SV=1 |
| 46 | 205 | G5E902\|G5E902_MOUSE | 83.65 | 12 | 12 | 1.31E+07 | 4 | 4 | 7 | Carbamidomethylation | 39736 | MCG10343 isoform CRA_b OS=Mus musculus OX=10090 GN=Slc25a3 PE=1 SV=1 |
| 57 | 232 | sp\|P17182\|ENOA_MOUSE | 80.34 | 7 | 7 | 4.40E+06 | 4 | 4 | 4 |  | 47141 | Alpha-enolase OS=Mus musculus OX=10090 GN=Eno1 PE=1 SV=3 |
| 57 | 233 | Q5FW97\|Q5FW97_MOUSE | 80.34 | 7 | 7 | 4.40E+06 | 4 | 4 | 4 |  | 47141 | Enolase 1 alpha non-neuron OS=Mus musculus OX=10090 GN=EG433182 PE=1 SV=1 |
| 64 | 216 | sp\|Q61425\|HCDH_MOUSE | 79.97 | 10 | 10 | 4.38E+06 | 4 | 4 | 4 |  | 34464 | Hydroxyacyl-coenzyme A dehydrogenase mitochondrial OS=Mus musculus OX=10090 GN=Hadh PE=1 SV=2 |
| 71 | 195 | Q3UBI6\|Q3UBI6_MOUSE | 75.25 | 12 | 12 | 6.12E+06 | 4 | 4 | 4 |  | 31351 | Uncharacterized protein OS=Mus musculus OX=10090 GN=Rpl7 PE=2 SV=1 |
| 71 | 196 | sp\|P14148\|RL7_MOUSE | 75.25 | 12 | 12 | 6.12E+06 | 4 | 4 | 4 |  | 31420 | 60S ribosomal protein L7 OS=Mus musculus OX=10090 GN=Rpl7 PE=1 SV=2 |
| 71 | 197 | Q5M9N8\|Q5M9N8_MOUSE | 75.25 | 12 | 12 | 6.12E+06 | 4 | 4 | 4 |  | 31420 | Ribosomal protein L7 OS=Mus musculus OX=10090 GN=Rpl7 PE=1 SV=1 |
| 71 | 241 | F6XI62\|F6XI62_MOUSE | 75.25 | 12 | 12 | 6.12E+06 | 4 | 4 | 4 |  | 32536 | 60S ribosomal protein L7 (Fragment) OS=Mus musculus OX=10090 GN=Rpl7 PE=1 SV=1 |
| 84 | 203 | sp\|Q9D051\|ODPB_MOUSE | 74.99 | 6 | 6 | 2.59E+06 | 2 | 2 | 2 |  | 38937 | Pyruvate dehydrogenase E1 component subunit beta mitochondrial OS=Mus musculus OX=10090 GN=Pdhb PE=1 SV=1 |
| 58 | 206 | A2A5N1\|A2A5N1_MOUSE | 72.94 | 18 | 18 | 6.70E+05 | 3 | 1 | 5 |  | 18349 | 14-3-3 protein beta/alpha (Fragment) OS=Mus musculus OX=10090 GN=Ywhab PE=1 SV=1 |
| 58 | 207 | sp\|Q9CQV8\|1433B_MOUSE | 72.94 | 12 | 12 | 6.70E+05 | 3 | 1 | 5 |  | 28086 | 14-3-3 protein beta/alpha OS=Mus musculus OX=10090 GN=Ywhab PE=1 SV=3 |
| 58 | 208 | A2A5N2\|A2A5N2_MOUSE | 72.94 | 12 | 12 | 6.70E+05 | 3 | 1 | 5 |  | 28086 | Tyrosine 3-monooxygenase/tryptophan 5-monooxygenase activation protein beta polypeptide OS=Mus musculus OX=10090 GN=Ywhab PE=2 SV=1 |
| 101 | 486 | sp\|Q9CR57\|RL14_MOUSE | 69.97 | 10 | 10 | 1.31E+06 | 2 | 2 | 2 | Carbamidomethylation | 23564 | 60S ribosomal protein L14 OS=Mus musculus OX=10090 GN=Rpl14 PE=1 SV=3 |
| 101 | 487 | Q9CWK0\|Q9CWK0_MOUSE | 69.97 | 10 | 10 | 1.31E+06 | 2 | 2 | 2 | Carbamidomethylation | 26316 | Ribosomal_L14e domain-containing protein OS=Mus musculus OX=10090 GN=Rpl14 PE=2 SV=1 |
| 100 | 485 | Q9D8W6\|Q9D8W6_MOUSE | 68.64 | 8 | 8 | 3.63E+06 | 2 | 2 | 2 |  | 21194 | GTP:AMP phosphotransferase AK3 mitochondrial OS=Mus musculus OX=10090 GN=Ak3 PE=2 SV=1 |
| 100 | 488 | sp\|Q9WTP7\|KAD3_MOUSE | 68.64 | 7 | 7 | 3.63E+06 | 2 | 2 | 2 |  | 25426 | GTP:AMP phosphotransferase AK3 mitochondrial OS=Mus musculus OX=10090 GN=Ak3 PE=1 SV=3 |
| 65 | 352 | sp\|P42125\|ECI1_MOUSE | 66.84 | 13 | 13 | 8.08E+06 | 3 | 3 | 5 |  | 32250 | Enoyl-CoA delta isomerase 1 mitochondrial OS=Mus musculus OX=10090 GN=Eci1 PE=1 SV=2 |
| 148 | 442 | sp\|Q8BMF4\|ODP2_MOUSE | 66.45 | 2 | 2 | 1.36E+06 | 1 | 1 | 1 |  | 67942 | Dihydrolipoyllysine-residue acetyltransferase component of pyruvate dehydrogenase complex mitochondrial OS=Mus musculus OX=10090 GN=Dlat PE=1 SV=2 |
| 82 | 323 | sp\|Q9CPQ1\|COX6C_MOUSE | 66.3 | 36 | 36 | 2.00E+06 | 3 | 3 | 3 | Acetylation (Protein N-term) | 8469 | Cytochrome c oxidase subunit 6C OS=Mus musculus OX=10090 GN=Cox6c PE=1 SV=3 |
| 66 | 265 | sp\|P68510\|1433F_MOUSE | 65.89 | 10 | 10 | 0.00E+00 | 3 | 1 | 5 |  | 28212 | 14-3-3 protein eta OS=Mus musculus OX=10090 GN=Ywhah PE=1 SV=2 |
| 81 | 312 | sp\|P38647\|GRP75_MOUSE | 65.35 | 4 | 4 | 3.70E+06 | 3 | 2 | 3 |  | 73461 | Stress-70 protein mitochondrial OS=Mus musculus OX=10090 GN=Hspa9 PE=1 SV=3 |
| 59 | 337 | A0JLV3\|A0JLV3_MOUSE | 64.58 | 22 | 22 | 1.09E+07 | 3 | 3 | 6 | Pyro-glu from Q | 13579 | Histone H2B (Fragment) OS=Mus musculus OX=10090 GN=Hist1h2bj PE=2 SV=1 |
| 59 | 344 | sp\|Q64478\|H2B1H_MOUSE | 64.58 | 21 | 21 | 1.09E+07 | 3 | 3 | 6 | Pyro-glu from Q | 13920 | Histone H2B type 1-H OS=Mus musculus OX=10090 GN=Hist1h2bh PE=1 SV=3 |
| 59 | 338 | B2RVD5\|B2RVD5_MOUSE | 64.58 | 21 | 21 | 1.09E+07 | 3 | 3 | 6 | Pyro-glu from Q | 13920 | Histone H2B OS=Mus musculus OX=10090 GN=H2bc12 PE=2 SV=1 |
| 59 | 339 | B2RTK3\|B2RTK3_MOUSE | 64.58 | 21 | 21 | 1.09E+07 | 3 | 3 | 6 | Pyro-glu from Q | 13936 | Histone H2B OS=Mus musculus OX=10090 GN=H2bc14 PE=1 SV=1 |
| 59 | 340 | sp\|P10854\|H2B1M_MOUSE | 64.58 | 21 | 21 | 1.09E+07 | 3 | 3 | 6 | Pyro-glu from Q | 13936 | Histone H2B type 1-M OS=Mus musculus OX=10090 GN=H2bc14 PE=1 SV=2 |
| 59 | 341 | sp\|Q6ZWY9\|H2B1C_MOUSE | 64.58 | 21 | 21 | 1.09E+07 | 3 | 3 | 6 | Pyro-glu from Q | 13906 | Histone H2B type 1-C/E/G OS=Mus musculus OX=10090 GN=H2bc4 PE=1 SV=3 |
| 59 | 342 | sp\|Q64475\|H2B1B_MOUSE | 64.58 | 21 | 21 | 1.09E+07 | 3 | 3 | 6 | Pyro-glu from Q | 13952 | Histone H2B type 1-B OS=Mus musculus OX=10090 GN=Hist1h2bb PE=1 SV=3 |
| 59 | 345 | sp\|Q64525\|H2B2B_MOUSE | 64.58 | 21 | 21 | 1.09E+07 | 3 | 3 | 6 | Pyro-glu from Q | 13920 | Histone H2B type 2-B OS=Mus musculus OX=10090 GN=Hist2h2bb PE=1 SV=3 |
| 59 | 346 | sp\|P10853\|H2B1F_MOUSE | 64.58 | 21 | 21 | 1.09E+07 | 3 | 3 | 6 | Pyro-glu from Q | 13936 | Histone H2B type 1-F/J/L OS=Mus musculus OX=10090 GN=H2bc7 PE=1 SV=2 |
| 59 | 347 | sp\|Q8CGP1\|H2B1K_MOUSE | 64.58 | 21 | 21 | 1.09E+07 | 3 | 3 | 6 | Pyro-glu from Q | 13920 | Histone H2B type 1-K OS=Mus musculus OX=10090 GN=H2bc12 PE=1 SV=3 |
| 59 | 343 | sp\|Q8CGP2\|H2B1P_MOUSE | 64.58 | 21 | 21 | 1.09E+07 | 3 | 3 | 6 | Pyro-glu from Q | 13992 | Histone H2B type 1-P OS=Mus musculus OX=10090 GN=Hist1h2bp PE=1 SV=3 |
| 59 | 349 | A0JNS9\|A0JNS9_MOUSE | 64.58 | 21 | 21 | 1.09E+07 | 3 | 3 | 6 | Pyro-glu from Q | 14179 | Histone H2B OS=Mus musculus OX=10090 GN=H2bc1 PE=2 SV=1 |
| 59 | 348 | sp\|P70696\|H2B1A_MOUSE | 64.58 | 21 | 21 | 1.09E+07 | 3 | 3 | 6 | Pyro-glu from Q | 14237 | Histone H2B type 1-A OS=Mus musculus OX=10090 GN=H2bc1 PE=1 SV=3 |
| 59 | 350 | Q8CBB6\|Q8CBB6_MOUSE | 64.58 | 20 | 20 | 1.09E+07 | 3 | 3 | 6 | Pyro-glu from Q | 14888 | Histone H2B OS=Mus musculus OX=10090 GN=Hist1h2bq PE=2 SV=1 |
| 59 | 351 | Q921L4\|Q921L4_MOUSE | 64.58 | 20 | 20 | 1.09E+07 | 3 | 3 | 6 | Pyro-glu from Q | 14939 | Histone H2B OS=Mus musculus OX=10090 GN=LOC665622 PE=2 SV=1 |
| 74 | 405 | sp\|Q60931\|VDAC3_MOUSE | 61.18 | 7 | 7 | 1.24E+06 | 2 | 1 | 2 |  | 30753 | Voltage-dependent anion-selective channel protein 3 OS=Mus musculus OX=10090 GN=Vdac3 PE=1 SV=1 |
| 74 | 404 | Q3TX38\|Q3TX38_MOUSE | 61.18 | 7 | 7 | 1.24E+06 | 2 | 1 | 2 |  | 30753 | Uncharacterized protein OS=Mus musculus OX=10090 GN=Vdac3 PE=1 SV=1 |
| 74 | 407 | J3QMG3\|J3QMG3_MOUSE | 61.18 | 7 | 7 | 1.24E+06 | 2 | 1 | 2 |  | 30852 | Voltage-dependent anion-selective channel protein 3 OS=Mus musculus OX=10090 GN=Vdac3 PE=1 SV=1 |
| 74 | 406 | Q5EBQ0\|Q5EBQ0_MOUSE | 61.18 | 7 | 7 | 1.24E+06 | 2 | 1 | 2 |  | 30884 | Voltage-dependent anion channel 3 OS=Mus musculus OX=10090 GN=Vdac3 PE=2 SV=1 |
| 86 | 369 | B7ZP22\|B7ZP22_MOUSE | 60.11 | 6 | 6 | 1.55E+06 | 2 | 2 | 2 | Pyro-glu from Q | 35965 | Heterogeneous nuclear ribonucleoprotein A2/B1 OS=Mus musculus OX=10090 GN=Hnrnpa2b1 PE=2 SV=1 |
| 86 | 370 | sp\|O88569\|ROA2_MOUSE | 60.11 | 6 | 6 | 1.55E+06 | 2 | 2 | 2 | Pyro-glu from Q | 37403 | Heterogeneous nuclear ribonucleoproteins A2/B1 OS=Mus musculus OX=10090 GN=Hnrnpa2b1 PE=1 SV=2 |
| 85 | 418 | sp\|P12970\|RL7A_MOUSE | 58.89 | 6 | 6 | 1.43E+06 | 2 | 2 | 2 |  | 29977 | 60S ribosomal protein L7a OS=Mus musculus OX=10090 GN=Rpl7a PE=1 SV=2 |
| 85 | 417 | Q58ET1\|Q58ET1_MOUSE | 58.89 | 6 | 6 | 1.43E+06 | 2 | 2 | 2 |  | 29977 | MCG11348 OS=Mus musculus OX=10090 GN=Rpl7a PE=1 SV=1 |
| 85 | 419 | Q6P1A9\|Q6P1A9_MOUSE | 58.89 | 6 | 6 | 1.43E+06 | 2 | 2 | 2 |  | 30025 | Ribosomal protein L7A OS=Mus musculus OX=10090 GN=Rpl7a PE=2 SV=1 |
| 85 | 420 | Q80UT7\|Q80UT7_MOUSE | 58.89 | 6 | 6 | 1.43E+06 | 2 | 2 | 2 |  | 30474 | Rpl7a protein (Fragment) OS=Mus musculus OX=10090 GN=Rpl7a PE=2 SV=1 |
| 78 | 313 | Q3U7T8\|Q3U7T8_MOUSE | 55.81 | 3 | 3 | 1.61E+06 | 2 | 1 | 3 |  | 68498 | Uncharacterized protein (Fragment) OS=Mus musculus OX=10090 GN=Hspa5 PE=2 SV=1 |
| 78 | 314 | Q3TI47\|Q3TI47_MOUSE | 55.81 | 3 | 3 | 1.61E+06 | 2 | 1 | 3 |  | 72346 | Uncharacterized protein OS=Mus musculus OX=10090 GN=Hspa5 PE=2 SV=1 |
| 78 | 315 | Q3U9G2\|Q3U9G2_MOUSE | 55.81 | 3 | 3 | 1.61E+06 | 2 | 1 | 3 |  | 72406 | Uncharacterized protein OS=Mus musculus OX=10090 GN=Hspa5 PE=2 SV=1 |
| 78 | 316 | sp\|P20029\|BIP_MOUSE | 55.81 | 3 | 3 | 1.61E+06 | 2 | 1 | 3 |  | 72422 | Endoplasmic reticulum chaperone BiP OS=Mus musculus OX=10090 GN=Hspa5 PE=1 SV=3 |
| 78 | 318 | Q3TKF8\|Q3TKF8_MOUSE | 55.81 | 3 | 3 | 1.61E+06 | 2 | 1 | 3 |  | 72350 | Uncharacterized protein OS=Mus musculus OX=10090 GN=Hspa5 PE=2 SV=1 |
| 78 | 319 | Q3U6V3\|Q3U6V3_MOUSE | 55.81 | 3 | 3 | 1.61E+06 | 2 | 1 | 3 |  | 72382 | Uncharacterized protein OS=Mus musculus OX=10090 GN=Hspa5 PE=2 SV=1 |
| 78 | 317 | Q9DC41\|Q9DC41_MOUSE | 55.81 | 3 | 3 | 1.61E+06 | 2 | 1 | 3 |  | 72423 | Uncharacterized protein OS=Mus musculus OX=10090 GN=Hspa5 PE=2 SV=1 |
| 78 | 320 | Q3TWF2\|Q3TWF2_MOUSE | 55.81 | 3 | 3 | 1.61E+06 | 2 | 1 | 3 |  | 72463 | Uncharacterized protein OS=Mus musculus OX=10090 GN=Hspa5 PE=2 SV=1 |
| 72 | 392 | sp\|P48036\|ANXA5_MOUSE | 55.51 | 8 | 8 | 4.86E+06 | 3 | 3 | 4 |  | 35752 | Annexin A5 OS=Mus musculus OX=10090 GN=Anxa5 PE=1 SV=1 |
| 149 | 586 | Q5SW86\|Q5SW86_MOUSE | 53.14 | 10 | 10 | 8.37E+05 | 1 | 1 | 1 |  | 13598 | RAB1A member RAS oncogene family OS=Mus musculus OX=10090 GN=Rab1a PE=1 SV=1 |
| 149 | 587 | Q5SW87\|Q5SW87_MOUSE | 53.14 | 9 | 9 | 8.37E+05 | 1 | 1 | 1 |  | 15025 | RAB1A member RAS oncogene family OS=Mus musculus OX=10090 GN=Rab1a PE=1 SV=1 |
| 149 | 588 | Q5SW88\|Q5SW88_MOUSE | 53.14 | 6 | 6 | 8.37E+05 | 1 | 1 | 1 |  | 22372 | RAB1A member RAS oncogene family OS=Mus musculus OX=10090 GN=Rab1a PE=1 SV=1 |
| 149 | 590 | Q3UB66\|Q3UB66_MOUSE | 53.14 | 6 | 6 | 8.37E+05 | 1 | 1 | 1 |  | 22679 | Uncharacterized protein OS=Mus musculus OX=10090 GN=Rab1a PE=2 SV=1 |
| 149 | 591 | Q0PD67\|Q0PD67_MOUSE | 53.14 | 6 | 6 | 8.37E+05 | 1 | 1 | 1 |  | 22678 | RAB1 member RAS oncogene family isoform CRA_a OS=Mus musculus OX=10090 GN=Rab1a PE=1 SV=1 |
| 149 | 589 | sp\|P62821\|RAB1A_MOUSE | 53.14 | 6 | 6 | 8.37E+05 | 1 | 1 | 1 |  | 22678 | Ras-related protein Rab-1A OS=Mus musculus OX=10090 GN=Rab1A PE=1 SV=3 |
| 149 | 592 | Q6ZPF0\|Q6ZPF0_MOUSE | 53.14 | 5 | 5 | 8.37E+05 | 1 | 1 | 1 |  | 27335 | MKIAA3012 protein (Fragment) OS=Mus musculus OX=10090 GN=Rab1a PE=2 SV=1 |
| 106 | 549 | Q14BR4\|Q14BR4_MOUSE | 52.82 | 12 | 12 | 1.51E+06 | 2 | 2 | 2 |  | 20397 | ADP-ribosylation factor 4 OS=Mus musculus OX=10090 GN=Arf4 PE=1 SV=1 |
| 106 | 548 | sp\|P61750\|ARF4_MOUSE | 52.82 | 12 | 12 | 1.51E+06 | 2 | 2 | 2 |  | 20397 | ADP-ribosylation factor 4 OS=Mus musculus OX=10090 GN=Arf4 PE=1 SV=2 |
| 98 | 727 | sp\|O55143\|AT2A2_MOUSE | 52.72 | 1 | 1 | 1.20E+06 | 1 | 1 | 2 | Acetylation (Protein N-term) | 114858 | Sarcoplasmic/endoplasmic reticulum calcium ATPase 2 OS=Mus musculus OX=10090 GN=Atp2a2 PE=1 SV=2 |
| 98 | 738 | Q5DTI2\|Q5DTI2_MOUSE | 52.72 | 1 | 1 | 1.20E+06 | 1 | 1 | 2 | Acetylation (Protein N-term) | 116600 | ATPase Ca++ transporting cardiac muscle slow twitch 2 isoform CRA_b (Fragment) OS=Mus musculus OX=10090 GN=Atp2a2 PE=2 SV=1 |
| 151 | 599 | A0A1B0GSL5\|A0A1B0GSL5_MOUSE | 51.87 | 10 | 10 | 3.55E+05 | 1 | 1 | 1 | Acetylation (Protein N-term) | 11804 | 60S ribosomal protein L13a OS=Mus musculus OX=10090 GN=Rpl13a PE=1 SV=1 |
| 151 | 600 | A0A1B0GSC2\|A0A1B0GSC2_MOUSE | 51.87 | 9 | 9 | 3.55E+05 | 1 | 1 | 1 | Acetylation (Protein N-term) | 13960 | 60S ribosomal protein L13a (Fragment) OS=Mus musculus OX=10090 GN=Rpl13a PE=1 SV=1 |
| 151 | 601 | A0A1B0GRH1\|A0A1B0GRH1_MOUSE | 51.87 | 9 | 9 | 3.55E+05 | 1 | 1 | 1 | Acetylation (Protein N-term) | 14330 | 60S ribosomal protein L13a OS=Mus musculus OX=10090 GN=Rpl13a PE=1 SV=1 |
| 151 | 602 | A0A1B0GSB2\|A0A1B0GSB2_MOUSE | 51.87 | 7 | 7 | 3.55E+05 | 1 | 1 | 1 | Acetylation (Protein N-term) | 16450 | 60S ribosomal protein L13a OS=Mus musculus OX=10090 GN=Rpl13a PE=1 SV=1 |
| 151 | 603 | Q3TDS9\|Q3TDS9_MOUSE | 51.87 | 6 | 6 | 3.55E+05 | 1 | 1 | 1 | Acetylation (Protein N-term) | 20052 | Uncharacterized protein OS=Mus musculus OX=10090 GN=Rpl13a PE=2 SV=1 |
| 151 | 604 | Q5M9M0\|Q5M9M0_MOUSE | 51.87 | 5 | 5 | 3.55E+05 | 1 | 1 | 1 | Acetylation (Protein N-term) | 23464 | MCG23455 isoform CRA_e OS=Mus musculus OX=10090 GN=Rpl13a PE=1 SV=1 |
| 151 | 605 | sp\|P19253\|RL13A_MOUSE | 51.87 | 5 | 5 | 3.55E+05 | 1 | 1 | 1 | Acetylation (Protein N-term) | 23464 | 60S ribosomal protein L13a OS=Mus musculus OX=10090 GN=Rpl13a PE=1 SV=4 |
| 150 | 682 | A0A1L1SRW0\|A0A1L1SRW0_MOUSE | 51.55 | 14 | 14 | 6.67E+05 | 1 | 1 | 1 |  | 10126 | 40S ribosomal protein SA (Fragment) OS=Mus musculus OX=10090 GN=Rpsa PE=1 SV=1 |
| 150 | 684 | B2CY77\|B2CY77_MOUSE | 51.55 | 4 | 4 | 6.67E+05 | 1 | 1 | 1 |  | 32850 | Laminin receptor (Fragment) OS=Mus musculus OX=10090 GN=Rpsa PE=2 SV=1 |
| 150 | 683 | sp\|P14206\|RSSA_MOUSE | 51.55 | 4 | 4 | 6.67E+05 | 1 | 1 | 1 |  | 32838 | 40S ribosomal protein SA OS=Mus musculus OX=10090 GN=Rpsa PE=1 SV=4 |
| 103 | 518 | sp\|P00405\|COX2_MOUSE | 50.7 | 6 | 6 | 1.35E+07 | 2 | 2 | 2 |  | 25976 | Cytochrome c oxidase subunit 2 OS=Mus musculus OX=10090 GN=Mtco2 PE=1 SV=1 |
| 103 | 509 | A3E4B0\|A3E4B0_MOUSE | 50.7 | 6 | 6 | 1.35E+07 | 2 | 2 | 2 |  | 25946 | Cytochrome c oxidase subunit 2 OS=Mus musculus musculus OX=39442 GN=COX2 PE=3 SV=1 |
| 103 | 519 | A0A0F6PXF3\|A0A0F6PXF3_MOUSE | 50.7 | 6 | 6 | 1.35E+07 | 2 | 2 | 2 |  | 25976 | Cytochrome c oxidase subunit 2 OS=Mus musculus helgolandicus OX=1643390 GN=COXII PE=3 SV=1 |
| 103 | 510 | A3R481\|A3R481_MOUSE | 50.7 | 6 | 6 | 1.35E+07 | 2 | 2 | 2 |  | 25975 | Cytochrome c oxidase subunit 2 OS=Mus musculus domesticus OX=10092 GN=COXII PE=3 SV=1 |
| 103 | 511 | A3R455\|A3R455_MUSMC | 50.7 | 6 | 6 | 1.35E+07 | 2 | 2 | 2 |  | 25976 | Cytochrome c oxidase subunit 2 OS=Mus musculus castaneus OX=10091 GN=COXII PE=3 SV=1 |
| 103 | 520 | Q5GA81\|Q5GA81_MUSMM | 50.7 | 6 | 6 | 1.35E+07 | 2 | 2 | 2 |  | 25976 | Cytochrome c oxidase subunit 2 OS=Mus musculus molossinus OX=57486 GN=COX2 PE=3 SV=1 |
| 103 | 512 | Q7JCZ1\|Q7JCZ1_MOUSE | 50.7 | 6 | 6 | 1.35E+07 | 2 | 2 | 2 |  | 25976 | Cytochrome c oxidase subunit 2 OS=Mus musculus OX=10090 GN=mt-Co2 PE=1 SV=1 |
| 103 | 521 | A0A023J6F3\|A0A023J6F3_MOUSE | 50.7 | 6 | 6 | 1.35E+07 | 2 | 2 | 2 |  | 25976 | Cytochrome c oxidase subunit 2 OS=Mus musculus musculus OX=39442 GN=COX2 PE=3 SV=1 |
| 103 | 513 | K7XK22\|K7XK22_MOUSE | 50.7 | 6 | 6 | 1.35E+07 | 2 | 2 | 2 |  | 25990 | Cytochrome c oxidase subunit 2 OS=Mus musculus domesticus OX=10092 GN=COXII PE=3 SV=1 |
| 103 | 514 | K7XKA7\|K7XKA7_MOUSE | 50.7 | 6 | 6 | 1.35E+07 | 2 | 2 | 2 |  | 25962 | Cytochrome c oxidase subunit 2 OS=Mus musculus domesticus OX=10092 GN=COXII PE=3 SV=1 |
| 103 | 515 | A0A023J6I7\|A0A023J6I7_MUSMC | 50.7 | 6 | 6 | 1.35E+07 | 2 | 2 | 2 |  | 25990 | Cytochrome c oxidase subunit 2 OS=Mus musculus castaneus OX=10091 GN=COX2 PE=3 SV=1 |
| 103 | 516 | Q7JD03\|Q7JD03_MOUSE | 50.7 | 6 | 6 | 1.35E+07 | 2 | 2 | 2 |  | 25976 | Cytochrome c oxidase subunit 2 OS=Mus musculus domesticus OX=10092 GN=COX2 PE=3 SV=1 |
| 103 | 517 | A0A075DC90\|A0A075DC90_MOUSE | 50.7 | 6 | 6 | 1.35E+07 | 2 | 2 | 2 |  | 26003 | Cytochrome c oxidase subunit 2 OS=Mus musculus OX=10090 GN=COX2 PE=3 SV=1 |
| 83 | 394 | A1L0U3\|A1L0U3_MOUSE | 49.84 | 15 | 15 | 2.32E+06 | 3 | 3 | 3 |  | 15030 | Histone H3 (Fragment) OS=Mus musculus OX=10090 GN=Hist1h3e PE=2 SV=1 |
| 83 | 395 | F8WI35\|F8WI35_MOUSE | 49.84 | 15 | 15 | 2.32E+06 | 3 | 3 | 3 |  | 15199 | Histone H3 OS=Mus musculus OX=10090 GN=H3f3a PE=1 SV=1 |
| 83 | 396 | A1L0V4\|A1L0V4_MOUSE | 49.84 | 15 | 15 | 2.32E+06 | 3 | 3 | 3 |  | 15273 | Histone H3 (Fragment) OS=Mus musculus OX=10090 GN=Hist1h3i PE=2 SV=1 |
| 83 | 397 | B9EI85\|B9EI85_MOUSE | 49.84 | 15 | 15 | 2.32E+06 | 3 | 3 | 3 |  | 15388 | Histone H3 OS=Mus musculus OX=10090 GN=Hist2h3b PE=2 SV=1 |
| 83 | 398 | sp\|P68433\|H31_MOUSE | 49.84 | 15 | 15 | 2.32E+06 | 3 | 3 | 3 |  | 15404 | Histone H3.1 OS=Mus musculus OX=10090 GN=H3c1 PE=1 SV=2 |
| 83 | 400 | sp\|P84244\|H33_MOUSE | 49.84 | 15 | 15 | 2.32E+06 | 3 | 3 | 3 |  | 15328 | Histone H3.3 OS=Mus musculus OX=10090 GN=H3-3a PE=1 SV=2 |
| 83 | 401 | sp\|P84228\|H32_MOUSE | 49.84 | 15 | 15 | 2.32E+06 | 3 | 3 | 3 |  | 15388 | Histone H3.2 OS=Mus musculus OX=10090 GN=Hist1h3b PE=1 SV=2 |
| 83 | 399 | sp\|P02301\|H3C_MOUSE | 49.84 | 15 | 15 | 2.32E+06 | 3 | 3 | 3 |  | 15315 | Histone H3.3C OS=Mus musculus OX=10090 GN=H3f3c PE=3 SV=3 |
| 83 | 403 | A0A1W2P768\|A0A1W2P768_MOUSE | 49.84 | 11 | 11 | 2.32E+06 | 3 | 3 | 3 |  | 20247 | H3 clustered histone 14 OS=Mus musculus OX=10090 GN=H3c14 PE=1 SV=1 |
| 73 | 443 | A0A0J9YKD4\|A0A0J9YKD4_MOUSE | 49.65 | 6 | 6 | 4.20E+06 | 3 | 3 | 4 | Carbamidomethylation | 35108 | Creatine kinase M-type OS=Mus musculus OX=10090 GN=Ckm PE=1 SV=1 |
| 73 | 444 | sp\|P07310\|KCRM_MOUSE | 49.65 | 5 | 5 | 4.20E+06 | 3 | 3 | 4 | Carbamidomethylation | 43045 | Creatine kinase M-type OS=Mus musculus OX=10090 GN=Ckm PE=1 SV=1 |
| 73 | 445 | A2RTA0\|A2RTA0_MOUSE | 49.65 | 5 | 5 | 4.20E+06 | 3 | 3 | 4 | Carbamidomethylation | 43045 | Creatine kinase muscle OS=Mus musculus OX=10090 GN=Ckm PE=1 SV=1 |
| 73 | 446 | Q9D6U7\|Q9D6U7_MOUSE | 49.65 | 5 | 5 | 4.20E+06 | 3 | 3 | 4 | Carbamidomethylation | 43031 | Uncharacterized protein OS=Mus musculus OX=10090 GN=Ckm PE=2 SV=1 |
| 102 | 531 | A0A0G2JG95\|A0A0G2JG95_MOUSE | 49 | 11 | 11 | 3.05E+06 | 2 | 2 | 2 |  | 20294 | Serine/threonine-protein phosphatase PGAM5 mitochondrial (Fragment) OS=Mus musculus OX=10090 GN=Pgam5 PE=1 SV=1 |
| 102 | 533 | B7ZNW0\|B7ZNW0_MOUSE | 49 | 7 | 7 | 3.05E+06 | 2 | 2 | 2 |  | 32022 | Pgam5 protein OS=Mus musculus OX=10090 GN=Pgam5 PE=2 SV=1 |
| 102 | 532 | sp\|Q8BX10\|PGAM5_MOUSE | 49 | 7 | 7 | 3.05E+06 | 2 | 2 | 2 |  | 31994 | Serine/threonine-protein phosphatase PGAM5 mitochondrial OS=Mus musculus OX=10090 GN=Pgam5 PE=1 SV=1 |
| 102 | 534 | A1A4A7\|A1A4A7_MOUSE | 49 | 7 | 7 | 3.05E+06 | 2 | 2 | 2 |  | 32286 | Pgam5 protein (Fragment) OS=Mus musculus OX=10090 GN=Pgam5 PE=2 SV=1 |
| 97 | 6030 | Q3TKR5\|Q3TKR5_MOUSE | 48.48 | 4 | 4 | 3.58E+06 | 2 | 2 | 2 |  | 34269 | Ribosomal protein L5 OS=Mus musculus OX=10090 GN=Rpl5 PE=2 SV=1 |
| 75 | 365 | A0A4U9FFL2\|A0A4U9FFL2_MOUSE | 48.03 | 13 | 13 | 2.90E+05 | 2 | 1 | 2 | Carbamidomethylation; Deamidation (NQ); Oxidation (M) | 35634 | IgG1 (Fragment) OS=Mus musculus OX=10090 GN=Ighg1 PE=4 SV=1 |
| 75 | 366 | A0A075B5P4\|A0A075B5P4_MOUSE | 48.03 | 13 | 13 | 2.90E+05 | 2 | 1 | 2 | Carbamidomethylation; Deamidation (NQ); Oxidation (M) | 35752 | Ig gamma-1 chain C region secreted form (Fragment) OS=Mus musculus OX=10090 GN=Ighg1 PE=1 SV=1 |
| 75 | 367 | A0A0A6YWR2\|A0A0A6YWR2_MOUSE | 48.03 | 11 | 11 | 2.90E+05 | 2 | 1 | 2 | Carbamidomethylation; Deamidation (NQ); Oxidation (M) | 43434 | Ig gamma-1 chain C region secreted form (Fragment) OS=Mus musculus OX=10090 GN=Ighg1 PE=1 SV=1 |
| 75 | 356 | A0A0B6VMB2\|A0A0B6VMB2_MOUSE | 48.03 | 9 | 9 | 2.90E+05 | 2 | 1 | 2 | Carbamidomethylation; Deamidation (NQ); Oxidation (M) | 50725 | MAb 31C6 heavy chain OS=Mus musculus OX=10090 GN=HC PE=4 SV=1 |
| 75 | 368 | A0A0C6E3V3\|A0A0C6E3V3_MOUSE | 48.03 | 9 | 9 | 2.90E+05 | 2 | 1 | 2 | Carbamidomethylation; Deamidation (NQ); Oxidation (M) | 51022 | HC protein OS=Mus musculus OX=10090 GN=HC PE=2 SV=1 |
| 130 | 613 | Q3UJS0\|Q3UJS0_MOUSE | 47.6 | 4 | 4 | 2.06E+06 | 1 | 1 | 1 |  | 28067 | Ribosomal_L2_C domain-containing protein OS=Mus musculus OX=10090 GN=Rpl8 PE=2 SV=1 |
| 130 | 614 | sp\|P62918\|RL8_MOUSE | 47.6 | 4 | 4 | 2.06E+06 | 1 | 1 | 1 |  | 28025 | 60S ribosomal protein L8 OS=Mus musculus OX=10090 GN=Rpl8 PE=1 SV=2 |
| 108 | 502 | A0A0A6YXL3\|A0A0A6YXL3_MOUSE | 47.47 | 10 | 10 | 2.34E+06 | 1 | 1 | 1 |  | 9942 | 60S ribosomal protein L31 OS=Mus musculus OX=10090 GN=Rpl31 PE=1 SV=1 |
| 108 | 449 | sp\|P62900\|RL31_MOUSE | 47.47 | 7 | 7 | 2.34E+06 | 1 | 1 | 1 |  | 14463 | 60S ribosomal protein L31 OS=Mus musculus OX=10090 GN=Rpl31 PE=1 SV=1 |
| 108 | 448 | Q5M9K9\|Q5M9K9_MOUSE | 47.47 | 7 | 7 | 2.34E+06 | 1 | 1 | 1 |  | 14463 | MCG126194 isoform CRA_a OS=Mus musculus OX=10090 GN=Rpl31 PE=1 SV=1 |
| 108 | 450 | Q9CY93\|Q9CY93_MOUSE | 47.47 | 7 | 7 | 2.34E+06 | 1 | 1 | 1 |  | 14411 | Uncharacterized protein OS=Mus musculus OX=10090 GN=Rpl31 PE=2 SV=1 |
| 108 | 503 | A0A0A6YX26\|A0A0A6YX26_MOUSE | 47.47 | 7 | 7 | 2.34E+06 | 1 | 1 | 1 |  | 14997 | 60S ribosomal protein L31 OS=Mus musculus OX=10090 GN=Rpl31 PE=1 SV=1 |
| 104 | 540 | Q50HX1\|Q50HX1_MOUSE | 47.46 | 9 | 9 | 9.49E+05 | 2 | 2 | 2 |  | 21817 | RAB14 protein variant OS=Mus musculus OX=10090 GN=Rab14 PE=2 SV=1 |
| 104 | 539 | Q50HX3\|Q50HX3_MOUSE | 47.46 | 9 | 9 | 9.49E+05 | 2 | 2 | 2 |  | 21889 | RAB14 protein variant OS=Mus musculus OX=10090 GN=Rab14 PE=1 SV=1 |
| 104 | 541 | Q50HX2\|Q50HX2_MOUSE | 47.46 | 8 | 8 | 9.49E+05 | 2 | 2 | 2 |  | 23825 | RAB14 protein OS=Mus musculus OX=10090 GN=Rab14 PE=2 SV=1 |
| 104 | 543 | sp\|Q91V41\|RAB14_MOUSE | 47.46 | 8 | 8 | 9.49E+05 | 2 | 2 | 2 |  | 23897 | Ras-related protein Rab-14 OS=Mus musculus OX=10090 GN=Rab14 PE=1 SV=3 |
| 104 | 542 | Q50HX4\|Q50HX4_MOUSE | 47.46 | 8 | 8 | 9.49E+05 | 2 | 2 | 2 |  | 23897 | RAB14 protein OS=Mus musculus OX=10090 GN=Rab14 PE=1 SV=1 |
| 152 | 688 | E0CZA1\|E0CZA1_MOUSE | 47.37 | 5 | 5 | 8.81E+05 | 1 | 1 | 1 |  | 21526 | T-complex protein 1 subunit epsilon (Fragment) OS=Mus musculus OX=10090 GN=Cct5 PE=1 SV=1 |
| 152 | 695 | sp\|P80316\|TCPE_MOUSE | 47.37 | 2 | 2 | 8.81E+05 | 1 | 1 | 1 |  | 59624 | T-complex protein 1 subunit epsilon OS=Mus musculus OX=10090 GN=Cct5 PE=1 SV=1 |
| 79 | 385 | G3UX26\|G3UX26_MOUSE | 46.99 | 3 | 3 | 1.12E+06 | 1 | 1 | 1 |  | 30446 | Voltage-dependent anion-selective channel protein 2 OS=Mus musculus OX=10090 GN=Vdac2 PE=1 SV=1 |
| 79 | 386 | sp\|Q60930\|VDAC2_MOUSE | 46.99 | 3 | 3 | 1.12E+06 | 1 | 1 | 1 |  | 31733 | Voltage-dependent anion-selective channel protein 2 OS=Mus musculus OX=10090 GN=Vdac2 PE=1 SV=2 |
| 79 | 411 | A0A286YCR8\|A0A286YCR8_MOUSE | 46.99 | 3 | 3 | 1.12E+06 | 1 | 1 | 1 |  | 26741 | Voltage-dependent anion-selective channel protein 2 (Fragment) OS=Mus musculus OX=10090 GN=Vdac2 PE=1 SV=1 |
| 153 | 689 | Q6NWV5\|Q6NWV5_MOUSE | 46.81 | 5 | 5 | 1.64E+06 | 1 | 1 | 1 | PGAM1 | 22415 | Pgam1 protein (Fragment) OS=Mus musculus OX=10090 GN=Pgam1 PE=2 SV=1 |
| 153 | 691 | Q5NCI4\|Q5NCI4_MOUSE | 46.81 | 4 | 4 | 1.64E+06 | 1 | 1 | 1 |  | 28827 | Phosphoglycerate mutase OS=Mus musculus OX=10090 GN=Pgam2 PE=1 SV=1 |
| 153 | 692 | sp\|O70250\|PGAM2_MOUSE | 46.81 | 4 | 4 | 1.64E+06 | 1 | 1 | 1 |  | 28827 | Phosphoglycerate mutase 2 OS=Mus musculus OX=10090 GN=Pgam2 PE=1 SV=3 |
| 153 | 694 | Q3U7Z6\|Q3U7Z6_MOUSE | 46.81 | 4 | 4 | 1.64E+06 | 1 | 1 | 1 |  | 28832 | Phosphoglycerate mutase OS=Mus musculus OX=10090 GN=Pgam1 PE=1 SV=1 |
| 153 | 693 | sp\|Q9DBJ1\|PGAM1_MOUSE | 46.81 | 4 | 4 | 1.64E+06 | 1 | 1 | 1 |  | 28832 | Phosphoglycerate mutase 1 OS=Mus musculus OX=10090 GN=Pgam1 PE=1 SV=3 |
| 112 | 624 | sp\|Q9CR68\|UCRI_MOUSE | 46.77 | 3 | 3 | 1.20E+06 | 2 | 2 | 2 |  | 29368 | Cytochrome b-c1 complex subunit Rieske mitochondrial OS=Mus musculus OX=10090 GN=Uqcrfs1 PE=1 SV=1 |
| 87 | 505 | D3YXF4\|D3YXF4_MOUSE | 46.43 | 48 | 48 | 5.99E+05 | 2 | 1 | 3 |  | 5002 | 14-3-3 protein zeta/delta (Fragment) OS=Mus musculus OX=10090 GN=Ywhaz PE=1 SV=8 |
| 87 | 325 | D3YXN6\|D3YXN6_MOUSE | 46.43 | 24 | 24 | 5.99E+05 | 2 | 1 | 3 |  | 10098 | 14-3-3 protein zeta/delta (Fragment) OS=Mus musculus OX=10090 GN=Ywhaz PE=1 SV=1 |
| 87 | 326 | Q8BWN0\|Q8BWN0_MOUSE | 46.43 | 18 | 18 | 5.99E+05 | 2 | 1 | 3 |  | 13336 | 14_3_3 domain-containing protein OS=Mus musculus OX=10090 PE=2 SV=1 |
| 87 | 327 | A0A2I3BQ03\|A0A2I3BQ03_MOUSE | 46.43 | 13 | 13 | 5.99E+05 | 2 | 1 | 3 |  | 19065 | 14-3-3 protein zeta/delta (Fragment) OS=Mus musculus OX=10090 GN=Ywhaz PE=1 SV=1 |
| 87 | 334 | sp\|P63101\|1433Z_MOUSE | 46.43 | 9 | 9 | 5.99E+05 | 2 | 1 | 3 |  | 27771 | 14-3-3 protein zeta/delta OS=Mus musculus OX=10090 GN=Ywhaz PE=1 SV=1 |
| 107 | 467 | S4R1N6\|S4R1N6_MOUSE | 45.81 | 10 | 10 | 1.45E+06 | 1 | 1 | 1 |  | 12483 | 40S ribosomal protein S18 OS=Mus musculus OX=10090 GN=Rps18 PE=3 SV=1 |
| 107 | 469 | Q3TW65\|Q3TW65_MOUSE | 45.81 | 7 | 7 | 1.45E+06 | 1 | 1 | 1 |  | 17747 | Uncharacterized protein OS=Mus musculus OX=10090 GN=Rps18 PE=2 SV=1 |
| 107 | 470 | Q561N5\|Q561N5_MOUSE | 45.81 | 7 | 7 | 1.45E+06 | 1 | 1 | 1 |  | 17719 | MCG23000 isoform CRA_b OS=Mus musculus OX=10090 GN=Rps18 PE=2 SV=1 |
| 107 | 471 | sp\|P62270\|RS18_MOUSE | 45.81 | 7 | 7 | 1.45E+06 | 1 | 1 | 1 |  | 17719 | 40S ribosomal protein S18 OS=Mus musculus OX=10090 GN=Rps18 PE=1 SV=3 |
| 107 | 472 | F6YVP7\|F6YVP7_MOUSE | 45.81 | 7 | 7 | 1.45E+06 | 1 | 1 | 1 |  | 17672 | Predicted gene 10260 OS=Mus musculus OX=10090 GN=Gm10260 PE=3 SV=2 |
| 107 | 468 | A0A1Y7VKY1\|A0A1Y7VKY1_MOUSE | 45.81 | 7 | 7 | 1.45E+06 | 1 | 1 | 1 |  | 17749 | MCG116671 OS=Mus musculus OX=10090 GN=Rps18-ps5 PE=3 SV=1 |
| 154 | 583 | sp\|P68040\|RACK1_MOUSE | 45.4 | 3 | 3 | 5.23E+05 | 1 | 1 | 1 | RACK1 | 35077 | Receptor of activated protein C kinase 1 OS=Mus musculus OX=10090 GN=Rack1 PE=1 SV=3 |
| 88 | 525 | A0A3B2WDD2\|A0A3B2WDD2_MOUSE | 44.46 | 8 | 8 | 3.10E+06 | 2 | 2 | 3 |  | 21637 | Ribosomal protein OS=Mus musculus OX=10090 GN=Rpl10a PE=1 SV=1 |
| 88 | 526 | A0A3B2WBL1\|A0A3B2WBL1_MOUSE | 44.46 | 7 | 7 | 3.10E+06 | 2 | 2 | 3 |  | 24744 | Ribosomal protein OS=Mus musculus OX=10090 GN=Rpl10a PE=1 SV=1 |
| 88 | 527 | sp\|P53026\|RL10A_MOUSE | 44.46 | 7 | 7 | 3.10E+06 | 2 | 2 | 3 |  | 24916 | 60S ribosomal protein L10a OS=Mus musculus OX=10090 GN=Rpl10a PE=1 SV=3 |
| 88 | 529 | Q5XJF6\|Q5XJF6_MOUSE | 44.46 | 7 | 7 | 3.10E+06 | 2 | 2 | 3 |  | 24831 | Ribosomal protein OS=Mus musculus OX=10090 GN=Rpl10a PE=1 SV=1 |
| 88 | 528 | Q3U561\|Q3U561_MOUSE | 44.46 | 7 | 7 | 3.10E+06 | 2 | 2 | 3 |  | 24815 | Ribosomal protein OS=Mus musculus OX=10090 GN=Rpl10a PE=2 SV=1 |
| 95 | 556 | Q3TQL4\|Q3TQL4_MOUSE | 44.39 | 2 | 2 | 1.73E+06 | 2 | 2 | 2 |  | 75473 | MICOS complex subunit MIC60 (Fragment) OS=Mus musculus OX=10090 GN=Immt PE=2 SV=1 |
| 95 | 568 | Q3U7N2\|Q3U7N2_MOUSE | 44.39 | 2 | 2 | 1.73E+06 | 2 | 2 | 2 |  | 82513 | MICOS complex subunit MIC60 OS=Mus musculus OX=10090 GN=Immt PE=2 SV=1 |
| 95 | 569 | Q3TVZ5\|Q3TVZ5_MOUSE | 44.39 | 2 | 2 | 1.73E+06 | 2 | 2 | 2 |  | 82416 | MICOS complex subunit MIC60 OS=Mus musculus OX=10090 GN=Immt PE=2 SV=1 |
| 95 | 571 | sp\|Q8CAQ8\|MIC60_MOUSE | 44.39 | 2 | 2 | 1.73E+06 | 2 | 2 | 2 |  | 83900 | MICOS complex subunit Mic60 OS=Mus musculus OX=10090 GN=Immt PE=1 SV=1 |
| 95 | 545 | A6H604\|A6H604_MOUSE | 44.39 | 6 | 6 | 1.73E+06 | 2 | 2 | 2 |  | 30454 | MICOS complex subunit MIC60 (Fragment) OS=Mus musculus OX=10090 GN=Immt PE=2 SV=1 |
| 95 | 546 | A0A0U1RQ14\|A0A0U1RQ14_MOUSE | 44.39 | 5 | 5 | 1.73E+06 | 2 | 2 | 2 |  | 33001 | MICOS complex subunit MIC60 (Fragment) OS=Mus musculus OX=10090 GN=Immt PE=1 SV=1 |
| 95 | 547 | Q6P8Y5\|Q6P8Y5_MOUSE | 44.39 | 5 | 5 | 1.73E+06 | 2 | 2 | 2 |  | 34103 | MICOS complex subunit MIC60 (Fragment) OS=Mus musculus OX=10090 GN=Immt PE=2 SV=1 |
| 95 | 551 | Q2YDW0\|Q2YDW0_MOUSE | 44.39 | 3 | 3 | 1.73E+06 | 2 | 2 | 2 |  | 50156 | MICOS complex subunit MIC60 (Fragment) OS=Mus musculus OX=10090 GN=Immt PE=2 SV=1 |
| 95 | 557 | Q3TEY5\|Q3TEY5_MOUSE | 44.39 | 2 | 2 | 1.73E+06 | 2 | 2 | 2 |  | 75629 | MICOS complex subunit MIC60 OS=Mus musculus OX=10090 GN=Immt PE=2 SV=1 |
| 95 | 558 | E9Q800\|E9Q800_MOUSE | 44.39 | 2 | 2 | 1.73E+06 | 2 | 2 | 2 |  | 75601 | MICOS complex subunit MIC60 OS=Mus musculus OX=10090 GN=Immt PE=1 SV=1 |
| 155 | 745 | G3UY29\|G3UY29_MOUSE | 44.38 | 7 | 7 | 1.11E+06 | 1 | 1 | 1 |  | 16924 | MCG22989 isoform CRA_a OS=Mus musculus OX=10090 GN=Rab11b PE=4 SV=1 |
| 155 | 746 | E9Q3P9\|E9Q3P9_MOUSE | 44.38 | 7 | 7 | 1.11E+06 | 1 | 1 | 1 |  | 17375 | Ras-related protein Rab-11A OS=Mus musculus OX=10090 GN=Rab11a PE=4 SV=1 |
| 155 | 747 | F8WGS1\|F8WGS1_MOUSE | 44.38 | 7 | 7 | 1.11E+06 | 1 | 1 | 1 |  | 17668 | Ras-related protein Rab-11A (Fragment) OS=Mus musculus OX=10090 GN=Rab11a PE=4 SV=1 |
| 155 | 748 | sp\|P62492\|RB11A_MOUSE | 44.38 | 5 | 5 | 1.11E+06 | 1 | 1 | 1 |  | 24394 | Ras-related protein Rab-11A OS=Mus musculus OX=10090 GN=Rab11a PE=1 SV=3 |
| 155 | 749 | Q0PD45\|Q0PD45_MOUSE | 44.38 | 5 | 5 | 1.11E+06 | 1 | 1 | 1 |  | 24394 | RAB11a member RAS oncogene family OS=Mus musculus OX=10090 GN=Rab11a PE=1 SV=1 |
| 155 | 750 | A0A068BFR3\|A0A068BFR3_MOUSE | 44.38 | 5 | 5 | 1.11E+06 | 1 | 1 | 1 |  | 24488 | RAS oncogene family protein OS=Mus musculus OX=10090 GN=Rab11b PE=2 SV=1 |
| 155 | 751 | Q78ZJ8\|Q78ZJ8_MOUSE | 44.38 | 5 | 5 | 1.11E+06 | 1 | 1 | 1 |  | 24489 | MCG22989 isoform CRA_b OS=Mus musculus OX=10090 GN=Rab11b PE=1 SV=1 |
| 155 | 752 | sp\|P46638\|RB11B_MOUSE | 44.38 | 5 | 5 | 1.11E+06 | 1 | 1 | 1 |  | 24489 | Ras-related protein Rab-11B OS=Mus musculus OX=10090 GN=Rab11b PE=1 SV=3 |
| 96 | 464 | Q91YK6\|Q91YK6_MOUSE | 44.04 | 14 | 14 | 1.35E+06 | 2 | 1 | 2 |  | 16940 | Recurrent De Novo Mutations Disturbing the GTP/GDP Binding Pocket of RAB11B Cause Intellectual Disability and a Distinctive Brain Phenotype.protein (Fragment) OS=Mus musculus OX=10090 GN=Rpl23a PE=2 SV=1 |
| 96 | 465 | Q4V9X9\|Q4V9X9_MOUSE | 44.04 | 14 | 14 | 1.35E+06 | 2 | 1 | 2 |  | 17564 | Rpl23a protein (Fragment) OS=Mus musculus OX=10090 GN=Rpl23a PE=2 SV=1 |
| 96 | 447 | sp\|P62751\|RL23A_MOUSE | 44.04 | 13 | 13 | 1.35E+06 | 2 | 1 | 2 |  | 17695 | 60S ribosomal protein L23a OS=Mus musculus OX=10090 GN=Rpl23a PE=1 SV=1 |
| 96 | 466 | Q5M9M5\|Q5M9M5_MOUSE | 44.04 | 13 | 13 | 1.35E+06 | 2 | 1 | 2 |  | 17695 | MCG10806 OS=Mus musculus OX=10090 GN=Rpl23a PE=1 SV=1 |
| 109 | 544 | sp\|Q9WUM5\|SUCA_MOUSE | 43.33 | 3 | 3 | 5.41E+05 | 1 | 1 | 1 |  | 36155 | Succinate--CoA ligase [ADP/GDP-forming] subunit alpha mitochondrial OS=Mus musculus OX=10090 GN=Suclg1 PE=1 SV=4 |
| 156 | 493 | Q3ULW0\|Q3ULW0_MOUSE | 43.18 | 5 | 5 | 8.24E+05 | 1 | 1 | 1 | Ran | 24351 | GTP-binding nuclear protein Ran OS=Mus musculus OX=10090 GN=Ran PE=2 SV=1 |
| 156 | 494 | sp\|P62827\|RAN_MOUSE | 43.18 | 5 | 5 | 8.24E+05 | 1 | 1 | 1 |  | 24423 | GTP-binding nuclear protein Ran OS=Mus musculus OX=10090 GN=Ran PE=1 SV=3 |
| 156 | 709 | Q14AA6\|Q14AA6_MOUSE | 43.18 | 5 | 5 | 8.24E+05 | 1 | 1 | 1 |  | 24357 | GTP-binding nuclear protein Ran OS=Mus musculus OX=10090 GN=1700009N14Rik PE=2 SV=1 |
| 110 | 796 | sp\|P27661\|H2AX_MOUSE | 42.69 | 6 | 6 | 4.00E+06 | 1 | 1 | 1 |  | 15143 | Histone H2AX OS=Mus musculus OX=10090 GN=H2afx PE=1 SV=2 |
| 157 | 755 | B9EHN0\|B9EHN0_MOUSE | 40.32 | 1 | 1 | 5.76E+05 | 1 | 1 | 1 |  | 117809 | Ubiquitin-activating enzyme E1 Chr X OS=Mus musculus OX=10090 GN=Uba1 PE=1 SV=1 |
| 157 | 756 | sp\|Q02053\|UBA1_MOUSE | 40.32 | 1 | 1 | 5.76E+05 | 1 | 1 | 1 | UBA1 | 117809 | Ubiquitin-like modifier-activating enzyme 1 OS=Mus musculus OX=10090 GN=Uba1 PE=1 SV=1 |
| 157 | 757 | A0A1S6GWH5\|A0A1S6GWH5_MOUSE | 40.32 | 1 | 1 | 5.76E+05 | 1 | 1 | 1 |  | 124236 | UBA_e1_C domain-containing protein OS=Mus musculus OX=10090 GN=Uba1 PE=2 SV=1 |
| 111 | 754 | A0A0N4SVQ1\|A0A0N4SVQ1_MOUSE | 39.71 | 16 | 16 | 2.03E+06 | 1 | 1 | 2 | NDUFA4 | 5877 | Cytochrome c oxidase subunit NDUFA4 OS=Mus musculus OX=10090 GN=Ndufa4 PE=1 SV=1 |
| 111 | 504 | sp\|Q62425\|NDUA4_MOUSE | 39.71 | 10 | 10 | 2.03E+06 | 1 | 1 | 2 |  | 9327 | Cytochrome c oxidase subunit NDUFA4 OS=Mus musculus OX=10090 GN=Ndufa4 PE=1 SV=2 |
| 119 | 474 | sp\|Q9Z2Z6\|MCAT_MOUSE | 39.24 | 3 | 3 | 6.18E+04 | 1 | 1 | 1 |  | 33027 | Mitochondrial carnitine/acylcarnitine carrier protein OS=Mus musculus OX=10090 GN=Slc25a20 PE=1 SV=1 |
| 119 | 475 | Q7TPW6\|Q7TPW6_MOUSE | 39.24 | 3 | 3 | 6.18E+04 | 1 | 1 | 1 |  | 32966 | Solute carrier family 25 (Mitochondrial carnitine/acylcarnitine translocase) member 20 OS=Mus musculus OX=10090 GN=Slc25a20 PE=2 SV=1 |
| 119 | 759 | sp\|Q8BW66\|S2548_MOUSE | 39.24 | 3 | 3 | 6.18E+04 | 1 | 1 | 1 |  | 33387 | Solute carrier family 25 member 48 OS=Mus musculus OX=10090 GN=Slc25a48 PE=1 SV=2 |
| 158 | 740 | sp\|P14152\|MDHC_MOUSE | 39.08 | 3 | 3 | 9.61E+05 | 1 | 1 | 1 |  | 36511 | Malate dehydrogenase cytoplasmic OS=Mus musculus OX=10090 GN=Mdh1 PE=1 SV=3 |
| 44 | 731 | A0A075B5U5\|A0A075B5U5_MOUSE | 39.04 | 21 | 21 | 2.68E+07 | 1 | 1 | 7 | Carbamidomethylation | 12857 | Immunoglobulin heavy variable V1-19 OS=Mus musculus OX=10090 GN=Ighv1-19 PE=1 SV=1 |
| 44 | 730 | A0A075B5V3\|A0A075B5V3_MOUSE | 39.04 | 21 | 21 | 2.68E+07 | 1 | 1 | 7 | Carbamidomethylation | 12904 | Immunoglobulin heavy variable 1-36 OS=Mus musculus OX=10090 GN=Ighv1-36 PE=4 SV=1 |
| 44 | 732 | A0A0A6YXT2\|A0A0A6YXT2_MOUSE | 39.04 | 21 | 21 | 2.68E+07 | 1 | 1 | 7 | Carbamidomethylation | 12961 | Immunoglobulin heavy variable 1-36 (Fragment) OS=Mus musculus OX=10090 GN=Ighv1-36 PE=4 SV=1 |
| 44 | 733 | A0A0A6YWX0\|A0A0A6YWX0_MOUSE | 39.04 | 21 | 21 | 2.68E+07 | 1 | 1 | 7 | Carbamidomethylation | 12914 | Immunoglobulin heavy variable V1-19 (Fragment) OS=Mus musculus OX=10090 GN=Ighv1-19 PE=1 SV=1 |
| 160 | 741 | B1ARA3\|B1ARA3_MOUSE | 37.79 | 8 | 8 | 5.00E+05 | 1 | 1 | 1 |  | 12217 | 60S ribosomal protein L26 (Fragment) OS=Mus musculus OX=10090 GN=Rpl26 PE=1 SV=1 |
| 160 | 742 | Q3U7N1\|Q3U7N1_MOUSE | 37.79 | 6 | 6 | 5.00E+05 | 1 | 1 | 1 |  | 17259 | KOW domain-containing protein OS=Mus musculus OX=10090 PE=2 SV=1 |
| 160 | 743 | Q4FZH2\|Q4FZH2_MOUSE | 37.79 | 6 | 6 | 5.00E+05 | 1 | 1 | 1 |  | 17258 | MCG50660 isoform CRA_a OS=Mus musculus OX=10090 GN=Rpl26 PE=1 SV=1 |
| 160 | 744 | sp\|P61255\|RL26_MOUSE | 37.79 | 6 | 6 | 5.00E+05 | 1 | 1 | 1 |  | 17258 | 60S ribosomal protein L26 OS=Mus musculus OX=10090 GN=Rpl26 PE=1 SV=1 |
| 128 | 606 | F7C106\|F7C106_MOUSE | 37.53 | 9 | 9 | 1.28E+06 | 1 | 1 | 1 |  | 9014 | Cytochrome c oxidase subunit 5B mitochondrial OS=Mus musculus OX=10090 GN=Cox5b PE=1 SV=2 |
| 128 | 607 | A0A0A6YVR0\|A0A0A6YVR0_MOUSE | 37.53 | 8 | 8 | 1.28E+06 | 1 | 1 | 1 |  | 10336 | Cytochrome c oxidase subunit 5B mitochondrial (Fragment) OS=Mus musculus OX=10090 GN=Cox5b PE=1 SV=1 |
| 128 | 608 | sp\|P19536\|COX5B_MOUSE | 37.53 | 6 | 6 | 1.28E+06 | 1 | 1 | 1 |  | 13813 | Cytochrome c oxidase subunit 5B mitochondrial OS=Mus musculus OX=10090 GN=Cox5b PE=1 SV=1 |
| 128 | 609 | Q9D881\|Q9D881_MOUSE | 37.53 | 6 | 6 | 1.28E+06 | 1 | 1 | 1 |  | 13847 | Cytochrome c oxidase subunit 5B mitochondrial OS=Mus musculus OX=10090 GN=Gm11273 PE=1 SV=1 |
| 159 | 578 | Q4VAG4\|Q4VAG4_MOUSE | 36.96 | 10 | 10 | 1.38E+06 | 1 | 1 | 1 |  | 14759 | MCG12304 OS=Mus musculus OX=10090 GN=Rpl22 PE=1 SV=1 |
| 159 | 579 | sp\|P67984\|RL22_MOUSE | 36.96 | 10 | 10 | 1.38E+06 | 1 | 1 | 1 |  | 14759 | 60S ribosomal protein L22 OS=Mus musculus OX=10090 GN=Rpl22 PE=1 SV=2 |
| 129 | 801 | A2AUR7\|A2AUR7_MOUSE | 35.73 | 4 | 4 | 7.44E+05 | 1 | 1 | 1 | RSU1 | 29543 | Ras suppressor protein 1 OS=Mus musculus OX=10090 GN=Rsu1 PE=1 SV=1 |
| 129 | 802 | Q9D031\|Q9D031_MOUSE | 35.73 | 4 | 4 | 7.44E+05 | 1 | 1 | 1 |  | 31461 | Ras suppressor protein 1 OS=Mus musculus OX=10090 GN=Rsu1 PE=1 SV=1 |
| 129 | 803 | sp\|Q01730\|RSU1_MOUSE | 35.73 | 4 | 4 | 7.44E+05 | 1 | 1 | 1 |  | 31550 | Ras suppressor protein 1 OS=Mus musculus OX=10090 GN=Rsu1 PE=1 SV=3 |
| 129 | 797 | E0CXG5\|E0CXG5_MOUSE | 35.73 | 18 | 18 | 7.44E+05 | 1 | 1 | 1 |  | 6267 | Ras suppressor protein 1 OS=Mus musculus OX=10090 GN=Rsu1 PE=1 SV=1 |
| 129 | 798 | A0A0A6YWZ2\|A0A0A6YWZ2_MOUSE | 35.73 | 16 | 16 | 7.44E+05 | 1 | 1 | 1 |  | 7167 | Ras suppressor protein 1 OS=Mus musculus OX=10090 GN=Rsu1 PE=1 SV=1 |
| 129 | 799 | B1AYQ0\|B1AYQ0_MOUSE | 35.73 | 9 | 9 | 7.44E+05 | 1 | 1 | 1 |  | 12635 | Ras suppressor protein 1 (Fragment) OS=Mus musculus OX=10090 GN=Rsu1 PE=1 SV=1 |
| 129 | 800 | Q3TFN7\|Q3TFN7_MOUSE | 35.73 | 4 | 4 | 7.44E+05 | 1 | 1 | 1 |  | 27608 | Uncharacterized protein OS=Mus musculus OX=10090 GN=Rsu1 PE=2 SV=1 |
| 161 | 821 | Q9DCY1\|Q9DCY1_MOUSE | 34.61 | 4 | 4 | 1.23E+06 | 1 | 1 | 1 |  | 23713 | Peptidyl-prolyl cis-trans isomerase OS=Mus musculus OX=10090 GN=Ppib PE=1 SV=1 |
| 161 | 820 | sp\|P24369\|PPIB_MOUSE | 34.61 | 4 | 4 | 1.23E+06 | 1 | 1 | 1 |  | 23713 | Peptidyl-prolyl cis-trans isomerase B OS=Mus musculus OX=10090 GN=Ppib PE=1 SV=2 |
| 163 | 822 | sp\|Q93092\|TALDO_MOUSE | 34.16 | 2 | 2 | 7.21E+05 | 1 | 1 | 1 |  | 37387 | Transaldolase OS=Mus musculus OX=10090 GN=Taldo1 PE=1 SV=2 |
| 163 | 823 | A0A1B0GR11\|A0A1B0GR11_MOUSE | 34.16 | 2 | 2 | 7.21E+05 | 1 | 1 | 1 |  | 42151 | Transaldolase OS=Mus musculus OX=10090 GN=Taldo1 PE=1 SV=1 |
| 162 | 849 | Q4VA32\|Q4VA32_MOUSE | 33.95 | 7 | 7 | 4.63E+05 | 1 | 1 | 1 |  | 15183 | Thioesterase superfamily member 2 OS=Mus musculus OX=10090 GN=Acot13 PE=1 SV=1 |
| 162 | 848 | sp\|Q9CQR4\|ACO13_MOUSE | 33.95 | 7 | 7 | 4.63E+05 | 1 | 1 | 1 |  | 15183 | Acyl-coenzyme A thioesterase 13 OS=Mus musculus OX=10090 GN=Acot13 PE=1 SV=1 |
| 132 | 710 | sp\|P06329\|HVM50_MOUSE | 33.79 | 21 | 21 | 2.87E+06 | 1 | 1 | 1 | Carbamidomethylation; Oxidation (M) | 13311 | Ig heavy chain V region AC38 15.3 OS=Mus musculus OX=10090 PE=1 SV=1 |
| 166 | 593 | E9PZ67\|E9PZ67_MOUSE | 33.03 | 2 | 2 | 8.64E+05 | 1 | 1 | 1 |  | 36424 | Calsequestrin (Fragment) OS=Mus musculus OX=10090 GN=Casq2 PE=1 SV=1 |
| 166 | 594 | sp\|O09161\|CASQ2_MOUSE | 33.03 | 2 | 2 | 8.64E+05 | 1 | 1 | 1 |  | 48176 | Calsequestrin-2 OS=Mus musculus OX=10090 GN=Casq2 PE=1 SV=3 |
| 166 | 595 | F6QYE1\|F6QYE1_MOUSE | 33.03 | 2 | 2 | 8.64E+05 | 1 | 1 | 1 |  | 48436 | Calsequestrin OS=Mus musculus OX=10090 GN=Casq2 PE=1 SV=1 |
| 166 | 596 | B9EHC7\|B9EHC7_MOUSE | 33.03 | 2 | 2 | 8.64E+05 | 1 | 1 | 1 |  | 50086 | Calsequestrin OS=Mus musculus OX=10090 GN=Casq2 PE=2 SV=1 |
| 164 | 815 | Q0QEZ4\|Q0QEZ4_MOUSE | 32.64 | 4 | 4 | 4.59E+05 | 1 | 1 | 1 |  | 27208 | Succinate dehydrogenase [ubiquinone] iron-sulfur subunit mitochondrial (Fragment) OS=Mus musculus OX=10090 GN=Sdhb PE=2 SV=1 |
| 164 | 816 | Q3TE45\|Q3TE45_MOUSE | 32.64 | 4 | 4 | 4.59E+05 | 1 | 1 | 1 |  | 31818 | Succinate dehydrogenase [ubiquinone] iron-sulfur subunit mitochondrial OS=Mus musculus OX=10090 GN=Sdhb PE=2 SV=1 |
| 164 | 817 | sp\|Q9CQA3\|SDHB_MOUSE | 32.64 | 4 | 4 | 4.59E+05 | 1 | 1 | 1 |  | 31814 | Succinate dehydrogenase [ubiquinone] iron-sulfur subunit mitochondrial OS=Mus musculus OX=10090 GN=Sdhb PE=1 SV=1 |
| 167 | 828 | A0A087WS46\|A0A087WS46_MOUSE | 32.02 | 4 | 4 | 8.14E+05 | 1 | 1 | 1 |  | 20137 | Eukaryotic translation elongation factor 1 beta 2 OS=Mus musculus OX=10090 GN=Eef1b2 PE=1 SV=1 |
| 167 | 837 | sp\|O70251\|EF1B_MOUSE | 32.02 | 3 | 3 | 8.14E+05 | 1 | 1 | 1 |  | 24694 | Elongation factor 1-beta OS=Mus musculus OX=10090 GN=Eef1b PE=1 SV=5 |
| 165 | 809 | A0A494BBD8\|A0A494BBD8_MOUSE | 32.01 | 3 | 3 | 1.54E+06 | 1 | 1 | 1 |  | 33945 | Annexin OS=Mus musculus OX=10090 GN=Anxa1 PE=1 SV=1 |
| 165 | 811 | Q4FJV4\|Q4FJV4_MOUSE | 32.01 | 2 | 2 | 1.54E+06 | 1 | 1 | 1 |  | 38734 | Annexin OS=Mus musculus OX=10090 GN=Anxa1 PE=1 SV=1 |
| 165 | 6039 | B7STB7\|B7STB7_MOUSE | 32.01 | 2 | 2 | 1.54E+06 | 1 | 1 | 1 |  | 38750 | Annexin OS=Mus musculus OX=10090 GN=Anxa1 PE=2 SV=1 |
| 165 | 812 | Q3U5N9\|Q3U5N9_MOUSE | 32.01 | 2 | 2 | 1.54E+06 | 1 | 1 | 1 |  | 38675 | Annexin OS=Mus musculus OX=10090 GN=Anxa1 PE=2 SV=1 |
| 165 | 6040 | sp\|P10107\|ANXA1_MOUSE | 32.01 | 2 | 2 | 1.54E+06 | 1 | 1 | 1 |  | 38734 | Annexin A1 OS=Mus musculus OX=10090 GN=Anxa1 PE=1 SV=2 |
| 165 | 6041 | Q4FK88\|Q4FK88_MOUSE | 32.01 | 2 | 2 | 1.54E+06 | 1 | 1 | 1 |  | 38734 | Annexin OS=Mus musculus OX=10090 GN=Anxa1 PE=2 SV=1 |
| 165 | 814 | Q3US43\|Q3US43_MOUSE | 32.01 | 2 | 2 | 1.54E+06 | 1 | 1 | 1 |  | 40289 | Annexin OS=Mus musculus OX=10090 GN=Anxa1 PE=2 SV=1 |
| 170 | 626 | Q545F8\|Q545F8_MOUSE | 31.36 | 3 | 3 | 1.49E+06 | 1 | 1 | 1 |  | 27504 | 40S ribosomal protein S4 OS=Mus musculus OX=10090 GN=Rps4x PE=2 SV=1 |
| 170 | 627 | Q3V1Z5\|Q3V1Z5_MOUSE | 31.36 | 3 | 3 | 1.49E+06 | 1 | 1 | 1 |  | 29184 | 40S ribosomal protein S4 OS=Mus musculus OX=10090 GN=Rps4l PE=2 SV=1 |
| 170 | 628 | sp\|P62702\|RS4X_MOUSE | 31.36 | 3 | 3 | 1.49E+06 | 1 | 1 | 1 |  | 29598 | 40S ribosomal protein S4 X isoform OS=Mus musculus OX=10090 GN=Rps4x PE=1 SV=2 |
| 170 | 629 | Q545X8\|Q545X8_MOUSE | 31.36 | 3 | 3 | 1.49E+06 | 1 | 1 | 1 |  | 29598 | 40S ribosomal protein S4 OS=Mus musculus OX=10090 GN=Rps4x PE=1 SV=1 |
| 170 | 630 | Q3UXQ6\|Q3UXQ6_MOUSE | 31.36 | 3 | 3 | 1.49E+06 | 1 | 1 | 1 |  | 29564 | 40S ribosomal protein S4 OS=Mus musculus OX=10090 GN=Rps4x PE=2 SV=1 |
| 170 | 867 | V9GWY0\|V9GWY0_MOUSE | 31.36 | 3 | 3 | 1.49E+06 | 1 | 1 | 1 |  | 29939 | 40S ribosomal protein S4 OS=Mus musculus OX=10090 GN=Gm15013 PE=3 SV=1 |
| 168 | 804 | A0A1L1SQA8\|A0A1L1SQA8_MOUSE | 30.99 | 11 | 11 | 1.32E+06 | 1 | 1 | 1 |  | 10309 | 40S ribosomal protein S25 OS=Mus musculus OX=10090 GN=Rps25 PE=1 SV=1 |
| 168 | 806 | sp\|P62852\|RS25_MOUSE | 30.99 | 8 | 8 | 1.32E+06 | 1 | 1 | 1 |  | 13742 | 40S ribosomal protein S25 OS=Mus musculus OX=10090 GN=Rps25 PE=1 SV=1 |
| 168 | 805 | Q58EA6\|Q58EA6_MOUSE | 30.99 | 8 | 8 | 1.32E+06 | 1 | 1 | 1 |  | 13742 | 40S ribosomal protein S25 OS=Mus musculus OX=10090 GN=Rps25 PE=1 SV=1 |
| 169 | 879 | Q52L87\|Q52L87_MOUSE | 30.84 | 2 | 2 | 4.14E+05 | 1 | 1 | 1 |  | 49909 | Tubulin alpha chain OS=Mus musculus OX=10090 GN=Tuba1c PE=1 SV=1 |
| 171 | 863 | D3YTL4\|D3YTL4_MOUSE | 30.08 | 12 | 12 | 1.09E+06 | 1 | 1 | 1 |  | 7074 | Troponin I slow skeletal muscle (Fragment) OS=Mus musculus OX=10090 GN=Tnni1 PE=1 SV=1 |
| 171 | 868 | D3YUN3\|D3YUN3_MOUSE | 30.08 | 4 | 4 | 1.09E+06 | 1 | 1 | 1 |  | 19843 | Troponin I slow skeletal muscle OS=Mus musculus OX=10090 GN=Tnni1 PE=1 SV=1 |
| 171 | 869 | F6QC77\|F6QC77_MOUSE | 30.08 | 4 | 4 | 1.09E+06 | 1 | 1 | 1 |  | 20947 | Troponin I slow skeletal muscle (Fragment) OS=Mus musculus OX=10090 GN=Tnni1 PE=1 SV=1 |
| 171 | 870 | sp\|Q9WUZ5\|TNNI1_MOUSE | 30.08 | 4 | 4 | 1.09E+06 | 1 | 1 | 1 |  | 21698 | Troponin I slow skeletal muscle OS=Mus musculus OX=10090 GN=Tnni1 PE=1 SV=3 |
| 172 | 615 | sp\|P97351\|RS3A_MOUSE | 28.57 | 3 | 3 | 6.05E+05 | 1 | 1 | 1 |  | 29885 | 40S ribosomal protein S3a OS=Mus musculus OX=10090 GN=Rps3a PE=1 SV=3 |
| 172 | 616 | Q9D1S3\|Q9D1S3_MOUSE | 28.57 | 3 | 3 | 6.05E+05 | 1 | 1 | 1 |  | 29886 | 40S ribosomal protein S3a OS=Mus musculus OX=10090 GN=Rps3a1 PE=2 SV=1 |
| 172 | 617 | Q3UAC2\|Q3UAC2_MOUSE | 28.57 | 3 | 3 | 6.05E+05 | 1 | 1 | 1 |  | 29913 | 40S ribosomal protein S3a OS=Mus musculus OX=10090 GN=Rps3a1 PE=2 SV=1 |
| 172 | 618 | Q3UJU5\|Q3UJU5_MOUSE | 28.57 | 3 | 3 | 6.05E+05 | 1 | 1 | 1 |  | 29943 | 40S ribosomal protein S3a OS=Mus musculus OX=10090 GN=Rps3a1 PE=2 SV=1 |
| 172 | 619 | Q564F3\|Q564F3_MOUSE | 28.57 | 3 | 3 | 6.05E+05 | 1 | 1 | 1 |  | 29885 | 40S ribosomal protein S3a OS=Mus musculus OX=10090 GN=Rps3a1 PE=1 SV=1 |
| 172 | 6044 | Q3U5P8\|Q3U5P8_MOUSE | 28.57 | 3 | 3 | 6.05E+05 | 1 | 1 | 1 |  | 29827 | 40S ribosomal protein S3a OS=Mus musculus OX=10090 GN=Rps3a1 PE=2 SV=1 |
| 89 | 873 | A2AQD6\|A2AQD6_MOUSE | 28.35 | 1 | 1 | 6.44E+06 | 1 | 1 | 3 |  | 133462 | Protein ITPRID2 OS=Mus musculus OX=10090 GN=Itprid2 PE=1 SV=1 |
| 89 | 874 | A2AQD5\|A2AQD5_MOUSE | 28.35 | 1 | 1 | 6.44E+06 | 1 | 1 | 3 |  | 134671 | Protein ITPRID2 OS=Mus musculus OX=10090 GN=Itprid2 PE=1 SV=1 |
| 89 | 875 | sp\|Q922B9\|ITPI2_MOUSE | 28.35 | 1 | 1 | 6.44E+06 | 1 | 1 | 3 |  | 136947 | Protein ITPRID2 OS=Mus musculus OX=10090 GN=Itprid2 PE=1 SV=3 |
| 173 | 852 | B0V2N8\|B0V2N8_MOUSE | 28.35 | 6 | 6 | 4.05E+05 | 1 | 1 | 1 |  | 19596 | Annexin (Fragment) OS=Mus musculus OX=10090 GN=Anxa2 PE=1 SV=1 |
| 173 | 853 | B0V2N7\|B0V2N7_MOUSE | 28.35 | 5 | 5 | 4.05E+05 | 1 | 1 | 1 |  | 21857 | Annexin (Fragment) OS=Mus musculus OX=10090 GN=Anxa2 PE=1 SV=1 |
| 173 | 854 | Q99KH3\|Q99KH3_MOUSE | 28.35 | 5 | 5 | 4.05E+05 | 1 | 1 | 1 |  | 25878 | Annexin OS=Mus musculus OX=10090 GN=Anxa2 PE=2 SV=1 |
| 173 | 855 | B0V2N5\|B0V2N5_MOUSE | 28.35 | 4 | 4 | 4.05E+05 | 1 | 1 | 1 |  | 31263 | Annexin (Fragment) OS=Mus musculus OX=10090 GN=Anxa2 PE=1 SV=1 |
| 173 | 856 | Q542G9\|Q542G9_MOUSE | 28.35 | 3 | 3 | 4.05E+05 | 1 | 1 | 1 |  | 38676 | Annexin OS=Mus musculus OX=10090 GN=Anxa2 PE=1 SV=1 |
| 173 | 858 | Q9CZI7\|Q9CZI7_MOUSE | 28.35 | 3 | 3 | 4.05E+05 | 1 | 1 | 1 |  | 38609 | Annexin OS=Mus musculus OX=10090 GN=Anxa2 PE=2 SV=1 |
| 173 | 857 | sp\|P07356\|ANXA2_MOUSE | 28.35 | 3 | 3 | 4.05E+05 | 1 | 1 | 1 |  | 38676 | Annexin A2 OS=Mus musculus OX=10090 GN=Anxa2 PE=1 SV=2 |
| 113 | 6080 | Q9DCU1\|Q9DCU1_MOUSE | 27.98 | 4 | 4 | 7.61E+05 | 1 | 1 | 1 | GSTs | 25361 | Glutathione S-transferase OS=Mus musculus OX=10090 GN=Gsta3 PE=2 SV=1 |
| 174 | 883 | sp\|P54071\|IDHP_MOUSE | 27.54 | 2 | 2 | 9.08E+05 | 1 | 1 | 1 |  | 50906 | Isocitrate dehydrogenase [NADP] mitochondrial OS=Mus musculus OX=10090 GN=Idh2 PE=1 SV=3 |
| 131 | 637 | Q78XY9\|Q78XY9_MOUSE | 26.66 | 3 | 3 | 1.26E+06 | 1 | 1 | 1 |  | 34369 | MCG23377 isoform CRA_a OS=Mus musculus OX=10090 GN=Ubb PE=2 SV=1 |
| 134 | 6055 | A0A0J9YV40\|A0A0J9YV40_MOUSE | 24.35 | 4 | 4 | 0.00E+00 | 1 | 1 | 1 | Pyro-glu from Q | 25514 | Histone RNA hairpin-binding protein OS=Mus musculus OX=10090 GN=Slbp PE=1 SV=1 |
| 134 | 6056 | Q3V2L1\|Q3V2L1_MOUSE | 24.35 | 3 | 3 | 0.00E+00 | 1 | 1 | 1 | Pyro-glu from Q | 28926 | Histone RNA hairpin-binding protein OS=Mus musculus OX=10090 GN=Slbp PE=1 SV=1 |
| 134 | 6057 | Q3U4T7\|Q3U4T7_MOUSE | 24.35 | 3 | 3 | 0.00E+00 | 1 | 1 | 1 | Pyro-glu from Q | 31603 | MCG16335 isoform CRA_b OS=Mus musculus OX=10090 GN=Slbp PE=1 SV=1 |
| 134 | 6058 | sp\|P97440\|SLBP_MOUSE | 24.35 | 3 | 3 | 0.00E+00 | 1 | 1 | 1 | Pyro-glu from Q | 31603 | Histone RNA hairpin-binding protein OS=Mus musculus OX=10090 GN=Slbp PE=1 SV=1 |
| 51 | 6059 | F6QFD1\|F6QFD1_MOUSE | 24.21 | 1 | 1 | 2.70E+07 | 1 | 1 | 4 | PDE | 85560 | Phosphodiesterase (Fragment) OS=Mus musculus OX=10090 GN=Pde4d PE=1 SV=1 |
| 175 | 639 | sp\|Q2UY11\|COSA1_MOUSE | 23.66 | 1 | 1 | 5.04E+05 | 1 | 1 | 1 | Oxidation (M) | 118749 | Collagen alpha-1(XXVIII) chain OS=Mus musculus OX=10090 GN=Col28a1 PE=2 SV=1 |
| 176 | 3578 | Q5DUA5\|Q5DUA5_MOUSE | 23.17 | 0 | 0 | 7.72E+05 | 1 | 1 | 1 |  | 361682 | Cadherin 23 OS=Mus musculus OX=10090 GN=Cdh23 PE=2 SV=1 |
| 176 | 6067 | E9Q7M6\|E9Q7M6_MOUSE | 23.17 | 0 | 0 | 7.72E+05 | 1 | 1 | 1 |  | 365772 | Cadherin-23 OS=Mus musculus OX=10090 GN=Cdh23 PE=1 SV=1 |
| 176 | 6068 | K4DI74\|K4DI74_MOUSE | 23.17 | 0 | 0 | 7.72E+05 | 1 | 1 | 1 |  | 369379 | Cadherin-23 OS=Mus musculus OX=10090 GN=Cdh23 PE=1 SV=1 |
| 176 | 3583 | Q5DUA3\|Q5DUA3_MOUSE | 23.17 | 0 | 0 | 7.72E+05 | 1 | 1 | 1 |  | 369365 | Cadherin 23 OS=Mus musculus OX=10090 GN=Cdh23 PE=2 SV=1 |
| 176 | 6069 | F8WIF5\|F8WIF5_MOUSE | 23.17 | 0 | 0 | 7.72E+05 | 1 | 1 | 1 |  | 369437 | Cadherin-23 OS=Mus musculus OX=10090 GN=Cdh23 PE=1 SV=1 |
| 135 | 974 | sp\|Q99KI0\|ACON_MOUSE | 22.9 | 1 | 1 | 1.18E+06 | 1 | 1 | 1 |  | 85464 | Aconitate hydratase mitochondrial OS=Mus musculus OX=10090 GN=Aco2 PE=1 SV=1 |
| 177 | 537 | sp\|P35980\|RL18_MOUSE | 22.68 | 4 | 4 | 8.44E+05 | 1 | 1 | 1 |  | 21645 | 60S ribosomal protein L18 OS=Mus musculus OX=10090 GN=Rpl18 PE=1 SV=3 |
| 177 | 536 | Q58EW0\|Q58EW0_MOUSE | 22.68 | 4 | 4 | 8.44E+05 | 1 | 1 | 1 |  | 21645 | MCG132477 isoform CRA_a OS=Mus musculus OX=10090 GN=Rpl18 PE=1 SV=1 |
| 177 | 538 | Q642K1\|Q642K1_MOUSE | 22.68 | 4 | 4 | 8.44E+05 | 1 | 1 | 1 |  | 21644 | Ribosomal protein L18 OS=Mus musculus OX=10090 GN=Rpl18 PE=2 SV=1 |
| 178 | 1003 | Q3U4N4\|Q3U4N4_MOUSE | 22.2 | 8 | 8 | 2.09E+06 | 1 | 1 | 1 |  | 21254 | Uncharacterized protein OS=Mus musculus OX=10090 GN=Cavin1 PE=2 SV=1 |
| 179 | 958 | A0A1L1SUT8\|A0A1L1SUT8_MOUSE | 21.57 | 12 | 12 | 7.23E+06 | 1 | 1 | 1 |  | 6523 | Cryptochrome-1 (Fragment) OS=Mus musculus OX=10090 GN=Cry1 PE=4 SV=1 |
| 179 | 972 | sp\|P97784\|CRY1_MOUSE | 21.57 | 1 | 1 | 7.23E+06 | 1 | 1 | 1 | CRY1 | 68001 | Cryptochrome-1 OS=Mus musculus OX=10090 GN=Cry1 PE=1 SV=1 |
| 137 | 530 | sp\|P56135\|ATPK_MOUSE | 20.83 | 10 | 10 | 1.90E+06 | 1 | 1 | 1 | Acetylation (Protein N-term) | 10344 | ATP synthase subunit f mitochondrial OS=Mus musculus OX=10090 GN=Atp5mf PE=1 SV=3 |
| 139 | 4257 | B2RUG6\|B2RUG6_MOUSE | 20.45 | 0 | 0 | 1.87E+07 | 1 | 1 | 1 |  | 222195 | Dock4 protein OS=Mus musculus OX=10090 GN=Dock4 PE=2 SV=1 |
| 139 | 4258 | sp\|P59764\|DOCK4_MOUSE | 20.45 | 0 | 0 | 1.87E+07 | 1 | 1 | 1 |  | 226548 | Dedicator of cytokinesis protein 4 OS=Mus musculus OX=10090 GN=Dock4 PE=1 SV=1 |
