# Supplementary material for: Tert promotes cardiac regenerative repair after MI through alleviating ROS-induced DNA damage response in cardiomyocyte
Source: Cell Death Discov. 2024 Aug 26;10:381. doi: 10.1038/s41420-024-02135-8 (PMC11347641; doi:10.1038/s41420-024-02135-8)
Supplement: Supplementary file 2 — Proteins identified by mass spectrometry from sample 2 [file 41420_2024_2135_MOESM2_ESM.docx]

Proteins identified by mass spectrometry from sample 2

| Protein Group | Protein ID | Accession | -10lgP | Coverage (%) | Coverage (%) 2 | Area 2 | #Peptides | #Unique | #Spec 2 | PTM | Avg. Mass | Description |
| --- | --- | --- | --- | --- | --- | --- | --- | --- | --- | --- | --- | --- |
| 2 | 5 | Q8C2Q8\|Q8C2Q8_MOUSE | 178.64 | 46 | 46 | 1.30E+08 | 14 | 14 | 44 | Carbamidomethylation; Oxidation (M); Pyro-glu from Q | 30256 | ATP synthase subunit gamma OS=Mus musculus OX=10090 GN=Atp5c1 PE=1 SV=1 |
| 2 | 6 | A2AKU9\|A2AKU9_MOUSE | 178.64 | 43 | 43 | 1.30E+08 | 14 | 14 | 44 | Carbamidomethylation; Oxidation (M); Pyro-glu from Q | 32771 | ATP synthase subunit gamma OS=Mus musculus OX=10090 GN=Atp5c1 PE=1 SV=1 |
| 2 | 10 | Q9ERA8\|Q9ERA8_MOUSE | 178.64 | 43 | 43 | 1.30E+08 | 14 | 14 | 44 | Carbamidomethylation; Oxidation (M); Pyro-glu from Q | 32852 | ATP synthase subunit gamma OS=Mus musculus OX=10090 GN=Atp5c1 PE=2 SV=1 |
| 2 | 8 | sp\|Q91VR2\|ATPG_MOUSE | 178.64 | 43 | 43 | 1.30E+08 | 14 | 14 | 44 | Carbamidomethylation; Oxidation (M); Pyro-glu from Q | 32886 | ATP synthase subunit gamma mitochondrial OS=Mus musculus OX=10090 GN=Atp5f1c PE=1 SV=1 |
| 2 | 7 | Q3UD06\|Q3UD06_MOUSE | 178.64 | 43 | 43 | 1.30E+08 | 14 | 14 | 44 | Carbamidomethylation; Oxidation (M); Pyro-glu from Q | 32886 | ATP synthase subunit gamma OS=Mus musculus OX=10090 GN=Atp5c1 PE=1 SV=1 |
| 4 | 11 | sp\|P56480\|ATPB_MOUSE | 172.35 | 31 | 31 | 1.20E+08 | 13 | 13 | 40 | Oxidation (M) | 56301 | ATP synthase subunit beta mitochondrial OS=Mus musculus OX=10090 GN=Atp5f1b PE=1 SV=2 |
| 9 | 50 | sp\|P15864\|H12_MOUSE | 172.21 | 44 | 44 | 2.38E+07 | 12 | 4 | 36 | Acetylation (Protein N-term); Deamidation (NQ) | 21267 | Histone H1.2 OS=Mus musculus OX=10090 GN=H1-2 PE=1 SV=2 |
| 9 | 51 | Q5SZA3\|Q5SZA3_MOUSE | 172.21 | 44 | 44 | 2.38E+07 | 12 | 4 | 36 | Acetylation (Protein N-term); Deamidation (NQ) | 21267 | Histone cluster 1 H1c OS=Mus musculus OX=10090 GN=H1f2 PE=1 SV=1 |
| 10 | 12 | Q3V235\|Q3V235_MOUSE | 167.54 | 53 | 53 | 6.08E+07 | 19 | 19 | 34 | Acetylation (Protein N-term); Oxidation (M); Pyro-glu from Q | 33296 | Prohibitin OS=Mus musculus OX=10090 GN=Phb2 PE=1 SV=1 |
| 10 | 13 | sp\|O35129\|PHB2_MOUSE | 167.54 | 53 | 53 | 6.08E+07 | 19 | 19 | 34 | Acetylation (Protein N-term); Oxidation (M); Pyro-glu from Q | 33296 | Prohibitin-2 OS=Mus musculus OX=10090 GN=Phb2 PE=1 SV=1 |
| 12 | 101 | sp\|P43274\|H14_MOUSE | 162.89 | 36 | 36 | 2.36E+07 | 9 | 3 | 30 | Acetylation (Protein N-term); Deamidation (NQ) | 21977 | Histone H1.4 OS=Mus musculus OX=10090 GN=H1-4 PE=1 SV=2 |
| 11 | 75 | Q3U6K8\|Q3U6K8_MOUSE | 147.89 | 30 | 30 | 6.86E+07 | 11 | 7 | 30 | Carbamidomethylation; Acetylation (Protein N-term); Deamidation (NQ) | 30725 | Uncharacterized protein OS=Mus musculus OX=10090 GN=Vdac1 PE=2 SV=1 |
| 11 | 76 | sp\|Q60932\|VDAC1_MOUSE | 147.89 | 29 | 29 | 6.86E+07 | 11 | 7 | 30 | Carbamidomethylation; Acetylation (Protein N-term); Deamidation (NQ) | 32351 | Voltage-dependent anion-selective channel protein 1 OS=Mus musculus OX=10090 GN=Vdac1 PE=1 SV=3 |
| 5 | 288 | sp\|P01868\|IGHG1_MOUSE | 147.78 | 23 | 23 | 8.24E+08 | 5 | 4 | 36 | Carbamidomethylation; Deamidation (NQ); Oxidation (M) | 35705 | Ig gamma-1 chain C region secreted form OS=Mus musculus OX=10090 GN=Ighg1 PE=1 SV=1 |
| 5 | 289 | sp\|P01869\|IGH1M_MOUSE | 147.78 | 19 | 19 | 8.24E+08 | 5 | 4 | 36 | Carbamidomethylation; Deamidation (NQ); Oxidation (M) | 43387 | Ig gamma-1 chain C region membrane-bound form OS=Mus musculus OX=10090 GN=Ighg1 PE=1 SV=2 |
| 5 | 249 | A0A0M4KM70\|A0A0M4KM70_MOUSE | 147.78 | 17 | 17 | 8.24E+08 | 5 | 4 | 36 | Carbamidomethylation; Deamidation (NQ); Oxidation (M) | 50295 | Monoclonal 11D8 anti-human butyrylcholinesterase (BChE) heavy chain OS=Mus musculus OX=10090 PE=2 SV=1 |
| 5 | 251 | Q99LC4\|Q99LC4_MOUSE | 147.78 | 16 | 16 | 8.24E+08 | 5 | 4 | 36 | Carbamidomethylation; Deamidation (NQ); Oxidation (M) | 51008 | Igh protein OS=Mus musculus OX=10090 GN=Igh PE=1 SV=1 |
| 5 | 250 | U5LP42\|U5LP42_MOUSE | 147.78 | 16 | 16 | 8.24E+08 | 5 | 4 | 36 | Carbamidomethylation; Deamidation (NQ); Oxidation (M) | 51051 | Anti-H5N1 hemagglutinin monoclonal anitbody H5M9 heavy chain (Fragment) OS=Mus musculus OX=10090 PE=2 SV=1 |
| 14 | 14 | sp\|P48962\|ADT1_MOUSE | 146.18 | 35 | 35 | 1.24E+07 | 12 | 5 | 23 | Carbamidomethylation; Acetylation (Protein N-term); Deamidation (NQ); Pyro-glu from Q | 32904 | ADP/ATP translocase 1 OS=Mus musculus OX=10090 GN=Slc25a4 PE=1 SV=4 |
| 23 | 52 | sp\|P63268\|ACTH_MOUSE | 141.68 | 20 | 20 | 1.21E+07 | 8 | 2 | 14 | Oxidation (M) | 41877 | Actin gamma-enteric smooth muscle OS=Mus musculus OX=10090 GN=Actg2 PE=1 SV=1 |
| 23 | 53 | Q3UJ36\|Q3UJ36_MOUSE | 141.68 | 20 | 20 | 1.21E+07 | 8 | 2 | 14 | Oxidation (M) | 41877 | Actin gamma 2 smooth muscle enteric isoform CRA_a OS=Mus musculus OX=10090 GN=Actg2 PE=2 SV=1 |
| 23 | 54 | Q3U122\|Q3U122_MOUSE | 141.68 | 20 | 20 | 1.21E+07 | 8 | 2 | 14 | Oxidation (M) | 41995 | Uncharacterized protein OS=Mus musculus OX=10090 GN=Acta2 PE=2 SV=1 |
| 23 | 25 | Q9CXK3\|Q9CXK3_MOUSE | 141.68 | 20 | 20 | 1.21E+07 | 8 | 2 | 14 | Oxidation (M) | 41947 | Uncharacterized protein OS=Mus musculus OX=10090 GN=Actc1 PE=2 SV=1 |
| 23 | 31 | Q3TG92\|Q3TG92_MOUSE | 141.68 | 20 | 20 | 1.21E+07 | 8 | 2 | 14 | Oxidation (M) | 42047 | Uncharacterized protein OS=Mus musculus OX=10090 GN=Actc1 PE=2 SV=1 |
| 23 | 29 | sp\|P68134\|ACTS_MOUSE | 141.68 | 20 | 20 | 1.21E+07 | 8 | 2 | 14 | Oxidation (M) | 42051 | Actin alpha skeletal muscle OS=Mus musculus OX=10090 GN=Acta1 PE=1 SV=1 |
| 23 | 26 | sp\|P68033\|ACTC_MOUSE | 141.68 | 20 | 20 | 1.21E+07 | 8 | 2 | 14 | Oxidation (M) | 42019 | Actin alpha cardiac muscle 1 OS=Mus musculus OX=10090 GN=Actc1 PE=1 SV=1 |
| 23 | 27 | sp\|P62737\|ACTA_MOUSE | 141.68 | 20 | 20 | 1.21E+07 | 8 | 2 | 14 | Oxidation (M) | 42009 | Actin aortic smooth muscle OS=Mus musculus OX=10090 GN=Acta2 PE=1 SV=1 |
| 23 | 28 | Q497E4\|Q497E4_MOUSE | 141.68 | 20 | 20 | 1.21E+07 | 8 | 2 | 14 | Oxidation (M) | 42019 | Actin alpha cardiac muscle 1 OS=Mus musculus OX=10090 GN=Actc1 PE=2 SV=1 |
| 16 | 98 | D2KHZ9\|D2KHZ9_MOUSE | 139.29 | 27 | 27 | 5.68E+07 | 8 | 8 | 21 | Carbamidomethylation; Deamidation (NQ) | 35810 | Glyceraldehyde-3-phosphate dehydrogenase OS=Mus musculus OX=10090 GN=GAPDH PE=2 SV=1 |
| 16 | 97 | sp\|P16858\|G3P_MOUSE | 139.29 | 27 | 27 | 5.68E+07 | 8 | 8 | 21 | Carbamidomethylation; Deamidation (NQ) | 35810 | Glyceraldehyde-3-phosphate dehydrogenase OS=Mus musculus OX=10090 GN=Gapdh PE=1 SV=2 |
| 16 | 99 | A0A0A0MQF6\|A0A0A0MQF6_MOUSE | 139.29 | 25 | 25 | 5.68E+07 | 8 | 8 | 21 | Carbamidomethylation; Deamidation (NQ) | 38653 | Glyceraldehyde-3-phosphate dehydrogenase OS=Mus musculus OX=10090 GN=Gapdh PE=1 SV=1 |
| 25 | 137 | Q1WWK3\|Q1WWK3_MOUSE | 136.06 | 27 | 27 | 1.42E+07 | 7 | 5 | 13 | Acetylation (Protein N-term) | 22445 | Hist1h1b protein (Fragment) OS=Mus musculus OX=10090 GN=H1f5 PE=2 SV=1 |
| 25 | 138 | sp\|P43276\|H15_MOUSE | 136.06 | 27 | 27 | 1.42E+07 | 7 | 5 | 13 | Acetylation (Protein N-term) | 22576 | Histone H1.5 OS=Mus musculus OX=10090 GN=H1-5 PE=1 SV=2 |
| 22 | 22 | sp\|P67778\|PHB_MOUSE | 131.88 | 34 | 34 | 2.50E+07 | 10 | 10 | 15 | Pyro-glu from Q | 29820 | Prohibitin OS=Mus musculus OX=10090 GN=Phb PE=1 SV=1 |
| 24 | 67 | B2RQQ1\|B2RQQ1_MOUSE | 129.45 | 6 | 6 | 1.93E+06 | 14 | 4 | 14 | Deamidation (NQ); Pyro-glu from Q | 223563 | MCG133649 isoform CRA_a OS=Mus musculus OX=10090 GN=Myh6 PE=1 SV=1 |
| 24 | 68 | sp\|Q02566\|MYH6_MOUSE | 129.45 | 6 | 6 | 1.93E+06 | 14 | 4 | 14 | Deamidation (NQ); Pyro-glu from Q | 223563 | Myosin-6 OS=Mus musculus OX=10090 GN=Myh6 PE=1 SV=2 |
| 24 | 69 | Q2TAW4\|Q2TAW4_MOUSE | 129.45 | 6 | 6 | 1.93E+06 | 14 | 4 | 14 | Deamidation (NQ); Pyro-glu from Q | 223562 | Myosin heavy polypeptide 6 cardiac muscle alpha OS=Mus musculus OX=10090 GN=Myh6 PE=2 SV=1 |
| 21 | 70 | sp\|P62259\|1433E_MOUSE | 124.14 | 25 | 25 | 2.53E+07 | 8 | 6 | 14 | Acetylation (Protein N-term); Deamidation (NQ) | 29174 | 14-3-3 protein epsilon OS=Mus musculus OX=10090 GN=Ywhae PE=1 SV=1 |
| 21 | 71 | Q8BPH1\|Q8BPH1_MOUSE | 124.14 | 25 | 25 | 2.53E+07 | 8 | 6 | 14 | Acetylation (Protein N-term); Deamidation (NQ) | 29189 | 14_3_3 domain-containing protein OS=Mus musculus OX=10090 GN=Ywhae PE=2 SV=1 |
| 21 | 72 | Q5SS40\|Q5SS40_MOUSE | 124.14 | 25 | 25 | 2.53E+07 | 8 | 6 | 14 | Acetylation (Protein N-term); Deamidation (NQ) | 29174 | Tyrosine 3-monooxygenase/tryptophan 5-monooxygenase activation protein epsilon polypeptide isoform CRA_c OS=Mus musculus OX=10090 GN=Ywhae PE=1 SV=1 |
| 27 | 43 | Q4KL81\|Q4KL81_MOUSE | 121.82 | 15 | 15 | 3.15E+06 | 7 | 1 | 12 | Oxidation (M) | 41793 | Actin gamma cytoplasmic 1 OS=Mus musculus OX=10090 GN=Actg1 PE=2 SV=1 |
| 27 | 44 | Q3U5R4\|Q3U5R4_MOUSE | 121.82 | 15 | 15 | 3.15E+06 | 7 | 1 | 12 | Oxidation (M) | 41709 | Uncharacterized protein OS=Mus musculus OX=10090 GN=Actb PE=2 SV=1 |
| 27 | 45 | sp\|P63260\|ACTG_MOUSE | 121.82 | 15 | 15 | 3.15E+06 | 7 | 1 | 12 | Oxidation (M) | 41793 | Actin cytoplasmic 2 OS=Mus musculus OX=10090 GN=Actg1 PE=1 SV=1 |
| 27 | 42 | Q3UAF6\|Q3UAF6_MOUSE | 121.82 | 15 | 15 | 3.15E+06 | 7 | 1 | 12 | Oxidation (M) | 41811 | Uncharacterized protein OS=Mus musculus OX=10090 GN=Actb PE=2 SV=1 |
| 27 | 46 | sp\|P60710\|ACTB_MOUSE | 121.82 | 15 | 15 | 3.15E+06 | 7 | 1 | 12 | Oxidation (M) | 41737 | Actin cytoplasmic 1 OS=Mus musculus OX=10090 GN=Actb PE=1 SV=1 |
| 27 | 47 | B2RRX1\|B2RRX1_MOUSE | 121.82 | 15 | 15 | 3.15E+06 | 7 | 1 | 12 | Oxidation (M) | 41737 | Actin beta OS=Mus musculus OX=10090 GN=Actb PE=2 SV=1 |
| 27 | 48 | Q3UAF7\|Q3UAF7_MOUSE | 121.82 | 15 | 15 | 3.15E+06 | 7 | 1 | 12 | Oxidation (M) | 41751 | Uncharacterized protein OS=Mus musculus OX=10090 GN=Actb PE=2 SV=1 |
| 33 | 39 | B2RY26\|B2RY26_MOUSE | 121.47 | 5 | 5 | 3.52E+05 | 11 | 1 | 11 |  | 222847 | Myh7 protein OS=Mus musculus OX=10090 GN=Myh7 PE=2 SV=1 |
| 33 | 41 | B2RXX9\|B2RXX9_MOUSE | 121.47 | 5 | 5 | 3.52E+05 | 11 | 1 | 11 |  | 222877 | Myosin heavy polypeptide 7 cardiac muscle beta OS=Mus musculus OX=10090 GN=Myh7 PE=2 SV=1 |
| 33 | 40 | sp\|Q91Z83\|MYH7_MOUSE | 121.47 | 5 | 5 | 3.52E+05 | 11 | 1 | 11 |  | 222877 | Myosin-7 OS=Mus musculus OX=10090 GN=Myh7 PE=2 SV=1 |
| 30 | 16 | Q3TRH3\|Q3TRH3_MOUSE | 121.3 | 14 | 14 | 1.57E+07 | 8 | 7 | 10 | Carbamidomethylation | 70901 | Uncharacterized protein OS=Mus musculus OX=10090 GN=Hspa8 PE=2 SV=1 |
| 30 | 17 | Q504P4\|Q504P4_MOUSE | 121.3 | 15 | 15 | 1.57E+07 | 8 | 7 | 10 | Carbamidomethylation | 68779 | Heat shock cognate 71 kDa protein OS=Mus musculus OX=10090 GN=Hspa8 PE=1 SV=1 |
| 30 | 20 | Q3UBA6\|Q3UBA6_MOUSE | 121.3 | 14 | 14 | 1.57E+07 | 8 | 7 | 10 | Carbamidomethylation | 70899 | Uncharacterized protein OS=Mus musculus OX=10090 GN=Hspa8 PE=2 SV=1 |
| 30 | 18 | sp\|P63017\|HSP7C_MOUSE | 121.3 | 14 | 14 | 1.57E+07 | 8 | 7 | 10 | Carbamidomethylation | 70871 | Heat shock cognate 71 kDa protein OS=Mus musculus OX=10090 GN=Hspa8 PE=1 SV=1 |
| 30 | 15 | Q3TEK2\|Q3TEK2_MOUSE | 121.3 | 14 | 14 | 1.57E+07 | 8 | 7 | 10 | Carbamidomethylation | 70857 | Uncharacterized protein OS=Mus musculus OX=10090 GN=Hspa8 PE=2 SV=1 |
| 30 | 37 | Q3TF16\|Q3TF16_MOUSE | 121.3 | 14 | 14 | 1.57E+07 | 8 | 7 | 10 | Carbamidomethylation | 70885 | Uncharacterized protein OS=Mus musculus OX=10090 GN=Hspa8 PE=2 SV=1 |
| 30 | 21 | Q3TH56\|Q3TH56_MOUSE | 121.3 | 14 | 14 | 1.57E+07 | 8 | 7 | 10 | Carbamidomethylation | 70872 | Uncharacterized protein OS=Mus musculus OX=10090 GN=Hspa8 PE=2 SV=1 |
| 30 | 19 | Q3TQ13\|Q3TQ13_MOUSE | 121.3 | 14 | 14 | 1.57E+07 | 8 | 7 | 10 | Carbamidomethylation | 70871 | Uncharacterized protein OS=Mus musculus OX=10090 GN=Hspa8 PE=2 SV=1 |
| 19 | 94 | sp\|P51881\|ADT2_MOUSE | 121.22 | 23 | 23 | 5.32E+06 | 9 | 2 | 17 | Acetylation (Protein N-term) | 32931 | ADP/ATP translocase 2 OS=Mus musculus OX=10090 GN=Slc25a5 PE=1 SV=3 |
| 19 | 95 | Q545A2\|Q545A2_MOUSE | 121.22 | 23 | 23 | 5.32E+06 | 9 | 2 | 17 | Acetylation (Protein N-term) | 32931 | MCG11560 OS=Mus musculus OX=10090 GN=Slc25a5 PE=1 SV=1 |
| 41 | 128 | sp\|Q9DCT2\|NDUS3_MOUSE | 115.26 | 16 | 16 | 2.40E+07 | 4 | 4 | 8 |  | 30149 | NADH dehydrogenase [ubiquinone] iron-sulfur protein 3 mitochondrial OS=Mus musculus OX=10090 GN=Ndufs3 PE=1 SV=2 |
| 29 | 64 | sp\|P08249\|MDHM_MOUSE | 112.89 | 25 | 25 | 1.52E+07 | 8 | 8 | 11 | Carbamidomethylation | 35611 | Malate dehydrogenase mitochondrial OS=Mus musculus OX=10090 GN=Mdh2 PE=1 SV=3 |
| 37 | 91 | sp\|Q9DCW4\|ETFB_MOUSE | 112.25 | 24 | 24 | 1.05E+07 | 6 | 6 | 9 |  | 27623 | Electron transfer flavoprotein subunit beta OS=Mus musculus OX=10090 GN=Etfb PE=1 SV=3 |
| 26 | 109 | D3Z6F5\|D3Z6F5_MOUSE | 109.84 | 19 | 19 | 1.55E+07 | 12 | 11 | 13 | Pyro-glu from Q | 54595 | ATP synthase subunit alpha OS=Mus musculus OX=10090 GN=Atp5a1 PE=1 SV=1 |
| 26 | 108 | sp\|Q03265\|ATPA_MOUSE | 109.84 | 17 | 17 | 1.55E+07 | 12 | 11 | 13 | Pyro-glu from Q | 59753 | ATP synthase subunit alpha mitochondrial OS=Mus musculus OX=10090 GN=Atp5f1a PE=1 SV=1 |
| 40 | 82 | sp\|Q9DB77\|QCR2_MOUSE | 109.07 | 17 | 17 | 1.15E+07 | 7 | 7 | 8 |  | 48235 | Cytochrome b-c1 complex subunit 2 mitochondrial OS=Mus musculus OX=10090 GN=Uqcrc2 PE=1 SV=1 |
| 31 | 122 | sp\|P10126\|EF1A1_MOUSE | 108.69 | 13 | 13 | 5.40E+07 | 7 | 7 | 10 |  | 50114 | Elongation factor 1-alpha 1 OS=Mus musculus OX=10090 GN=Eef1a1 PE=1 SV=3 |
| 31 | 123 | Q3UA81\|Q3UA81_MOUSE | 108.69 | 13 | 13 | 5.40E+07 | 7 | 7 | 10 |  | 50113 | Elongation factor 1-alpha OS=Mus musculus OX=10090 GN=Eef1a1 PE=2 SV=1 |
| 31 | 124 | Q3UZQ3\|Q3UZQ3_MOUSE | 108.69 | 13 | 13 | 5.40E+07 | 7 | 7 | 10 |  | 50066 | Elongation factor 1-alpha OS=Mus musculus OX=10090 GN=Eef1a1 PE=2 SV=1 |
| 31 | 125 | Q3TII3\|Q3TII3_MOUSE | 108.69 | 13 | 13 | 5.40E+07 | 7 | 7 | 10 |  | 50104 | Elongation factor 1-alpha OS=Mus musculus OX=10090 GN=Eef1a1 PE=2 SV=1 |
| 31 | 121 | Q58E64\|Q58E64_MOUSE | 108.69 | 13 | 13 | 5.40E+07 | 7 | 7 | 10 |  | 50114 | Elongation factor 1-alpha OS=Mus musculus OX=10090 GN=Eef1a1 PE=1 SV=1 |
| 36 | 153 | sp\|Q99LC5\|ETFA_MOUSE | 106.64 | 20 | 20 | 1.70E+07 | 6 | 6 | 9 | Carbamidomethylation | 35009 | Electron transfer flavoprotein subunit alpha mitochondrial OS=Mus musculus OX=10090 GN=Etfa PE=1 SV=2 |
| 45 | 55 | sp\|P62908\|RS3_MOUSE | 102.95 | 20 | 20 | 5.40E+06 | 5 | 5 | 6 | Carbamidomethylation | 26674 | 40S ribosomal protein S3 OS=Mus musculus OX=10090 GN=Rps3 PE=1 SV=1 |
| 45 | 57 | Q9CZP6\|Q9CZP6_MOUSE | 102.95 | 20 | 20 | 5.40E+06 | 5 | 5 | 6 | Carbamidomethylation | 26602 | KH type-2 domain-containing protein OS=Mus musculus OX=10090 GN=Rps3 PE=2 SV=1 |
| 45 | 56 | Q5YLW3\|Q5YLW3_MOUSE | 102.95 | 20 | 20 | 5.40E+06 | 5 | 5 | 6 | Carbamidomethylation | 26674 | Ribosomal protein S3 OS=Mus musculus OX=10090 GN=Rps3 PE=1 SV=1 |
| 32 | 179 | sp\|P43275\|H11_MOUSE | 102.79 | 27 | 27 | 3.87E+06 | 5 | 3 | 10 | Acetylation (Protein N-term) | 21785 | Histone H1.1 OS=Mus musculus OX=10090 GN=H1-1 PE=1 SV=2 |
| 35 | 112 | Q3TJD4\|Q3TJD4_MOUSE | 102.76 | 18 | 18 | 1.52E+07 | 7 | 7 | 10 | Pyro-glu from Q | 28948 | Uncharacterized protein OS=Mus musculus OX=10090 GN=Atp5pb PE=2 SV=1 |
| 35 | 113 | sp\|Q9CQQ7\|AT5F1_MOUSE | 102.76 | 18 | 18 | 1.52E+07 | 7 | 7 | 10 | Pyro-glu from Q | 28949 | ATP synthase F(0) complex subunit B1 mitochondrial OS=Mus musculus OX=10090 GN=Atp5pb PE=1 SV=1 |
| 35 | 111 | Q3UF04\|Q3UF04_MOUSE | 102.76 | 18 | 18 | 1.52E+07 | 7 | 7 | 10 | Pyro-glu from Q | 28919 | Uncharacterized protein OS=Mus musculus OX=10090 GN=Atp5pb PE=2 SV=1 |
| 35 | 114 | Q5I0W0\|Q5I0W0_MOUSE | 102.76 | 18 | 18 | 1.52E+07 | 7 | 7 | 10 | Pyro-glu from Q | 28949 | ATP synthase H+ transporting mitochondrial F0 complex subunit b isoform 1 OS=Mus musculus OX=10090 GN=Atp5pb PE=1 SV=1 |
| 47 | 154 | sp\|Q99JY0\|ECHB_MOUSE | 96.85 | 11 | 11 | 7.23E+06 | 6 | 6 | 7 |  | 51386 | Trifunctional enzyme subunit beta mitochondrial OS=Mus musculus OX=10090 GN=Hadhb PE=1 SV=1 |
| 48 | 267 | Q3TWG9\|Q3TWG9_MOUSE | 87.86 | 7 | 7 | 1.19E+07 | 3 | 3 | 7 |  | 46530 | SERPIN domain-containing protein OS=Mus musculus OX=10090 GN=Serpinh1 PE=2 SV=1 |
| 48 | 268 | Q3TJK3\|Q3TJK3_MOUSE | 87.86 | 7 | 7 | 1.19E+07 | 3 | 3 | 7 |  | 46535 | SERPIN domain-containing protein OS=Mus musculus OX=10090 GN=Serpinh1 PE=2 SV=1 |
| 48 | 269 | Q3TMD2\|Q3TMD2_MOUSE | 87.86 | 7 | 7 | 1.19E+07 | 3 | 3 | 7 |  | 46520 | SERPIN domain-containing protein OS=Mus musculus OX=10090 GN=Serpinh1 PE=2 SV=1 |
| 48 | 270 | Q8BVU9\|Q8BVU9_MOUSE | 87.86 | 7 | 7 | 1.19E+07 | 3 | 3 | 7 |  | 46511 | SERPIN domain-containing protein OS=Mus musculus OX=10090 GN=Serpinh1 PE=2 SV=1 |
| 48 | 271 | sp\|P19324\|SERPH_MOUSE | 87.86 | 7 | 7 | 1.19E+07 | 3 | 3 | 7 |  | 46534 | Serpin H1 OS=Mus musculus OX=10090 GN=Serpinh1 PE=1 SV=3 |
| 48 | 272 | Q8BV87\|Q8BV87_MOUSE | 87.86 | 7 | 7 | 1.19E+07 | 3 | 3 | 7 |  | 46520 | SERPIN domain-containing protein OS=Mus musculus OX=10090 GN=Serpinh1 PE=2 SV=1 |
| 69 | 252 | sp\|Q9DB20\|ATPO_MOUSE | 87.75 | 14 | 14 | 4.07E+06 | 3 | 3 | 3 |  | 23364 | ATP synthase subunit O mitochondrial OS=Mus musculus OX=10090 GN=Atp5po PE=1 SV=1 |
| 69 | 253 | Q3TF25\|Q3TF25_MOUSE | 87.75 | 14 | 14 | 4.07E+06 | 3 | 3 | 3 |  | 23364 | Uncharacterized protein OS=Mus musculus OX=10090 GN=Atp5o PE=2 SV=1 |
| 61 | 203 | sp\|Q9D051\|ODPB_MOUSE | 87.54 | 12 | 12 | 4.13E+06 | 4 | 4 | 4 |  | 38937 | Pyruvate dehydrogenase E1 component subunit beta mitochondrial OS=Mus musculus OX=10090 GN=Pdhb PE=1 SV=1 |
| 53 | 188 | sp\|Q9CR62\|M2OM_MOUSE | 84.68 | 9 | 9 | 2.35E+06 | 3 | 3 | 3 | Acetylation (Protein N-term) | 34155 | Mitochondrial 2-oxoglutarate/malate carrier protein OS=Mus musculus OX=10090 GN=Slc25a11 PE=1 SV=3 |
| 53 | 189 | Q5SX53\|Q5SX53_MOUSE | 84.68 | 9 | 9 | 2.35E+06 | 3 | 3 | 3 | Acetylation (Protein N-term) | 34155 | Solute carrier family 25 (Mitochondrial carrier oxoglutarate carrier) member 11 isoform CRA_b OS=Mus musculus OX=10090 GN=Slc25a11 PE=1 SV=1 |
| 54 | 266 | Q3TXR5\|Q3TXR5_MOUSE | 82.1 | 14 | 14 | 6.43E+06 | 4 | 4 | 5 |  | 24206 | S5 DRBM domain-containing protein OS=Mus musculus OX=10090 GN=Rps2 PE=2 SV=1 |
| 54 | 273 | D3YVC1\|D3YVC1_MOUSE | 82.1 | 12 | 12 | 6.43E+06 | 4 | 4 | 5 |  | 28601 | 40S ribosomal protein S2 (Fragment) OS=Mus musculus OX=10090 GN=Rps2 PE=1 SV=1 |
| 54 | 274 | Q58EU3\|Q58EU3_MOUSE | 82.1 | 11 | 11 | 6.43E+06 | 4 | 4 | 5 |  | 31231 | MCG12811 isoform CRA_b OS=Mus musculus OX=10090 GN=Rps2 PE=1 SV=1 |
| 54 | 275 | Q3TI78\|Q3TI78_MOUSE | 82.1 | 11 | 11 | 6.43E+06 | 4 | 4 | 5 |  | 31209 | S5 DRBM domain-containing protein OS=Mus musculus OX=10090 GN=Rps2 PE=2 SV=1 |
| 54 | 277 | sp\|P25444\|RS2_MOUSE | 82.1 | 11 | 11 | 6.43E+06 | 4 | 4 | 5 |  | 31231 | 40S ribosomal protein S2 OS=Mus musculus OX=10090 GN=Rps2 PE=1 SV=3 |
| 54 | 276 | Q3TL20\|Q3TL20_MOUSE | 82.1 | 11 | 11 | 6.43E+06 | 4 | 4 | 5 |  | 31230 | S5 DRBM domain-containing protein OS=Mus musculus OX=10090 GN=Rps2 PE=2 SV=1 |
| 54 | 278 | Q3TXS9\|Q3TXS9_MOUSE | 82.1 | 11 | 11 | 6.43E+06 | 4 | 4 | 5 |  | 31188 | S5 DRBM domain-containing protein OS=Mus musculus OX=10090 GN=Rps2 PE=2 SV=1 |
| 54 | 279 | Q3TLE5\|Q3TLE5_MOUSE | 82.1 | 11 | 11 | 6.43E+06 | 4 | 4 | 5 |  | 31255 | S5 DRBM domain-containing protein OS=Mus musculus OX=10090 GN=Rps2 PE=2 SV=1 |
| 54 | 280 | Q3UB36\|Q3UB36_MOUSE | 82.1 | 11 | 11 | 6.43E+06 | 4 | 4 | 5 |  | 31682 | S5 DRBM domain-containing protein OS=Mus musculus OX=10090 GN=Rps2 PE=2 SV=1 |
| 54 | 281 | D3YWJ3\|D3YWJ3_MOUSE | 82.1 | 11 | 11 | 6.43E+06 | 4 | 4 | 5 |  | 32105 | 40S ribosomal protein S2 OS=Mus musculus OX=10090 GN=Rps2 PE=1 SV=1 |
| 50 | 243 | sp\|Q3UV17\|K22O_MOUSE | 78.18 | 4 | 4 | 3.00E+07 | 4 | 2 | 6 |  | 62845 | Keratin type II cytoskeletal 2 oral OS=Mus musculus OX=10090 GN=Krt76 PE=1 SV=1 |
| 49 | 405 | sp\|Q60931\|VDAC3_MOUSE | 76.24 | 10 | 10 | 6.92E+05 | 4 | 1 | 6 |  | 30753 | Voltage-dependent anion-selective channel protein 3 OS=Mus musculus OX=10090 GN=Vdac3 PE=1 SV=1 |
| 49 | 404 | Q3TX38\|Q3TX38_MOUSE | 76.24 | 10 | 10 | 6.92E+05 | 4 | 1 | 6 |  | 30753 | Uncharacterized protein OS=Mus musculus OX=10090 GN=Vdac3 PE=1 SV=1 |
| 49 | 407 | J3QMG3\|J3QMG3_MOUSE | 76.24 | 10 | 10 | 6.92E+05 | 4 | 1 | 6 |  | 30852 | Voltage-dependent anion-selective channel protein 3 OS=Mus musculus OX=10090 GN=Vdac3 PE=1 SV=1 |
| 49 | 406 | Q5EBQ0\|Q5EBQ0_MOUSE | 76.24 | 10 | 10 | 6.92E+05 | 4 | 1 | 6 |  | 30884 | Voltage-dependent anion channel 3 OS=Mus musculus OX=10090 GN=Vdac3 PE=2 SV=1 |
| 62 | 206 | A2A5N1\|A2A5N1_MOUSE | 74.08 | 21 | 21 | 2.40E+06 | 4 | 2 | 4 |  | 18349 | 14-3-3 protein beta/alpha (Fragment) OS=Mus musculus OX=10090 GN=Ywhab PE=1 SV=1 |
| 62 | 207 | sp\|Q9CQV8\|1433B_MOUSE | 74.08 | 14 | 14 | 2.40E+06 | 4 | 2 | 4 |  | 28086 | 14-3-3 protein beta/alpha OS=Mus musculus OX=10090 GN=Ywhab PE=1 SV=3 |
| 62 | 208 | A2A5N2\|A2A5N2_MOUSE | 74.08 | 14 | 14 | 2.40E+06 | 4 | 2 | 4 |  | 28086 | Tyrosine 3-monooxygenase/tryptophan 5-monooxygenase activation protein beta polypeptide OS=Mus musculus OX=10090 GN=Ywhab PE=2 SV=1 |
| 55 | 216 | sp\|Q61425\|HCDH_MOUSE | 71.97 | 9 | 9 | 5.50E+06 | 4 | 4 | 5 |  | 34464 | Hydroxyacyl-coenzyme A dehydrogenase mitochondrial OS=Mus musculus OX=10090 GN=Hadh PE=1 SV=2 |
| 64 | 195 | Q3UBI6\|Q3UBI6_MOUSE | 68.32 | 9 | 9 | 2.71E+06 | 3 | 3 | 4 | Acetylation (Protein N-term); Deamidation (NQ); Oxidation (M) | 31351 | Uncharacterized protein OS=Mus musculus OX=10090 GN=Rpl7 PE=2 SV=1 |
| 64 | 196 | sp\|P14148\|RL7_MOUSE | 68.32 | 9 | 9 | 2.71E+06 | 3 | 3 | 4 | Acetylation (Protein N-term); Deamidation (NQ); Oxidation (M) | 31420 | 60S ribosomal protein L7 OS=Mus musculus OX=10090 GN=Rpl7 PE=1 SV=2 |
| 64 | 244 | Q3TK73\|Q3TK73_MOUSE | 68.32 | 9 | 9 | 2.71E+06 | 3 | 3 | 4 | Acetylation (Protein N-term); Deamidation (NQ); Oxidation (M) | 31390 | Uncharacterized protein OS=Mus musculus OX=10090 GN=Rpl7 PE=2 SV=1 |
| 64 | 197 | Q5M9N8\|Q5M9N8_MOUSE | 68.32 | 9 | 9 | 2.71E+06 | 3 | 3 | 4 | Acetylation (Protein N-term); Deamidation (NQ); Oxidation (M) | 31420 | Ribosomal protein L7 OS=Mus musculus OX=10090 GN=Rpl7 PE=1 SV=1 |
| 60 | 235 | Q8C1Y3\|Q8C1Y3_MOUSE | 67.91 | 15 | 15 | 4.80E+06 | 3 | 3 | 4 |  | 19253 | H15 domain-containing protein OS=Mus musculus OX=10090 GN=H1f0 PE=2 SV=1 |
| 60 | 225 | Q3U4Y0\|Q3U4Y0_MOUSE | 67.91 | 14 | 14 | 4.80E+06 | 3 | 3 | 4 |  | 20847 | H15 domain-containing protein OS=Mus musculus OX=10090 GN=H1f0 PE=2 SV=1 |
| 60 | 204 | sp\|P10922\|H10_MOUSE | 67.91 | 14 | 14 | 4.80E+06 | 3 | 3 | 4 |  | 20861 | Histone H1.0 OS=Mus musculus OX=10090 GN=H1-0 PE=2 SV=4 |
| 46 | 385 | G3UX26\|G3UX26_MOUSE | 66.65 | 8 | 8 | 2.76E+06 | 4 | 2 | 6 |  | 30446 | Voltage-dependent anion-selective channel protein 2 OS=Mus musculus OX=10090 GN=Vdac2 PE=1 SV=1 |
| 46 | 386 | sp\|Q60930\|VDAC2_MOUSE | 66.65 | 7 | 7 | 2.76E+06 | 4 | 2 | 6 |  | 31733 | Voltage-dependent anion-selective channel protein 2 OS=Mus musculus OX=10090 GN=Vdac2 PE=1 SV=2 |
| 46 | 411 | A0A286YCR8\|A0A286YCR8_MOUSE | 66.65 | 9 | 9 | 2.76E+06 | 4 | 2 | 6 |  | 26741 | Voltage-dependent anion-selective channel protein 2 (Fragment) OS=Mus musculus OX=10090 GN=Vdac2 PE=1 SV=1 |
| 66 | 191 | Q3UFI4\|Q3UFI4_MOUSE | 65.23 | 8 | 8 | 2.31E+06 | 3 | 3 | 3 |  | 33529 | 60S ribosomal protein L6 OS=Mus musculus OX=10090 GN=Rpl6 PE=2 SV=1 |
| 66 | 192 | sp\|P47911\|RL6_MOUSE | 65.23 | 8 | 8 | 2.31E+06 | 3 | 3 | 3 |  | 33510 | 60S ribosomal protein L6 OS=Mus musculus OX=10090 GN=Rpl6 PE=1 SV=3 |
| 66 | 193 | Q3UCH0\|Q3UCH0_MOUSE | 65.23 | 8 | 8 | 2.31E+06 | 3 | 3 | 3 |  | 33510 | 60S ribosomal protein L6 OS=Mus musculus OX=10090 GN=Rpl6 PE=1 SV=1 |
| 77 | 369 | B7ZP22\|B7ZP22_MOUSE | 63.17 | 7 | 7 | 1.92E+06 | 2 | 2 | 2 | Pyro-glu from Q | 35965 | Heterogeneous nuclear ribonucleoprotein A2/B1 OS=Mus musculus OX=10090 GN=Hnrnpa2b1 PE=2 SV=1 |
| 77 | 370 | sp\|O88569\|ROA2_MOUSE | 63.17 | 7 | 7 | 1.92E+06 | 2 | 2 | 2 | Pyro-glu from Q | 37403 | Heterogeneous nuclear ribonucleoproteins A2/B1 OS=Mus musculus OX=10090 GN=Hnrnpa2b1 PE=1 SV=2 |
| 63 | 232 | sp\|P17182\|ENOA_MOUSE | 60.57 | 7 | 7 | 2.70E+06 | 4 | 4 | 4 |  | 47141 | Alpha-enolase OS=Mus musculus OX=10090 GN=Eno1 PE=1 SV=3 |
| 63 | 233 | Q5FW97\|Q5FW97_MOUSE | 60.57 | 7 | 7 | 2.70E+06 | 4 | 4 | 4 |  | 47141 | Enolase 1 alpha non-neuron OS=Mus musculus OX=10090 GN=EG433182 PE=1 SV=1 |
| 101 | 659 | A0A0R4J049\|A0A0R4J049_MOUSE | 58.08 | 4 | 4 | 6.65E+05 | 1 | 1 | 1 | PRMT5 | 72738 | Protein arginine N-methyltransferase 5 OS=Mus musculus OX=10090 GN=Prmt5 PE=1 SV=1 |
| 101 | 643 | sp\|Q8CIG8\|ANM5_MOUSE | 58.08 | 4 | 4 | 6.65E+05 | 1 | 1 | 1 |  | 72680 | Protein arginine N-methyltransferase 5 OS=Mus musculus OX=10090 GN=Prmt5 PE=1 SV=3 |
| 52 | 336 | Q3UB63\|Q3UB63_MOUSE | 58.01 | 8 | 8 | 8.52E+06 | 3 | 3 | 5 |  | 39645 | Uncharacterized protein OS=Mus musculus OX=10090 GN=Slc25a3 PE=2 SV=1 |
| 52 | 259 | Q3THU8\|Q3THU8_MOUSE | 58.01 | 8 | 8 | 8.52E+06 | 3 | 3 | 5 |  | 39613 | Uncharacterized protein OS=Mus musculus OX=10090 GN=Slc25a3 PE=2 SV=1 |
| 52 | 260 | Q3U995\|Q3U995_MOUSE | 58.01 | 8 | 8 | 8.52E+06 | 3 | 3 | 5 |  | 39633 | Uncharacterized protein OS=Mus musculus OX=10090 GN=Slc25a3 PE=2 SV=1 |
| 52 | 261 | sp\|Q8VEM8\|MPCP_MOUSE | 58.01 | 8 | 8 | 8.52E+06 | 3 | 3 | 5 |  | 39632 | Phosphate carrier protein mitochondrial OS=Mus musculus OX=10090 GN=Slc25a3 PE=1 SV=1 |
| 52 | 205 | G5E902\|G5E902_MOUSE | 58.01 | 8 | 8 | 8.52E+06 | 3 | 3 | 5 |  | 39736 | MCG10343 isoform CRA_b OS=Mus musculus OX=10090 GN=Slc25a3 PE=1 SV=1 |
| 114 | 442 | sp\|Q8BMF4\|ODP2_MOUSE | 57.77 | 2 | 2 | 4.91E+05 | 1 | 1 | 1 |  | 67942 | Dihydrolipoyllysine-residue acetyltransferase component of pyruvate dehydrogenase complex mitochondrial OS=Mus musculus OX=10090 GN=Dlat PE=1 SV=2 |
| 79 | 485 | Q9D8W6\|Q9D8W6_MOUSE | 57.68 | 8 | 8 | 1.66E+06 | 2 | 2 | 2 |  | 21194 | GTP:AMP phosphotransferase AK3 mitochondrial OS=Mus musculus OX=10090 GN=Ak3 PE=2 SV=1 |
| 79 | 488 | sp\|Q9WTP7\|KAD3_MOUSE | 57.68 | 7 | 7 | 1.66E+06 | 2 | 2 | 2 |  | 25426 | GTP:AMP phosphotransferase AK3 mitochondrial OS=Mus musculus OX=10090 GN=Ak3 PE=1 SV=3 |
| 56 | 337 | A0JLV3\|A0JLV3_MOUSE | 56.11 | 22 | 22 | 7.97E+06 | 3 | 3 | 5 | Pyro-glu from Q | 13579 | Histone H2B (Fragment) OS=Mus musculus OX=10090 GN=Hist1h2bj PE=2 SV=1 |
| 56 | 344 | sp\|Q64478\|H2B1H_MOUSE | 56.11 | 21 | 21 | 7.97E+06 | 3 | 3 | 5 | Pyro-glu from Q | 13920 | Histone H2B type 1-H OS=Mus musculus OX=10090 GN=Hist1h2bh PE=1 SV=3 |
| 56 | 338 | B2RVD5\|B2RVD5_MOUSE | 56.11 | 21 | 21 | 7.97E+06 | 3 | 3 | 5 | Pyro-glu from Q | 13920 | Histone H2B OS=Mus musculus OX=10090 GN=H2bc12 PE=2 SV=1 |
| 56 | 339 | B2RTK3\|B2RTK3_MOUSE | 56.11 | 21 | 21 | 7.97E+06 | 3 | 3 | 5 | Pyro-glu from Q | 13936 | Histone H2B OS=Mus musculus OX=10090 GN=H2bc14 PE=1 SV=1 |
| 56 | 340 | sp\|P10854\|H2B1M_MOUSE | 56.11 | 21 | 21 | 7.97E+06 | 3 | 3 | 5 | Pyro-glu from Q | 13936 | Histone H2B type 1-M OS=Mus musculus OX=10090 GN=H2bc14 PE=1 SV=2 |
| 56 | 341 | sp\|Q6ZWY9\|H2B1C_MOUSE | 56.11 | 21 | 21 | 7.97E+06 | 3 | 3 | 5 | Pyro-glu from Q | 13906 | Histone H2B type 1-C/E/G OS=Mus musculus OX=10090 GN=H2bc4 PE=1 SV=3 |
| 56 | 342 | sp\|Q64475\|H2B1B_MOUSE | 56.11 | 21 | 21 | 7.97E+06 | 3 | 3 | 5 | Pyro-glu from Q | 13952 | Histone H2B type 1-B OS=Mus musculus OX=10090 GN=Hist1h2bb PE=1 SV=3 |
| 56 | 345 | sp\|Q64525\|H2B2B_MOUSE | 56.11 | 21 | 21 | 7.97E+06 | 3 | 3 | 5 | Pyro-glu from Q | 13920 | Histone H2B type 2-B OS=Mus musculus OX=10090 GN=Hist2h2bb PE=1 SV=3 |
| 56 | 346 | sp\|P10853\|H2B1F_MOUSE | 56.11 | 21 | 21 | 7.97E+06 | 3 | 3 | 5 | Pyro-glu from Q | 13936 | Histone H2B type 1-F/J/L OS=Mus musculus OX=10090 GN=H2bc7 PE=1 SV=2 |
| 56 | 347 | sp\|Q8CGP1\|H2B1K_MOUSE | 56.11 | 21 | 21 | 7.97E+06 | 3 | 3 | 5 | Pyro-glu from Q | 13920 | Histone H2B type 1-K OS=Mus musculus OX=10090 GN=H2bc12 PE=1 SV=3 |
| 56 | 343 | sp\|Q8CGP2\|H2B1P_MOUSE | 56.11 | 21 | 21 | 7.97E+06 | 3 | 3 | 5 | Pyro-glu from Q | 13992 | Histone H2B type 1-P OS=Mus musculus OX=10090 GN=Hist1h2bp PE=1 SV=3 |
| 56 | 349 | A0JNS9\|A0JNS9_MOUSE | 56.11 | 21 | 21 | 7.97E+06 | 3 | 3 | 5 | Pyro-glu from Q | 14179 | Histone H2B OS=Mus musculus OX=10090 GN=H2bc1 PE=2 SV=1 |
| 56 | 348 | sp\|P70696\|H2B1A_MOUSE | 56.11 | 21 | 21 | 7.97E+06 | 3 | 3 | 5 | Pyro-glu from Q | 14237 | Histone H2B type 1-A OS=Mus musculus OX=10090 GN=H2bc1 PE=1 SV=3 |
| 56 | 350 | Q8CBB6\|Q8CBB6_MOUSE | 56.11 | 20 | 20 | 7.97E+06 | 3 | 3 | 5 | Pyro-glu from Q | 14888 | Histone H2B OS=Mus musculus OX=10090 GN=Hist1h2bq PE=2 SV=1 |
| 56 | 351 | Q921L4\|Q921L4_MOUSE | 56.11 | 20 | 20 | 7.97E+06 | 3 | 3 | 5 | Pyro-glu from Q | 14939 | Histone H2B OS=Mus musculus OX=10090 GN=LOC665622 PE=2 SV=1 |
| 78 | 495 | H3BKD0\|H3BKD0_MOUSE | 53.92 | 8 | 8 | 1.04E+06 | 2 | 2 | 2 | hnRNPK | 33198 | Heterogeneous nuclear ribonucleoprotein K (Fragment) OS=Mus musculus OX=10090 GN=Hnrnpk PE=1 SV=1 |
| 78 | 452 | B2M1R6\|B2M1R6_MOUSE | 53.92 | 6 | 6 | 1.04E+06 | 2 | 2 | 2 |  | 48562 | Heterogeneous nuclear ribonucleoprotein K OS=Mus musculus OX=10090 GN=Hnrnpk PE=1 SV=1 |
| 78 | 453 | Q3U9Q3\|Q3U9Q3_MOUSE | 53.92 | 6 | 6 | 1.04E+06 | 2 | 2 | 2 |  | 49880 | Uncharacterized protein OS=Mus musculus OX=10090 GN=Hnrnpk PE=2 SV=1 |
| 78 | 454 | Q3U6X2\|Q3U6X2_MOUSE | 53.92 | 5 | 5 | 1.04E+06 | 2 | 2 | 2 |  | 51063 | Uncharacterized protein OS=Mus musculus OX=10090 GN=Hnrnpk PE=2 SV=1 |
| 78 | 456 | Q3TJ38\|Q3TJ38_MOUSE | 53.92 | 5 | 5 | 1.04E+06 | 2 | 2 | 2 |  | 51018 | Uncharacterized protein OS=Mus musculus OX=10090 GN=Hnrnpk PE=2 SV=1 |
| 78 | 457 | sp\|P61979\|HNRPK_MOUSE | 53.92 | 5 | 5 | 1.04E+06 | 2 | 2 | 2 |  | 50976 | Heterogeneous nuclear ribonucleoprotein K OS=Mus musculus OX=10090 GN=Hnrnpk PE=1 SV=1 |
| 78 | 455 | Q3TUA1\|Q3TUA1_MOUSE | 53.92 | 5 | 5 | 1.04E+06 | 2 | 2 | 2 |  | 51006 | Uncharacterized protein OS=Mus musculus OX=10090 GN=Hnrnpk PE=2 SV=1 |
| 78 | 459 | Q5FWJ5\|Q5FWJ5_MOUSE | 53.92 | 5 | 5 | 1.04E+06 | 2 | 2 | 2 |  | 51028 | Hnrpk protein OS=Mus musculus OX=10090 GN=Hnrnpk PE=2 SV=1 |
| 78 | 458 | Q3TL71\|Q3TL71_MOUSE | 53.92 | 5 | 5 | 1.04E+06 | 2 | 2 | 2 |  | 51008 | Uncharacterized protein OS=Mus musculus OX=10090 GN=Hnrnpk PE=2 SV=1 |
| 82 | 467 | S4R1N6\|S4R1N6_MOUSE | 52.11 | 19 | 19 | 2.17E+06 | 2 | 2 | 2 |  | 12483 | 40S ribosomal protein S18 OS=Mus musculus OX=10090 GN=Rps18 PE=3 SV=1 |
| 82 | 469 | Q3TW65\|Q3TW65_MOUSE | 52.11 | 13 | 13 | 2.17E+06 | 2 | 2 | 2 |  | 17747 | Uncharacterized protein OS=Mus musculus OX=10090 GN=Rps18 PE=2 SV=1 |
| 82 | 470 | Q561N5\|Q561N5_MOUSE | 52.11 | 13 | 13 | 2.17E+06 | 2 | 2 | 2 |  | 17719 | MCG23000 isoform CRA_b OS=Mus musculus OX=10090 GN=Rps18 PE=2 SV=1 |
| 82 | 471 | sp\|P62270\|RS18_MOUSE | 52.11 | 13 | 13 | 2.17E+06 | 2 | 2 | 2 |  | 17719 | 40S ribosomal protein S18 OS=Mus musculus OX=10090 GN=Rps18 PE=1 SV=3 |
| 82 | 472 | F6YVP7\|F6YVP7_MOUSE | 52.11 | 13 | 13 | 2.17E+06 | 2 | 2 | 2 |  | 17672 | Predicted gene 10260 OS=Mus musculus OX=10090 GN=Gm10260 PE=3 SV=2 |
| 82 | 468 | A0A1Y7VKY1\|A0A1Y7VKY1_MOUSE | 52.11 | 13 | 13 | 2.17E+06 | 2 | 2 | 2 |  | 17749 | MCG116671 OS=Mus musculus OX=10090 GN=Rps18-ps5 PE=3 SV=1 |
| 75 | 449 | sp\|P62900\|RL31_MOUSE | 50.44 | 14 | 14 | 2.34E+06 | 2 | 2 | 2 |  | 14463 | 60S ribosomal protein L31 OS=Mus musculus OX=10090 GN=Rpl31 PE=1 SV=1 |
| 75 | 448 | Q5M9K9\|Q5M9K9_MOUSE | 50.44 | 14 | 14 | 2.34E+06 | 2 | 2 | 2 |  | 14463 | MCG126194 isoform CRA_a OS=Mus musculus OX=10090 GN=Rpl31 PE=1 SV=1 |
| 75 | 450 | Q9CY93\|Q9CY93_MOUSE | 50.44 | 14 | 14 | 2.34E+06 | 2 | 2 | 2 |  | 14411 | Uncharacterized protein OS=Mus musculus OX=10090 GN=Rpl31 PE=2 SV=1 |
| 75 | 502 | A0A0A6YXL3\|A0A0A6YXL3_MOUSE | 50.44 | 20 | 20 | 2.34E+06 | 2 | 2 | 2 |  | 9942 | 60S ribosomal protein L31 OS=Mus musculus OX=10090 GN=Rpl31 PE=1 SV=1 |
| 75 | 503 | A0A0A6YX26\|A0A0A6YX26_MOUSE | 50.44 | 13 | 13 | 2.34E+06 | 2 | 2 | 2 |  | 14997 | 60S ribosomal protein L31 OS=Mus musculus OX=10090 GN=Rpl31 PE=1 SV=1 |
| 80 | 504 | sp\|Q62425\|NDUA4_MOUSE | 49.64 | 18 | 18 | 2.05E+06 | 2 | 2 | 2 |  | 9327 | Cytochrome c oxidase subunit NDUFA4 OS=Mus musculus OX=10090 GN=Ndufa4 PE=1 SV=2 |
| 104 | 710 | sp\|P06329\|HVM50_MOUSE | 49.07 | 21 | 21 | 1.61E+06 | 1 | 1 | 1 | Carbamidomethylation; Oxidation (M) | 13311 | Ig heavy chain V region AC38 15.3 OS=Mus musculus OX=10090 PE=1 SV=1 |
| 68 | 314 | Q3TI47\|Q3TI47_MOUSE | 49.02 | 3 | 3 | 2.54E+06 | 2 | 1 | 2 |  | 72346 | Uncharacterized protein OS=Mus musculus OX=10090 GN=Hspa5 PE=2 SV=1 |
| 70 | 518 | sp\|P00405\|COX2_MOUSE | 48.1 | 6 | 6 | 8.05E+06 | 2 | 2 | 3 |  | 25976 | Cytochrome c oxidase subunit 2 OS=Mus musculus OX=10090 GN=Mtco2 PE=1 SV=1 |
| 70 | 509 | A3E4B0\|A3E4B0_MOUSE | 48.1 | 6 | 6 | 8.05E+06 | 2 | 2 | 3 |  | 25946 | Cytochrome c oxidase subunit 2 OS=Mus musculus musculus OX=39442 GN=COX2 PE=3 SV=1 |
| 70 | 519 | A0A0F6PXF3\|A0A0F6PXF3_MOUSE | 48.1 | 6 | 6 | 8.05E+06 | 2 | 2 | 3 |  | 25976 | Cytochrome c oxidase subunit 2 OS=Mus musculus helgolandicus OX=1643390 GN=COXII PE=3 SV=1 |
| 70 | 510 | A3R481\|A3R481_MOUSE | 48.1 | 6 | 6 | 8.05E+06 | 2 | 2 | 3 |  | 25975 | Cytochrome c oxidase subunit 2 OS=Mus musculus domesticus OX=10092 GN=COXII PE=3 SV=1 |
| 70 | 511 | A3R455\|A3R455_MUSMC | 48.1 | 6 | 6 | 8.05E+06 | 2 | 2 | 3 |  | 25976 | Cytochrome c oxidase subunit 2 OS=Mus musculus castaneus OX=10091 GN=COXII PE=3 SV=1 |
| 70 | 520 | Q5GA81\|Q5GA81_MUSMM | 48.1 | 6 | 6 | 8.05E+06 | 2 | 2 | 3 |  | 25976 | Cytochrome c oxidase subunit 2 OS=Mus musculus molossinus OX=57486 GN=COX2 PE=3 SV=1 |
| 70 | 512 | Q7JCZ1\|Q7JCZ1_MOUSE | 48.1 | 6 | 6 | 8.05E+06 | 2 | 2 | 3 |  | 25976 | Cytochrome c oxidase subunit 2 OS=Mus musculus OX=10090 GN=mt-Co2 PE=1 SV=1 |
| 70 | 521 | A0A023J6F3\|A0A023J6F3_MOUSE | 48.1 | 6 | 6 | 8.05E+06 | 2 | 2 | 3 |  | 25976 | Cytochrome c oxidase subunit 2 OS=Mus musculus musculus OX=39442 GN=COX2 PE=3 SV=1 |
| 70 | 513 | K7XK22\|K7XK22_MOUSE | 48.1 | 6 | 6 | 8.05E+06 | 2 | 2 | 3 |  | 25990 | Cytochrome c oxidase subunit 2 OS=Mus musculus domesticus OX=10092 GN=COXII PE=3 SV=1 |
| 70 | 514 | K7XKA7\|K7XKA7_MOUSE | 48.1 | 6 | 6 | 8.05E+06 | 2 | 2 | 3 |  | 25962 | Cytochrome c oxidase subunit 2 OS=Mus musculus domesticus OX=10092 GN=COXII PE=3 SV=1 |
| 70 | 515 | A0A023J6I7\|A0A023J6I7_MUSMC | 48.1 | 6 | 6 | 8.05E+06 | 2 | 2 | 3 |  | 25990 | Cytochrome c oxidase subunit 2 OS=Mus musculus castaneus OX=10091 GN=COX2 PE=3 SV=1 |
| 70 | 516 | Q7JD03\|Q7JD03_MOUSE | 48.1 | 6 | 6 | 8.05E+06 | 2 | 2 | 3 |  | 25976 | Cytochrome c oxidase subunit 2 OS=Mus musculus domesticus OX=10092 GN=COX2 PE=3 SV=1 |
| 70 | 517 | A0A075DC90\|A0A075DC90_MOUSE | 48.1 | 6 | 6 | 8.05E+06 | 2 | 2 | 3 |  | 26003 | Cytochrome c oxidase subunit 2 OS=Mus musculus OX=10090 GN=COX2 PE=3 SV=1 |
| 67 | 365 | A0A4U9FFL2\|A0A4U9FFL2_MOUSE | 47.71 | 13 | 13 | 3.14E+07 | 2 | 1 | 3 | Carbamidomethylation; Deamidation (NQ); Oxidation (M) | 35634 | IgG1 (Fragment) OS=Mus musculus OX=10090 GN=Ighg1 PE=4 SV=1 |
| 67 | 366 | A0A075B5P4\|A0A075B5P4_MOUSE | 47.71 | 13 | 13 | 3.14E+07 | 2 | 1 | 3 | Carbamidomethylation; Deamidation (NQ); Oxidation (M) | 35752 | Ig gamma-1 chain C region secreted form (Fragment) OS=Mus musculus OX=10090 GN=Ighg1 PE=1 SV=1 |
| 67 | 367 | A0A0A6YWR2\|A0A0A6YWR2_MOUSE | 47.71 | 11 | 11 | 3.14E+07 | 2 | 1 | 3 | Carbamidomethylation; Deamidation (NQ); Oxidation (M) | 43434 | Ig gamma-1 chain C region secreted form (Fragment) OS=Mus musculus OX=10090 GN=Ighg1 PE=1 SV=1 |
| 67 | 356 | A0A0B6VMB2\|A0A0B6VMB2_MOUSE | 47.71 | 9 | 9 | 3.14E+07 | 2 | 1 | 3 | Carbamidomethylation; Deamidation (NQ); Oxidation (M) | 50725 | MAb 31C6 heavy chain OS=Mus musculus OX=10090 GN=HC PE=4 SV=1 |
| 67 | 368 | A0A0C6E3V3\|A0A0C6E3V3_MOUSE | 47.71 | 9 | 9 | 3.14E+07 | 2 | 1 | 3 | Carbamidomethylation; Deamidation (NQ); Oxidation (M) | 51022 | HC protein OS=Mus musculus OX=10090 GN=HC PE=2 SV=1 |
| 115 | 689 | Q6NWV5\|Q6NWV5_MOUSE | 47.22 | 5 | 5 | 7.85E+05 | 1 | 1 | 1 |  | 22415 | Pgam1 protein (Fragment) OS=Mus musculus OX=10090 GN=Pgam1 PE=2 SV=1 |
| 115 | 691 | Q5NCI4\|Q5NCI4_MOUSE | 47.22 | 4 | 4 | 7.85E+05 | 1 | 1 | 1 |  | 28827 | Phosphoglycerate mutase OS=Mus musculus OX=10090 GN=Pgam2 PE=1 SV=1 |
| 115 | 692 | sp\|O70250\|PGAM2_MOUSE | 47.22 | 4 | 4 | 7.85E+05 | 1 | 1 | 1 |  | 28827 | Phosphoglycerate mutase 2 OS=Mus musculus OX=10090 GN=Pgam2 PE=1 SV=3 |
| 115 | 694 | Q3U7Z6\|Q3U7Z6_MOUSE | 47.22 | 4 | 4 | 7.85E+05 | 1 | 1 | 1 |  | 28832 | Phosphoglycerate mutase OS=Mus musculus OX=10090 GN=Pgam1 PE=1 SV=1 |
| 115 | 693 | sp\|Q9DBJ1\|PGAM1_MOUSE | 47.22 | 4 | 4 | 7.85E+05 | 1 | 1 | 1 |  | 28832 | Phosphoglycerate mutase 1 OS=Mus musculus OX=10090 GN=Pgam1 PE=1 SV=3 |
| 71 | 145 | A0A0A6YWC8\|A0A0A6YWC8_MOUSE | 47.09 | 4 | 4 | 4.77E+05 | 2 | 1 | 3 |  | 49193 | Vimentin OS=Mus musculus OX=10090 GN=Vim PE=1 SV=1 |
| 71 | 147 | Q3TWV0\|Q3TWV0_MOUSE | 47.09 | 3 | 3 | 4.77E+05 | 2 | 1 | 3 |  | 53666 | IF rod domain-containing protein OS=Mus musculus OX=10090 GN=Vim PE=2 SV=1 |
| 71 | 148 | Q3UAX1\|Q3UAX1_MOUSE | 47.09 | 3 | 3 | 4.77E+05 | 2 | 1 | 3 |  | 53558 | IF rod domain-containing protein OS=Mus musculus OX=10090 GN=Vim PE=2 SV=1 |
| 71 | 149 | sp\|P20152\|VIME_MOUSE | 47.09 | 3 | 3 | 4.77E+05 | 2 | 1 | 3 |  | 53688 | Vimentin OS=Mus musculus OX=10090 GN=Vim PE=1 SV=3 |
| 71 | 150 | Q5FWJ3\|Q5FWJ3_MOUSE | 47.09 | 3 | 3 | 4.77E+05 | 2 | 1 | 3 |  | 53688 | Vimentin OS=Mus musculus OX=10090 GN=Vim PE=1 SV=1 |
| 71 | 151 | Q3TFD9\|Q3TFD9_MOUSE | 47.09 | 3 | 3 | 4.77E+05 | 2 | 1 | 3 |  | 53689 | IF rod domain-containing protein OS=Mus musculus OX=10090 GN=Vim PE=2 SV=1 |
| 71 | 152 | Q3U6S1\|Q3U6S1_MOUSE | 47.09 | 3 | 3 | 4.77E+05 | 2 | 1 | 3 |  | 53674 | IF rod domain-containing protein OS=Mus musculus OX=10090 GN=Vim PE=2 SV=1 |
| 71 | 146 | Q3V2S4\|Q3V2S4_MOUSE | 47.09 | 3 | 3 | 4.77E+05 | 2 | 1 | 3 |  | 53748 | IF rod domain-containing protein OS=Mus musculus OX=10090 GN=Vim PE=2 SV=1 |
| 131 | 727 | sp\|O55143\|AT2A2_MOUSE | 46.02 | 1 | 1 | 8.23E+05 | 1 | 1 | 1 | Acetylation (Protein N-term) | 114858 | Sarcoplasmic/endoplasmic reticulum calcium ATPase 2 OS=Mus musculus OX=10090 GN=Atp2a2 PE=1 SV=2 |
| 131 | 738 | Q5DTI2\|Q5DTI2_MOUSE | 46.02 | 1 | 1 | 8.23E+05 | 1 | 1 | 1 | Acetylation (Protein N-term) | 116600 | ATPase Ca++ transporting cardiac muscle slow twitch 2 isoform CRA_b (Fragment) OS=Mus musculus OX=10090 GN=Atp2a2 PE=2 SV=1 |
| 116 | 696 | D3YV25\|D3YV25_MOUSE | 44.85 | 22 | 22 | 8.43E+05 | 1 | 1 | 1 |  | 5319 | ADP-ribosylation factor 3 (Fragment) OS=Mus musculus OX=10090 GN=Arf3 PE=4 SV=1 |
| 116 | 697 | E9Q2C2\|E9Q2C2_MOUSE | 44.85 | 17 | 17 | 8.43E+05 | 1 | 1 | 1 |  | 7229 | ADP-ribosylation factor 4 OS=Mus musculus OX=10090 GN=Arf4 PE=4 SV=1 |
| 116 | 549 | Q14BR4\|Q14BR4_MOUSE | 44.85 | 6 | 6 | 8.43E+05 | 1 | 1 | 1 |  | 20397 | ADP-ribosylation factor 4 OS=Mus musculus OX=10090 GN=Arf4 PE=1 SV=1 |
| 116 | 698 | sp\|P84084\|ARF5_MOUSE | 44.85 | 6 | 6 | 8.43E+05 | 1 | 1 | 1 |  | 20530 | ADP-ribosylation factor 5 OS=Mus musculus OX=10090 GN=Arf5 PE=1 SV=2 |
| 116 | 548 | sp\|P61750\|ARF4_MOUSE | 44.85 | 6 | 6 | 8.43E+05 | 1 | 1 | 1 |  | 20397 | ADP-ribosylation factor 4 OS=Mus musculus OX=10090 GN=Arf4 PE=1 SV=2 |
| 116 | 699 | Q9DD04\|Q9DD04_MOUSE | 44.85 | 6 | 6 | 8.43E+05 | 1 | 1 | 1 |  | 20478 | Uncharacterized protein OS=Mus musculus OX=10090 GN=Arf4 PE=2 SV=1 |
| 116 | 700 | sp\|Q8BSL7\|ARF2_MOUSE | 44.85 | 6 | 6 | 8.43E+05 | 1 | 1 | 1 |  | 20746 | ADP-ribosylation factor 2 OS=Mus musculus OX=10090 GN=Arf2 PE=1 SV=2 |
| 116 | 701 | sp\|P61205\|ARF3_MOUSE | 44.85 | 6 | 6 | 8.43E+05 | 1 | 1 | 1 |  | 20601 | ADP-ribosylation factor 3 OS=Mus musculus OX=10090 GN=Arf3 PE=2 SV=2 |
| 116 | 702 | Q3U344\|Q3U344_MOUSE | 44.85 | 6 | 6 | 8.43E+05 | 1 | 1 | 1 |  | 20601 | ADP-ribosylation factor 3 OS=Mus musculus OX=10090 GN=Arf3 PE=2 SV=1 |
| 116 | 703 | sp\|P84078\|ARF1_MOUSE | 44.85 | 6 | 6 | 8.43E+05 | 1 | 1 | 1 |  | 20697 | ADP-ribosylation factor 1 OS=Mus musculus OX=10090 GN=Arf1 PE=1 SV=2 |
| 83 | 6030 | Q3TKR5\|Q3TKR5_MOUSE | 44.48 | 4 | 4 | 2.44E+06 | 2 | 2 | 2 |  | 34269 | Ribosomal protein L5 OS=Mus musculus OX=10090 GN=Rpl5 PE=2 SV=1 |
| 83 | 9663 | sp\|P47962\|RL5_MOUSE | 44.48 | 4 | 4 | 2.44E+06 | 2 | 2 | 2 |  | 34401 | 60S ribosomal protein L5 OS=Mus musculus OX=10090 GN=Rpl5 PE=1 SV=3 |
| 83 | 9664 | Q58EU6\|Q58EU6_MOUSE | 44.48 | 4 | 4 | 2.44E+06 | 2 | 2 | 2 |  | 34401 | MCG13589 OS=Mus musculus OX=10090 GN=Rpl5 PE=1 SV=1 |
| 117 | 714 | Q3UIC3\|Q3UIC3_MOUSE | 44.16 | 6 | 6 | 5.42E+05 | 1 | 1 | 1 |  | 18632 | Ldh_1_C domain-containing protein (Fragment) OS=Mus musculus OX=10090 GN=Ldha PE=2 SV=1 |
| 117 | 715 | Q99K20\|Q99K20_MOUSE | 44.16 | 3 | 3 | 5.42E+05 | 1 | 1 | 1 |  | 34503 | L-lactate dehydrogenase (Fragment) OS=Mus musculus OX=10090 GN=Ldha PE=2 SV=1 |
| 117 | 716 | A0A1B0GSR9\|A0A1B0GSR9_MOUSE | 44.16 | 3 | 3 | 5.42E+05 | 1 | 1 | 1 |  | 34599 | L-lactate dehydrogenase OS=Mus musculus OX=10090 GN=Ldha PE=1 SV=1 |
| 117 | 717 | Q3UDU4\|Q3UDU4_MOUSE | 44.16 | 3 | 3 | 5.42E+05 | 1 | 1 | 1 |  | 36265 | L-lactate dehydrogenase OS=Mus musculus OX=10090 GN=Ldha PE=2 SV=1 |
| 117 | 718 | Q3TCI7\|Q3TCI7_MOUSE | 44.16 | 3 | 3 | 5.42E+05 | 1 | 1 | 1 |  | 36500 | L-lactate dehydrogenase OS=Mus musculus OX=10090 GN=Ldha PE=2 SV=1 |
| 117 | 719 | Q564E2\|Q564E2_MOUSE | 44.16 | 3 | 3 | 5.42E+05 | 1 | 1 | 1 |  | 36499 | L-lactate dehydrogenase OS=Mus musculus OX=10090 GN=Ldha PE=1 SV=1 |
| 117 | 720 | Q3THB4\|Q3THB4_MOUSE | 44.16 | 3 | 3 | 5.42E+05 | 1 | 1 | 1 |  | 36500 | L-lactate dehydrogenase OS=Mus musculus OX=10090 GN=Ldha PE=2 SV=1 |
| 117 | 721 | sp\|P06151\|LDHA_MOUSE | 44.16 | 3 | 3 | 5.42E+05 | 1 | 1 | 1 |  | 36499 | L-lactate dehydrogenase A chain OS=Mus musculus OX=10090 GN=Ldha PE=1 SV=3 |
| 117 | 722 | Q3TI99\|Q3TI99_MOUSE | 44.16 | 3 | 3 | 5.42E+05 | 1 | 1 | 1 |  | 36485 | L-lactate dehydrogenase OS=Mus musculus OX=10090 GN=Ldha PE=2 SV=1 |
| 117 | 723 | A0A1B0GSX0\|A0A1B0GSX0_MOUSE | 44.16 | 3 | 3 | 5.42E+05 | 1 | 1 | 1 |  | 39758 | L-lactate dehydrogenase OS=Mus musculus OX=10090 GN=Ldha PE=1 SV=1 |
| 81 | 229 | H7BXC3\|H7BXC3_MOUSE | 44.11 | 11 | 11 | 1.65E+06 | 2 | 2 | 2 |  | 17997 | Triosephosphate isomerase OS=Mus musculus OX=10090 GN=Tpi1 PE=1 SV=1 |
| 81 | 178 | sp\|P17751\|TPIS_MOUSE | 44.11 | 6 | 6 | 1.65E+06 | 2 | 2 | 2 |  | 32192 | Triosephosphate isomerase OS=Mus musculus OX=10090 GN=Tpi1 PE=1 SV=4 |
| 118 | 613 | Q3UJS0\|Q3UJS0_MOUSE | 42.98 | 4 | 4 | 1.72E+06 | 1 | 1 | 1 |  | 28067 | Ribosomal_L2_C domain-containing protein OS=Mus musculus OX=10090 GN=Rpl8 PE=2 SV=1 |
| 118 | 614 | sp\|P62918\|RL8_MOUSE | 42.98 | 4 | 4 | 1.72E+06 | 1 | 1 | 1 |  | 28025 | 60S ribosomal protein L8 OS=Mus musculus OX=10090 GN=Rpl8 PE=1 SV=2 |
| 121 | 753 | sp\|P63038\|CH60_MOUSE | 42.18 | 2 | 2 | 6.57E+05 | 1 | 1 | 1 |  | 60956 | 60 kDa heat shock protein mitochondrial OS=Mus musculus OX=10090 GN=Hspd1 PE=1 SV=1 |
| 119 | 599 | A0A1B0GSL5\|A0A1B0GSL5_MOUSE | 42.09 | 10 | 10 | 4.43E+06 | 1 | 1 | 1 | Acetylation (Protein N-term) | 11804 | 60S ribosomal protein L13a OS=Mus musculus OX=10090 GN=Rpl13a PE=1 SV=1 |
| 119 | 600 | A0A1B0GSC2\|A0A1B0GSC2_MOUSE | 42.09 | 9 | 9 | 4.43E+06 | 1 | 1 | 1 | Acetylation (Protein N-term) | 13960 | 60S ribosomal protein L13a (Fragment) OS=Mus musculus OX=10090 GN=Rpl13a PE=1 SV=1 |
| 119 | 601 | A0A1B0GRH1\|A0A1B0GRH1_MOUSE | 42.09 | 9 | 9 | 4.43E+06 | 1 | 1 | 1 | Acetylation (Protein N-term) | 14330 | 60S ribosomal protein L13a OS=Mus musculus OX=10090 GN=Rpl13a PE=1 SV=1 |
| 119 | 602 | A0A1B0GSB2\|A0A1B0GSB2_MOUSE | 42.09 | 7 | 7 | 4.43E+06 | 1 | 1 | 1 | Acetylation (Protein N-term) | 16450 | 60S ribosomal protein L13a OS=Mus musculus OX=10090 GN=Rpl13a PE=1 SV=1 |
| 119 | 603 | Q3TDS9\|Q3TDS9_MOUSE | 42.09 | 6 | 6 | 4.43E+06 | 1 | 1 | 1 | Acetylation (Protein N-term) | 20052 | Uncharacterized protein OS=Mus musculus OX=10090 GN=Rpl13a PE=2 SV=1 |
| 119 | 604 | Q5M9M0\|Q5M9M0_MOUSE | 42.09 | 5 | 5 | 4.43E+06 | 1 | 1 | 1 | Acetylation (Protein N-term) | 23464 | MCG23455 isoform CRA_e OS=Mus musculus OX=10090 GN=Rpl13a PE=1 SV=1 |
| 119 | 605 | sp\|P19253\|RL13A_MOUSE | 42.09 | 5 | 5 | 4.43E+06 | 1 | 1 | 1 | Acetylation (Protein N-term) | 23464 | 60S ribosomal protein L13a OS=Mus musculus OX=10090 GN=Rpl13a PE=1 SV=4 |
| 76 | 557 | Q3TEY5\|Q3TEY5_MOUSE | 41.8 | 2 | 2 | 1.52E+06 | 2 | 2 | 2 |  | 75629 | MICOS complex subunit MIC60 OS=Mus musculus OX=10090 GN=Immt PE=2 SV=1 |
| 76 | 558 | E9Q800\|E9Q800_MOUSE | 41.8 | 2 | 2 | 1.52E+06 | 2 | 2 | 2 |  | 75601 | MICOS complex subunit MIC60 OS=Mus musculus OX=10090 GN=Immt PE=1 SV=1 |
| 76 | 568 | Q3U7N2\|Q3U7N2_MOUSE | 41.8 | 2 | 2 | 1.52E+06 | 2 | 2 | 2 |  | 82513 | MICOS complex subunit MIC60 OS=Mus musculus OX=10090 GN=Immt PE=2 SV=1 |
| 76 | 569 | Q3TVZ5\|Q3TVZ5_MOUSE | 41.8 | 2 | 2 | 1.52E+06 | 2 | 2 | 2 |  | 82416 | MICOS complex subunit MIC60 OS=Mus musculus OX=10090 GN=Immt PE=2 SV=1 |
| 76 | 571 | sp\|Q8CAQ8\|MIC60_MOUSE | 41.8 | 2 | 2 | 1.52E+06 | 2 | 2 | 2 |  | 83900 | MICOS complex subunit Mic60 OS=Mus musculus OX=10090 GN=Immt PE=1 SV=1 |
| 76 | 545 | A6H604\|A6H604_MOUSE | 41.8 | 6 | 6 | 1.52E+06 | 2 | 2 | 2 |  | 30454 | MICOS complex subunit MIC60 (Fragment) OS=Mus musculus OX=10090 GN=Immt PE=2 SV=1 |
| 76 | 546 | A0A0U1RQ14\|A0A0U1RQ14_MOUSE | 41.8 | 5 | 5 | 1.52E+06 | 2 | 2 | 2 |  | 33001 | MICOS complex subunit MIC60 (Fragment) OS=Mus musculus OX=10090 GN=Immt PE=1 SV=1 |
| 76 | 547 | Q6P8Y5\|Q6P8Y5_MOUSE | 41.8 | 5 | 5 | 1.52E+06 | 2 | 2 | 2 |  | 34103 | MICOS complex subunit MIC60 (Fragment) OS=Mus musculus OX=10090 GN=Immt PE=2 SV=1 |
| 76 | 551 | Q2YDW0\|Q2YDW0_MOUSE | 41.8 | 3 | 3 | 1.52E+06 | 2 | 2 | 2 |  | 50156 | MICOS complex subunit MIC60 (Fragment) OS=Mus musculus OX=10090 GN=Immt PE=2 SV=1 |
| 76 | 556 | Q3TQL4\|Q3TQL4_MOUSE | 41.8 | 2 | 2 | 1.52E+06 | 2 | 2 | 2 |  | 75473 | MICOS complex subunit MIC60 (Fragment) OS=Mus musculus OX=10090 GN=Immt PE=2 SV=1 |
| 120 | 711 | B1AV14\|B1AV14_MOUSE | 41.69 | 6 | 6 | 2.10E+05 | 1 | 1 | 1 |  | 21063 | MICOS complex subunit OS=Mus musculus OX=10090 GN=Apool PE=1 SV=1 |
| 120 | 712 | sp\|Q78IK4\|MIC27_MOUSE | 41.69 | 4 | 4 | 2.10E+05 | 1 | 1 | 1 |  | 29261 | MICOS complex subunit Mic27 OS=Mus musculus OX=10090 GN=Apool PE=1 SV=1 |
| 120 | 713 | Q9CZB4\|Q9CZB4_MOUSE | 41.69 | 4 | 4 | 2.10E+05 | 1 | 1 | 1 |  | 29329 | MICOS complex subunit OS=Mus musculus OX=10090 GN=Apool PE=2 SV=1 |
| 123 | 688 | E0CZA1\|E0CZA1_MOUSE | 40.63 | 5 | 5 | 4.27E+05 | 1 | 1 | 1 |  | 21526 | T-complex protein 1 subunit epsilon (Fragment) OS=Mus musculus OX=10090 GN=Cct5 PE=1 SV=1 |
| 123 | 695 | sp\|P80316\|TCPE_MOUSE | 40.63 | 2 | 2 | 4.27E+05 | 1 | 1 | 1 |  | 59624 | T-complex protein 1 subunit epsilon OS=Mus musculus OX=10090 GN=Cct5 PE=1 SV=1 |
| 122 | 745 | G3UY29\|G3UY29_MOUSE | 40.27 | 7 | 7 | 6.01E+05 | 1 | 1 | 1 |  | 16924 | MCG22989 isoform CRA_a OS=Mus musculus OX=10090 GN=Rab11b PE=4 SV=1 |
| 122 | 746 | E9Q3P9\|E9Q3P9_MOUSE | 40.27 | 7 | 7 | 6.01E+05 | 1 | 1 | 1 |  | 17375 | Ras-related protein Rab-11A OS=Mus musculus OX=10090 GN=Rab11a PE=4 SV=1 |
| 122 | 747 | F8WGS1\|F8WGS1_MOUSE | 40.27 | 7 | 7 | 6.01E+05 | 1 | 1 | 1 |  | 17668 | Ras-related protein Rab-11A (Fragment) OS=Mus musculus OX=10090 GN=Rab11a PE=4 SV=1 |
| 122 | 748 | sp\|P62492\|RB11A_MOUSE | 40.27 | 5 | 5 | 6.01E+05 | 1 | 1 | 1 |  | 24394 | Ras-related protein Rab-11A OS=Mus musculus OX=10090 GN=Rab11a PE=1 SV=3 |
| 122 | 749 | Q0PD45\|Q0PD45_MOUSE | 40.27 | 5 | 5 | 6.01E+05 | 1 | 1 | 1 |  | 24394 | RAB11a member RAS oncogene family OS=Mus musculus OX=10090 GN=Rab11a PE=1 SV=1 |
| 122 | 750 | A0A068BFR3\|A0A068BFR3_MOUSE | 40.27 | 5 | 5 | 6.01E+05 | 1 | 1 | 1 |  | 24488 | RAS oncogene family protein OS=Mus musculus OX=10090 GN=Rab11b PE=2 SV=1 |
| 122 | 751 | Q78ZJ8\|Q78ZJ8_MOUSE | 40.27 | 5 | 5 | 6.01E+05 | 1 | 1 | 1 |  | 24489 | MCG22989 isoform CRA_b OS=Mus musculus OX=10090 GN=Rab11b PE=1 SV=1 |
| 122 | 752 | sp\|P46638\|RB11B_MOUSE | 40.27 | 5 | 5 | 6.01E+05 | 1 | 1 | 1 |  | 24489 | Ras-related protein Rab-11B OS=Mus musculus OX=10090 GN=Rab11b PE=1 SV=3 |
| 84 | 394 | A1L0U3\|A1L0U3_MOUSE | 39.96 | 10 | 10 | 1.29E+06 | 2 | 2 | 2 |  | 15030 | Histone H3 (Fragment) OS=Mus musculus OX=10090 GN=Hist1h3e PE=2 SV=1 |
| 84 | 395 | F8WI35\|F8WI35_MOUSE | 39.96 | 10 | 10 | 1.29E+06 | 2 | 2 | 2 |  | 15199 | Histone H3 OS=Mus musculus OX=10090 GN=H3f3a PE=1 SV=1 |
| 84 | 396 | A1L0V4\|A1L0V4_MOUSE | 39.96 | 10 | 10 | 1.29E+06 | 2 | 2 | 2 |  | 15273 | Histone H3 (Fragment) OS=Mus musculus OX=10090 GN=Hist1h3i PE=2 SV=1 |
| 84 | 397 | B9EI85\|B9EI85_MOUSE | 39.96 | 10 | 10 | 1.29E+06 | 2 | 2 | 2 |  | 15388 | Histone H3 OS=Mus musculus OX=10090 GN=Hist2h3b PE=2 SV=1 |
| 84 | 398 | sp\|P68433\|H31_MOUSE | 39.96 | 10 | 10 | 1.29E+06 | 2 | 2 | 2 |  | 15404 | Histone H3.1 OS=Mus musculus OX=10090 GN=H3c1 PE=1 SV=2 |
| 84 | 400 | sp\|P84244\|H33_MOUSE | 39.96 | 10 | 10 | 1.29E+06 | 2 | 2 | 2 |  | 15328 | Histone H3.3 OS=Mus musculus OX=10090 GN=H3-3a PE=1 SV=2 |
| 84 | 401 | sp\|P84228\|H32_MOUSE | 39.96 | 10 | 10 | 1.29E+06 | 2 | 2 | 2 |  | 15388 | Histone H3.2 OS=Mus musculus OX=10090 GN=Hist1h3b PE=1 SV=2 |
| 84 | 399 | sp\|P02301\|H3C_MOUSE | 39.96 | 10 | 10 | 1.29E+06 | 2 | 2 | 2 |  | 15315 | Histone H3.3C OS=Mus musculus OX=10090 GN=H3f3c PE=3 SV=3 |
| 84 | 403 | A0A1W2P768\|A0A1W2P768_MOUSE | 39.96 | 7 | 7 | 1.29E+06 | 2 | 2 | 2 |  | 20247 | H3 clustered histone 14 OS=Mus musculus OX=10090 GN=H3c14 PE=1 SV=1 |
| 100 | 606 | F7C106\|F7C106_MOUSE | 39.66 | 9 | 9 | 7.73E+05 | 1 | 1 | 1 |  | 9014 | Cytochrome c oxidase subunit 5B mitochondrial OS=Mus musculus OX=10090 GN=Cox5b PE=1 SV=2 |
| 100 | 607 | A0A0A6YVR0\|A0A0A6YVR0_MOUSE | 39.66 | 8 | 8 | 7.73E+05 | 1 | 1 | 1 |  | 10336 | Cytochrome c oxidase subunit 5B mitochondrial (Fragment) OS=Mus musculus OX=10090 GN=Cox5b PE=1 SV=1 |
| 100 | 608 | sp\|P19536\|COX5B_MOUSE | 39.66 | 6 | 6 | 7.73E+05 | 1 | 1 | 1 |  | 13813 | Cytochrome c oxidase subunit 5B mitochondrial OS=Mus musculus OX=10090 GN=Cox5b PE=1 SV=1 |
| 100 | 609 | Q9D881\|Q9D881_MOUSE | 39.66 | 6 | 6 | 7.73E+05 | 1 | 1 | 1 |  | 13847 | Cytochrome c oxidase subunit 5B mitochondrial OS=Mus musculus OX=10090 GN=Gm11273 PE=1 SV=1 |
| 124 | 586 | Q5SW86\|Q5SW86_MOUSE | 39.54 | 10 | 10 | 3.99E+05 | 1 | 1 | 1 |  | 13598 | RAB1A member RAS oncogene family OS=Mus musculus OX=10090 GN=Rab1a PE=1 SV=1 |
| 124 | 587 | Q5SW87\|Q5SW87_MOUSE | 39.54 | 9 | 9 | 3.99E+05 | 1 | 1 | 1 |  | 15025 | RAB1A member RAS oncogene family OS=Mus musculus OX=10090 GN=Rab1a PE=1 SV=1 |
| 124 | 588 | Q5SW88\|Q5SW88_MOUSE | 39.54 | 6 | 6 | 3.99E+05 | 1 | 1 | 1 |  | 22372 | RAB1A member RAS oncogene family OS=Mus musculus OX=10090 GN=Rab1a PE=1 SV=1 |
| 124 | 590 | Q3UB66\|Q3UB66_MOUSE | 39.54 | 6 | 6 | 3.99E+05 | 1 | 1 | 1 |  | 22679 | Uncharacterized protein OS=Mus musculus OX=10090 GN=Rab1a PE=2 SV=1 |
| 124 | 591 | Q0PD67\|Q0PD67_MOUSE | 39.54 | 6 | 6 | 3.99E+05 | 1 | 1 | 1 |  | 22678 | RAB1 member RAS oncogene family isoform CRA_a OS=Mus musculus OX=10090 GN=Rab1a PE=1 SV=1 |
| 124 | 589 | sp\|P62821\|RAB1A_MOUSE | 39.54 | 6 | 6 | 3.99E+05 | 1 | 1 | 1 |  | 22678 | Ras-related protein Rab-1A OS=Mus musculus OX=10090 GN=Rab1A PE=1 SV=3 |
| 124 | 592 | Q6ZPF0\|Q6ZPF0_MOUSE | 39.54 | 5 | 5 | 3.99E+05 | 1 | 1 | 1 |  | 27335 | MKIAA3012 protein (Fragment) OS=Mus musculus OX=10090 GN=Rab1a PE=2 SV=1 |
| 125 | 578 | Q4VAG4\|Q4VAG4_MOUSE | 38.19 | 10 | 10 | 1.58E+06 | 1 | 1 | 1 |  | 14759 | MCG12304 OS=Mus musculus OX=10090 GN=Rpl22 PE=1 SV=1 |
| 125 | 579 | sp\|P67984\|RL22_MOUSE | 38.19 | 10 | 10 | 1.58E+06 | 1 | 1 | 1 |  | 14759 | 60S ribosomal protein L22 OS=Mus musculus OX=10090 GN=Rpl22 PE=1 SV=2 |
| 128 | 809 | A0A494BBD8\|A0A494BBD8_MOUSE | 37.38 | 3 | 3 | 1.23E+06 | 1 | 1 | 1 |  | 33945 | Annexin OS=Mus musculus OX=10090 GN=Anxa1 PE=1 SV=1 |
| 126 | 760 | Q8R4A4\|Q8R4A4_MOUSE | 37.24 | 5 | 5 | 7.31E+05 | 1 | 1 | 1 |  | 21920 | Cell cycle p34 CDC2 kinase protein (Fragment) OS=Mus musculus OX=10090 PE=4 SV=1 |
| 126 | 761 | D3Z2T9\|D3Z2T9_MOUSE | 37.24 | 5 | 5 | 7.31E+05 | 1 | 1 | 1 |  | 22894 | Cyclin-dependent kinase 1 (Fragment) OS=Mus musculus OX=10090 GN=Cdk1 PE=1 SV=1 |
| 126 | 762 | Q99JW7\|Q99JW7_MOUSE | 37.24 | 3 | 3 | 7.31E+05 | 1 | 1 | 1 |  | 33832 | Cdc2a protein (Fragment) OS=Mus musculus OX=10090 GN=Cdk1 PE=2 SV=1 |
| 126 | 763 | sp\|P11440\|CDK1_MOUSE | 37.24 | 3 | 3 | 7.31E+05 | 1 | 1 | 1 |  | 34107 | Cyclin-dependent kinase 1 OS=Mus musculus OX=10090 GN=Cdk1 PE=1 SV=3 |
| 127 | 741 | B1ARA3\|B1ARA3_MOUSE | 37.18 | 8 | 8 | 3.91E+05 | 1 | 1 | 1 |  | 12217 | 60S ribosomal protein L26 (Fragment) OS=Mus musculus OX=10090 GN=Rpl26 PE=1 SV=1 |
| 127 | 742 | Q3U7N1\|Q3U7N1_MOUSE | 37.18 | 6 | 6 | 3.91E+05 | 1 | 1 | 1 |  | 17259 | KOW domain-containing protein OS=Mus musculus OX=10090 PE=2 SV=1 |
| 127 | 743 | Q4FZH2\|Q4FZH2_MOUSE | 37.18 | 6 | 6 | 3.91E+05 | 1 | 1 | 1 |  | 17258 | MCG50660 isoform CRA_a OS=Mus musculus OX=10090 GN=Rpl26 PE=1 SV=1 |
| 127 | 744 | sp\|P61255\|RL26_MOUSE | 37.18 | 6 | 6 | 3.91E+05 | 1 | 1 | 1 |  | 17258 | 60S ribosomal protein L26 OS=Mus musculus OX=10090 GN=Rpl26 PE=1 SV=1 |
| 103 | 464 | Q91YK6\|Q91YK6_MOUSE | 36.78 | 9 | 9 | 1.33E+06 | 1 | 1 | 1 |  | 16940 | Rpl23a protein (Fragment) OS=Mus musculus OX=10090 GN=Rpl23a PE=2 SV=1 |
| 103 | 465 | Q4V9X9\|Q4V9X9_MOUSE | 36.78 | 8 | 8 | 1.33E+06 | 1 | 1 | 1 |  | 17564 | Rpl23a protein (Fragment) OS=Mus musculus OX=10090 GN=Rpl23a PE=2 SV=1 |
| 103 | 447 | sp\|P62751\|RL23A_MOUSE | 36.78 | 8 | 8 | 1.33E+06 | 1 | 1 | 1 |  | 17695 | 60S ribosomal protein L23a OS=Mus musculus OX=10090 GN=Rpl23a PE=1 SV=1 |
| 103 | 466 | Q5M9M5\|Q5M9M5_MOUSE | 36.78 | 8 | 8 | 1.33E+06 | 1 | 1 | 1 |  | 17695 | MCG10806 OS=Mus musculus OX=10090 GN=Rpl23a PE=1 SV=1 |
| 44 | 731 | A0A075B5U5\|A0A075B5U5_MOUSE | 36.16 | 21 | 21 | 1.99E+07 | 1 | 1 | 5 | Carbamidomethylation | 12857 | Immunoglobulin heavy variable V1-19 OS=Mus musculus OX=10090 GN=Ighv1-19 PE=1 SV=1 |
| 44 | 730 | A0A075B5V3\|A0A075B5V3_MOUSE | 36.16 | 21 | 21 | 1.99E+07 | 1 | 1 | 5 | Carbamidomethylation | 12904 | Immunoglobulin heavy variable 1-36 OS=Mus musculus OX=10090 GN=Ighv1-36 PE=4 SV=1 |
| 44 | 732 | A0A0A6YXT2\|A0A0A6YXT2_MOUSE | 36.16 | 21 | 21 | 1.99E+07 | 1 | 1 | 5 | Carbamidomethylation | 12961 | Immunoglobulin heavy variable 1-36 (Fragment) OS=Mus musculus OX=10090 GN=Ighv1-36 PE=4 SV=1 |
| 44 | 733 | A0A0A6YWX0\|A0A0A6YWX0_MOUSE | 36.16 | 21 | 21 | 1.99E+07 | 1 | 1 | 5 | Carbamidomethylation | 12914 | Immunoglobulin heavy variable V1-19 (Fragment) OS=Mus musculus OX=10090 GN=Ighv1-19 PE=1 SV=1 |
| 51 | 9674 | O70286\|O70286_MOUSE | 36 | 4 | 4 | 2.20E+07 | 2 | 1 | 5 |  | 23905 | Cyclic AMP specific phosphodiesterase PDE4D5A (Fragment) OS=Mus musculus OX=10090 GN=Pde4d PE=2 SV=1 |
| 51 | 6059 | F6QFD1\|F6QFD1_MOUSE | 36 | 1 | 1 | 2.20E+07 | 2 | 1 | 5 |  | 85560 | Phosphodiesterase (Fragment) OS=Mus musculus OX=10090 GN=Pde4d PE=1 SV=1 |
| 102 | 493 | Q3ULW0\|Q3ULW0_MOUSE | 35.27 | 5 | 5 | 5.44E+05 | 1 | 1 | 1 |  | 24351 | GTP-binding nuclear protein Ran OS=Mus musculus OX=10090 GN=Ran PE=2 SV=1 |
| 102 | 494 | sp\|P62827\|RAN_MOUSE | 35.27 | 5 | 5 | 5.44E+05 | 1 | 1 | 1 |  | 24423 | GTP-binding nuclear protein Ran OS=Mus musculus OX=10090 GN=Ran PE=1 SV=3 |
| 102 | 709 | Q14AA6\|Q14AA6_MOUSE | 35.27 | 5 | 5 | 5.44E+05 | 1 | 1 | 1 |  | 24357 | GTP-binding nuclear protein Ran OS=Mus musculus OX=10090 GN=1700009N14Rik PE=2 SV=1 |
| 85 | 767 | G3UWL7\|G3UWL7_MOUSE | 34.43 | 10 | 10 | 3.28E+06 | 1 | 1 | 2 |  | 9118 | Histone H2A OS=Mus musculus OX=10090 GN=H2az1 PE=1 SV=1 |
| 85 | 768 | Q3UA95\|Q3UA95_MOUSE | 34.43 | 9 | 9 | 3.28E+06 | 1 | 1 | 2 |  | 10984 | Histone H2A OS=Mus musculus OX=10090 GN=H2az1 PE=2 SV=1 |
| 85 | 769 | A0A0N4SV66\|A0A0N4SV66_MOUSE | 34.43 | 7 | 7 | 3.28E+06 | 1 | 1 | 2 |  | 13660 | Histone H2A OS=Mus musculus OX=10090 GN=H2aj PE=1 SV=1 |
| 85 | 770 | A0AUV1\|A0AUV1_MOUSE | 34.43 | 7 | 7 | 3.28E+06 | 1 | 1 | 2 |  | 13819 | Histone H2A (Fragment) OS=Mus musculus OX=10090 GN=H2ac12 PE=2 SV=1 |
| 85 | 771 | sp\|Q3THW5\|H2AV_MOUSE | 34.43 | 7 | 7 | 3.28E+06 | 1 | 1 | 2 |  | 13509 | Histone H2A.V OS=Mus musculus OX=10090 GN=H2afv PE=1 SV=3 |
| 85 | 775 | sp\|P0C0S6\|H2AZ_MOUSE | 34.43 | 7 | 7 | 3.28E+06 | 1 | 1 | 2 |  | 13553 | Histone H2A.Z OS=Mus musculus OX=10090 GN=H2az1 PE=1 SV=2 |
| 85 | 772 | sp\|Q8CGP6\|H2A1H_MOUSE | 34.43 | 7 | 7 | 3.28E+06 | 1 | 1 | 2 |  | 13950 | Histone H2A type 1-H OS=Mus musculus OX=10090 GN=Hist1h2ah PE=1 SV=3 |
| 85 | 773 | A3KPD0\|A3KPD0_MOUSE | 34.43 | 7 | 7 | 3.28E+06 | 1 | 1 | 2 |  | 13950 | Histone H2A OS=Mus musculus OX=10090 GN=H2ac12 PE=2 SV=1 |
| 85 | 774 | B2RVP5\|B2RVP5_MOUSE | 34.43 | 7 | 7 | 3.28E+06 | 1 | 1 | 2 |  | 13509 | Histone H2A OS=Mus musculus OX=10090 GN=H2az2 PE=2 SV=1 |
| 85 | 776 | Q149V4\|Q149V4_MOUSE | 34.43 | 7 | 7 | 3.28E+06 | 1 | 1 | 2 |  | 13988 | Histone H2A OS=Mus musculus OX=10090 GN=H2ac20 PE=2 SV=1 |
| 85 | 777 | Q8CGP4\|Q8CGP4_MOUSE | 34.43 | 7 | 7 | 3.28E+06 | 1 | 1 | 2 |  | 14056 | Histone H2A OS=Mus musculus OX=10090 GN=H2ac1 PE=1 SV=1 |
| 85 | 778 | sp\|Q64523\|H2A2C_MOUSE | 34.43 | 7 | 7 | 3.28E+06 | 1 | 1 | 2 |  | 13988 | Histone H2A type 2-C OS=Mus musculus OX=10090 GN=Hist2h2ac PE=1 SV=3 |
| 85 | 779 | B2RWH3\|B2RWH3_MOUSE | 34.43 | 7 | 7 | 3.28E+06 | 1 | 1 | 2 |  | 14095 | Histone H2A OS=Mus musculus OX=10090 GN=Hist2h2aa1 PE=2 SV=1 |
| 85 | 780 | sp\|C0HKE1\|H2A1B_MOUSE | 34.43 | 7 | 7 | 3.28E+06 | 1 | 1 | 2 |  | 14135 | Histone H2A type 1-B OS=Mus musculus OX=10090 GN=H2ac4 PE=1 SV=1 |
| 85 | 781 | sp\|Q6GSS7\|H2A2A_MOUSE | 34.43 | 7 | 7 | 3.28E+06 | 1 | 1 | 2 |  | 14095 | Histone H2A type 2-A OS=Mus musculus OX=10090 GN=Hist2h2aa1 PE=1 SV=3 |
| 85 | 782 | sp\|Q8BFU2\|H2A3_MOUSE | 34.43 | 7 | 7 | 3.28E+06 | 1 | 1 | 2 |  | 14121 | Histone H2A type 3 OS=Mus musculus OX=10090 GN=Hist3h2a PE=1 SV=3 |
| 85 | 788 | B2RVF0\|B2RVF0_MOUSE | 34.43 | 7 | 7 | 3.28E+06 | 1 | 1 | 2 |  | 14135 | Histone H2A OS=Mus musculus OX=10090 GN=Hist1h2ad PE=2 SV=1 |
| 85 | 789 | sp\|C0HKE5\|H2A1G_MOUSE | 34.43 | 7 | 7 | 3.28E+06 | 1 | 1 | 2 |  | 14135 | Histone H2A type 1-G OS=Mus musculus OX=10090 GN=H2ac11 PE=1 SV=1 |
| 85 | 783 | sp\|C0HKE9\|H2A1P_MOUSE | 34.43 | 7 | 7 | 3.28E+06 | 1 | 1 | 2 |  | 14135 | Histone H2A type 1-P OS=Mus musculus OX=10090 GN=Hist1h2ap PE=1 SV=1 |
| 85 | 790 | sp\|C0HKE4\|H2A1E_MOUSE | 34.43 | 7 | 7 | 3.28E+06 | 1 | 1 | 2 |  | 14135 | Histone H2A type 1-E OS=Mus musculus OX=10090 GN=H2ac8 PE=1 SV=1 |
| 85 | 791 | sp\|Q8CGP7\|H2A1K_MOUSE | 34.43 | 7 | 7 | 3.28E+06 | 1 | 1 | 2 |  | 14150 | Histone H2A type 1-K OS=Mus musculus OX=10090 GN=H2ac15 PE=1 SV=3 |
| 85 | 784 | A2AB79\|A2AB79_MOUSE | 34.43 | 7 | 7 | 3.28E+06 | 1 | 1 | 2 |  | 14121 | Histone H2A OS=Mus musculus OX=10090 GN=H2aw PE=2 SV=1 |
| 85 | 785 | sp\|C0HKE7\|H2A1N_MOUSE | 34.43 | 7 | 7 | 3.28E+06 | 1 | 1 | 2 |  | 14135 | Histone H2A type 1-N OS=Mus musculus OX=10090 GN=Hist1h2an PE=1 SV=1 |
| 85 | 786 | sp\|Q64522\|H2A2B_MOUSE | 34.43 | 7 | 7 | 3.28E+06 | 1 | 1 | 2 |  | 14013 | Histone H2A type 2-B OS=Mus musculus OX=10090 GN=Hist2h2ab PE=1 SV=3 |
| 85 | 792 | sp\|C0HKE2\|H2A1C_MOUSE | 34.43 | 7 | 7 | 3.28E+06 | 1 | 1 | 2 |  | 14135 | Histone H2A type 1-C OS=Mus musculus OX=10090 GN=Hist1h2ac PE=1 SV=1 |
| 85 | 787 | sp\|C0HKE6\|H2A1I_MOUSE | 34.43 | 7 | 7 | 3.28E+06 | 1 | 1 | 2 |  | 14135 | Histone H2A type 1-I OS=Mus musculus OX=10090 GN=H2ac13 PE=1 SV=1 |
| 85 | 793 | sp\|C0HKE3\|H2A1D_MOUSE | 34.43 | 7 | 7 | 3.28E+06 | 1 | 1 | 2 |  | 14135 | Histone H2A type 1-D OS=Mus musculus OX=10090 GN=H2ac7 PE=1 SV=1 |
| 85 | 794 | sp\|C0HKE8\|H2A1O_MOUSE | 34.43 | 7 | 7 | 3.28E+06 | 1 | 1 | 2 |  | 14135 | Histone H2A type 1-O OS=Mus musculus OX=10090 GN=Hist1h2ao PE=1 SV=1 |
| 85 | 795 | Q64426\|Q64426_MOUSE | 34.43 | 7 | 7 | 3.28E+06 | 1 | 1 | 2 |  | 14746 | Histone H2A (Fragment) OS=Mus musculus domesticus OX=10092 GN=H2A PE=2 SV=1 |
| 85 | 796 | sp\|P27661\|H2AX_MOUSE | 34.43 | 6 | 6 | 3.28E+06 | 1 | 1 | 2 |  | 15143 | Histone H2AX OS=Mus musculus OX=10090 GN=H2afx PE=1 SV=2 |
| 129 | 828 | A0A087WS46\|A0A087WS46_MOUSE | 34.12 | 4 | 4 | 9.17E+05 | 1 | 1 | 1 |  | 20137 | Eukaryotic translation elongation factor 1 beta 2 OS=Mus musculus OX=10090 GN=Eef1b2 PE=1 SV=1 |
| 129 | 837 | sp\|O70251\|EF1B_MOUSE | 34.12 | 3 | 3 | 9.17E+05 | 1 | 1 | 1 |  | 24694 | Elongation factor 1-beta OS=Mus musculus OX=10090 GN=Eef1b PE=1 SV=5 |
| 130 | 740 | sp\|P14152\|MDHC_MOUSE | 32.32 | 3 | 3 | 9.10E+05 | 1 | 1 | 1 |  | 36511 | Malate dehydrogenase cytoplasmic OS=Mus musculus OX=10090 GN=Mdh1 PE=1 SV=3 |
| 74 | 873 | A2AQD6\|A2AQD6_MOUSE | 31.5 | 1 | 1 | 3.97E+06 | 1 | 1 | 2 |  | 133462 | Protein ITPRID2 OS=Mus musculus OX=10090 GN=Itprid2 PE=1 SV=1 |
| 74 | 875 | sp\|Q922B9\|ITPI2_MOUSE | 31.5 | 1 | 1 | 3.97E+06 | 1 | 1 | 2 |  | 136947 | Protein ITPRID2 OS=Mus musculus OX=10090 GN=Itprid2 PE=1 SV=3 |
| 74 | 874 | A2AQD5\|A2AQD5_MOUSE | 31.5 | 1 | 1 | 3.97E+06 | 1 | 1 | 2 |  | 134671 | Protein ITPRID2 OS=Mus musculus OX=10090 GN=Itprid2 PE=1 SV=1 |
| 132 | 418 | sp\|P12970\|RL7A_MOUSE | 31 | 3 | 3 | 7.61E+05 | 1 | 1 | 1 |  | 29977 | 60S ribosomal protein L7a OS=Mus musculus OX=10090 GN=Rpl7a PE=1 SV=2 |
| 132 | 417 | Q58ET1\|Q58ET1_MOUSE | 31 | 3 | 3 | 7.61E+05 | 1 | 1 | 1 |  | 29977 | MCG11348 OS=Mus musculus OX=10090 GN=Rpl7a PE=1 SV=1 |
| 132 | 419 | Q6P1A9\|Q6P1A9_MOUSE | 31 | 3 | 3 | 7.61E+05 | 1 | 1 | 1 |  | 30025 | Ribosomal protein L7A OS=Mus musculus OX=10090 GN=Rpl7a PE=2 SV=1 |
| 132 | 451 | Q5EBG5\|Q5EBG5_MOUSE | 31 | 3 | 3 | 7.61E+05 | 1 | 1 | 1 |  | 29905 | Ribosomal protein L7A OS=Mus musculus OX=10090 GN=Rpl7a PE=2 SV=1 |
| 132 | 420 | Q80UT7\|Q80UT7_MOUSE | 31 | 3 | 3 | 7.61E+05 | 1 | 1 | 1 |  | 30474 | Rpl7a protein (Fragment) OS=Mus musculus OX=10090 GN=Rpl7a PE=2 SV=1 |
| 133 | 544 | sp\|Q9WUM5\|SUCA_MOUSE | 30.84 | 3 | 3 | 6.84E+05 | 1 | 1 | 1 |  | 36155 | Succinate--CoA ligase [ADP/GDP-forming] subunit alpha mitochondrial OS=Mus musculus OX=10090 GN=Suclg1 PE=1 SV=4 |
| 134 | 633 | A5JUZ1\|A5JUZ1_MOUSE | 27.53 | 12 | 12 | 7.46E+05 | 1 | 1 | 1 |  | 8565 | Ubiqutin subunit 1 (Fragment) OS=Mus musculus OX=10090 GN=Ubc PE=2 SV=1 |
| 134 | 9671 | A0A0A6YW67\|A0A0A6YW67_MOUSE | 27.53 | 12 | 12 | 7.46E+05 | 1 | 1 | 1 |  | 8728 | MCG23377 isoform CRA_b OS=Mus musculus OX=10090 GN=Gm8797 PE=4 SV=1 |
| 134 | 9672 | Q5M9K3\|Q5M9K3_MOUSE | 27.53 | 7 | 7 | 7.46E+05 | 1 | 1 | 1 |  | 14728 | MCG23116 isoform CRA_a OS=Mus musculus OX=10090 GN=Uba52 PE=2 SV=1 |
| 134 | 634 | Q642L7\|Q642L7_MOUSE | 27.53 | 6 | 6 | 7.46E+05 | 1 | 1 | 1 |  | 17951 | MCG13441 OS=Mus musculus OX=10090 GN=Rps27a PE=2 SV=1 |
| 134 | 9673 | E9Q4P0\|E9Q4P0_MOUSE | 27.53 | 5 | 5 | 7.46E+05 | 1 | 1 | 1 |  | 22234 | KxDL motif-containing protein 1 (Fragment) OS=Mus musculus OX=10090 GN=Kxd1 PE=1 SV=1 |
| 134 | 635 | E9QNP0\|E9QNP0_MOUSE | 27.53 | 4 | 4 | 7.46E+05 | 1 | 1 | 1 |  | 26856 | KxDL motif-containing protein 1 OS=Mus musculus OX=10090 GN=Kxd1 PE=1 SV=1 |
| 134 | 636 | Q3TH47\|Q3TH47_MOUSE | 27.53 | 3 | 3 | 7.46E+05 | 1 | 1 | 1 |  | 31269 | Uncharacterized protein OS=Mus musculus OX=10090 GN=Ubc PE=2 SV=1 |
| 134 | 637 | Q78XY9\|Q78XY9_MOUSE | 27.53 | 3 | 3 | 7.46E+05 | 1 | 1 | 1 |  | 34369 | MCG23377 isoform CRA_a OS=Mus musculus OX=10090 GN=Ubb PE=2 SV=1 |
| 134 | 638 | Q8R0Z9\|Q8R0Z9_MOUSE | 27.53 | 3 | 3 | 7.46E+05 | 1 | 1 | 1 |  | 39816 | Ubc protein OS=Mus musculus OX=10090 GN=Ubc PE=2 SV=1 |
| 134 | 640 | Q922Z8\|Q922Z8_MOUSE | 27.53 | 2 | 2 | 7.46E+05 | 1 | 1 | 1 |  | 65457 | Ubc protein OS=Mus musculus OX=10090 GN=Ubc PE=2 SV=1 |
| 134 | 641 | Q8VC46\|Q8VC46_MOUSE | 27.53 | 1 | 1 | 7.46E+05 | 1 | 1 | 1 |  | 74003 | Ubc protein OS=Mus musculus OX=10090 GN=Ubc PE=2 SV=1 |
| 43 | 1150 | F7DC05\|F7DC05_MOUSE | 27.5 | 4 | 4 | 9.38E+07 | 1 | 1 | 1 |  | 15397 | Myeloperoxidase (Fragment) OS=Mus musculus OX=10090 GN=Mpo PE=1 SV=1 |
| 43 | 1159 | Q571G0\|Q571G0_MOUSE | 27.5 | 1 | 1 | 9.38E+07 | 1 | 1 | 1 |  | 81111 | MKIAA4033 protein (Fragment) OS=Mus musculus OX=10090 GN=Mpo PE=2 SV=1 |
| 43 | 1160 | sp\|P11247\|PERM_MOUSE | 27.5 | 1 | 1 | 9.38E+07 | 1 | 1 | 1 |  | 81182 | Myeloperoxidase OS=Mus musculus OX=10090 GN=Mpo PE=1 SV=2 |
| 43 | 1161 | Q6RFG4\|Q6RFG4_MOUSE | 27.5 | 1 | 1 | 9.38E+07 | 1 | 1 | 1 |  | 81068 | Myeloperoxidase OS=Mus musculus OX=10090 GN=Mpo PE=2 SV=1 |
| 43 | 1162 | Q7TMS4\|Q7TMS4_MOUSE | 27.5 | 1 | 1 | 9.38E+07 | 1 | 1 | 1 |  | 81168 | Mpo protein OS=Mus musculus OX=10090 GN=Mpo PE=2 SV=1 |
| 135 | 974 | sp\|Q99KI0\|ACON_MOUSE | 26.66 | 1 | 1 | 8.86E+05 | 1 | 1 | 1 |  | 85464 | Aconitate hydratase mitochondrial OS=Mus musculus OX=10090 GN=Aco2 PE=1 SV=1 |
| 86 | 844 | Q99JL6\|Q99JL6_MOUSE | 26.49 | 6 | 6 | 1.20E+06 | 1 | 1 | 2 |  | 15108 | Ribosomal protein OS=Mus musculus OX=10090 GN=Rpl10a PE=2 SV=1 |
| 86 | 525 | A0A3B2WDD2\|A0A3B2WDD2_MOUSE | 26.49 | 4 | 4 | 1.20E+06 | 1 | 1 | 2 |  | 21637 | Ribosomal protein OS=Mus musculus OX=10090 GN=Rpl10a PE=1 SV=1 |
| 86 | 526 | A0A3B2WBL1\|A0A3B2WBL1_MOUSE | 26.49 | 4 | 4 | 1.20E+06 | 1 | 1 | 2 |  | 24744 | Ribosomal protein OS=Mus musculus OX=10090 GN=Rpl10a PE=1 SV=1 |
| 86 | 527 | sp\|P53026\|RL10A_MOUSE | 26.49 | 4 | 4 | 1.20E+06 | 1 | 1 | 2 |  | 24916 | 60S ribosomal protein L10a OS=Mus musculus OX=10090 GN=Rpl10a PE=1 SV=3 |
| 86 | 529 | Q5XJF6\|Q5XJF6_MOUSE | 26.49 | 4 | 4 | 1.20E+06 | 1 | 1 | 2 |  | 24831 | Ribosomal protein OS=Mus musculus OX=10090 GN=Rpl10a PE=1 SV=1 |
| 86 | 528 | Q3U561\|Q3U561_MOUSE | 26.49 | 4 | 4 | 1.20E+06 | 1 | 1 | 2 |  | 24815 | Ribosomal protein OS=Mus musculus OX=10090 GN=Rpl10a PE=2 SV=1 |
| 137 | 804 | A0A1L1SQA8\|A0A1L1SQA8_MOUSE | 26.06 | 11 | 11 | 1.06E+06 | 1 | 1 | 1 |  | 10309 | 40S ribosomal protein S25 OS=Mus musculus OX=10090 GN=Rps25 PE=1 SV=1 |
| 137 | 806 | sp\|P62852\|RS25_MOUSE | 26.06 | 8 | 8 | 1.06E+06 | 1 | 1 | 1 |  | 13742 | 40S ribosomal protein S25 OS=Mus musculus OX=10090 GN=Rps25 PE=1 SV=1 |
| 137 | 805 | Q58EA6\|Q58EA6_MOUSE | 26.06 | 8 | 8 | 1.06E+06 | 1 | 1 | 1 |  | 13742 | 40S ribosomal protein S25 OS=Mus musculus OX=10090 GN=Rps25 PE=1 SV=1 |
| 138 | 323 | sp\|Q9CPQ1\|COX6C_MOUSE | 25.96 | 16 | 16 | 8.15E+05 | 1 | 1 | 1 | Acetylation (Protein N-term) | 8469 | Cytochrome c oxidase subunit 6C OS=Mus musculus OX=10090 GN=Cox6c PE=1 SV=3 |
| 139 | 9679 | Q91WS8\|Q91WS8_MOUSE | 25.55 | 2 | 2 | 1.22E+06 | 1 | 1 | 1 |  | 46437 | Acyl-Coenzyme A dehydrogenase medium chain OS=Mus musculus OX=10090 GN=Acadm PE=2 SV=1 |
| 140 | 1038 | Q3U617\|Q3U617_MOUSE | 23.91 | 4 | 4 | 1.26E+06 | 1 | 1 | 1 |  | 21320 | PCI domain-containing protein OS=Mus musculus OX=10090 GN=Psmd8 PE=2 SV=1 |
| 140 | 1039 | Q3TG45\|Q3TG45_MOUSE | 23.91 | 3 | 3 | 1.26E+06 | 1 | 1 | 1 |  | 28526 | 26S proteasome non-ATPase regulatory subunit 8 OS=Mus musculus OX=10090 GN=Psmd8 PE=1 SV=1 |
| 140 | 1040 | Q99JB5\|Q99JB5_MOUSE | 23.91 | 3 | 3 | 1.26E+06 | 1 | 1 | 1 |  | 29939 | Proteasome (Prosome macropain) 26S subunit non-ATPase 8 OS=Mus musculus OX=10090 GN=Psmd8 PE=2 SV=1 |
| 140 | 6104 | Q9CPS5\|Q9CPS5_MOUSE | 23.91 | 3 | 3 | 1.26E+06 | 1 | 1 | 1 |  | 32769 | 26S proteasome non-ATPase regulatory subunit 8 OS=Mus musculus OX=10090 GN=Psmd8 PE=1 SV=2 |
| 140 | 6105 | Q8BKP5\|Q8BKP5_MOUSE | 23.91 | 3 | 3 | 1.26E+06 | 1 | 1 | 1 |  | 32709 | PCI domain-containing protein OS=Mus musculus OX=10090 GN=Psmd8 PE=2 SV=1 |
| 140 | 1042 | Q3TW90\|Q3TW90_MOUSE | 23.91 | 3 | 3 | 1.26E+06 | 1 | 1 | 1 |  | 32742 | PCI domain-containing protein OS=Mus musculus OX=10090 GN=Psmd8 PE=2 SV=1 |
| 140 | 1041 | Q3TI95\|Q3TI95_MOUSE | 23.91 | 3 | 3 | 1.26E+06 | 1 | 1 | 1 |  | 32678 | PCI domain-containing protein OS=Mus musculus OX=10090 GN=Psmd8 PE=2 SV=1 |
| 140 | 1043 | Q3TVY0\|Q3TVY0_MOUSE | 23.91 | 3 | 3 | 1.26E+06 | 1 | 1 | 1 |  | 34827 | PCI domain-containing protein OS=Mus musculus OX=10090 GN=Psmd8 PE=2 SV=1 |
| 140 | 1044 | sp\|Q9CX56\|PSMD8_MOUSE | 23.91 | 2 | 2 | 1.26E+06 | 1 | 1 | 1 |  | 39930 | 26S proteasome non-ATPase regulatory subunit 8 OS=Mus musculus OX=10090 GN=Psmd8 PE=1 SV=2 |
| 141 | 9681 | Q3MI66\|Q3MI66_MOUSE | 23.74 | 2 | 2 | 5.74E+06 | 1 | 1 | 1 |  | 35136 | Olfr700 protein (Fragment) OS=Mus musculus OX=10090 GN=Olfr700 PE=2 SV=1 |
| 141 | 9682 | Q9EPF6\|Q9EPF6_MOUSE | 23.74 | 2 | 2 | 5.74E+06 | 1 | 1 | 1 |  | 35120 | MCG1046656 OS=Mus musculus OX=10090 GN=Olfr704 PE=3 SV=1 |
| 141 | 9683 | K7N641\|K7N641_MOUSE | 23.74 | 2 | 2 | 5.74E+06 | 1 | 1 | 1 |  | 34932 | Olfactory receptor 694 OS=Mus musculus OX=10090 GN=Olfr694 PE=3 SV=1 |
| 141 | 9684 | Q8VF88\|Q8VF88_MOUSE | 23.74 | 2 | 2 | 5.74E+06 | 1 | 1 | 1 |  | 34928 | Olfactory receptor 694 OS=Mus musculus OX=10090 GN=Olfr694 PE=2 SV=1 |
| 141 | 9685 | Q8VFM5\|Q8VFM5_MOUSE | 23.74 | 2 | 2 | 5.74E+06 | 1 | 1 | 1 |  | 35283 | Olfactory receptor 700 OS=Mus musculus OX=10090 GN=Olfr700 PE=2 SV=1 |
| 141 | 9686 | Q7TRN3\|Q7TRN3_MOUSE | 23.74 | 2 | 2 | 5.74E+06 | 1 | 1 | 1 |  | 35091 | Olfactory receptor 699 OS=Mus musculus OX=10090 GN=Olfr699 PE=3 SV=1 |
| 141 | 9687 | Q8VGD7\|Q8VGD7_MOUSE | 23.74 | 2 | 2 | 5.74E+06 | 1 | 1 | 1 |  | 35454 | Olfactory receptor 1395 OS=Mus musculus OX=10090 GN=Olfr1395 PE=3 SV=1 |
| 141 | 9688 | Q8VFM4\|Q8VFM4_MOUSE | 23.74 | 2 | 2 | 5.74E+06 | 1 | 1 | 1 |  | 35471 | Olfactory receptor 697 OS=Mus musculus OX=10090 GN=Olfr697 PE=2 SV=1 |
| 141 | 9689 | Q2M2Q3\|Q2M2Q3_MOUSE | 23.74 | 2 | 2 | 5.74E+06 | 1 | 1 | 1 |  | 35362 | Olfr703 protein (Fragment) OS=Mus musculus OX=10090 GN=Olfr703 PE=2 SV=1 |
| 141 | 9690 | Q9EPF5\|Q9EPF5_MOUSE | 23.74 | 2 | 2 | 5.74E+06 | 1 | 1 | 1 |  | 35493 | Olfactory receptor 703 OS=Mus musculus OX=10090 GN=Olfr703 PE=3 SV=1 |
| 142 | 1066 | D6RH37\|D6RH37_MOUSE | 22.84 | 1 | 1 | 4.05E+05 | 1 | 1 | 1 |  | 87195 | Serine/threonine-protein kinase N1 OS=Mus musculus OX=10090 GN=Pkn1 PE=1 SV=1 |
| 142 | 1071 | sp\|P70268\|PKN1_MOUSE | 22.84 | 1 | 1 | 4.05E+05 | 1 | 1 | 1 |  | 104411 | Serine/threonine-protein kinase N1 OS=Mus musculus OX=10090 GN=Pkn1 PE=1 SV=3 |
| 106 | 648 | sp\|Q499E0\|BRNP3_MOUSE | 20.54 | 1 | 1 | 0.00E+00 | 1 | 1 | 1 |  | 88483 | BMP/retinoic acid-inducible neural-specific protein 3 OS=Mus musculus OX=10090 GN=Brinp3 PE=2 SV=2 |
| 143 | 993 | A0A2K6EDJ7\|A0A2K6EDJ7_MOUSE | 20.28 | 1 | 1 | 3.98E+06 | 1 | 1 | 1 |  | 100324 | Inter alpha-trypsin inhibitor heavy chain 4 OS=Mus musculus OX=10090 GN=Itih4 PE=1 SV=1 |
| 143 | 996 | sp\|A6X935\|ITIH4_MOUSE | 20.28 | 1 | 1 | 3.98E+06 | 1 | 1 | 1 |  | 104660 | Inter alpha-trypsin inhibitor heavy chain 4 OS=Mus musculus OX=10090 GN=Itih4 PE=1 SV=2 |
| 105 | 626 | Q545F8\|Q545F8_MOUSE | 20.07 | 3 | 3 | 6.56E+05 | 1 | 1 | 1 |  | 27504 | 40S ribosomal protein S4 OS=Mus musculus OX=10090 GN=Rps4x PE=2 SV=1 |
| 105 | 627 | Q3V1Z5\|Q3V1Z5_MOUSE | 20.07 | 3 | 3 | 6.56E+05 | 1 | 1 | 1 |  | 29184 | 40S ribosomal protein S4 OS=Mus musculus OX=10090 GN=Rps4l PE=2 SV=1 |
| 105 | 628 | sp\|P62702\|RS4X_MOUSE | 20.07 | 3 | 3 | 6.56E+05 | 1 | 1 | 1 |  | 29598 | 40S ribosomal protein S4 X isoform OS=Mus musculus OX=10090 GN=Rps4x PE=1 SV=2 |
| 105 | 629 | Q545X8\|Q545X8_MOUSE | 20.07 | 3 | 3 | 6.56E+05 | 1 | 1 | 1 |  | 29598 | 40S ribosomal protein S4 OS=Mus musculus OX=10090 GN=Rps4x PE=1 SV=1 |
| 105 | 630 | Q3UXQ6\|Q3UXQ6_MOUSE | 20.07 | 3 | 3 | 6.56E+05 | 1 | 1 | 1 |  | 29564 | 40S ribosomal protein S4 OS=Mus musculus OX=10090 GN=Rps4x PE=2 SV=1 |
| 105 | 867 | V9GWY0\|V9GWY0_MOUSE | 20.07 | 3 | 3 | 6.56E+05 | 1 | 1 | 1 |  | 29939 | 40S ribosomal protein S4 OS=Mus musculus OX=10090 GN=Gm15013 PE=3 SV=1 |
