# Supplementary material for: Tert promotes cardiac regenerative repair after MI through alleviating ROS-induced DNA damage response in cardiomyocyte
Source: Cell Death Discov. 2024 Aug 26;10:381. doi: 10.1038/s41420-024-02135-8 (PMC11347641; doi:10.1038/s41420-024-02135-8)
Supplement: Supplementary file 3 — Proteins identified by mass spectrometry from sample 3 [file 41420_2024_2135_MOESM3_ESM.docx]

Proteins identified by mass spectrometry from sample 3

| Protein Group | Protein ID | Accession | -10lgP | Coverage (%) | Coverage (%) 3 | Area 3 | #Peptides | #Unique | #Spec 3 | PTM | Avg. Mass | Description |
| --- | --- | --- | --- | --- | --- | --- | --- | --- | --- | --- | --- | --- |
| 8 | 50 | sp\|P15864\|H12_MOUSE | 154.37 | 39 | 39 | 4.36E+06 | 10 | 3 | 17 | Acetylation (Protein N-term) | 21267 | Histone H1.2 OS=Mus musculus OX=10090 GN=H1-2 PE=1 SV=2 |
| 8 | 51 | Q5SZA3\|Q5SZA3_MOUSE | 154.37 | 39 | 39 | 4.36E+06 | 10 | 3 | 17 | Acetylation (Protein N-term) | 21267 | Histone cluster 1 H1c OS=Mus musculus OX=10090 GN=H1f2 PE=1 SV=1 |
| 13 | 101 | sp\|P43274\|H14_MOUSE | 143.59 | 32 | 32 | 1.09E+07 | 7 | 2 | 16 | Acetylation (Protein N-term) | 21977 | Histone H1.4 OS=Mus musculus OX=10090 GN=H1-4 PE=1 SV=2 |
| 4 | 111 | Q3UF04\|Q3UF04_MOUSE | 131.51 | 27 | 27 | 5.94E+07 | 9 | 9 | 28 | Oxidation (M); Pyro-glu from Q | 28919 | Uncharacterized protein OS=Mus musculus OX=10090 GN=Atp5pb PE=2 SV=1 |
| 4 | 112 | Q3TJD4\|Q3TJD4_MOUSE | 131.51 | 27 | 27 | 5.94E+07 | 9 | 9 | 28 | Oxidation (M); Pyro-glu from Q | 28948 | Uncharacterized protein OS=Mus musculus OX=10090 GN=Atp5pb PE=2 SV=1 |
| 4 | 113 | sp\|Q9CQQ7\|AT5F1_MOUSE | 131.51 | 27 | 27 | 5.94E+07 | 9 | 9 | 28 | Oxidation (M); Pyro-glu from Q | 28949 | ATP synthase F(0) complex subunit B1 mitochondrial OS=Mus musculus OX=10090 GN=Atp5pb PE=1 SV=1 |
| 4 | 114 | Q5I0W0\|Q5I0W0_MOUSE | 131.51 | 27 | 27 | 5.94E+07 | 9 | 9 | 28 | Oxidation (M); Pyro-glu from Q | 28949 | ATP synthase H+ transporting mitochondrial F0 complex subunit b isoform 1 OS=Mus musculus OX=10090 GN=Atp5pb PE=1 SV=1 |
| 6 | 288 | sp\|P01868\|IGHG1_MOUSE | 124.91 | 20 | 20 | 5.33E+08 | 3 | 3 | 22 | Carbamidomethylation; Deamidation (NQ) | 35705 | Ig gamma-1 chain C region secreted form OS=Mus musculus OX=10090 GN=Ighg1 PE=1 SV=1 |
| 6 | 289 | sp\|P01869\|IGH1M_MOUSE | 124.91 | 17 | 17 | 5.33E+08 | 3 | 3 | 22 | Carbamidomethylation; Deamidation (NQ) | 43387 | Ig gamma-1 chain C region membrane-bound form OS=Mus musculus OX=10090 GN=Ighg1 PE=1 SV=2 |
| 6 | 249 | A0A0M4KM70\|A0A0M4KM70_MOUSE | 124.91 | 14 | 14 | 5.33E+08 | 3 | 3 | 22 | Carbamidomethylation; Deamidation (NQ) | 50295 | Monoclonal 11D8 anti-human butyrylcholinesterase (BChE) heavy chain OS=Mus musculus OX=10090 PE=2 SV=1 |
| 6 | 250 | U5LP42\|U5LP42_MOUSE | 124.91 | 14 | 14 | 5.33E+08 | 3 | 3 | 22 | Carbamidomethylation; Deamidation (NQ) | 51051 | Anti-H5N1 hemagglutinin monoclonal anitbody H5M9 heavy chain (Fragment) OS=Mus musculus OX=10090 PE=2 SV=1 |
| 6 | 251 | Q99LC4\|Q99LC4_MOUSE | 124.91 | 14 | 14 | 5.33E+08 | 3 | 3 | 22 | Carbamidomethylation; Deamidation (NQ) | 51008 | Igh protein OS=Mus musculus OX=10090 GN=Igh PE=1 SV=1 |
| 19 | 11 | sp\|P56480\|ATPB_MOUSE | 124.46 | 13 | 13 | 1.96E+07 | 5 | 5 | 10 | Oxidation (M) | 56301 | ATP synthase subunit beta mitochondrial OS=Mus musculus OX=10090 GN=Atp5f1b PE=1 SV=2 |
| 16 | 137 | Q1WWK3\|Q1WWK3_MOUSE | 119.63 | 20 | 20 | 4.01E+06 | 4 | 2 | 10 | Acetylation (Protein N-term) | 22445 | Hist1h1b protein (Fragment) OS=Mus musculus OX=10090 GN=H1f5 PE=2 SV=1 |
| 16 | 138 | sp\|P43276\|H15_MOUSE | 119.63 | 20 | 20 | 4.01E+06 | 4 | 2 | 10 | Acetylation (Protein N-term) | 22576 | Histone H1.5 OS=Mus musculus OX=10090 GN=H1-5 PE=1 SV=2 |
| 27 | 82 | sp\|Q9DB77\|QCR2_MOUSE | 101.73 | 11 | 11 | 5.80E+06 | 4 | 4 | 6 | Oxidation (M); Pyro-glu from Q | 48235 | Cytochrome b-c1 complex subunit 2 mitochondrial OS=Mus musculus OX=10090 GN=Uqcrc2 PE=1 SV=1 |
| 28 | 23 | Q8BVI9\|Q8BVI9_MOUSE | 96.38 | 10 | 10 | 3.79E+06 | 3 | 2 | 5 | Carbamidomethylation; Deamidation (NQ) | 32904 | Uncharacterized protein OS=Mus musculus OX=10090 GN=Slc25a4 PE=2 SV=1 |
| 28 | 14 | sp\|P48962\|ADT1_MOUSE | 96.38 | 10 | 10 | 3.79E+06 | 3 | 2 | 5 | Carbamidomethylation; Deamidation (NQ) | 32904 | ADP/ATP translocase 1 OS=Mus musculus OX=10090 GN=Slc25a4 PE=1 SV=4 |
| 29 | 12 | Q3V235\|Q3V235_MOUSE | 94.73 | 11 | 11 | 4.60E+06 | 4 | 4 | 4 |  | 33296 | Prohibitin OS=Mus musculus OX=10090 GN=Phb2 PE=1 SV=1 |
| 29 | 13 | sp\|O35129\|PHB2_MOUSE | 94.73 | 11 | 11 | 4.60E+06 | 4 | 4 | 4 |  | 33296 | Prohibitin-2 OS=Mus musculus OX=10090 GN=Phb2 PE=1 SV=1 |
| 22 | 126 | A0A1D5RLD8\|A0A1D5RLD8_MOUSE | 87.86 | 14 | 14 | 1.38E+07 | 4 | 4 | 7 | Carbamidomethylation; Deamidation (NQ) | 35812 | Glyceraldehyde-3-phosphate dehydrogenase OS=Mus musculus OX=10090 GN=Gm10358 PE=1 SV=1 |
| 22 | 97 | sp\|P16858\|G3P_MOUSE | 87.86 | 14 | 14 | 1.38E+07 | 4 | 4 | 7 | Carbamidomethylation; Deamidation (NQ) | 35810 | Glyceraldehyde-3-phosphate dehydrogenase OS=Mus musculus OX=10090 GN=Gapdh PE=1 SV=2 |
| 22 | 98 | D2KHZ9\|D2KHZ9_MOUSE | 87.86 | 14 | 14 | 1.38E+07 | 4 | 4 | 7 | Carbamidomethylation; Deamidation (NQ) | 35810 | Glyceraldehyde-3-phosphate dehydrogenase OS=Mus musculus OX=10090 GN=GAPDH PE=2 SV=1 |
| 22 | 99 | A0A0A0MQF6\|A0A0A0MQF6_MOUSE | 87.86 | 13 | 13 | 1.38E+07 | 4 | 4 | 7 | Carbamidomethylation; Deamidation (NQ) | 38653 | Glyceraldehyde-3-phosphate dehydrogenase OS=Mus musculus OX=10090 GN=Gapdh PE=1 SV=1 |
| 39 | 353 | Q99N15\|Q99N15_MOUSE | 86.97 | 14 | 14 | 6.73E+06 | 3 | 3 | 4 |  | 27274 | 17beta-hydroxysteroid dehydrogenase type 10/short chain L-3-hydroxyacyl-CoA dehydrogenase OS=Mus musculus OX=10090 GN=Hsd17b10 PE=1 SV=1 |
| 39 | 354 | sp\|O08756\|HCD2_MOUSE | 86.97 | 14 | 14 | 6.73E+06 | 3 | 3 | 4 |  | 27419 | 3-hydroxyacyl-CoA dehydrogenase type-2 OS=Mus musculus OX=10090 GN=Hsd17b10 PE=1 SV=4 |
| 39 | 355 | A2AFQ2\|A2AFQ2_MOUSE | 86.97 | 14 | 14 | 6.73E+06 | 3 | 3 | 4 |  | 28374 | 3-hydroxyacyl-CoA dehydrogenase type-2 OS=Mus musculus OX=10090 GN=Hsd17b10 PE=1 SV=1 |
| 31 | 178 | sp\|P17751\|TPIS_MOUSE | 84.27 | 14 | 14 | 5.17E+06 | 4 | 4 | 5 | Carbamidomethylation | 32192 | Triosephosphate isomerase OS=Mus musculus OX=10090 GN=Tpi1 PE=1 SV=4 |
| 36 | 154 | sp\|Q99JY0\|ECHB_MOUSE | 78.42 | 7 | 7 | 5.58E+06 | 4 | 4 | 4 |  | 51386 | Trifunctional enzyme subunit beta mitochondrial OS=Mus musculus OX=10090 GN=Hadhb PE=1 SV=1 |
| 30 | 191 | Q3UFI4\|Q3UFI4_MOUSE | 76.48 | 9 | 9 | 6.64E+06 | 3 | 3 | 5 |  | 33529 | 60S ribosomal protein L6 OS=Mus musculus OX=10090 GN=Rpl6 PE=2 SV=1 |
| 30 | 192 | sp\|P47911\|RL6_MOUSE | 76.48 | 9 | 9 | 6.64E+06 | 3 | 3 | 5 |  | 33510 | 60S ribosomal protein L6 OS=Mus musculus OX=10090 GN=Rpl6 PE=1 SV=3 |
| 30 | 193 | Q3UCH0\|Q3UCH0_MOUSE | 76.48 | 9 | 9 | 6.64E+06 | 3 | 3 | 5 |  | 33510 | 60S ribosomal protein L6 OS=Mus musculus OX=10090 GN=Rpl6 PE=1 SV=1 |
| 54 | 282 | sp\|P14602\|HSPB1_MOUSE | 76.44 | 14 | 14 | 2.91E+06 | 3 | 3 | 3 | Pyro-glu from Q | 23014 | Heat shock protein beta-1 OS=Mus musculus OX=10090 GN=Hspb1 PE=1 SV=3 |
| 54 | 283 | Q545F4\|Q545F4_MOUSE | 76.44 | 14 | 14 | 2.91E+06 | 3 | 3 | 3 | Pyro-glu from Q | 23014 | Hspb1 protein OS=Mus musculus OX=10090 GN=Hspb1 PE=2 SV=1 |
| 44 | 128 | sp\|Q9DCT2\|NDUS3_MOUSE | 73.04 | 10 | 10 | 4.05E+06 | 2 | 2 | 4 |  | 30149 | NADH dehydrogenase [ubiquinone] iron-sulfur protein 3 mitochondrial OS=Mus musculus OX=10090 GN=Ndufs3 PE=1 SV=2 |
| 56 | 94 | sp\|P51881\|ADT2_MOUSE | 71.85 | 5 | 5 | 5.38E+05 | 2 | 1 | 3 | Acetylation (Protein N-term) | 32931 | ADP/ATP translocase 2 OS=Mus musculus OX=10090 GN=Slc25a5 PE=1 SV=3 |
| 56 | 95 | Q545A2\|Q545A2_MOUSE | 71.85 | 5 | 5 | 5.38E+05 | 2 | 1 | 3 | Acetylation (Protein N-term) | 32931 | MCG11560 OS=Mus musculus OX=10090 GN=Slc25a5 PE=1 SV=1 |
| 64 | 574 | B8JJH1\|B8JJH1_MOUSE | 71.3 | 27 | 27 | 2.77E+05 | 1 | 1 | 1 | Acetylation (Protein N-term) | 6701 | Myosin-7 (Fragment) OS=Mus musculus OX=10090 GN=Myh7 PE=1 SV=8 |
| 64 | 575 | A0A2I3BQV3\|A0A2I3BQV3_MOUSE | 71.3 | 18 | 18 | 2.77E+05 | 1 | 1 | 1 | Acetylation (Protein N-term) | 9758 | Myosin-7 (Fragment) OS=Mus musculus OX=10090 GN=Myh7 PE=1 SV=1 |
| 64 | 576 | Q1WJL6\|Q1WJL6_MOUSE | 71.3 | 17 | 17 | 2.77E+05 | 1 | 1 | 1 | Acetylation (Protein N-term) | 10550 | Cardiac myosin heavy chain beta (Fragment) OS=Mus musculus OX=10090 GN=Myh7 PE=1 SV=1 |
| 64 | 40 | sp\|Q91Z83\|MYH7_MOUSE | 71.3 | 1 | 1 | 2.77E+05 | 1 | 1 | 1 | Acetylation (Protein N-term) | 222877 | Myosin-7 OS=Mus musculus OX=10090 GN=Myh7 PE=2 SV=1 |
| 64 | 39 | B2RY26\|B2RY26_MOUSE | 71.3 | 1 | 1 | 2.77E+05 | 1 | 1 | 1 | Acetylation (Protein N-term) | 222847 | Myh7 protein OS=Mus musculus OX=10090 GN=Myh7 PE=2 SV=1 |
| 64 | 41 | B2RXX9\|B2RXX9_MOUSE | 71.3 | 1 | 1 | 2.77E+05 | 1 | 1 | 1 | Acetylation (Protein N-term) | 222877 | Myosin heavy polypeptide 7 cardiac muscle beta OS=Mus musculus OX=10090 GN=Myh7 PE=2 SV=1 |
| 43 | 172 | Q08EK4\|Q08EK4_MOUSE | 70.68 | 3 | 3 | 4.23E+05 | 2 | 1 | 3 |  | 61302 | Keratin 77 OS=Mus musculus OX=10090 GN=Krt77 PE=2 SV=1 |
| 43 | 174 | Q08EK5\|Q08EK5_MOUSE | 70.68 | 3 | 3 | 4.23E+05 | 2 | 1 | 3 |  | 61359 | Keratin 77 OS=Mus musculus OX=10090 GN=Krt77 PE=1 SV=1 |
| 43 | 175 | sp\|Q6IFZ6\|K2C1B_MOUSE | 70.68 | 3 | 3 | 4.23E+05 | 2 | 1 | 3 |  | 61359 | Keratin type II cytoskeletal 1b OS=Mus musculus OX=10090 GN=Krt77 PE=1 SV=1 |
| 37 | 259 | Q3THU8\|Q3THU8_MOUSE | 67.71 | 6 | 6 | 2.84E+06 | 2 | 2 | 3 |  | 39613 | Uncharacterized protein OS=Mus musculus OX=10090 GN=Slc25a3 PE=2 SV=1 |
| 37 | 260 | Q3U995\|Q3U995_MOUSE | 67.71 | 6 | 6 | 2.84E+06 | 2 | 2 | 3 |  | 39633 | Uncharacterized protein OS=Mus musculus OX=10090 GN=Slc25a3 PE=2 SV=1 |
| 37 | 261 | sp\|Q8VEM8\|MPCP_MOUSE | 67.71 | 6 | 6 | 2.84E+06 | 2 | 2 | 3 |  | 39632 | Phosphate carrier protein mitochondrial OS=Mus musculus OX=10090 GN=Slc25a3 PE=1 SV=1 |
| 37 | 205 | G5E902\|G5E902_MOUSE | 67.71 | 6 | 6 | 2.84E+06 | 2 | 2 | 3 |  | 39736 | MCG10343 isoform CRA_b OS=Mus musculus OX=10090 GN=Slc25a3 PE=1 SV=1 |
| 25 | 243 | sp\|Q3UV17\|K22O_MOUSE | 66.69 | 3 | 3 | 4.90E+06 | 3 | 2 | 5 |  | 62845 | Keratin type II cytoskeletal 2 oral OS=Mus musculus OX=10090 GN=Krt76 PE=1 SV=1 |
| 51 | 81 | Q3UAA9\|Q3UAA9_MOUSE | 65.1 | 7 | 7 | 5.78E+05 | 3 | 1 | 3 |  | 41679 | Uncharacterized protein OS=Mus musculus OX=10090 GN=Actb PE=2 SV=1 |
| 51 | 42 | Q3UAF6\|Q3UAF6_MOUSE | 65.1 | 7 | 7 | 5.78E+05 | 3 | 1 | 3 |  | 41811 | Uncharacterized protein OS=Mus musculus OX=10090 GN=Actb PE=2 SV=1 |
| 51 | 90 | Q3UGS0\|Q3UGS0_MOUSE | 65.1 | 7 | 7 | 5.78E+05 | 3 | 1 | 3 |  | 41765 | Uncharacterized protein OS=Mus musculus OX=10090 GN=Actb PE=2 SV=1 |
| 51 | 49 | Q3TSB7\|Q3TSB7_MOUSE | 65.1 | 7 | 7 | 5.78E+05 | 3 | 1 | 3 |  | 41738 | Uncharacterized protein OS=Mus musculus OX=10090 GN=Actg1 PE=2 SV=1 |
| 51 | 119 | Q3UBP6\|Q3UBP6_MOUSE | 65.1 | 7 | 7 | 5.78E+05 | 3 | 1 | 3 |  | 41769 | Uncharacterized protein OS=Mus musculus OX=10090 GN=Actb PE=2 SV=1 |
| 51 | 43 | Q4KL81\|Q4KL81_MOUSE | 65.1 | 7 | 7 | 5.78E+05 | 3 | 1 | 3 |  | 41793 | Actin gamma cytoplasmic 1 OS=Mus musculus OX=10090 GN=Actg1 PE=2 SV=1 |
| 51 | 44 | Q3U5R4\|Q3U5R4_MOUSE | 65.1 | 7 | 7 | 5.78E+05 | 3 | 1 | 3 |  | 41709 | Uncharacterized protein OS=Mus musculus OX=10090 GN=Actb PE=2 SV=1 |
| 51 | 47 | B2RRX1\|B2RRX1_MOUSE | 65.1 | 7 | 7 | 5.78E+05 | 3 | 1 | 3 |  | 41737 | Actin beta OS=Mus musculus OX=10090 GN=Actb PE=2 SV=1 |
| 51 | 48 | Q3UAF7\|Q3UAF7_MOUSE | 65.1 | 7 | 7 | 5.78E+05 | 3 | 1 | 3 |  | 41751 | Uncharacterized protein OS=Mus musculus OX=10090 GN=Actb PE=2 SV=1 |
| 51 | 132 | E9Q1F2\|E9Q1F2_MOUSE | 65.1 | 9 | 9 | 5.78E+05 | 3 | 1 | 3 |  | 32564 | Actin cytoplasmic 1 OS=Mus musculus OX=10090 GN=Actb PE=1 SV=1 |
| 42 | 188 | sp\|Q9CR62\|M2OM_MOUSE | 64.82 | 8 | 8 | 1.59E+06 | 2 | 2 | 2 | Acetylation (Protein N-term) | 34155 | Mitochondrial 2-oxoglutarate/malate carrier protein OS=Mus musculus OX=10090 GN=Slc25a11 PE=1 SV=3 |
| 42 | 189 | Q5SX53\|Q5SX53_MOUSE | 64.82 | 8 | 8 | 1.59E+06 | 2 | 2 | 2 | Acetylation (Protein N-term) | 34155 | Solute carrier family 25 (Mitochondrial carrier oxoglutarate carrier) member 11 isoform CRA_b OS=Mus musculus OX=10090 GN=Slc25a11 PE=1 SV=1 |
| 57 | 252 | sp\|Q9DB20\|ATPO_MOUSE | 61.81 | 11 | 11 | 2.61E+06 | 2 | 2 | 3 |  | 23364 | ATP synthase subunit O mitochondrial OS=Mus musculus OX=10090 GN=Atp5po PE=1 SV=1 |
| 57 | 253 | Q3TF25\|Q3TF25_MOUSE | 61.81 | 11 | 11 | 2.61E+06 | 2 | 2 | 3 |  | 23364 | Uncharacterized protein OS=Mus musculus OX=10090 GN=Atp5o PE=2 SV=1 |
| 52 | 53 | Q3UJ36\|Q3UJ36_MOUSE | 61.55 | 9 | 9 | 1.74E+06 | 3 | 1 | 3 |  | 41877 | Actin gamma 2 smooth muscle enteric isoform CRA_a OS=Mus musculus OX=10090 GN=Actg2 PE=2 SV=1 |
| 52 | 25 | Q9CXK3\|Q9CXK3_MOUSE | 61.55 | 9 | 9 | 1.74E+06 | 3 | 1 | 3 |  | 41947 | Uncharacterized protein OS=Mus musculus OX=10090 GN=Actc1 PE=2 SV=1 |
| 52 | 59 | Q3UIJ3\|Q3UIJ3_MOUSE | 61.55 | 9 | 9 | 1.74E+06 | 3 | 1 | 3 |  | 42118 | Uncharacterized protein OS=Mus musculus OX=10090 GN=Actc1 PE=2 SV=1 |
| 52 | 28 | Q497E4\|Q497E4_MOUSE | 61.55 | 9 | 9 | 1.74E+06 | 3 | 1 | 3 |  | 42019 | Actin alpha cardiac muscle 1 OS=Mus musculus OX=10090 GN=Actc1 PE=2 SV=1 |
| 52 | 54 | Q3U122\|Q3U122_MOUSE | 61.55 | 9 | 9 | 1.74E+06 | 3 | 1 | 3 |  | 41995 | Uncharacterized protein OS=Mus musculus OX=10090 GN=Acta2 PE=2 SV=1 |
| 52 | 31 | Q3TG92\|Q3TG92_MOUSE | 61.55 | 9 | 9 | 1.74E+06 | 3 | 1 | 3 |  | 42047 | Uncharacterized protein OS=Mus musculus OX=10090 GN=Actc1 PE=2 SV=1 |
| 40 | 357 | A0A1W2P6Q3\|A0A1W2P6Q3_MOUSE | 61.34 | 19 | 19 | 2.23E+06 | 3 | 3 | 3 | Acetylation (Protein N-term) | 19572 | T-complex protein 1 subunit beta OS=Mus musculus OX=10090 GN=Cct2 PE=1 SV=1 |
| 40 | 296 | A0A1W2P7B7\|A0A1W2P7B7_MOUSE | 61.34 | 16 | 16 | 2.23E+06 | 3 | 3 | 3 | Acetylation (Protein N-term) | 22940 | T-complex protein 1 subunit beta (Fragment) OS=Mus musculus OX=10090 GN=Cct2 PE=1 SV=1 |
| 40 | 297 | Q9JJD8\|Q9JJD8_MOUSE | 61.34 | 7 | 7 | 2.23E+06 | 3 | 3 | 3 | Acetylation (Protein N-term) | 52469 | Brain cDNA clone MNCb-1272 similar to Mus musculus chaperonin subunit 2 (beta) (Cct2) mRNA OS=Mus musculus OX=10090 GN=Cct2 PE=2 SV=1 |
| 40 | 298 | Q542X7\|Q542X7_MOUSE | 61.34 | 6 | 6 | 2.23E+06 | 3 | 3 | 3 | Acetylation (Protein N-term) | 57477 | Chaperonin subunit 2 (Beta) isoform CRA_a OS=Mus musculus OX=10090 GN=Cct2 PE=1 SV=1 |
| 40 | 299 | sp\|P80314\|TCPB_MOUSE | 61.34 | 6 | 6 | 2.23E+06 | 3 | 3 | 3 | Acetylation (Protein N-term) | 57477 | T-complex protein 1 subunit beta OS=Mus musculus OX=10090 GN=Cct2 PE=1 SV=4 |
| 73 | 130 | F2Z471\|F2Z471_MOUSE | 59.3 | 9 | 9 | 3.21E+06 | 2 | 2 | 2 | Acetylation (Protein N-term) | 28157 | Voltage-dependent anion-selective channel protein 1 OS=Mus musculus OX=10090 GN=Vdac1 PE=1 SV=1 |
| 73 | 89 | Q3THL7\|Q3THL7_MOUSE | 59.3 | 8 | 8 | 3.21E+06 | 2 | 2 | 2 | Acetylation (Protein N-term) | 30756 | Uncharacterized protein OS=Mus musculus OX=10090 GN=Vdac1 PE=2 SV=1 |
| 73 | 116 | Q3TIG8\|Q3TIG8_MOUSE | 59.3 | 8 | 8 | 3.21E+06 | 2 | 2 | 2 | Acetylation (Protein N-term) | 30796 | Uncharacterized protein OS=Mus musculus OX=10090 GN=Vdac1 PE=2 SV=1 |
| 73 | 75 | Q3U6K8\|Q3U6K8_MOUSE | 59.3 | 8 | 8 | 3.21E+06 | 2 | 2 | 2 | Acetylation (Protein N-term) | 30725 | Uncharacterized protein OS=Mus musculus OX=10090 GN=Vdac1 PE=2 SV=1 |
| 73 | 76 | sp\|Q60932\|VDAC1_MOUSE | 59.3 | 7 | 7 | 3.21E+06 | 2 | 2 | 2 | Acetylation (Protein N-term) | 32351 | Voltage-dependent anion-selective channel protein 1 OS=Mus musculus OX=10090 GN=Vdac1 PE=1 SV=3 |
| 53 | 15 | Q3TEK2\|Q3TEK2_MOUSE | 55.51 | 2 | 2 | 1.48E+06 | 1 | 1 | 1 |  | 70857 | Uncharacterized protein OS=Mus musculus OX=10090 GN=Hspa8 PE=2 SV=1 |
| 41 | 416 | sp\|P24472\|GSTA4_MOUSE | 53.89 | 10 | 10 | 4.20E+06 | 3 | 3 | 4 |  | 25564 | Glutathione S-transferase A4 OS=Mus musculus OX=10090 GN=Gsta4 PE=1 SV=3 |
| 34 | 525 | A0A3B2WDD2\|A0A3B2WDD2_MOUSE | 52.18 | 8 | 8 | 1.02E+07 | 2 | 2 | 5 |  | 21637 | Ribosomal protein OS=Mus musculus OX=10090 GN=Rpl10a PE=1 SV=1 |
| 34 | 526 | A0A3B2WBL1\|A0A3B2WBL1_MOUSE | 52.18 | 7 | 7 | 1.02E+07 | 2 | 2 | 5 |  | 24744 | Ribosomal protein OS=Mus musculus OX=10090 GN=Rpl10a PE=1 SV=1 |
| 34 | 527 | sp\|P53026\|RL10A_MOUSE | 52.18 | 7 | 7 | 1.02E+07 | 2 | 2 | 5 |  | 24916 | 60S ribosomal protein L10a OS=Mus musculus OX=10090 GN=Rpl10a PE=1 SV=3 |
| 34 | 528 | Q3U561\|Q3U561_MOUSE | 52.18 | 7 | 7 | 1.02E+07 | 2 | 2 | 5 |  | 24815 | Ribosomal protein OS=Mus musculus OX=10090 GN=Rpl10a PE=2 SV=1 |
| 34 | 529 | Q5XJF6\|Q5XJF6_MOUSE | 52.18 | 7 | 7 | 1.02E+07 | 2 | 2 | 5 |  | 24831 | Ribosomal protein OS=Mus musculus OX=10090 GN=Rpl10a PE=1 SV=1 |
| 75 | 729 | Q8K2S8\|Q8K2S8_MOUSE | 47.7 | 3 | 3 | 1.90E+06 | 1 | 1 | 2 |  | 28718 | Uqcrc1 protein OS=Mus musculus OX=10090 GN=Uqcrc1 PE=2 SV=1 |
| 75 | 734 | Q3TIC8\|Q3TIC8_MOUSE | 47.7 | 2 | 2 | 1.90E+06 | 1 | 1 | 2 |  | 52753 | Uncharacterized protein OS=Mus musculus OX=10090 GN=Uqcrc1 PE=2 SV=1 |
| 75 | 735 | sp\|Q9CZ13\|QCR1_MOUSE | 47.7 | 2 | 2 | 1.90E+06 | 1 | 1 | 2 |  | 52852 | Cytochrome b-c1 complex subunit 1 mitochondrial OS=Mus musculus OX=10090 GN=Uqcrc1 PE=1 SV=2 |
| 75 | 736 | Q3THM1\|Q3THM1_MOUSE | 47.7 | 2 | 2 | 1.90E+06 | 1 | 1 | 2 |  | 52840 | Uncharacterized protein OS=Mus musculus OX=10090 GN=Uqcrc1 PE=2 SV=1 |
| 33 | 109 | D3Z6F5\|D3Z6F5_MOUSE | 45.97 | 3 | 3 | 2.22E+06 | 2 | 1 | 3 |  | 54595 | ATP synthase subunit alpha OS=Mus musculus OX=10090 GN=Atp5a1 PE=1 SV=1 |
| 33 | 108 | sp\|Q03265\|ATPA_MOUSE | 45.97 | 2 | 2 | 2.22E+06 | 2 | 1 | 3 |  | 59753 | ATP synthase subunit alpha mitochondrial OS=Mus musculus OX=10090 GN=Atp5f1a PE=1 SV=1 |
| 74 | 530 | sp\|P56135\|ATPK_MOUSE | 45.66 | 24 | 24 | 2.29E+06 | 2 | 2 | 2 | Acetylation (Protein N-term) | 10344 | ATP synthase subunit f mitochondrial OS=Mus musculus OX=10090 GN=Atp5mf PE=1 SV=3 |
| 76 | 726 | Q58DZ1\|Q58DZ1_MOUSE | 44.98 | 12 | 12 | 1.64E+06 | 1 | 1 | 2 |  | 8053 | Rpl31 protein OS=Mus musculus OX=10090 GN=Rpl31 PE=2 SV=1 |
| 76 | 502 | A0A0A6YXL3\|A0A0A6YXL3_MOUSE | 44.98 | 10 | 10 | 1.64E+06 | 1 | 1 | 2 |  | 9942 | 60S ribosomal protein L31 OS=Mus musculus OX=10090 GN=Rpl31 PE=1 SV=1 |
| 76 | 448 | Q5M9K9\|Q5M9K9_MOUSE | 44.98 | 7 | 7 | 1.64E+06 | 1 | 1 | 2 |  | 14463 | MCG126194 isoform CRA_a OS=Mus musculus OX=10090 GN=Rpl31 PE=1 SV=1 |
| 76 | 449 | sp\|P62900\|RL31_MOUSE | 44.98 | 7 | 7 | 1.64E+06 | 1 | 1 | 2 |  | 14463 | 60S ribosomal protein L31 OS=Mus musculus OX=10090 GN=Rpl31 PE=1 SV=1 |
| 76 | 450 | Q9CY93\|Q9CY93_MOUSE | 44.98 | 7 | 7 | 1.64E+06 | 1 | 1 | 2 |  | 14411 | Uncharacterized protein OS=Mus musculus OX=10090 GN=Rpl31 PE=2 SV=1 |
| 76 | 503 | A0A0A6YX26\|A0A0A6YX26_MOUSE | 44.98 | 7 | 7 | 1.64E+06 | 1 | 1 | 2 |  | 14997 | 60S ribosomal protein L31 OS=Mus musculus OX=10090 GN=Rpl31 PE=1 SV=1 |
| 65 | 121 | Q58E64\|Q58E64_MOUSE | 44.79 | 2 | 2 | 1.47E+06 | 1 | 1 | 2 |  | 50114 | Elongation factor 1-alpha OS=Mus musculus OX=10090 GN=Eef1a1 PE=1 SV=1 |
| 65 | 122 | sp\|P10126\|EF1A1_MOUSE | 44.79 | 2 | 2 | 1.47E+06 | 1 | 1 | 2 |  | 50114 | Elongation factor 1-alpha 1 OS=Mus musculus OX=10090 GN=Eef1a1 PE=1 SV=3 |
| 65 | 123 | Q3UA81\|Q3UA81_MOUSE | 44.79 | 2 | 2 | 1.47E+06 | 1 | 1 | 2 |  | 50113 | Elongation factor 1-alpha OS=Mus musculus OX=10090 GN=Eef1a1 PE=2 SV=1 |
| 65 | 124 | Q3UZQ3\|Q3UZQ3_MOUSE | 44.79 | 2 | 2 | 1.47E+06 | 1 | 1 | 2 |  | 50066 | Elongation factor 1-alpha OS=Mus musculus OX=10090 GN=Eef1a1 PE=2 SV=1 |
| 65 | 125 | Q3TII3\|Q3TII3_MOUSE | 44.79 | 2 | 2 | 1.47E+06 | 1 | 1 | 2 |  | 50104 | Elongation factor 1-alpha OS=Mus musculus OX=10090 GN=Eef1a1 PE=2 SV=1 |
| 65 | 167 | sp\|P62631\|EF1A2_MOUSE | 44.79 | 2 | 2 | 1.47E+06 | 1 | 1 | 2 |  | 50454 | Elongation factor 1-alpha 2 OS=Mus musculus OX=10090 GN=Eef1a2 PE=1 SV=1 |
| 20 | 730 | A0A075B5V3\|A0A075B5V3_MOUSE | 41.91 | 21 | 21 | 2.20E+07 | 1 | 1 | 8 | Carbamidomethylation | 12904 | Immunoglobulin heavy variable 1-36 OS=Mus musculus OX=10090 GN=Ighv1-36 PE=4 SV=1 |
| 20 | 731 | A0A075B5U5\|A0A075B5U5_MOUSE | 41.91 | 21 | 21 | 2.20E+07 | 1 | 1 | 8 | Carbamidomethylation | 12857 | Immunoglobulin heavy variable V1-19 OS=Mus musculus OX=10090 GN=Ighv1-19 PE=1 SV=1 |
| 20 | 732 | A0A0A6YXT2\|A0A0A6YXT2_MOUSE | 41.91 | 21 | 21 | 2.20E+07 | 1 | 1 | 8 | Carbamidomethylation | 12961 | Immunoglobulin heavy variable 1-36 (Fragment) OS=Mus musculus OX=10090 GN=Ighv1-36 PE=4 SV=1 |
| 20 | 733 | A0A0A6YWX0\|A0A0A6YWX0_MOUSE | 41.91 | 21 | 21 | 2.20E+07 | 1 | 1 | 8 | Carbamidomethylation | 12914 | Immunoglobulin heavy variable V1-19 (Fragment) OS=Mus musculus OX=10090 GN=Ighv1-19 PE=1 SV=1 |
| 128 | 727 | sp\|O55143\|AT2A2_MOUSE | 41.18 | 1 | 1 | 1.04E+06 | 1 | 1 | 1 | Acetylation (Protein N-term) | 114858 | Sarcoplasmic/endoplasmic reticulum calcium ATPase 2 OS=Mus musculus OX=10090 GN=Atp2a2 PE=1 SV=2 |
| 128 | 738 | Q5DTI2\|Q5DTI2_MOUSE | 41.18 | 1 | 1 | 1.04E+06 | 1 | 1 | 1 | Acetylation (Protein N-term) | 116600 | ATPase Ca++ transporting cardiac muscle slow twitch 2 isoform CRA_b (Fragment) OS=Mus musculus OX=10090 GN=Atp2a2 PE=2 SV=1 |
| 66 | 442 | sp\|Q8BMF4\|ODP2_MOUSE | 40.89 | 1 | 1 | 7.67E+05 | 1 | 1 | 2 |  | 67942 | Dihydrolipoyllysine-residue acetyltransferase component of pyruvate dehydrogenase complex mitochondrial OS=Mus musculus OX=10090 GN=Dlat PE=1 SV=2 |
| 137 | 486 | sp\|Q9CR57\|RL14_MOUSE | 40.08 | 6 | 6 | 4.20E+06 | 1 | 1 | 1 |  | 23564 | 60S ribosomal protein L14 OS=Mus musculus OX=10090 GN=Rpl14 PE=1 SV=3 |
| 137 | 487 | Q9CWK0\|Q9CWK0_MOUSE | 40.08 | 6 | 6 | 4.20E+06 | 1 | 1 | 1 |  | 26316 | Ribosomal_L14e domain-containing protein OS=Mus musculus OX=10090 GN=Rpl14 PE=2 SV=1 |
| 77 | 839 | A0A1B0GSF7\|A0A1B0GSF7_MOUSE | 39.73 | 7 | 7 | 2.31E+06 | 1 | 1 | 2 |  | 15245 | 60S ribosomal protein L18 OS=Mus musculus OX=10090 GN=Rpl18 PE=1 SV=1 |
| 77 | 535 | A0A1B0GSS8\|A0A1B0GSS8_MOUSE | 39.73 | 6 | 6 | 2.31E+06 | 1 | 1 | 2 |  | 17958 | 60S ribosomal protein L18 OS=Mus musculus OX=10090 GN=Rpl18 PE=1 SV=1 |
| 77 | 840 | A0A1B0GQU8\|A0A1B0GQU8_MOUSE | 39.73 | 6 | 6 | 2.31E+06 | 1 | 1 | 2 |  | 18101 | 60S ribosomal protein L18 OS=Mus musculus OX=10090 GN=Rpl18 PE=1 SV=1 |
| 77 | 841 | Q0QEW9\|Q0QEW9_MOUSE | 39.73 | 5 | 5 | 2.31E+06 | 1 | 1 | 2 |  | 19231 | Ribosomal protein L18 (Fragment) OS=Mus musculus OX=10090 GN=Rpl18 PE=2 SV=1 |
| 77 | 536 | Q58EW0\|Q58EW0_MOUSE | 39.73 | 5 | 5 | 2.31E+06 | 1 | 1 | 2 |  | 21645 | MCG132477 isoform CRA_a OS=Mus musculus OX=10090 GN=Rpl18 PE=1 SV=1 |
| 77 | 537 | sp\|P35980\|RL18_MOUSE | 39.73 | 5 | 5 | 2.31E+06 | 1 | 1 | 2 |  | 21645 | 60S ribosomal protein L18 OS=Mus musculus OX=10090 GN=Rpl18 PE=1 SV=3 |
| 77 | 538 | Q642K1\|Q642K1_MOUSE | 39.73 | 5 | 5 | 2.31E+06 | 1 | 1 | 2 |  | 21644 | Ribosomal protein L18 OS=Mus musculus OX=10090 GN=Rpl18 PE=2 SV=1 |
| 139 | 443 | A0A0J9YKD4\|A0A0J9YKD4_MOUSE | 36.44 | 2 | 2 | 1.59E+06 | 1 | 1 | 1 |  | 35108 | Creatine kinase M-type OS=Mus musculus OX=10090 GN=Ckm PE=1 SV=1 |
| 139 | 444 | sp\|P07310\|KCRM_MOUSE | 36.44 | 2 | 2 | 1.59E+06 | 1 | 1 | 1 |  | 43045 | Creatine kinase M-type OS=Mus musculus OX=10090 GN=Ckm PE=1 SV=1 |
| 139 | 445 | A2RTA0\|A2RTA0_MOUSE | 36.44 | 2 | 2 | 1.59E+06 | 1 | 1 | 1 |  | 43045 | Creatine kinase muscle OS=Mus musculus OX=10090 GN=Ckm PE=1 SV=1 |
| 139 | 446 | Q9D6U7\|Q9D6U7_MOUSE | 36.44 | 2 | 2 | 1.59E+06 | 1 | 1 | 1 |  | 43031 | Uncharacterized protein OS=Mus musculus OX=10090 GN=Ckm PE=2 SV=1 |
| 60 | 356 | A0A0B6VMB2\|A0A0B6VMB2_MOUSE | 36.42 | 7 | 7 | 1.42E+07 | 1 | 1 | 2 | Carbamidomethylation; Deamidation (NQ); Oxidation (M) | 50725 | MAb 31C6 heavy chain OS=Mus musculus OX=10090 GN=HC PE=4 SV=1 |
| 60 | 365 | A0A4U9FFL2\|A0A4U9FFL2_MOUSE | 36.42 | 10 | 10 | 1.42E+07 | 1 | 1 | 2 | Carbamidomethylation; Deamidation (NQ); Oxidation (M) | 35634 | IgG1 (Fragment) OS=Mus musculus OX=10090 GN=Ighg1 PE=4 SV=1 |
| 60 | 366 | A0A075B5P4\|A0A075B5P4_MOUSE | 36.42 | 10 | 10 | 1.42E+07 | 1 | 1 | 2 | Carbamidomethylation; Deamidation (NQ); Oxidation (M) | 35752 | Ig gamma-1 chain C region secreted form (Fragment) OS=Mus musculus OX=10090 GN=Ighg1 PE=1 SV=1 |
| 60 | 367 | A0A0A6YWR2\|A0A0A6YWR2_MOUSE | 36.42 | 8 | 8 | 1.42E+07 | 1 | 1 | 2 | Carbamidomethylation; Deamidation (NQ); Oxidation (M) | 43434 | Ig gamma-1 chain C region secreted form (Fragment) OS=Mus musculus OX=10090 GN=Ighg1 PE=1 SV=1 |
| 60 | 368 | A0A0C6E3V3\|A0A0C6E3V3_MOUSE | 36.42 | 7 | 7 | 1.42E+07 | 1 | 1 | 2 | Carbamidomethylation; Deamidation (NQ); Oxidation (M) | 51022 | HC protein OS=Mus musculus OX=10090 GN=HC PE=2 SV=1 |
| 67 | 770 | A0AUV1\|A0AUV1_MOUSE | 35.71 | 7 | 7 | 2.58E+06 | 1 | 1 | 2 |  | 13819 | Histone H2A (Fragment) OS=Mus musculus OX=10090 GN=H2ac12 PE=2 SV=1 |
| 67 | 772 | sp\|Q8CGP6\|H2A1H_MOUSE | 35.71 | 7 | 7 | 2.58E+06 | 1 | 1 | 2 |  | 13950 | Histone H2A type 1-H OS=Mus musculus OX=10090 GN=Hist1h2ah PE=1 SV=3 |
| 67 | 773 | A3KPD0\|A3KPD0_MOUSE | 35.71 | 7 | 7 | 2.58E+06 | 1 | 1 | 2 |  | 13950 | Histone H2A OS=Mus musculus OX=10090 GN=H2ac12 PE=2 SV=1 |
| 67 | 776 | Q149V4\|Q149V4_MOUSE | 35.71 | 7 | 7 | 2.58E+06 | 1 | 1 | 2 |  | 13988 | Histone H2A OS=Mus musculus OX=10090 GN=H2ac20 PE=2 SV=1 |
| 67 | 777 | Q8CGP4\|Q8CGP4_MOUSE | 35.71 | 7 | 7 | 2.58E+06 | 1 | 1 | 2 |  | 14056 | Histone H2A OS=Mus musculus OX=10090 GN=H2ac1 PE=1 SV=1 |
| 67 | 778 | sp\|Q64523\|H2A2C_MOUSE | 35.71 | 7 | 7 | 2.58E+06 | 1 | 1 | 2 |  | 13988 | Histone H2A type 2-C OS=Mus musculus OX=10090 GN=Hist2h2ac PE=1 SV=3 |
| 67 | 779 | B2RWH3\|B2RWH3_MOUSE | 35.71 | 7 | 7 | 2.58E+06 | 1 | 1 | 2 |  | 14095 | Histone H2A OS=Mus musculus OX=10090 GN=Hist2h2aa1 PE=2 SV=1 |
| 67 | 780 | sp\|C0HKE1\|H2A1B_MOUSE | 35.71 | 7 | 7 | 2.58E+06 | 1 | 1 | 2 |  | 14135 | Histone H2A type 1-B OS=Mus musculus OX=10090 GN=H2ac4 PE=1 SV=1 |
| 67 | 781 | sp\|Q6GSS7\|H2A2A_MOUSE | 35.71 | 7 | 7 | 2.58E+06 | 1 | 1 | 2 |  | 14095 | Histone H2A type 2-A OS=Mus musculus OX=10090 GN=Hist2h2aa1 PE=1 SV=3 |
| 67 | 782 | sp\|Q8BFU2\|H2A3_MOUSE | 35.71 | 7 | 7 | 2.58E+06 | 1 | 1 | 2 |  | 14121 | Histone H2A type 3 OS=Mus musculus OX=10090 GN=Hist3h2a PE=1 SV=3 |
| 67 | 783 | sp\|C0HKE9\|H2A1P_MOUSE | 35.71 | 7 | 7 | 2.58E+06 | 1 | 1 | 2 |  | 14135 | Histone H2A type 1-P OS=Mus musculus OX=10090 GN=Hist1h2ap PE=1 SV=1 |
| 67 | 784 | A2AB79\|A2AB79_MOUSE | 35.71 | 7 | 7 | 2.58E+06 | 1 | 1 | 2 |  | 14121 | Histone H2A OS=Mus musculus OX=10090 GN=H2aw PE=2 SV=1 |
| 67 | 785 | sp\|C0HKE7\|H2A1N_MOUSE | 35.71 | 7 | 7 | 2.58E+06 | 1 | 1 | 2 |  | 14135 | Histone H2A type 1-N OS=Mus musculus OX=10090 GN=Hist1h2an PE=1 SV=1 |
| 67 | 786 | sp\|Q64522\|H2A2B_MOUSE | 35.71 | 7 | 7 | 2.58E+06 | 1 | 1 | 2 |  | 14013 | Histone H2A type 2-B OS=Mus musculus OX=10090 GN=Hist2h2ab PE=1 SV=3 |
| 67 | 787 | sp\|C0HKE6\|H2A1I_MOUSE | 35.71 | 7 | 7 | 2.58E+06 | 1 | 1 | 2 |  | 14135 | Histone H2A type 1-I OS=Mus musculus OX=10090 GN=H2ac13 PE=1 SV=1 |
| 67 | 788 | B2RVF0\|B2RVF0_MOUSE | 35.71 | 7 | 7 | 2.58E+06 | 1 | 1 | 2 |  | 14135 | Histone H2A OS=Mus musculus OX=10090 GN=Hist1h2ad PE=2 SV=1 |
| 67 | 789 | sp\|C0HKE5\|H2A1G_MOUSE | 35.71 | 7 | 7 | 2.58E+06 | 1 | 1 | 2 |  | 14135 | Histone H2A type 1-G OS=Mus musculus OX=10090 GN=H2ac11 PE=1 SV=1 |
| 67 | 790 | sp\|C0HKE4\|H2A1E_MOUSE | 35.71 | 7 | 7 | 2.58E+06 | 1 | 1 | 2 |  | 14135 | Histone H2A type 1-E OS=Mus musculus OX=10090 GN=H2ac8 PE=1 SV=1 |
| 67 | 791 | sp\|Q8CGP7\|H2A1K_MOUSE | 35.71 | 7 | 7 | 2.58E+06 | 1 | 1 | 2 |  | 14150 | Histone H2A type 1-K OS=Mus musculus OX=10090 GN=H2ac15 PE=1 SV=3 |
| 67 | 792 | sp\|C0HKE2\|H2A1C_MOUSE | 35.71 | 7 | 7 | 2.58E+06 | 1 | 1 | 2 |  | 14135 | Histone H2A type 1-C OS=Mus musculus OX=10090 GN=Hist1h2ac PE=1 SV=1 |
| 67 | 793 | sp\|C0HKE3\|H2A1D_MOUSE | 35.71 | 7 | 7 | 2.58E+06 | 1 | 1 | 2 |  | 14135 | Histone H2A type 1-D OS=Mus musculus OX=10090 GN=H2ac7 PE=1 SV=1 |
| 67 | 794 | sp\|C0HKE8\|H2A1O_MOUSE | 35.71 | 7 | 7 | 2.58E+06 | 1 | 1 | 2 |  | 14135 | Histone H2A type 1-O OS=Mus musculus OX=10090 GN=Hist1h2ao PE=1 SV=1 |
| 67 | 795 | Q64426\|Q64426_MOUSE | 35.71 | 7 | 7 | 2.58E+06 | 1 | 1 | 2 |  | 14746 | Histone H2A (Fragment) OS=Mus musculus domesticus OX=10092 GN=H2A PE=2 SV=1 |
| 67 | 796 | sp\|P27661\|H2AX_MOUSE | 35.71 | 6 | 6 | 2.58E+06 | 1 | 1 | 2 |  | 15143 | Histone H2AX OS=Mus musculus OX=10090 GN=H2afx PE=1 SV=2 |
| 67 | 767 | G3UWL7\|G3UWL7_MOUSE | 35.71 | 10 | 10 | 2.58E+06 | 1 | 1 | 2 |  | 9118 | Histone H2A OS=Mus musculus OX=10090 GN=H2az1 PE=1 SV=1 |
| 67 | 768 | Q3UA95\|Q3UA95_MOUSE | 35.71 | 9 | 9 | 2.58E+06 | 1 | 1 | 2 |  | 10984 | Histone H2A OS=Mus musculus OX=10090 GN=H2az1 PE=2 SV=1 |
| 67 | 769 | A0A0N4SV66\|A0A0N4SV66_MOUSE | 35.71 | 7 | 7 | 2.58E+06 | 1 | 1 | 2 |  | 13660 | Histone H2A OS=Mus musculus OX=10090 GN=H2aj PE=1 SV=1 |
| 67 | 771 | sp\|Q3THW5\|H2AV_MOUSE | 35.71 | 7 | 7 | 2.58E+06 | 1 | 1 | 2 |  | 13509 | Histone H2A.V OS=Mus musculus OX=10090 GN=H2afv PE=1 SV=3 |
| 67 | 774 | B2RVP5\|B2RVP5_MOUSE | 35.71 | 7 | 7 | 2.58E+06 | 1 | 1 | 2 |  | 13509 | Histone H2A OS=Mus musculus OX=10090 GN=H2az2 PE=2 SV=1 |
| 67 | 775 | sp\|P0C0S6\|H2AZ_MOUSE | 35.71 | 7 | 7 | 2.58E+06 | 1 | 1 | 2 |  | 13553 | Histone H2A.Z OS=Mus musculus OX=10090 GN=H2az1 PE=1 SV=2 |
| 138 | 804 | A0A1L1SQA8\|A0A1L1SQA8_MOUSE | 35.5 | 11 | 11 | 0.00E+00 | 1 | 1 | 1 |  | 10309 | 40S ribosomal protein S25 OS=Mus musculus OX=10090 GN=Rps25 PE=1 SV=1 |
| 138 | 805 | Q58EA6\|Q58EA6_MOUSE | 35.5 | 8 | 8 | 0.00E+00 | 1 | 1 | 1 |  | 13742 | 40S ribosomal protein S25 OS=Mus musculus OX=10090 GN=Rps25 PE=1 SV=1 |
| 138 | 806 | sp\|P62852\|RS25_MOUSE | 35.5 | 8 | 8 | 0.00E+00 | 1 | 1 | 1 |  | 13742 | 40S ribosomal protein S25 OS=Mus musculus OX=10090 GN=Rps25 PE=1 SV=1 |
| 78 | 509 | A3E4B0\|A3E4B0_MOUSE | 33.82 | 3 | 3 | 3.47E+06 | 1 | 1 | 2 |  | 25946 | Cytochrome c oxidase subunit 2 OS=Mus musculus musculus OX=39442 GN=COX2 PE=3 SV=1 |
| 78 | 510 | A3R481\|A3R481_MOUSE | 33.82 | 3 | 3 | 3.47E+06 | 1 | 1 | 2 |  | 25975 | Cytochrome c oxidase subunit 2 OS=Mus musculus domesticus OX=10092 GN=COXII PE=3 SV=1 |
| 78 | 511 | A3R455\|A3R455_MUSMC | 33.82 | 3 | 3 | 3.47E+06 | 1 | 1 | 2 |  | 25976 | Cytochrome c oxidase subunit 2 OS=Mus musculus castaneus OX=10091 GN=COXII PE=3 SV=1 |
| 78 | 512 | Q7JCZ1\|Q7JCZ1_MOUSE | 33.82 | 3 | 3 | 3.47E+06 | 1 | 1 | 2 |  | 25976 | Cytochrome c oxidase subunit 2 OS=Mus musculus OX=10090 GN=mt-Co2 PE=1 SV=1 |
| 78 | 764 | A0A023J607\|A0A023J607_MUSMC | 33.82 | 3 | 3 | 3.47E+06 | 1 | 1 | 2 |  | 26006 | Cytochrome c oxidase subunit 2 OS=Mus musculus castaneus OX=10091 GN=COX2 PE=3 SV=1 |
| 78 | 513 | K7XK22\|K7XK22_MOUSE | 33.82 | 3 | 3 | 3.47E+06 | 1 | 1 | 2 |  | 25990 | Cytochrome c oxidase subunit 2 OS=Mus musculus domesticus OX=10092 GN=COXII PE=3 SV=1 |
| 78 | 514 | K7XKA7\|K7XKA7_MOUSE | 33.82 | 3 | 3 | 3.47E+06 | 1 | 1 | 2 |  | 25962 | Cytochrome c oxidase subunit 2 OS=Mus musculus domesticus OX=10092 GN=COXII PE=3 SV=1 |
| 78 | 515 | A0A023J6I7\|A0A023J6I7_MUSMC | 33.82 | 3 | 3 | 3.47E+06 | 1 | 1 | 2 |  | 25990 | Cytochrome c oxidase subunit 2 OS=Mus musculus castaneus OX=10091 GN=COX2 PE=3 SV=1 |
| 78 | 516 | Q7JD03\|Q7JD03_MOUSE | 33.82 | 3 | 3 | 3.47E+06 | 1 | 1 | 2 |  | 25976 | Cytochrome c oxidase subunit 2 OS=Mus musculus domesticus OX=10092 GN=COX2 PE=3 SV=1 |
| 78 | 517 | A0A075DC90\|A0A075DC90_MOUSE | 33.82 | 3 | 3 | 3.47E+06 | 1 | 1 | 2 |  | 26003 | Cytochrome c oxidase subunit 2 OS=Mus musculus OX=10090 GN=COX2 PE=3 SV=1 |
| 78 | 518 | sp\|P00405\|COX2_MOUSE | 33.82 | 3 | 3 | 3.47E+06 | 1 | 1 | 2 |  | 25976 | Cytochrome c oxidase subunit 2 OS=Mus musculus OX=10090 GN=Mtco2 PE=1 SV=1 |
| 78 | 519 | A0A0F6PXF3\|A0A0F6PXF3_MOUSE | 33.82 | 3 | 3 | 3.47E+06 | 1 | 1 | 2 |  | 25976 | Cytochrome c oxidase subunit 2 OS=Mus musculus helgolandicus OX=1643390 GN=COXII PE=3 SV=1 |
| 78 | 520 | Q5GA81\|Q5GA81_MUSMM | 33.82 | 3 | 3 | 3.47E+06 | 1 | 1 | 2 |  | 25976 | Cytochrome c oxidase subunit 2 OS=Mus musculus molossinus OX=57486 GN=COX2 PE=3 SV=1 |
| 78 | 521 | A0A023J6F3\|A0A023J6F3_MOUSE | 33.82 | 3 | 3 | 3.47E+06 | 1 | 1 | 2 |  | 25976 | Cytochrome c oxidase subunit 2 OS=Mus musculus musculus OX=39442 GN=COX2 PE=3 SV=1 |
| 78 | 765 | A0A023J5Y1\|A0A023J5Y1_MOUSE | 33.82 | 3 | 3 | 3.47E+06 | 1 | 1 | 2 |  | 26006 | Cytochrome c oxidase subunit 2 OS=Mus musculus musculus OX=39442 GN=COX2 PE=3 SV=1 |
| 140 | 842 | A6H622\|A6H622_MOUSE | 31.14 | 5 | 5 | 6.01E+05 | 1 | 1 | 1 | Acetylation (Protein N-term) | 24452 | GTP-binding nuclear protein Ran OS=Mus musculus OX=10090 GN=Rasl2-9 PE=2 SV=1 |
| 140 | 493 | Q3ULW0\|Q3ULW0_MOUSE | 31.14 | 5 | 5 | 6.01E+05 | 1 | 1 | 1 | Acetylation (Protein N-term) | 24351 | GTP-binding nuclear protein Ran OS=Mus musculus OX=10090 GN=Ran PE=2 SV=1 |
| 140 | 843 | sp\|Q61820\|RANT_MOUSE | 31.14 | 5 | 5 | 6.01E+05 | 1 | 1 | 1 | Acetylation (Protein N-term) | 24452 | GTP-binding nuclear protein Ran testis-specific isoform OS=Mus musculus OX=10090 GN=Rasl2-9 PE=2 SV=1 |
| 140 | 494 | sp\|P62827\|RAN_MOUSE | 31.14 | 5 | 5 | 6.01E+05 | 1 | 1 | 1 | Acetylation (Protein N-term) | 24423 | GTP-binding nuclear protein Ran OS=Mus musculus OX=10090 GN=Ran PE=1 SV=3 |
| 127 | 585 | I6L958\|I6L958_MOUSE | 29.74 | 15 | 15 | 2.70E+06 | 1 | 1 | 1 | Carbamidomethylation | 25645 | Igk protein OS=Mus musculus OX=10090 GN=Igk PE=1 SV=1 |
| 127 | 562 | A2P1G9\|A2P1G9_MOUSE | 29.74 | 16 | 16 | 2.70E+06 | 1 | 1 | 1 | Carbamidomethylation | 24086 | Kappa light chain (Fragment) OS=Mus musculus OX=10090 GN=Igkc PE=1 SV=1 |
| 127 | 563 | A2NHM3\|A2NHM3_MOUSE | 29.74 | 16 | 16 | 2.70E+06 | 1 | 1 | 1 | Carbamidomethylation | 24165 | If kappa light chain (Fragment) OS=Mus musculus OX=10090 GN=Igkc PE=1 SV=1 |
| 127 | 564 | Q65ZC0\|Q65ZC0_MOUSE | 29.74 | 16 | 16 | 2.70E+06 | 1 | 1 | 1 | Carbamidomethylation | 23945 | Kappa light chain C_region (Fragment) OS=Mus musculus OX=10090 PE=1 SV=1 |
| 127 | 565 | A0A0D5ZY64\|A0A0D5ZY64_MOUSE | 29.74 | 14 | 14 | 2.70E+06 | 1 | 1 | 1 | Carbamidomethylation | 26293 | IgE L chain kappa OS=Mus musculus OX=10090 PE=2 SV=1 |
| 127 | 566 | A0A125T908\|A0A125T908_MOUSE | 29.74 | 14 | 14 | 2.70E+06 | 1 | 1 | 1 | Carbamidomethylation | 26307 | Light chain kappa OS=Mus musculus OX=10090 GN=Igk PE=2 SV=1 |
| 127 | 567 | Q58EU8\|Q58EU8_MOUSE | 29.74 | 14 | 14 | 2.70E+06 | 1 | 1 | 1 | Carbamidomethylation | 26302 | Igk protein OS=Mus musculus OX=10090 GN=Igk PE=1 SV=1 |
| 127 | 661 | A0A4U9FD83\|A0A4U9FD83_MOUSE | 29.74 | 32 | 32 | 2.70E+06 | 1 | 1 | 1 | Carbamidomethylation | 11778 | IgK (Fragment) OS=Mus musculus OX=10090 GN=Igkc PE=4 SV=1 |
| 127 | 660 | sp\|P01837\|IGKC_MOUSE | 29.74 | 32 | 32 | 2.70E+06 | 1 | 1 | 1 | Carbamidomethylation | 11934 | Immunoglobulin kappa constant OS=Mus musculus OX=10090 GN=Igkc PE=1 SV=2 |
| 127 | 662 | A0A0M3KL49\|A0A0M3KL49_MOUSE | 29.74 | 16 | 16 | 2.70E+06 | 1 | 1 | 1 | Carbamidomethylation | 24086 | Fab4201 heavy chain OS=Mus musculus OX=10090 PE=1 SV=1 |
| 127 | 663 | I6L978\|I6L978_MOUSE | 29.74 | 15 | 15 | 2.70E+06 | 1 | 1 | 1 | Carbamidomethylation | 25703 | Igk protein OS=Mus musculus OX=10090 GN=Igk PE=1 SV=1 |
| 127 | 664 | A0A0M4KEQ7\|A0A0M4KEQ7_MOUSE | 29.74 | 15 | 15 | 2.70E+06 | 1 | 1 | 1 | Carbamidomethylation | 25950 | Monoclonal 11D8 anti-human butyrylcholinesterase (BChE) light chain OS=Mus musculus OX=10090 PE=2 SV=1 |
| 127 | 665 | A0A0C6EL31\|A0A0C6EL31_MOUSE | 29.74 | 15 | 15 | 2.70E+06 | 1 | 1 | 1 | Carbamidomethylation | 26023 | LC protein OS=Mus musculus OX=10090 GN=LC PE=2 SV=1 |
| 127 | 666 | A0A0U5BC76\|A0A0U5BC76_MOUSE | 29.74 | 15 | 15 | 2.70E+06 | 1 | 1 | 1 | Carbamidomethylation | 26046 | MAb 6H10 light chain OS=Mus musculus OX=10090 GN=LC PE=2 SV=1 |
| 127 | 667 | A0A0B6VQ20\|A0A0B6VQ20_MOUSE | 29.74 | 15 | 15 | 2.70E+06 | 1 | 1 | 1 | Carbamidomethylation | 25855 | MAb 31C6 light chain OS=Mus musculus OX=10090 GN=LC PE=4 SV=1 |
| 127 | 668 | I6L991\|I6L991_MOUSE | 29.74 | 15 | 15 | 2.70E+06 | 1 | 1 | 1 | Carbamidomethylation | 25858 | Uncharacterized protein OS=Mus musculus OX=10090 PE=1 SV=1 |
| 127 | 669 | A0A125T904\|A0A125T904_MOUSE | 29.74 | 15 | 15 | 2.70E+06 | 1 | 1 | 1 | Carbamidomethylation | 25703 | Light chain kappa OS=Mus musculus OX=10090 GN=Igk PE=2 SV=1 |
| 127 | 670 | I6L9E2\|I6L9E2_MOUSE | 29.74 | 14 | 14 | 2.70E+06 | 1 | 1 | 1 | Carbamidomethylation | 25861 | Uncharacterized protein OS=Mus musculus OX=10090 PE=2 SV=1 |
| 127 | 671 | Q5XFY8\|Q5XFY8_MOUSE | 29.74 | 14 | 14 | 2.70E+06 | 1 | 1 | 1 | Carbamidomethylation | 25835 | Uncharacterized protein OS=Mus musculus OX=10090 PE=1 SV=1 |
| 127 | 672 | I6L9E1\|I6L9E1_MOUSE | 29.74 | 14 | 14 | 2.70E+06 | 1 | 1 | 1 | Carbamidomethylation | 26299 | Uncharacterized protein OS=Mus musculus OX=10090 PE=2 SV=1 |
| 127 | 673 | Q52L95\|Q52L95_MOUSE | 29.74 | 14 | 14 | 2.70E+06 | 1 | 1 | 1 | Carbamidomethylation | 26446 | Igk protein OS=Mus musculus OX=10090 GN=Igk PE=2 SV=1 |
| 127 | 674 | Q7TS98\|Q7TS98_MOUSE | 29.74 | 14 | 14 | 2.70E+06 | 1 | 1 | 1 | Carbamidomethylation | 26455 | Anti-colorectal carcinoma light chain OS=Mus musculus OX=10090 PE=1 SV=1 |
| 127 | 675 | A0A5E3\|A0A5E3_MOUSE | 29.74 | 14 | 14 | 2.70E+06 | 1 | 1 | 1 | Carbamidomethylation | 26044 | LOC100046793 protein OS=Mus musculus OX=10090 GN=LOC100046793 PE=1 SV=1 |
| 127 | 676 | A0A0E4B213\|A0A0E4B213_MOUSE | 29.74 | 14 | 14 | 2.70E+06 | 1 | 1 | 1 | Carbamidomethylation | 26257 | MAb 44B1 light chain OS=Mus musculus OX=10090 GN=LC PE=2 SV=1 |
| 127 | 677 | A0A0F7R1B7\|A0A0F7R1B7_MOUSE | 29.74 | 14 | 14 | 2.70E+06 | 1 | 1 | 1 | Carbamidomethylation | 26337 | MAb 106 light chain OS=Mus musculus OX=10090 GN=LC PE=2 SV=1 |
| 127 | 678 | A0A0F7R5U8\|A0A0F7R5U8_MOUSE | 29.74 | 14 | 14 | 2.70E+06 | 1 | 1 | 1 | Carbamidomethylation | 26590 | MAb 110 light chain OS=Mus musculus OX=10090 GN=LC PE=2 SV=1 |
| 127 | 679 | Q52L64\|Q52L64_MOUSE | 29.74 | 14 | 14 | 2.70E+06 | 1 | 1 | 1 | Carbamidomethylation | 26609 | ENSMUSG00000076577 protein OS=Mus musculus OX=10090 GN=Igkv8-30 PE=1 SV=1 |
| 141 | 12757 | G3X9K0\|G3X9K0_MOUSE | 29 | 2 | 2 | 0.00E+00 | 1 | 1 | 1 |  | 42783 | Lysophosphatidic acid receptor 5 OS=Mus musculus OX=10090 GN=Lpar5 PE=1 SV=1 |
| 143 | 891 | F6V9F1\|F6V9F1_MOUSE | 27.92 | 4 | 4 | 7.17E+05 | 1 | 1 | 1 |  | 18942 | Protein-L-isoaspartate(D-aspartate) O-methyltransferase (Fragment) OS=Mus musculus OX=10090 GN=Pcmt1 PE=1 SV=1 |
| 143 | 892 | Q8BPI6\|Q8BPI6_MOUSE | 27.92 | 3 | 3 | 7.17E+05 | 1 | 1 | 1 |  | 22416 | Protein-L-isoaspartate O-methyltransferase (Fragment) OS=Mus musculus OX=10090 GN=Pcmt1 PE=2 SV=1 |
| 143 | 893 | Q545L9\|Q545L9_MOUSE | 27.92 | 3 | 3 | 7.17E+05 | 1 | 1 | 1 |  | 24634 | Protein-L-isoaspartate O-methyltransferase OS=Mus musculus OX=10090 GN=Pcmt1 PE=2 SV=1 |
| 143 | 894 | sp\|P23506\|PIMT_MOUSE | 27.92 | 3 | 3 | 7.17E+05 | 1 | 1 | 1 |  | 24634 | Protein-L-isoaspartate(D-aspartate) O-methyltransferase OS=Mus musculus OX=10090 GN=Pcmt1 PE=1 SV=3 |
| 143 | 896 | F7D432\|F7D432_MOUSE | 27.92 | 3 | 3 | 7.17E+05 | 1 | 1 | 1 |  | 29168 | Protein-L-isoaspartate O-methyltransferase (Fragment) OS=Mus musculus OX=10090 GN=Pcmt1 PE=1 SV=1 |
| 143 | 897 | E9PWE0\|E9PWE0_MOUSE | 27.92 | 2 | 2 | 7.17E+05 | 1 | 1 | 1 |  | 30398 | Protein-L-isoaspartate O-methyltransferase OS=Mus musculus OX=10090 GN=Pcmt1 PE=1 SV=1 |
| 143 | 898 | E0CYV0\|E0CYV0_MOUSE | 27.92 | 2 | 2 | 7.17E+05 | 1 | 1 | 1 |  | 30441 | Protein-L-isoaspartate O-methyltransferase OS=Mus musculus OX=10090 GN=Pcmt1 PE=1 SV=1 |
| 142 | 337 | A0JLV3\|A0JLV3_MOUSE | 27.43 | 7 | 7 | 3.67E+06 | 1 | 1 | 1 |  | 13579 | Histone H2B (Fragment) OS=Mus musculus OX=10090 GN=Hist1h2bj PE=2 SV=1 |
| 142 | 496 | sp\|Q8CGP0\|H2B3B_MOUSE | 27.43 | 7 | 7 | 3.67E+06 | 1 | 1 | 1 |  | 13908 | Histone H2B type 3-B OS=Mus musculus OX=10090 GN=Hist3h2bb PE=1 SV=3 |
| 142 | 338 | B2RVD5\|B2RVD5_MOUSE | 27.43 | 7 | 7 | 3.67E+06 | 1 | 1 | 1 |  | 13920 | Histone H2B OS=Mus musculus OX=10090 GN=H2bc12 PE=2 SV=1 |
| 142 | 497 | sp\|Q64524\|H2B2E_MOUSE | 27.43 | 7 | 7 | 3.67E+06 | 1 | 1 | 1 |  | 13993 | Histone H2B type 2-E OS=Mus musculus OX=10090 GN=Hist2h2be PE=1 SV=3 |
| 142 | 339 | B2RTK3\|B2RTK3_MOUSE | 27.43 | 7 | 7 | 3.67E+06 | 1 | 1 | 1 |  | 13936 | Histone H2B OS=Mus musculus OX=10090 GN=H2bc14 PE=1 SV=1 |
| 142 | 340 | sp\|P10854\|H2B1M_MOUSE | 27.43 | 7 | 7 | 3.67E+06 | 1 | 1 | 1 |  | 13936 | Histone H2B type 1-M OS=Mus musculus OX=10090 GN=H2bc14 PE=1 SV=2 |
| 142 | 341 | sp\|Q6ZWY9\|H2B1C_MOUSE | 27.43 | 7 | 7 | 3.67E+06 | 1 | 1 | 1 |  | 13906 | Histone H2B type 1-C/E/G OS=Mus musculus OX=10090 GN=H2bc4 PE=1 SV=3 |
| 142 | 342 | sp\|Q64475\|H2B1B_MOUSE | 27.43 | 7 | 7 | 3.67E+06 | 1 | 1 | 1 |  | 13952 | Histone H2B type 1-B OS=Mus musculus OX=10090 GN=Hist1h2bb PE=1 SV=3 |
| 142 | 343 | sp\|Q8CGP2\|H2B1P_MOUSE | 27.43 | 7 | 7 | 3.67E+06 | 1 | 1 | 1 |  | 13992 | Histone H2B type 1-P OS=Mus musculus OX=10090 GN=Hist1h2bp PE=1 SV=3 |
| 142 | 344 | sp\|Q64478\|H2B1H_MOUSE | 27.43 | 7 | 7 | 3.67E+06 | 1 | 1 | 1 |  | 13920 | Histone H2B type 1-H OS=Mus musculus OX=10090 GN=Hist1h2bh PE=1 SV=3 |
| 142 | 498 | sp\|Q9D2U9\|H2B3A_MOUSE | 27.43 | 7 | 7 | 3.67E+06 | 1 | 1 | 1 |  | 13994 | Histone H2B type 3-A OS=Mus musculus OX=10090 GN=Hist3h2ba PE=1 SV=3 |
| 142 | 345 | sp\|Q64525\|H2B2B_MOUSE | 27.43 | 7 | 7 | 3.67E+06 | 1 | 1 | 1 |  | 13920 | Histone H2B type 2-B OS=Mus musculus OX=10090 GN=Hist2h2bb PE=1 SV=3 |
| 142 | 346 | sp\|P10853\|H2B1F_MOUSE | 27.43 | 7 | 7 | 3.67E+06 | 1 | 1 | 1 |  | 13936 | Histone H2B type 1-F/J/L OS=Mus musculus OX=10090 GN=H2bc7 PE=1 SV=2 |
| 142 | 347 | sp\|Q8CGP1\|H2B1K_MOUSE | 27.43 | 7 | 7 | 3.67E+06 | 1 | 1 | 1 |  | 13920 | Histone H2B type 1-K OS=Mus musculus OX=10090 GN=H2bc12 PE=1 SV=3 |
| 142 | 348 | sp\|P70696\|H2B1A_MOUSE | 27.43 | 7 | 7 | 3.67E+06 | 1 | 1 | 1 |  | 14237 | Histone H2B type 1-A OS=Mus musculus OX=10090 GN=H2bc1 PE=1 SV=3 |
| 142 | 349 | A0JNS9\|A0JNS9_MOUSE | 27.43 | 7 | 7 | 3.67E+06 | 1 | 1 | 1 |  | 14179 | Histone H2B OS=Mus musculus OX=10090 GN=H2bc1 PE=2 SV=1 |
| 142 | 350 | Q8CBB6\|Q8CBB6_MOUSE | 27.43 | 7 | 7 | 3.67E+06 | 1 | 1 | 1 |  | 14888 | Histone H2B OS=Mus musculus OX=10090 GN=Hist1h2bq PE=2 SV=1 |
| 142 | 351 | Q921L4\|Q921L4_MOUSE | 27.43 | 7 | 7 | 3.67E+06 | 1 | 1 | 1 |  | 14939 | Histone H2B OS=Mus musculus OX=10090 GN=LOC665622 PE=2 SV=1 |
| 144 | 2363 | sp\|Q61647\|HYAS1_MOUSE | 27.26 | 1 | 1 | 5.33E+05 | 1 | 1 | 1 |  | 65545 | Hyaluronan synthase 1 OS=Mus musculus OX=10090 GN=Has1 PE=1 SV=1 |
| 144 | 2364 | Q05A37\|Q05A37_MOUSE | 27.26 | 1 | 1 | 5.33E+05 | 1 | 1 | 1 |  | 65559 | Hyaluronan synthase1 OS=Mus musculus OX=10090 GN=Has1 PE=2 SV=1 |
| 129 | 611 | Q66K04\|Q66K04_MOUSE | 26.61 | 5 | 5 | 2.35E+06 | 1 | 1 | 1 | Deamidation (NQ) | 52259 | Igh protein OS=Mus musculus OX=10090 GN=Igh PE=2 SV=1 |
| 58 | 873 | A2AQD6\|A2AQD6_MOUSE | 25.99 | 1 | 1 | 3.61E+06 | 1 | 1 | 3 |  | 133462 | Protein ITPRID2 OS=Mus musculus OX=10090 GN=Itprid2 PE=1 SV=1 |
| 58 | 874 | A2AQD5\|A2AQD5_MOUSE | 25.99 | 1 | 1 | 3.61E+06 | 1 | 1 | 3 |  | 134671 | Protein ITPRID2 OS=Mus musculus OX=10090 GN=Itprid2 PE=1 SV=1 |
| 58 | 875 | sp\|Q922B9\|ITPI2_MOUSE | 25.99 | 1 | 1 | 3.61E+06 | 1 | 1 | 3 |  | 136947 | Protein ITPRID2 OS=Mus musculus OX=10090 GN=Itprid2 PE=1 SV=3 |
| 145 | 939 | Q3TD78\|Q3TD78_MOUSE | 25.4 | 2 | 2 | 7.12E+05 | 1 | 1 | 1 |  | 32871 | NIPSNAP domain-containing protein OS=Mus musculus OX=10090 GN=Nipsnap2 PE=2 SV=1 |
| 145 | 12766 | Q3UGS1\|Q3UGS1_MOUSE | 25.4 | 2 | 2 | 7.12E+05 | 1 | 1 | 1 |  | 37535 | NIPSNAP domain-containing protein OS=Mus musculus OX=10090 GN=Nipsnap1 PE=2 SV=1 |
| 149 | 1012 | Q3TKB9\|Q3TKB9_MOUSE | 22.24 | 2 | 2 | 6.38E+05 | 1 | 1 | 1 |  | 64738 | HATPase_c domain-containing protein (Fragment) OS=Mus musculus OX=10090 GN=Hsp90aa1 PE=2 SV=1 |
| 149 | 12776 | A0PJ91\|A0PJ91_MOUSE | 22.24 | 2 | 2 | 6.38E+05 | 1 | 1 | 1 |  | 65894 | Hsp90aa1 protein (Fragment) OS=Mus musculus OX=10090 GN=Hsp90aa1 PE=2 SV=1 |
| 149 | 12777 | Q3TKA2\|Q3TKA2_MOUSE | 22.24 | 2 | 2 | 6.38E+05 | 1 | 1 | 1 |  | 84789 | HATPase_c domain-containing protein OS=Mus musculus OX=10090 GN=Hsp90aa1 PE=2 SV=1 |
| 149 | 12778 | Q80Y52\|Q80Y52_MOUSE | 22.24 | 2 | 2 | 6.38E+05 | 1 | 1 | 1 |  | 84788 | Heat shock protein 90 alpha (Cytosolic) class A member 1 OS=Mus musculus OX=10090 GN=Hsp90aa1 PE=1 SV=2 |
| 70 | 12795 | sp\|Q8CI78\|RMND1_MOUSE | 22.21 | 1 | 1 | 1.01E+08 | 1 | 1 | 2 |  | 51843 | Required for meiotic nuclear division protein 1 homolog OS=Mus musculus OX=10090 GN=Rmnd1 PE=1 SV=1 |
| 70 | 1177 | Q3USI2\|Q3USI2_MOUSE | 22.21 | 0 | 0 | 1.01E+08 | 1 | 1 | 2 |  | 150818 | Uncharacterized protein (Fragment) OS=Mus musculus OX=10090 GN=Lamb2 PE=2 SV=1 |
| 70 | 1033 | F6ZZB1\|F6ZZB1_MOUSE | 22.21 | 1 | 1 | 1.01E+08 | 1 | 1 | 2 |  | 119656 | Neurobeachin-like protein 2 (Fragment) OS=Mus musculus OX=10090 GN=Nbeal2 PE=1 SV=1 |
| 70 | 12921 | F6VTL9\|F6VTL9_MOUSE | 22.21 | 0 | 0 | 1.01E+08 | 1 | 1 | 2 |  | 223702 | Neurobeachin-like protein 2 (Fragment) OS=Mus musculus OX=10090 GN=Nbeal2 PE=1 SV=1 |
| 70 | 1273 | A0A0G2JFQ4\|A0A0G2JFQ4_MOUSE | 22.21 | 0 | 0 | 1.01E+08 | 1 | 1 | 2 |  | 299042 | Neurobeachin-like protein 2 OS=Mus musculus OX=10090 GN=Nbeal2 PE=1 SV=1 |
| 70 | 6186 | B7ZWL5\|B7ZWL5_MOUSE | 22.21 | 0 | 0 | 1.01E+08 | 1 | 1 | 2 |  | 299749 | Nbeal2 protein OS=Mus musculus OX=10090 GN=Nbeal2 PE=2 SV=1 |
| 70 | 1283 | B2RXS1\|B2RXS1_MOUSE | 22.21 | 0 | 0 | 1.01E+08 | 1 | 1 | 2 |  | 302718 | Nbeal2 protein OS=Mus musculus OX=10090 GN=Nbeal2 PE=2 SV=1 |
| 131 | 979 | Q3U5D0\|Q3U5D0_MOUSE | 21.96 | 2 | 2 | 6.48E+07 | 1 | 1 | 1 |  | 46981 | Spermatogenesis-associated protein 7 homolog OS=Mus musculus OX=10090 GN=Spata7 PE=2 SV=1 |
| 131 | 983 | Q3TTL3\|Q3TTL3_MOUSE | 21.96 | 1 | 1 | 6.48E+07 | 1 | 1 | 1 |  | 62081 | SPATA7 isoform OS=Mus musculus OX=10090 GN=Spata7 PE=2 SV=1 |
| 81 | 710 | sp\|P06329\|HVM50_MOUSE | 21.67 | 21 | 21 | 2.64E+06 | 1 | 1 | 1 | Carbamidomethylation; Oxidation (M) | 13311 | Ig heavy chain V region AC38 15.3 OS=Mus musculus OX=10090 PE=1 SV=1 |
| 130 | 2745 | sp\|Q8C3I8\|HGH1_MOUSE | 21.49 | 2 | 2 | 9.76E+06 | 1 | 1 | 1 |  | 42916 | Protein HGH1 homolog OS=Mus musculus OX=10090 GN=Hgh1 PE=1 SV=1 |
| 24 | 6573 | sp\|Q61235\|SNTB2_MOUSE | 20.77 | 1 | 1 | 2.39E+07 | 1 | 1 | 1 |  | 56382 | Beta-2-syntrophin OS=Mus musculus OX=10090 GN=Sntb2 PE=1 SV=2 |
| 24 | 1674 | A0A140LIQ1\|A0A140LIQ1_MOUSE | 20.77 | 1 | 1 | 2.39E+07 | 1 | 1 | 1 |  | 62404 | Transforming acidic coiled-coil-containing protein 2 OS=Mus musculus OX=10090 GN=Tacc2 PE=1 SV=1 |
| 24 | 1693 | A0A140LIJ2\|A0A140LIJ2_MOUSE | 20.77 | 1 | 1 | 2.39E+07 | 1 | 1 | 1 |  | 63732 | Transforming acidic coiled-coil-containing protein 2 OS=Mus musculus OX=10090 GN=Tacc2 PE=1 SV=1 |
| 24 | 1991 | A0A140LJ29\|A0A140LJ29_MOUSE | 20.77 | 1 | 1 | 2.39E+07 | 1 | 1 | 1 |  | 77503 | Transforming acidic coiled-coil-containing protein 2 (Fragment) OS=Mus musculus OX=10090 GN=Tacc2 PE=1 SV=1 |
| 24 | 2346 | A0A140LIQ6\|A0A140LIQ6_MOUSE | 20.77 | 1 | 1 | 2.39E+07 | 1 | 1 | 1 |  | 91053 | Transforming acidic coiled-coil-containing protein 2 (Fragment) OS=Mus musculus OX=10090 GN=Tacc2 PE=1 SV=1 |
| 24 | 3079 | Q3UL40\|Q3UL40_MOUSE | 20.77 | 0 | 0 | 2.39E+07 | 1 | 1 | 1 |  | 112182 | TACC_C domain-containing protein (Fragment) OS=Mus musculus OX=10090 GN=Tacc2 PE=2 SV=1 |
| 24 | 3086 | E9QL08\|E9QL08_MOUSE | 20.77 | 0 | 0 | 2.39E+07 | 1 | 1 | 1 |  | 112936 | Transforming acidic coiled-coil-containing protein 2 OS=Mus musculus OX=10090 GN=Tacc2 PE=1 SV=1 |
| 24 | 3502 | E9Q9Z4\|E9Q9Z4_MOUSE | 20.77 | 0 | 0 | 2.39E+07 | 1 | 1 | 1 |  | 123931 | Transforming acidic coiled-coil-containing protein 2 OS=Mus musculus OX=10090 GN=Tacc2 PE=1 SV=1 |
| 24 | 3514 | sp\|Q9JJG0\|TACC2_MOUSE | 20.77 | 0 | 0 | 2.39E+07 | 1 | 1 | 1 |  | 124130 | Transforming acidic coiled-coil-containing protein 2 OS=Mus musculus OX=10090 GN=Tacc2 PE=1 SV=2 |
| 24 | 1978 | sp\|Q6PAC4\|PCARE_MOUSE | 20.77 | 0 | 0 | 2.39E+07 | 1 | 1 | 1 |  | 139297 | Photoreceptor cilium actin regulator OS=Mus musculus OX=10090 GN=Pcare PE=2 SV=1 |
| 24 | 5823 | E9Q8T1\|E9Q8T1_MOUSE | 20.77 | 0 | 0 | 2.39E+07 | 1 | 1 | 1 |  | 305230 | Transforming acidic coiled-coil-containing protein 2 OS=Mus musculus OX=10090 GN=Tacc2 PE=1 SV=1 |
