# Supplementary material for: Tert promotes cardiac regenerative repair after MI through alleviating ROS-induced DNA damage response in cardiomyocyte
Source: Cell Death Discov. 2024 Aug 26;10:381. doi: 10.1038/s41420-024-02135-8 (PMC11347641; doi:10.1038/s41420-024-02135-8)

Supplementary figures and figure legends

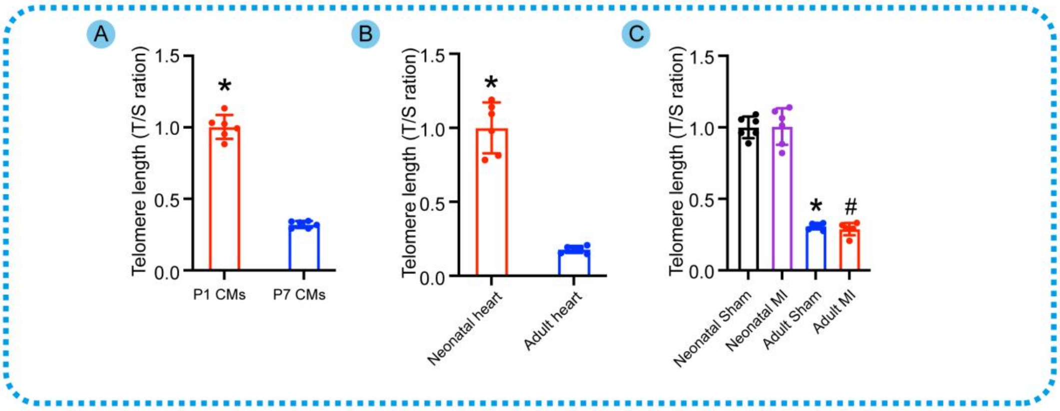

Supplemental Figure 1: Figures related to Figure 1.

A: The telomere lengths of CMs derived from P1 and P7 mice (*P <0.05; n=6 cell samples per group). **B**: The telomere lengths of heart tissues from neonatal and adult

mice (*P <0.05; n=6 heart samples per group). **C**: Telomere lengths of cardiac tissues in post-infarct hearts from neonatal and adult mice of both Sham and MI groups (*P <0.05 vs. Neonatal Sham group;#P <0.05 vs. Neonatal MI group; n=6 heart samples per group). Error bars indicate SD of six biological repeats. An unpaired *t*-test in **A-B,** and one-way ANOVA in **C** were utilized to determine the statistical significance.

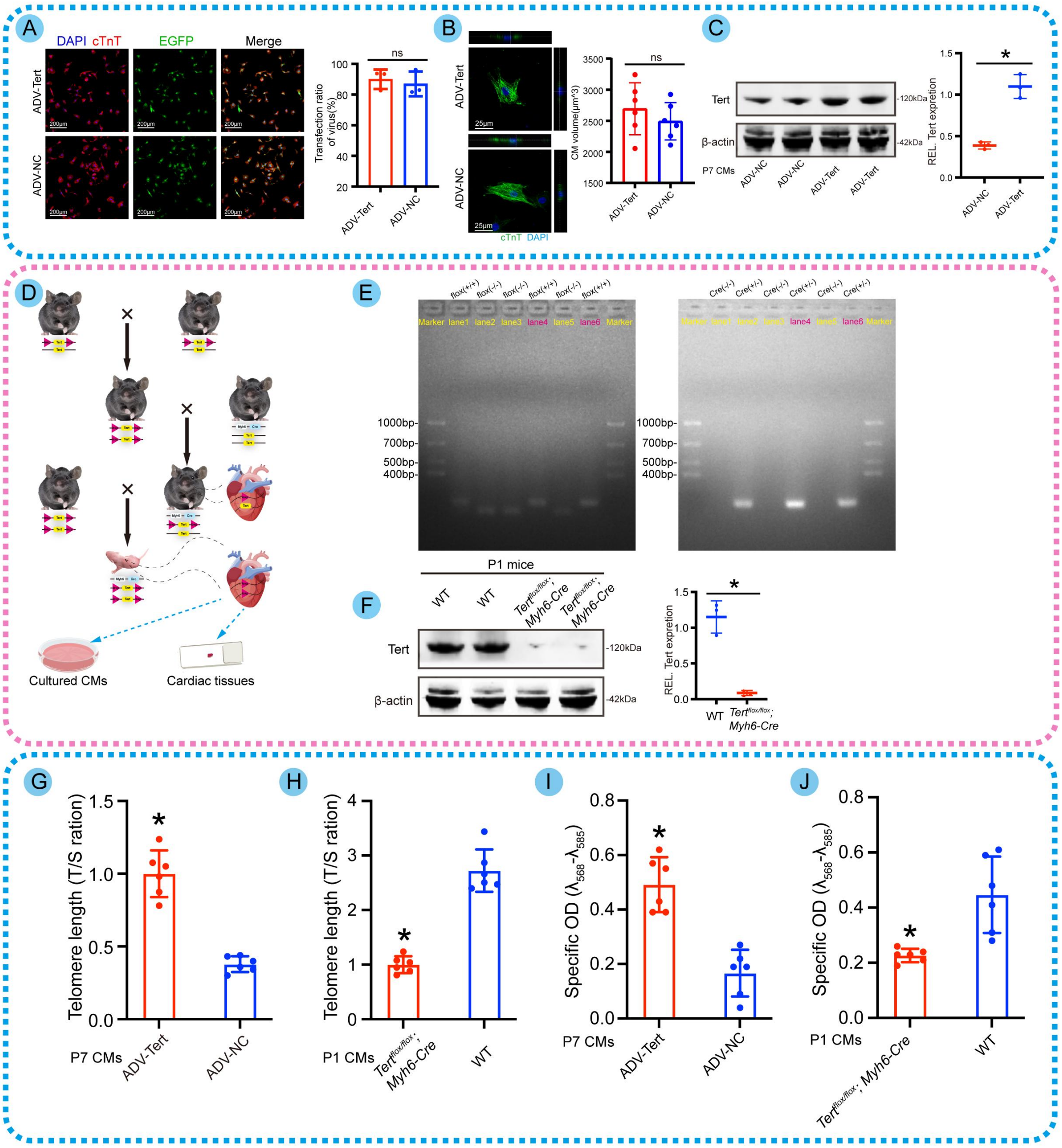

Supplemental Figure 2: Figures related to Figure 2.

A: Immunofluorescence labeling of EGFP co-localized with CMs and quantitative analysis to assess transfection efficiency of overexpression vectors in vitro (ns P>0.05; 139 CMs from 6 images of 3 mice in ADV-Tert group; 158 CMs from 6

images of 3 mice in ADV-NC group). **B:** Immunofluorescence labeling of cTnT and quantitative analysis to determine the effects of Tert overexpression on CM size (ns P>0.05; 18 CMs from 6 images of 3 mice in ADV-Tert group; 18 CMs from 6 images of 3 mice in ADV-NC group). **C:** Western blotting assay for detection of Tert in P7 CMs with Tert overexpression to determine the intervention efficiency of the virus (*P <0.05; n=3 cell samples per group, the experiment was replicated in the laboratory a total of three times). **D:** Construction strategy flow of heart-specific Tert-deletion mice. **E:** Agarose gel electrophoresis plot for identification of genotype to confirm successful establishment of heart-specific deletion mice. Samples in the same numbered lanes are identical in both left and right panels. As the left and right panel exhibited that the genotype both flox(+/+) and Cre(+/-) were shown in lane 4 and 6, which indicated that lane 4 and 6 correspond to heart-specific Tert-deletion mice. **F:** Western blotting assay for detection of Tert in hearts from wild-type and heart-specific Tert-deletion mice (*P <0.05; n=3 mice per group). **G** and **H**: Telomere lengths in P7 CMs with Tert overexpression and P1 CMs isolated from WT or $Tert^{flox/flox}$; *Myh6*-Cre mice (*P <0.05 vs. ADV-NC group or WT group; n=6 cell samples per group). **I** and **J**: Assessment of Edu in P7 CMs with Tert overexpression and P1 CMs isolated from WT or $Tert^{flox/flox}$ mice (*P <0.05 vs. ADV-NC or WT; n=6 cell samples per group). Error bars indicate SD of three biological repeats in **A/C/F** and that of six biological repeats in **B/G-J**. An unpaired *t*-test in **A-C/F-J** was utilized to determine the statistical significance.

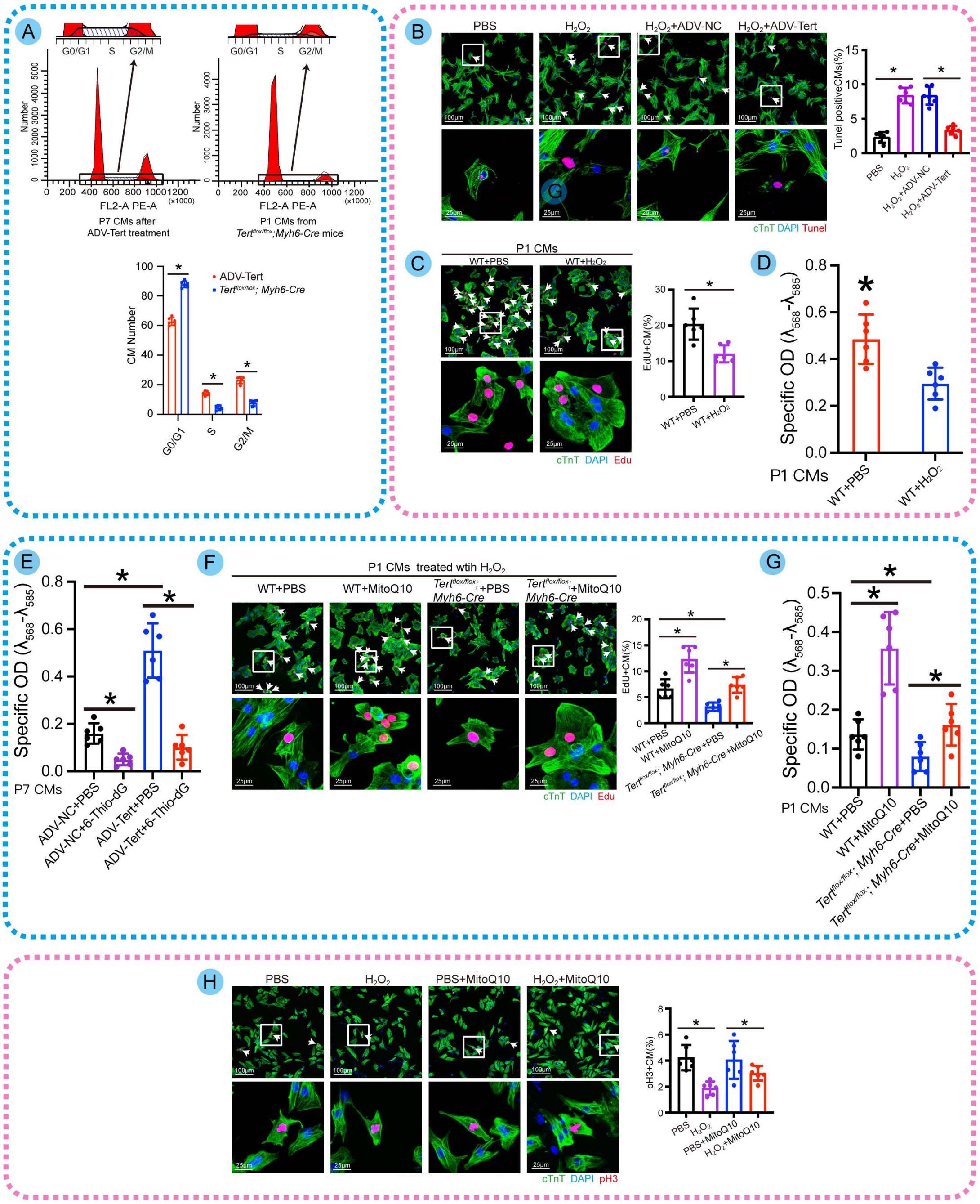

Supplemental Figure 3: Figures related to Figure 2.

A: Flow cytometry assay and quantitative analysis to determine the cell numbers in different phases of the cell cycle. P7 CMs were treated with Tert overexpression and P1 CMs were isolated from *Tert*$^{flox/flox}$*; Myh6*-Cre mice. The bottom panel is the

quantitative statistical plots(*P <0.05; n=6 cell samples per group). **B:** Immunofluorescence labeling of tunel to assess the effects of Tert overexpression on CM apoptosis (tunel+ CMs are indicated by arrows, *P <0.05; 118 CMs from 18 images of 6 mice in PBS group; 198 CMs from 18 images of 6 mice in H_2O_2 group; 203 CMs from 18 images of 6 mice in H_2O_2+ADV-NC group; 133 CMs from 18 images of 6 mice in H_2O_2+ADV-Tert group). **C:** Immunofluorescence images of Edu in wild-type P1 CMs to assess the effect of H_2O_2 treatment on CM proliferation (Edu+ CMs are indicated by arrows, *P <0.05; 311 CMs from 18 images of 6 mice in WT+PBS group; 294 CMs from 18 images of 6 mice in WT+H_2O_2 group). **D**: Assessment of Edu in wild-type P1 CMs to assess the effect of H_2O_2 treatment on CM proliferation (*P <0.05 vs. WT+PBS; n=6 cell samples per group). **E**: Assessment of Edu in H_2O_2-treated P7 CMs with different treatments detected by microplates assay (*P <0.05; n=6 cell samples per group). **F:** Immunofluorescence images of Edu in H_2O_2-stimulated CMs to assess the rescue effectiveness of MitoQ10 on CM proliferation after Tert knockdown (Edu+ CMs are indicated by arrows, *P <0.05; 238CMs from 18 images of 6 mice in WT+PBS group; 259 CMs from 18 images of 6 mice in WT+MitoQ10 group; 227 CMs from 18 images of 6 mice in $Tert^{flox/flox}$ *Myh6-Cre*+PBS group; 214 CMs from 18 images of 6 mice in $ert^{flox/flox}$ *Myh6-Cre*+MitoQ10 group). **G**: Assessment of Edu in H_2O_2-stimulated CMs to assess the rescue effectiveness of MitoQ10 on CM proliferation after Tert knockdown (*P <0.05; n=6 cell samples per group). **H:** Immunofluorescence images of pH3 in CMs to assess the rescue effectiveness of MitoQ10 on CM proliferation after H_2O_2 treatment (pH3+ CMs are indicated by arrows, *P <0.05; 201 CMs from 18 images of 6 mice in PBS group; 224 CMs from 18 images of 6 mice in H_2O_2 group; 196 CMs from 18 images of 6 mice in PBS+MitoQ10 group; 179 CMs from 18 images of 6 mice in H_2O_2+MitoQ10 group). Error bars indicate SD of six biological repeats. An unpaired *t*-test in **C-D**, one-way ANOVA in **B/E-H** and two-way ANOVA in **A** were utilized to determine the statistical significance.

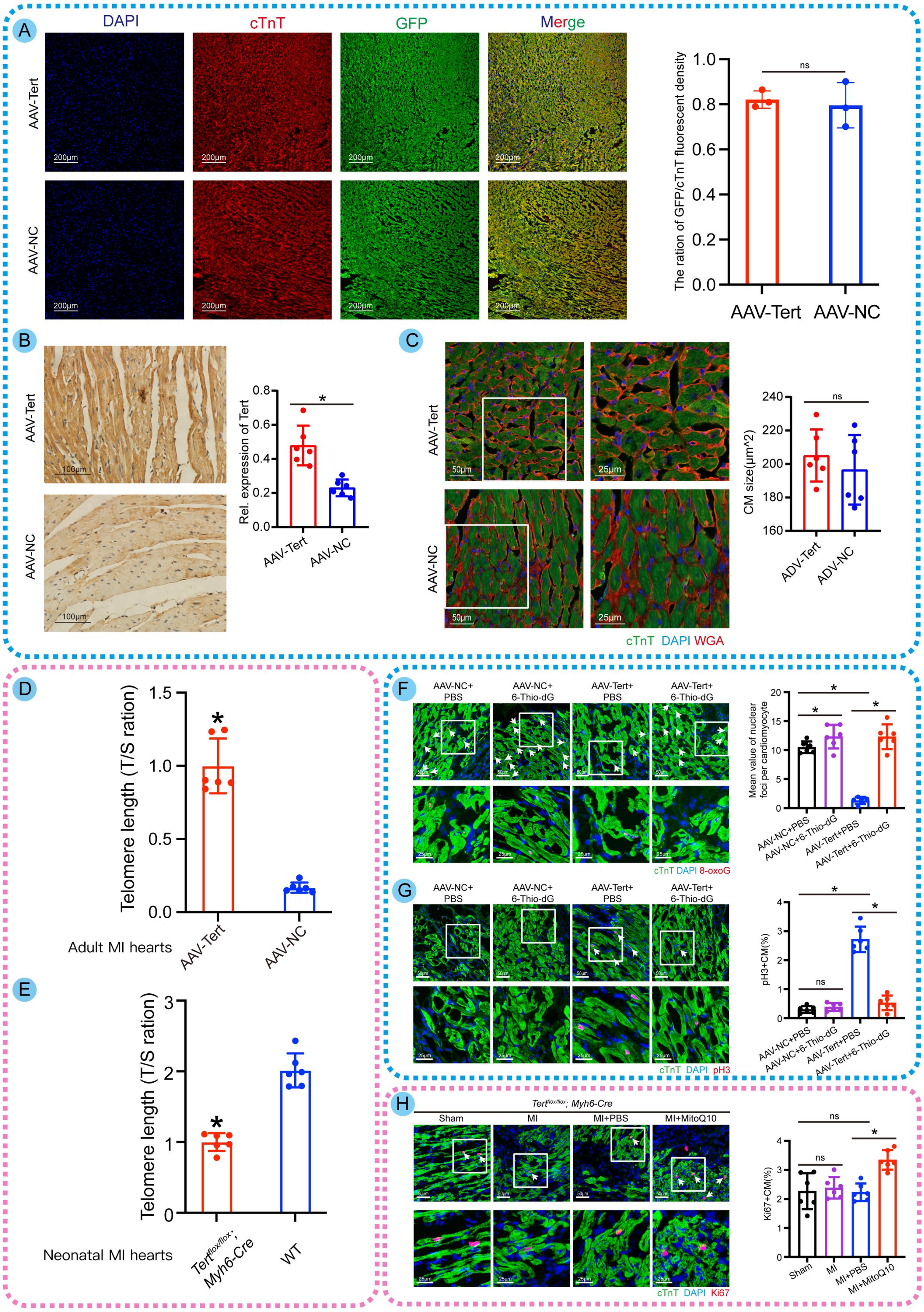

Supplemental Figure 4: Figures related to Figure 3.

A:Immunofluorescence staining images of AAV9-Tert overexpression vector carrying cTnT-specific promoter with GFP and empty vector after in vivo transfection into the heart. The right panel shows the statistical graph of effective infection of

myocardial tissue by the vectors(*P <0.05; n=3 mice per group). **B:** Immunofluorescence images of WGA in myocardial tissues from adult mice after Tert overexpression in vivo to assess the size of CMs (ns P>0.05; n=6 slices from 6 mice per group). **C:** Immunohistochemical images of Tert in myocardial tissues and quantitative analysis after Tert overexpression in vivo (*P <0.05; n=6 slices from 6 mice per group). **D** and **E**: Telomere lengths of heart tissues from adult mice with Tert overexpression and neonatal *Tert*$^{flox/flox}$*; Myh6*-Cre mice (*P <0.05; n=6 heart samples per group). **F:** Immunofluorescence images of 8-oxoG and quantitative analysis in each group of adult mice after various treatments (8-oxoG is indicated by arrows, *P <0.05; n=6 slices from 6 mice per group). **G:** Immunofluorescence images of pH3 and quantitative analysis in each group of adult mice after various treatments (pH3 is indicated by arrows, *P <0.05; n=6 slices from 6 mice per group). **H:** Immunofluorescence images of Ki67 in myocardial tissue and quantitative analysis. In *Tert*$^{flox/flox}$*; Myh6*-Cre mice, only MitoQ10 treatment promoted proliferation of CM (Ki67+ CMs are indicated by arrows, *P <0.05, ns P>0.05; n=6 slices from 6 mice per group). Error bars indicate SD of three biologcail repeats in **A** and that of six biological repeats in **B-H**. An unpaired *t*-test in **A-E** and one-way ANOVA in **F-H** were utilized to determine the statistical significance.

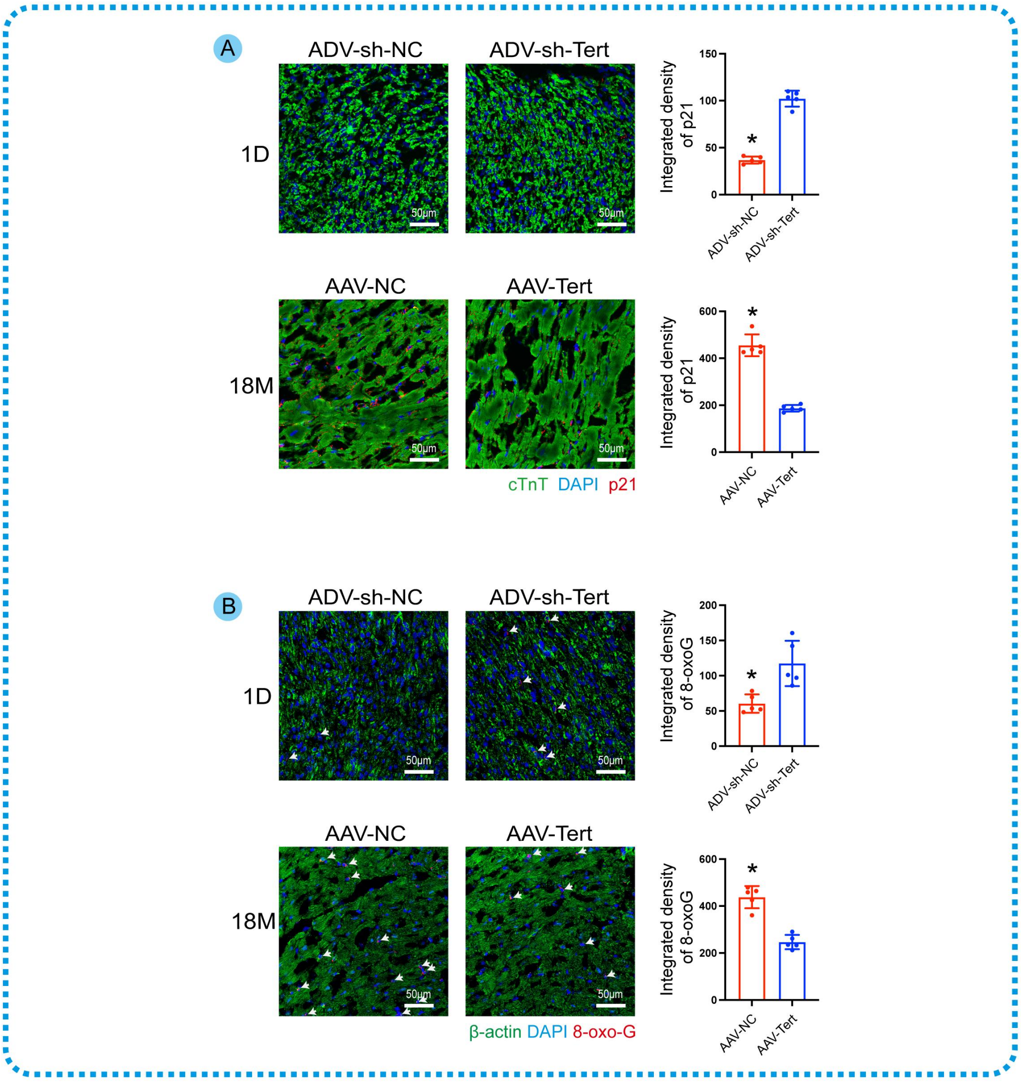

Supplemental Figure 5: Figures related to Figure 3.

A: Immunofluorescence images and quantitative analysis of p21 in frozen heart sections from normal neonatal mice and adult mice with Tert knockdown and overexpression, respectively (*P <0.05; n=5 slices from 5 animals per group). **B**:

Immunofluorescence images of 8-oxoG in frozen heart sections from normal neonatal mice and adult mice with Tert knockdown and overexpression, respectively. (*P <0.05; n=5 slices from 5 animals per group). Error bars indicate SD of five biologcail repeats. An unpaired *t*-test was utilized to determine the statistical significance.

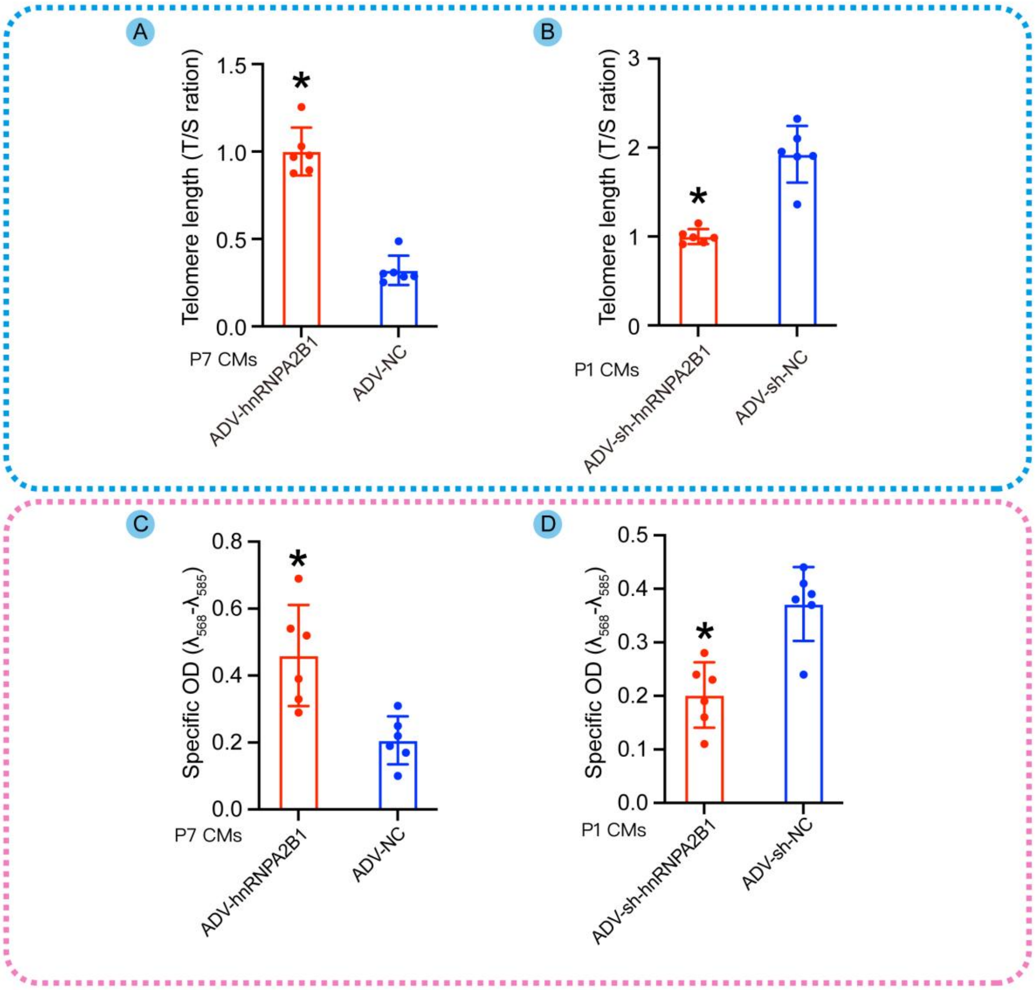

Supplemental Figure 6: Figures related to Figure 5.

A and **B**: Telomere lengths of P7 CMs with hnRNPA2B1 overexpression and P1 CMs with hnRNPA2B1 knockdown (*P <0.05 vs. ADV-NC or ADV-sh-NC; n=6 cell samples per group). Error bars indicate SD of six biological repeats. **C** and **D**:

Assessment of Edu in P7 CMs with hnRNPA2B1 overexpression and P1 CMs with hnRNPA2B1 knockdown (*P <0.05 vs. ADV-NC or ADV-sh-NC; n=6 cell samples per group). Error bars indicate SD of six biological repeats. An unpaired *t*-test was utilized to determine the statistical significance.

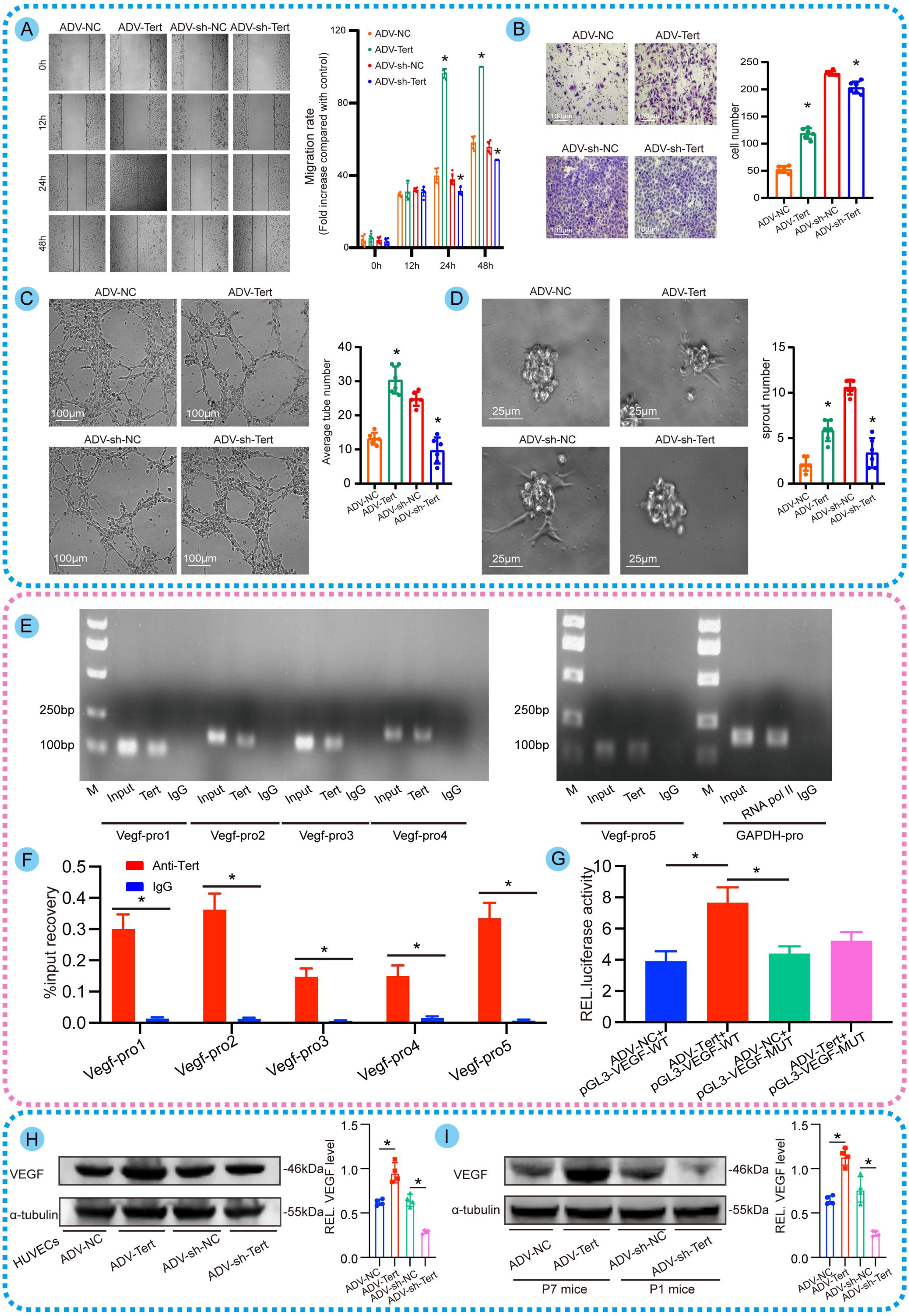

Supplemental Figure 7: Tert directly binds to the VEGF promoter and promotes angiogenesis.

A: Scratch assay for Tert overexpression and knockdown in HUVEC (*P <0.05 vs. ADV-NC or ADV-sh-NC; n=6 cell samples per group). **B:** Transwell assay for Tert

overexpression and knockdown in HUVEC in order to determine how Tert affects the invasion ability of endothelial cells (*P <0.05 vs. ADV-NC or ADV-sh-NC; n=6 cell samples per group). **C:** Tube-formation assay for Tert overexpression and knockdown in HUVEC, respectively, to determine whether Tert promotes neovascularization (*P <0.05 vs. ADV-NC or ADV-sh-NC; n=6 cell samples per group). **D:** Spheroid-sprouting assay for Tert overexpression and knockdown in HUVEC, respectively confirmed that Tert promotes endothelial cell sprouting, implying a positive effect of Tert on angiogenesis (*P <0.05 vs. ADV-NC or ADV-sh-NC; n=6 cell samples per group). **E:** Agarose gel electrophoresis plot of CHIP-qPCR showing that anti-Tert antibody enriches the five different genes of the predicted VEGF promoter. **F:** Normalized CHIP-qPCR results using the percent input method (*P <0.05 vs. IgG group; n=3 cell samples per group). Tert significantly immunoprecipitates VEGF promoters. **G:** Representative results of luciferase reporter assay, showing that the highest luciferase expression was observed in HUVEC co-transfected with ADV-Tert and the reporter plasmid, while the other groups received reduced luciferase levels (*P <0.05; n=3 cell samples per group). **H:** Western blotting assay for detection of VEGF in HUVEC after overexpression and knockdown of Tert (*P <0.05; n=4 cell samples per group, the experiment was replicated in the laboratory a total of three times). **I:** Western blotting assay for detection of VEGF in hearts from P7 mice with Tert overexpression and P1 mice with Tert knockdown (*P <0.05; n=4 cell samples per group, the experiment was replicated in the laboratory a total of three times). Error bars indicate SD of six biological repeats in **A-D**, that of three biological repeats in **F-G** and that of four biological repeats in **H-I**. A one-way ANOVA in **B-D/G-I** and two-way ANOVA in **A/F** were utilized to determine the statistical significance.

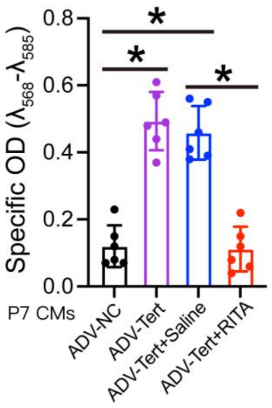

Supplemental Figure 8: Figures related to Figure 8.

Assessment of Edu in P7 CMs with different treatments (*P <0.05; n=6 cell samples per group). Error bars indicate SD of six biological repeats. A one-way ANOVA was utilized to determine the statistical significance.

Uncropped blots

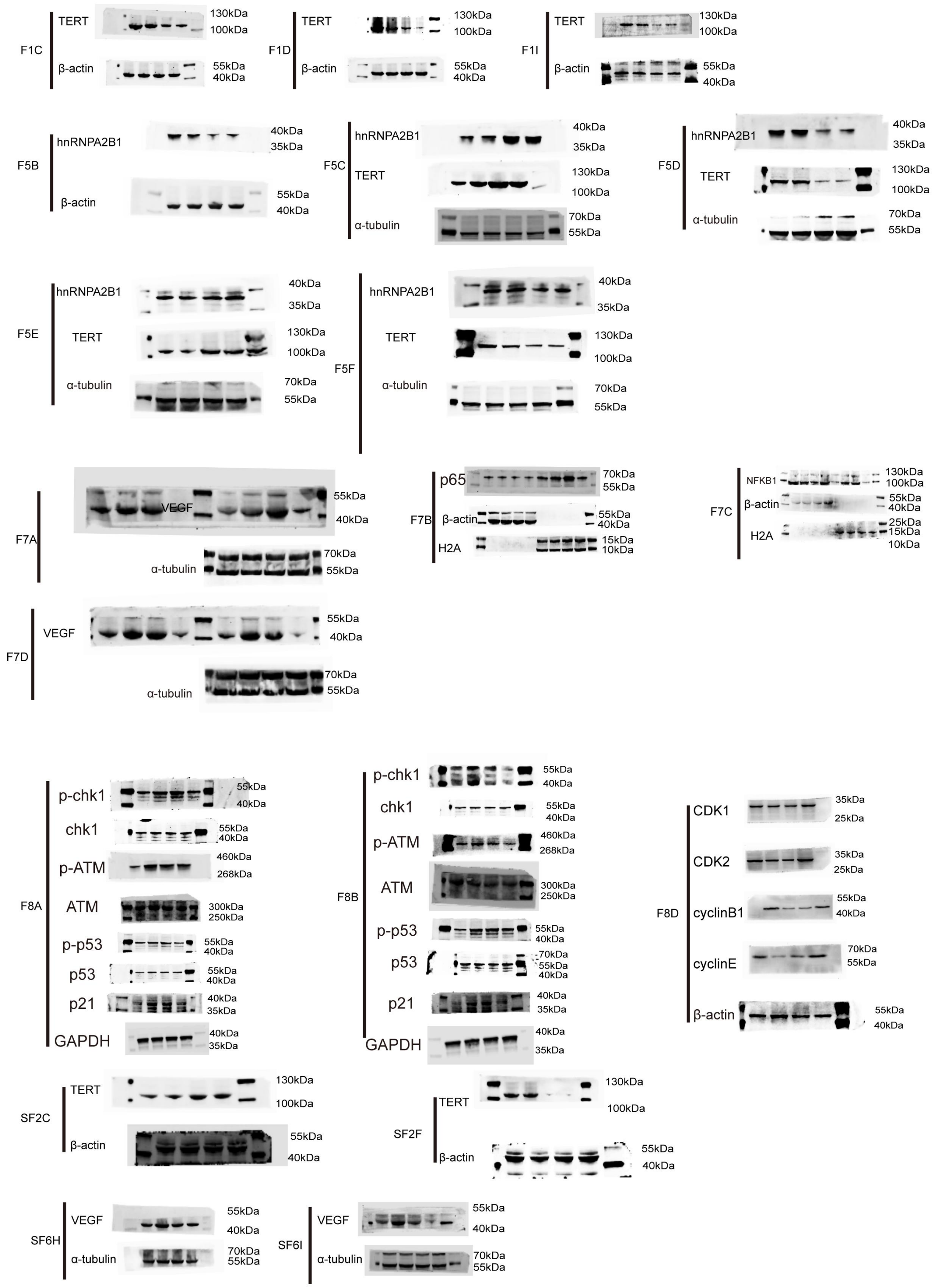

Supplement: Supplementary file 4 — uncropped blots and supplementary figures and legends [file 41420_2024_2135_MOESM4_ESM.pdf]
